# Efficacy of Neurostimulations for Upper Extremity Function Recovery after Stroke: A Systematic Review and Network Meta-Analysis

**DOI:** 10.3390/jcm11206162

**Published:** 2022-10-19

**Authors:** Tao Xue, Zeya Yan, Jiahao Meng, Wei Wang, Shujun Chen, Xin Wu, Feng Gu, Xinyu Tao, Wenxue Wu, Zhouqing Chen, Yutong Bai, Zhong Wang, Jianguo Zhang

**Affiliations:** 1 Department of Neurosurgery, Beijing Tiantan Hospital, Capital Medical University, Beijing 100070, China; 2 Department of Neurosurgery, The First Affiliated Hospital of Soochow University, Suzhou 215006, China; 3 Department of Neurology, Beijing Tiantan Hospital, Capital Medical University, Beijing 100070, China; 4 Department of Neurosurgery, Soochow Ninth Hospital, Suzhou 215124, China; 5 Department of Neurosurgery, Beijing Neurosurgical Institute, Beijing 100061, China; 6 Beijing Key Laboratory of Neurostimulation, Beijing 100054, China

**Keywords:** stroke, neurostimulation, upper extremity, network meta-analysis, Fugl-Meyer assessment

## Abstract

Background: Neurostimulations for the post-stroke recovery of upper extremity function has been explored in previous research, but there remains a controversy about the superiority of different neurostimulations. Methods: Randomized controlled trials (RCTs) were searched in MEDLINE, Embase, Cochrane Library and ClinicalTrials.gov, from 1 January 2000 to 1 June 2022. A conventional pair-wise meta-analysis with a random-effect model was used to evaluate direct evidence. Bayesian random effect models were used for network meta-analysis. The grading of the recommendations assessment, development and evaluation (GRADE) approach was applied to assess the clinical quality of the results. Results: A total of 88 RCTs, which enrolled 3491 participants, were included. For the Fugl-Meyer Assessment-Upper Extremity score change from the baseline to the longest follow-up, the following interventions showed a significant difference: VNS (MD = 4.12, 95%CrI: 0.54 to 7.80, moderate certainty), cNMES (MD = 3.98, 95%CrI: 1.05 to 6.92, low certainty), FES (MD = 7.83, 95%CrI: 4.42 to 11.32, very low certainty), drTMS (MD = 7.94, 95%CrI: 3.71 to 12.07, moderate certainty), LFrTMS (MD = 2.64, 95%CrI: 1.20 to 4.11, moderate certainty), HFrTMS (MD = 6.73, 95%CrI: 3.26 to 10.22, moderate certainty), and iTBS combined with LFrTMS (MD = 5.41, 95%CrI: 0.48 to 10.35, moderate certainty). Conclusions: The neurostimulations above the revealed significant efficacy for improving the upper limb function after stroke eased the suffering of the patient.

## 1. Introduction

Stroke is a serious cerebrovascular disease in which an artery supplying the brain becomes occluded or haemorrhaged [1]. According to previous global research, there were nearly 101 million people affected by stroke and 6.55 million deaths from stroke in 2019. It therefore represents a social and economic burden on individuals and families [2]. More than 80% of stroke survivors have been affected by hemiparesis of the contralateral limbs, and the probability of recovery in the upper extremity is <65% of that of the lower extremity [3,4,5,6]. Therefore, it is no exaggeration to say that the degree of upper extremity recovery could be the main clinical predictor of the rest of a patient’s life [7].

Regrettably, only 20% of the stroke survivors who received conventional physical rehabilitation return to normal life [8,9]. With advances in technology, neurostimulation technologies like neuromuscular electrical stimulation (NMES), transcranial direct current stimulation (tDCS), repetitive transcranial magnetic stimulation (rTMS), etc., have been proven to show significant efficacy for the recovery of upper limb hemiplegia after stroke [10,11,12,13,14,15,16]. While many systematic reviews have analyzed the efficacy of one technique compared to another, or different forms of the same kind, none of them systematically compared all neurostimulation technologies applied to the upper limb hemiplegia recovery after stroke. Therefore, we prepared this network meta-analysis and the conclusion of our research may be a more effective choice in clinical practice.

## 2. Materials and Methods

### 2.1. Study Protocol

This study protocol was registered in PROSPERO (CRD42021284405). Our research followed the guidelines of the Preferred Reporting Items for Systematic Reviews and Meta-Analyses (PRISMA) extension statement for NMA [17,18]. Additionally, we evaluated the quality and clinical significance of our research results following the grading of recommendations assessment, development and evaluation (GRADE) approach [19].

### 2.2. Eligibility Criteria

The inclusion criteria are as follows: (1) Study type, RCT; (2) restriction of language, i.e., English; (3) participants, adults ≥18 years with a history of unilateral stroke, whether ischemic or haemorrhagic; (4) interventions, vagus nerve stimulation (VNS), transcutaneous auricular VNS (taVNS), MCS, cyclic NMES (cNMES), EEG-triggered NMES (ENMES), functional electrical stimulation (FES), somatosensory electrical stimulation (SES) or transcutaneous electrical nerve stimulation (TENS), low frequency rTMS (LFrTMS), high frequency rTMS (HFrTMS), dual rTMS (drTMS), intermittent theta burst stimulation (iTBS), continuous TBS (cTBS), repetitive peripheral magnetic stimulation (rPMS) or functional magnetic stimulation (FMS), anodal tDCS (atDCS), cathodal tDCS (ctDCS), dual tDCS (dtDCS) and rehabilitation only (control); and (5) outcomes, at least evaluated the Fugl-Meyer Assessment-Upper Extremity (FMA-UE) score, which is considered the international criterion of assessing upper extremity motor paralysis [20].

The exclusion criteria are as follows: (1) Study type, conference abstracts, comments, reviews, protocols, and meta-analyses; (2) participants: <18 years or with neurodegenerative disorders, medical or psychiatric disorders, other intracranial diseases (i.e., intracranial space-occupying lesion), and contraindications to neurostimulation according to different types; and (3) interventions and control, the combination of one neurostimulation and specific therapy (i.e., mirror therapy, virtual reality technology) compared to rehabilitation or neurostimulation only.

### 2.3. Search Strategy

To identify the relevant literature, two investigators (TX and ZYY) searched MEDLINE, Embase, the Cochrane Library, and the Clinicaltrials.gov for published articles from 1 January 2000, to 1 June 2022, independently. The full search strategies applied to different databases are available in the Supplemental Materials (part A). The investigators also screened the relevant articles, such as systematic reviews and meta-analyses, to ensure the completeness of the included study.

### 2.4. Study Selection and Data Collection

**Figure 1:**
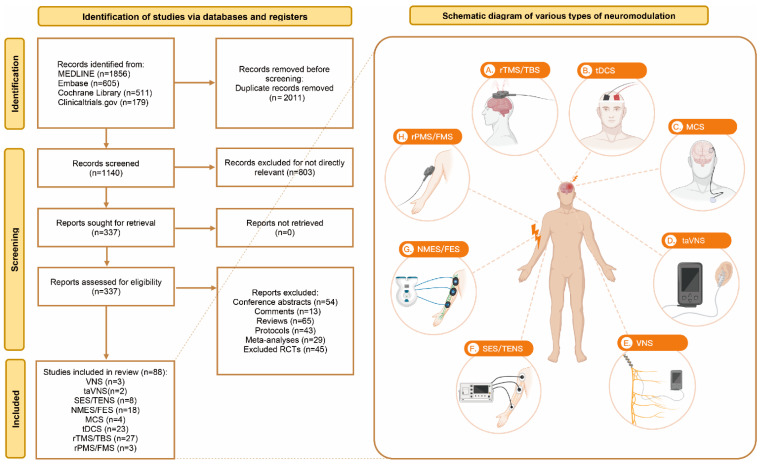
Study identification process and diagram of various types of neurostimulations.

Two investigators (TX and ZYY) assessed the eligibility of all the records searched from four databases according to the criteria above. Duplicates and articles, such as conference abstracts and comments, were excluded using EndNote X9 (Clarivate Analytics, Philadelphia, PA, USA). Further details of the selection process are shown in the flow diagram (Figure 1). In addition, we summarized the definitions and characteristics of each subtype of neurostimulation in Table 1. After selection and evaluation, the basic information of the included studies was extracted and shown in the Supplemental Materials (part B and C). During this process, any disagreements were discussed with the third investigator (JHM) to make the ultimate decision.

### 2.5. Outcomes

This research judged the following outcomes as crucial: Change in FMA-UE score from baseline to the longest follow-up (LFU), end of treatment (EOT), one month, and three months. The following outcomes were judged to be important, but not crucial: Change in Action Research Arm Test (ARAT) and Box and Block Test (BBT) from baseline to the longest follow-up. Given that the number of adverse events in most studies was zero, we gave up on the calculation of safety outcomes. Information on safety outcomes is available in the Supplemental Materials (part C).

### 2.6. Statistical Analysis

NMA was performed based on a Bayesian framework by applying the Markov chain Monte Carlo methods in the R software 3.5.2 (R Foundation for Statistical Computing, Vienna, Austria) using the ‘gemtc’ package; this involved four chains with over-dispersed initial values and Gibbs sampling based on 50,000 iterations after a burn-in phase of 20,000 iterations [21]. The efficacy of numerous neurostimulations was reported through the mean difference (MD) with a 95% credible interval (CrI) and was compared via direct and indirect evidence. As NMA is based on the consistency between direct and indirect evidence, we used a node-splitting method to confirm the consistency between direct and indirect evidence, estimating the local inconsistency, with a *p* value >0.05 meaning good consistency. The deviance information criterion (DIC) was used to assess the goodness-of-fit of the network model. The DIC values between consistent and inconsistent models were compared to evaluate global inconsistency, with a lower DIC value indicating a better model.

To further analyze the NMA heterogeneity, we conducted a meta-regression analysis for the change in FMA-UE compared to a random consistent model, including the following concomitant variables: Mean age, percentage of females, mean FMA-UE at baseline, percentage of haemorrhagic stroke, sample size, and mean duration since the stroke. Meanwhile, we performed a sensitivity analysis of NMA to evaluate the small study effect by comparing the DIC difference between the fixed model and the random model. A difference in DIC < 5 indicates no obvious influence.

The surface under the curve ranking area (SUCRA) was calculated to sequence the efficacy of the interventions, with a larger area under the curve indicating a better rank for the therapy. To make the results more explicit, we rearranged the SUCRA for 23 types of therapies according to the four primary outcomes. The mean cumulative probabilities with each intervention represent the ranking probabilities to some extent.

The GRADE approach was used to assess the direct comparison of pair-wise analyses and to evaluate the direct and indirect evidence of NMA, rating the certainty of the results of each comparison as high, moderate, low, or very low. We classified the interventions into three categories: The most effective, the least effective and inferior to the most effective/superior to the least effective according to the magnitude of effects, adapting the minimally contextualised framework [19]. Additionally, we used ‘Maybe’ to mark the low or very low certain evidence.

### 2.7. Risk of Bias

The risk of bias for the included studies was assessed with Review Manager 5.4 software (The Nordic Cochrane Center, Copenhagen, Denmark) and the Cochrane Collaboration’s risk-of-bias tool. Six fields were evaluated as follows: Selection bias, performance bias, detection bias, attrition bias, reporting bias, and other potential biases. Each bias criterion was classified into three evaluations: Low, unclear, and high. Funnel plots were evaluated to analyze the publication bias for outcomes. Conflicts were resolved through discussion with the third author (WW) until agreement was reached.

## 3. Results

### 3.1. Search Strategies and Study Characteristics

A total of 3151 studies were identified from four databases, and 2011 records were removed before screening due to duplication. Then, by performing a simple screening for titles and abstracts, we excluded 803 articles as not being directly relevant. Among the remaining 337 reports assessed for eligibility, 54 conference abstracts, 13 comments, 65 reviews, 43 protocols, and 29 meta-analyses and RCTs inconsistent with the abovementioned eligibility criteria were excluded. Finally, 88 RCTs containing eight major kinds of neurostimulation were included in our research and the catalogue of the included studies is declared in the Supplementary Materials (part D), including 3491 patients. The study selection process and a schematic diagram of neurostimulation are shown in Figure 1.

### 3.2. FMA-UE

**Figure 2:**
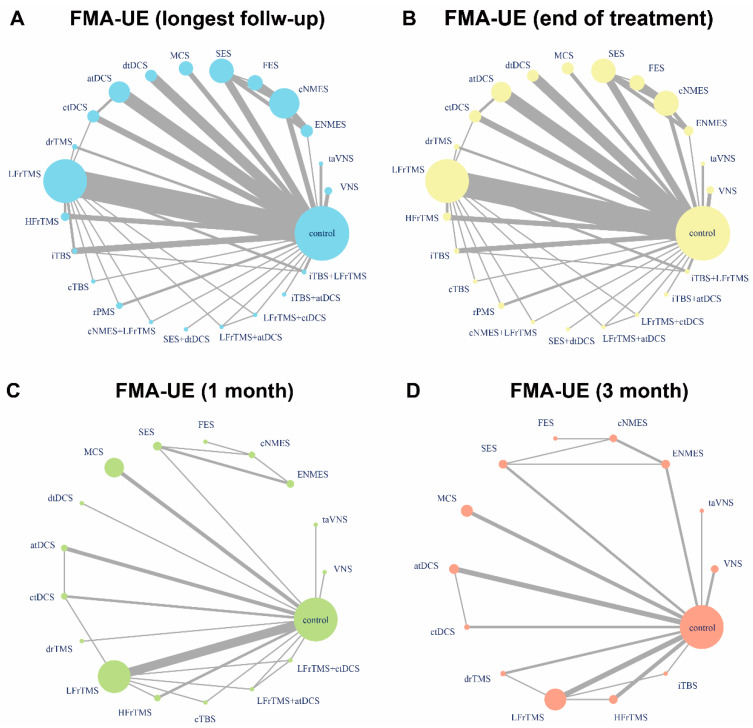
Network plots of available direct comparisons. Change in FMA-UE from baseline to longest follow-up (**A**), the end of treatment (**B**), one month (**C**), and three months (**D**). Each node (solid circle) stands for neurostimulation or rehabilitation only. The size of the nodes is proportional to the number of participants (i.e., sample size) involved in the specific intervention. The solid lines link treatments with direct comparison with the thickness proportional to the number of trials.

**Figure 3:**
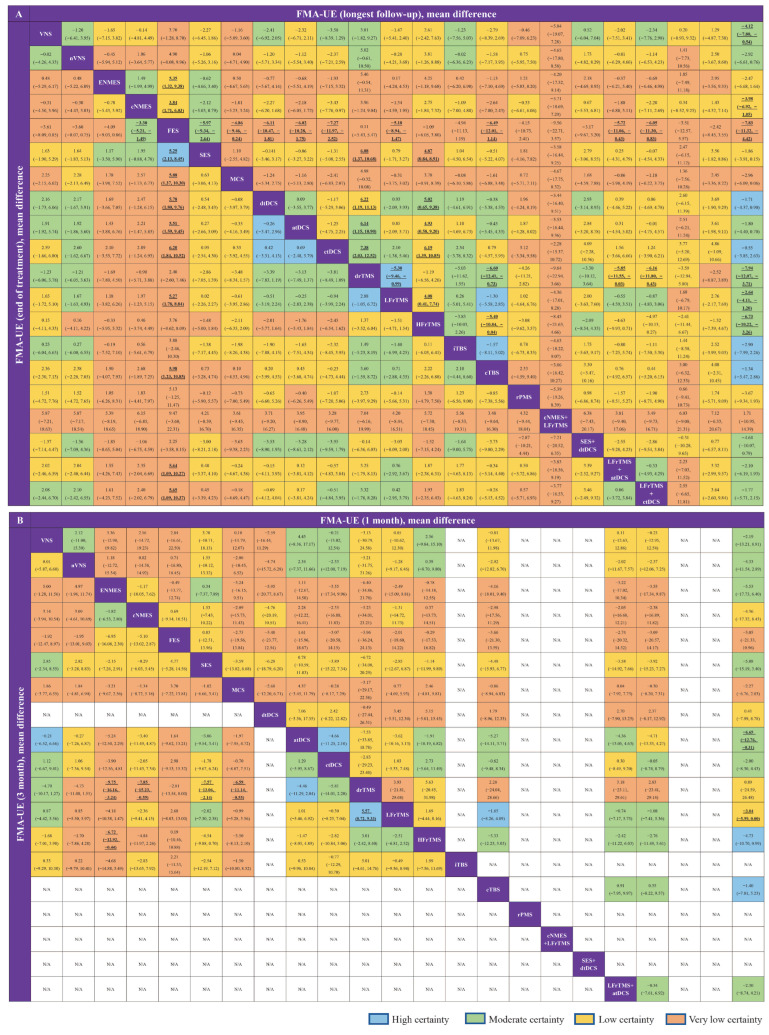
League tables of the outcome analysis. Change in FMA-UE from baseline to longest follow-up, end of treatment (**A**), one month, and three months (**B**). The league tables show the relative effects of each neurostimulation and rehabilitation only (the intervention on the column to the intervention of the row). The relative effects are measured as the mean difference for FMA-UE change with 95% CrI. Bold indicates statistical significance. The color of each cell indicates the certainty of evidence according to the Grading of recommendations, assessment, development, and evaluation.

Figure 2 shows the network plots for the score change of FMA-UE from baseline to the LFU, EOT, one month, and three months. The network estimates of all comparisons are illustrated in Figure 3. In addition, pair-wise meta-analysis and sensitivity analysis of FMA-UE was also conducted (Table 2) and the details were shown in the Supplementary Materials (part E and F). Further details of the GRADE evaluation can be found in the Supplementary Materials (part G).

**Figure 4:**
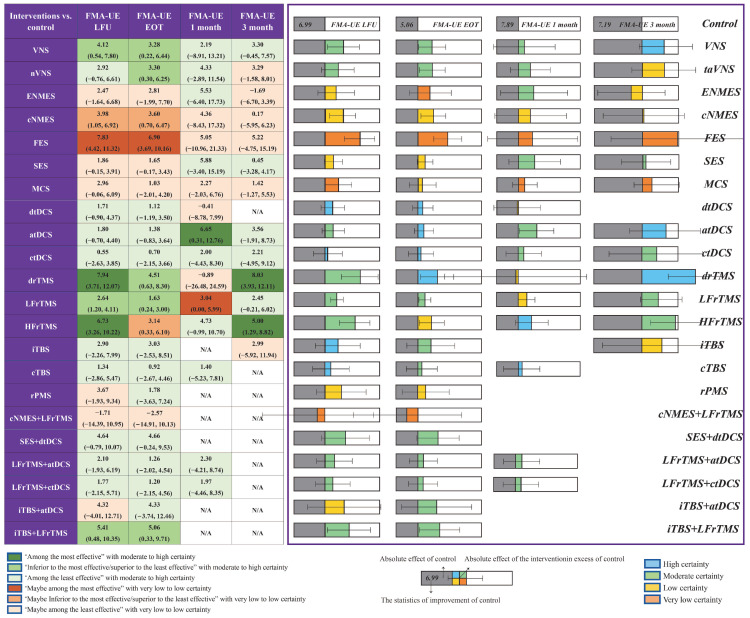
Summary of the absolute effects of neurostimulation compared to rehabilitation only. This pooled effect represents how much FMA-UE a person with stroke can expect to improve through rehabilitation alone and the absolute effect of the intervention excess of rehabilitation.

**Figure 5:**
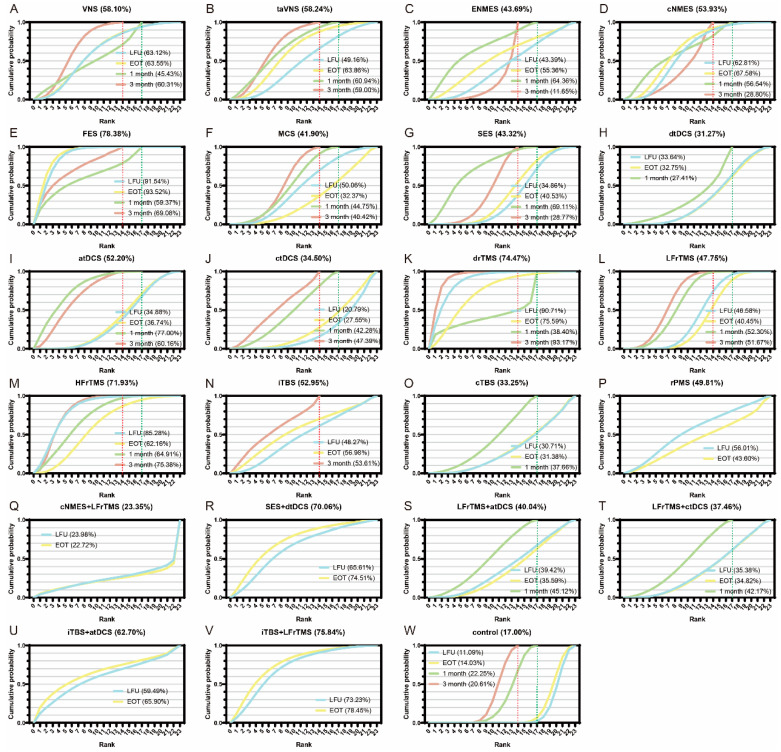
Modified surface under curve ranking area (SUCRA) value. Mean probability of each intervention with a specific rank for outcomes. A larger mean ranking value indicates a better rank for the intervention.

Figure 4 reveals the classification of interventions compared to rehabilitation only. For the primary outcomes of FMA-UE LFU, interventions such as VNS (4.12, 95%CrI 0.54 to 7.80; moderate certainty), cNMES (3.98, 95%CrI 1.05 to 6.92; low certainty), FES (7.83, 95%CrI 4.42 to 11.32; very low certainty), drTMS (7.94, 95%CrI 3.71 to 12.07; moderate certainty), LFrTMS (2.64, 95%CrI 1.20 to 4.11; moderate certainty), HFrTMS (6.73, 95%CrI 3.26 to 10.22; moderate certainty), and iTBS + LFrTMS (5.41, 95%CrI 0.48 to 10.35; moderate certainty) showed a significant difference, with FES, drTMS, and HFrTMS proving to be among the most effective compared to rehabilitation. As for the outcomes of FMA-UE EOT, VNS (3.28, 95%CrI 0.22 to 6.44; moderate certainty), taVNS (3.30, 95%CrI 0.30 to 6.25; moderate certainty), cNMES (3.60, 95%CrI 0.70 to 6.47; low certainty), FES (6.90, 95%CrI 3.69 to 10.16; very low certainty), drTMS (4.51, 95%CrI 0.63 to 8.30; high certainty), LFrTMS (1.63, 95%CrI 0.24 to 3.00; moderate certainty), HFrTMS (3.14, 95%CrI 0.33 to 6.10; low certainty), and iTBS + LFrTMS (5.06, 95%CrI 0.33 to 9.71; moderate certainty) showed statistical superiority compared to rehabilitation alone. FES was among the most effective. After treatment for one month, atDCS (6.65, 95%CrI 0.31 to 12.76; moderate certainty) and LFrTMS (3.04, 95%CrI 0.00 to 5.99; low certainty) showed obvious advantages of improving the FMA-UE. Moreover, at three months, drTMS (8.03, 95%CrI 3.93 to 12.11; high certainty) and HFrTMS (5.00, 95%CrI 1.29 to 8.82; moderate certainty) revealed significant statistical differences to with MID, both being among the most effective. According to the modified SUCRA format, the mean cumulative probability of FES ranks first (78.38%); iTBS + LFrTMS (75.84%), and drTMS (74.47%) came in second and third, respectively (Figure 5).

### 3.3. Safety

Most of the neurostimulations were safe and well tolerated. Of the included studies, non-invasive interventions, such as rTMS, tDCS, and taVNS, almost mentioned or reported no adverse events (AEs). Furthermore, most of the adverse events mentioned were common and included skin irritation, rash, and headache. The adverse events of invasive neurostimulation such as MCS and VNS usually involve infection and temporary pain, which are related to the invasive operation. Details on the AEs of the 88 studies are summarized in the Supplementary Materials (part C).

### 3.4. ARAT and BBT

The ARAT and BBT results are shown in our Supplementary Materials (part H and I). None of neurostimulation therapy showed significant difference compared to rehabilitation only.

### 3.5. Network Meta-Regression and Sensitivity Analysis

The meta-regression analysis demonstrated age, sex, baseline of mean FMA-UE, type of stroke, sample size and mean time since stroke did not influence the outcome for the change of FMA-UE (part J). As for the NMA sensitivity analysis, we found no obvious difference between the fixed model and random model. The DIC difference of all the results were <5 (part K).

### 3.6. Network Heterogeneity and Consistency

The results showed no obvious local inconsistency and heterogeneity (part L, M, N and O). Further construction of the global inconsistency model also showed that the DIC difference between them was <10 (part K), indicating that the results of the consistency model are reliable.

### 3.7. Risks of Bias

The risks of bias for all the included studies are shown on part P of the Supplementary Materials. The major risks of bias concentrate on blinding of participants and personnel (performance bias), with nearly 40% showing a high risk of bias. The analysis results of selection bias showed no high risk of bias, with a few unclear risks of bias. As for the remaining fields of bias, including detection, attrition reporting, and other bias, the total percentage of risk of bias was less than 20%. More details are shown in the Supplementary Materials, including the funnel plot for publication bias, which shows no obvious bias (part Q).

## 4. Discussion

Generally, we conducted this NMA based on 88 RCTs, which enrolled 3491 participants to distinguish the efficacy of neurostimulations and the combination of them. VNS, drTMS, LFrTMS, HFrTMS, and iTBS + LFrTMS revealed significant improvements in neurological function after stroke, with high or moderate certainty evidence for the primary results of FMA-UE LFU. Additionally, cNMES and FES also showed superiority to a certain extent, as compared to rehabilitation alone, albeit with low or very low certainty evidence. Moreover, drTMS and HFrTMS are among the most effective, and FES may be among the most effective based on our data analysis results.

Both types of VNS have proven effects in the rehabilitation of neurological diseases [22,23]. The latest meta-analysis conducted by Xie et al. systematically confirmed the efficacy of VNS and taVNS [24]. This is largely consistent with our pair-wise analysis results. Certainly, the evidence of four FMA-UE outcomes in our research is nearly all moderate certainty, demonstrating the clinical reliability of both. It is worth noting that the efficacy of taVNS has recently been confirmed by Liu et al. again. Moreover, taVNS seems to be more promising because it is easy to use at home and could be proposed as a complementary treatment [25].

tDCS is one of the most widely investigated, non-invasive electrical brain stimulations [26]. However, only atDCS revealed a significant improvement in the results of FMA-UE one month with moderate certainty, both in the results of pair-wise meta-analysis and NMA. As has been proven by Stephen Bornheim et al., atDCS seems to be an effective technique to accelerate functional recovery when applied in the acute stages of stroke. They used the change of the Wolf Motor Function Test as the primary outcome, which revealed significant differences at one month [27]. However, according to the conclusion drawn by Bernhard Elsner et al., ctDCS is a more promising option to improve the capacity in activities of daily living (ADL), while all tDCS seem invalid for improving FMA-UE [28]. Considering these results, more research, including more comprehensive evaluation criteria, need to be conducted.

rTMS is a non-invasive magnetic stimulation that modulates cortical excitability [29,30,31,32]. Through our NMA, we found that HFrTMS, LFrTMS, and drTMS all revealed a statistical difference compared to rehabilitation alone. Meanwhile, these showed almost confirmed efficacy in FMA-UE results through pair-wise meta-analysis. Moreover, according to SUCRA results, drTMS is superior to others, followed by HFrTMS. It is worth noting that the time from the onset of stroke to the evaluation of outcomes in these modulations is <3 months for all. As the previous literature suggests, there is a natural biological recovery process after stroke, apparently in the first one to three months, and primitive clinical, electrophysiological, and imaging parameters can predict ultimate clinical outcomes in a large number of patients [6,33]. Moreover, some research demonstrated that the Fugl-Meyer scores five days after stroke predict the score at six months post-stroke [34,35]. Thus, results of the current study are only statistically significant and we cannot ignore the natural recovery effect after stroke.

Unexpectedly, TBS, as a novel form of rTMS, is supposed to have more rapid and powerful effects than rTMS, but revealed no statistical advantage with the available network comparison of LFrTMS, irrespective of iTBS or cTBS [36]. In pair-wise analysis, somewhat differently, cTBS showed efficacy for the recovery of stroke to some degree, but with a lack of long-term research beyond three months. As for the combined application of iTBS and LFrTMS, only one of the included studies evaluated its efficacy, which is insufficient to come to any reliable conclusions.

As for cNMES and FES, despite showing some degree of significant difference, they do not appear to be as reliable as the neurostimulations above. In particular, FES has prominent efficacy compared to most neurostimulations, but with very low certainty simultaneously, ranking first in SUCRA results of FMA-UE. Moreover, regrettably, the lack of direct comparison between FES and rehabilitation means that no further pair-wise analysis is possible. Similarly, cNMES also shows no significant difference in pair-wise analysis. Hence, we cannot make further verification and more studies are needed to confirm their effectiveness. In addition, given that FES is a widespread modality used by rehabilitation specialists, FES may be more effective modalities for stroke and is a helpful dataset for those who treat poststroke patients.

eNMES, SES, MCS, dtDCS, and rPMS have proven efficacy according to previous literature, ranking in the middle level in this research, with no obvious statistical differences. We believe that reasons, such as lack of sufficient direct or indirect comparisons, may lead to inadequate verification of their efficacy. Therefore, we have reservations about the results of this component of our study.

Several limitations cannot be avoided in our analysis. First, as objective limitations of RCTs on neurostimulation, the sample size of major studies was restricted to those with more than 100 participants. In addition, the sample size differs considerably between the different neuromodulation modalities. For example, 33 for taVNS compared to 146 for HRrTMS. These may lead to a bias in inclusion and reduce the universality of the results in this paper. Second, in most of the included studies, the follow-up was <3 months; thus, we cannot explore the long-term effects of these therapies. Additionally, years since a stroke for the all modalities of TMS is very short, while it is much longer for VNS. This difference may influence the outcome. Then, considering the lack of direct head-to-head comparison between some neurostimulations, such as HFrTMS and drTMS, our conclusions based on indirect comparisons should be treated with caution. Moreover, head-to-head comparisons of the impact of a particular modality in a chronic stroke population with an acute stroke population will lead to divergent outcome results. Additionally, we cannot discount the possibility that the use of a "new" therapy will have a psychological impact on the patients being treated. Finally, the number of present studies comparing the combination of different neurostimulations was relatively low, which may lead to bias, and the results were unsatisfactory.

## 5. Conclusions

Through our analysis, VNS, taVNS, atDCS, drTMS, HFrTMS LFrTMS, cNMES, FES and iTBS + LFrTMS revealed significant efficacy to various degrees with different certainty levels. Our findings would be helpful for the clinical decisions made for the recovery of stroke. In the foreseeable future, more research directed at restorative neurostimulation may improve the prognosis of patients after stroke.

## Figures and Tables

**Table 1:** Characteristics of different neuromodulation techniques, including definition, sample size, age, gender, years since stroke, FMA-UE at baseline and side of hemiplegic paralysis.

Neuromodulation.	Subtype	Definition	Sample Size	Age, Mean ± SD	Female, *n* (%)	Years Since Stroke, Mean ± SD	FMA-UE, Mean ± SD	Hemiplegic Paralysis, Left/Right
1. VNS (vagus nerve stimulation)	1.1 VNS	A device is implanted into patient body to stimulate the cervical branch of vagus nerve directly through a simple surgery.	70	58.99 ± 10.88	25 (35.71%)	2.75 ± 2.13	34.57 ± 8.53	41/29
	1.2 taVNS	A noninvasive technique stimulates the other branch of the vagus nerve in body surface, like external auditory channel at the inner side of the vagus.	17	60.06 ± 13.29	8 (47.06%)	3.27 ± 6.54	19.47 ± 7.20	4/6
2. MCS (Motor cortex stimulation)	-	An invasive electrical stimulation which places the electrode at epidural area around the associated site of motor cortex activation through a craniotomy.	122	55.86 ± 11.17	52 (42.62%)	4.95 ± 5.49	36.91 ± 6.83	49/73
3. NMES/FES (Neuromuscular electrical stimulation /Functional electrical stimulation)	3.1 cNMES	This stimulation is provided by electrically activating hemiplegia muscle at a set frequency while the intensity at or above motor threshold. During the entire process, patient is generally a passive participant.	278	58.73 ± 12.48	107 (38.49%)	0.46 ± 1.53	27.29 ± 13.88	123/123
	3.2 ENMES	Patient is actively involved in the training and the electrical stimulation is provided when EMG signals generated by motion exceed a pre-set threshold.	113	57.47 ± 12.29	39 (34.51%)	1.04 ± 2.39	34.02 ± 15.52	57/55
	3.3 FES	It refers that tetanic muscle contractions of hemiplegia limb are induced to assist or reinstate some kinds of goal-directed movement, while patients or therapists could control the timing or intensity of stimulation.	156	55.8 ± 14.05	48 (30.77%)	0.70 ± 1.23	25.53 ± 11.39	72/63
4. SES/TENS (Somatosensory electrical stimulation/Transcutaneous nerve electrical stimulation)	-	An intervention involves low intensity electrical stimulation of peripheral nerves, which merely reaches the sensory threshold and below the motor threshold.	224	60.98 ± 13.95	102 (45.54%)	1.32 ± 2.01	30.47 ± 20.54	99/105
5. rTMS/TBS (Repetitive transcranial magnetic stimulation /Theta burst stimulation)	5.1 LFrTMS	A non-invasive magnetic stimulation modulates cortical excitability in stroke, and low-frequency rTMS (≤1 Hz) decreases the cortical excitability of the primary motor cortex of unaffected limb.	586	59.97 ± 12.84	206 (35.15%)	0.90 ± 2.33	34.69 ± 16.00	246/262
	5.2 HFrTMS	Similarly, high-frequency rTMS (≥5 Hz) facilitates the cortical excitability of the hemiplegic limb.	77	57.84 ± 9.13	24 (31.17%)	0.03 ± 0.04	28.24 ± 15.06	38/39
	5.3 drTMS	LF-rTMS applies to the unaffected side while HF-rTMS to the hemiplegic side for synergistic effect.	35	55.90 ± 8.89	5 (14.29%)	0.05 ± 0.01	38.14 ± 18.98	11/10
	5.4 iTBS	A variant of rTMS modulated ipsilesional primary motor cortex intermittently with a specific pattern of stimulation sequences in a shorter time.	49	59.7 ± 12.07	15 (30.61%)	0.45 ± 0.32	32.27 ± 16.55	17/20
	5.5 cTBS	Continuous theta burst stimulation brings down the excitability of the contralateral primary motor cortex for the rehabilitation of stoke.	7	61.3 ± 9.8	1 (14.29%)	1.21 ± 0.13	19.4 ± 14.2	2/5
6. rPMS/FMS (Repetitive peripheral magnetic stimulation /Functional magnetic stimulation)	-	A magnetic technology stimulates deep regions of muscles evoking muscle contraction with nearly no pain.	60	55.78 ± 13.02	19 (31.67%)	0.44 ± 1.19	27.55 ± 16.64	27/23
7. tDCS (Transcranial direct current stimulation)	7.1 atDCS	A body surface direct current stimulation places the anode slice on the motor cortex area of the affected side and facilitates the depolarization of neurons.	176	62.80 ± 11.70	70 (39.78%)	1.37 ± 1.99	28.65 ± 17.67	70/72
	7.2 ctDCS	On the contrary, cathodal tDCS is mounted on the the scalp surface of not damaged brain hemisphere, reducing the neuronal firing.	109	63.48 ± 10.13	45 (41.28%)	0.34 ± 0.93	22.45 ± 20.24	57/52
	7.3 dtDCS	The anode slice of tDCS device is mounted on the ipsilesional side while the cathode on the contralateral side at the same time to improve the rehabilitation of extremity function after stroke.	108	59.00 ± 11.26	37 (34.26%)	1.91 ± 1.54	36.79 ± 17.37	52/56

**Table 2:** Summary and detailed effect sizes from the pair-wise meta-analysis of efficacy outcomes from all the trials using the random effects models.

Outcomes	No. of Trials Contributing to the Meta-Analysis	No. of Participants Contributing to the Meta-Analysis	Effect Size		Heterogeneity			GRADE
			MD (95% CI)	*p* Value	I^2^ (%)	χ²	*p* Value	
**1.** **FMA-UE longest follow-up (compared with control)**								
VNS	3	145	**3.49 (1.56, 5.41)**	0.0004	0	1.12	0.57	⊕⊕⊕○ Moderate *
taVNS	2	33	**2.95 (0.90, 5.00)**	0.005	0	0.09	0.77	⊕⊕⊕○ Moderate *
ENMES	1	15	2.16 (−14.62, 18.94)	0.80	N/A	N/A	N/A	⊕⊕○○ Low *, ##
cNMES	4	111	4.30 (−0.38, 8.97)	0.07	45	5.41	0.14	⊕⊕○○ Low *, $
SES	6	252	**1.73 (0.73, 2.73)**	0.0007	23	6.47	0.26	⊕⊕○○ Low *, #
MCS	4	208	**2.63 (0.32, 4.95)**	0.03	28	4.19	0.24	⊕○○○ Very low **, #
dtDCS	8	205	1.48 (−0.09, 3.05)	0.06	0	2.86	0.90	⊕⊕⊕⊕ High
atDCS	10	361	1.64 (−1.50, 4.77)	0.31	45	16.44	0.06	⊕⊕⊕○ Moderate $
ctDCS	5	172	1.78 (−1.72, 5.29)	0.32	0	0.22	0.99	⊕⊕⊕⊕ High
drTMS	2	70	6.28 (−2.12, 14.68)	0.14	22	1.28	0.26	⊕⊕⊕○ Moderate #
LFrTMS	21	948	**2.99 (1.34, 4.63)**	0.0004	65	56.91	<0.0001	⊕⊕⊕○ Moderate $
HFrTMS	4	146	**7.11 (4.40, 9.82)**	<0.00001	0	1.49	0.68	⊕⊕⊕○ Moderate *
iTBS	5	98	3.10 (−1.90, 8.10)	0.22	0	1.93	0.75	⊕⊕⊕⊕ High
cTBS	1	13	**2.97 (1.26, 4.68)**	0.0007	N/A	N/A	N/A	⊕⊕⊕⊕ High
rPMS	2	82	1.66 (−4.15, 7.47)	0.58	0	0.51	0.47	⊕⊕○○ Low *, #
cNMES+LFrTMS	1	16	8.00 (−7.84, 23.84)	0.32	N/A	N/A	N/A	⊕○○○ Very low *, ##
SES+dtDCS	1	19	**4.64 (1.30, 7.98)**	0.006	N/A	N/A	N/A	⊕⊕⊕○ Moderate *
LFrTMS+atDCS	1	30	1.20 (−0.33, 2.73)	0.12	N/A	N/A	N/A	⊕⊕⊕○ Moderate *
LFrTMS+ctDCS	1	30	0.87 (−0.27, 2.01)	0.13	N/A	N/A	N/A	⊕⊕⊕○ Moderate *
iTBS+atDCS	1	24	4.33 (−2.93, 11.59)	0.24	N/A	N/A	N/A	⊕⊕○○ Low *, #
iTBS+LFrTMS	2	47	4.84 (−0.22, 9.89)	0.06	0	0.86	0.35	⊕⊕⊕○ Moderate #
**2.** **FMA-UE end of treatment (compared with control)**								
VNS	3	145	**2.83 (1.37, 4.30)**	0.0002	0	1.26	0.53	⊕⊕⊕○ Moderate *
taVNS	2	33	**3.54 (2.31, 4.77)**	<0.00001	0	0.42	0.52	⊕⊕⊕○ Moderate *
ENMES	1	15	3.96 (−13.51, 21.43)	0.66	N/A	N/A	N/A	⊕○○○ Very low *, ##
cNMES	3	65	4.28 (−1.74, 10.30)	0.16	63	5.37	0.07	⊕○○○ Very low *, #, $
SES	6	252	**1.70 (0.33, 3.07)**	0.01	45	9.02	0.11	⊕⊕○○ Low #, $
MCS	3	184	0.44 (−1.04, 1.93)	0.56	0	1.55	0.46	⊕⊕○○ Low **
dtDCS	8	205	0.95 (−0.53, 2.42)	0.21	0	5.83	0.56	⊕⊕⊕⊕ High
atDCS	10	361	0.80 (−1.10, 2.70)	0.41	10	9.98	0.35	⊕⊕⊕⊕ High
ctDCS	5	172	1.89 (−1.25, 4.81)	0.25	0	0.83	0.93	⊕⊕⊕⊕ High
drTMS	2	70	**5.47 (3.25, 7.69)**	<0.00001	0	0.34	0.56	⊕⊕⊕⊕ High
LFrTMS	19	823	**1.83 (0.69, 2.96)**	0.002	26	24.35	0.14	⊕⊕⊕○ Moderate #
HFrTMS	4	146	**3.21 (0.17, 6.25)**	0.04	34	4.56	0.21	⊕⊕○○ Low *, #
iTBS	4	84	2.62 (−3.06, 8.29)	0.37	0	1.62	0.65	⊕⊕⊕○ Moderate #
cTBS	1	13	**2.12 (0.40, 3.84)**	0.02	N/A	N/A	N/A	⊕⊕⊕○ Moderate #
rPMS	2	82	−0.23 (−6.82, 6.37)	0.95	11	1.12	0.29	⊕⊕○○ Low *, #
cNMES+LFrTMS	1	16	8.00 (−7.84, 23.84)	0.32	N/A	N/A	N/A	⊕○○○ Very low *, ##
SES+dtDCS	1	19	**4.64 (1.30, 7.98)**	0.006	N/A	N/A	N/A	⊕⊕⊕○ Moderate *
LFrTMS+atDCS	1	30	**0.80 (0.00 1.60)**	0.05	N/A	N/A	N/A	⊕⊕○○ Low *, #
LFrTMS+ctDCS	1	30	0.74 (−0.32, 1.80)	0.17	N/A	N/A	N/A	⊕⊕⊕○ Moderate *
iTBS+atDCS	1	24	4.33 (−2.93, 11.59)	0.24	N/A	N/A	N/A	⊕⊕⊕○ Moderate #
iTBS+LFrTMS	2	47	4.84 (−0.22, 9.89)	0.06	0	0.86	0.35	⊕⊕⊕○ Moderate #
**3.** **FMA-UE 1 month (compared with control)**								
VNS	1	17	2.23 (−6.41, 10.87)	0.61	N/A	N/A	N/A	⊕⊕⊕○ Moderate #
taVNS	1	21	**4.34 (2.95, 5.73)**	<0.00001	N/A	N/A	N/A	⊕⊕⊕○ Moderate *
SES	1	19	5.92 (−0.17, 12.01)	0.06	N/A	N/A	N/A	⊕⊕⊕○ Moderate #
MCS	3	200	2.03 (−0.47, 4.54)	0.11	58	4.71	0.09	⊕○○○ Very low **, $
dtDCS	1	19	−0.40 (−5.02, 4.22)	0.87	N/A	N/A	N/A	⊕⊕○○ Low *, #
atDCS	3	88	**6.37 (1.00, 11.73)**	0.02	0	0.98	0.61	⊕⊕⊕○ Moderate #
ctDCS	2	28	1.61 (−6.64, 9.86)	0.70	0	0.09	0.76	⊕⊕⊕○ Moderate #
drTMS	1	29	−0.82(−25.33, 23.69)	0.95	N/A	N/A	N/A	⊕⊕○○ Low ##
LFrTMS	7	493	3.28 (0.01, 6.55)	0.05	85	41.15	<0.00001	⊕⊕○○ Low #, $
HFrTMS	2	88	**4.33 (1.49, 7.16)**	0.003	7	1.08	0.30	⊕⊕⊕⊕ High
cTBS	1	13	**2.97 (1.26, 4.68)**	0.0007	N/A	N/A	N/A	⊕⊕⊕⊕ High
LFrTMS+atDCS	1	30	1.20 (−0.33,2.73)	0.12	N/A	N/A	N/A	⊕⊕⊕○ Moderate *
LFrTMS+ctDCS	1	30	0.87 (−0.27, 2.01)	0.13	N/A	N/A	N/A	⊕⊕⊕○ Moderate *
**4.** **FMA-UE 3 month (compared with control)**								
VNS	2	125	**3.14 (1.08, 5.21)**	0.003	0	0.32	0.57	⊕⊕⊕⊕ High
taVNS	1	21	**3.22 (0.48, 5.96)**	0.02	N/A	N/A	N/A	⊕⊕○○ Low *, #
ENMES	2	42	−2.61 (−8.19, 2.98)	0.36	0	0.35	0.55	⊕⊕○○ Low *, #
SES	2	45	0.65 (−2.15, 3.44)	0.65	0	0.04	0.83	⊕⊕⊕○ Moderate *
MCS	3	184	2.01 (−1.42, 5.45)	0.25	47	3.81	0.15	⊕○○○ Very low **, &
atDCS	4	161	3.54 (−1.43, 8.51)	0.16	0	1.92	0.59	⊕⊕⊕⊕ High
ctDCS	2	78	1.12 (−5.66, 7.91)	0.75	0	0.04	0.84	⊕⊕⊕○ Moderate #
drTMS	2	70	**7.48 (3.35, 11.61)**	0.0004	4	1.04	0.31	⊕⊕⊕⊕ High
LFrTMS	4	299	3.76 (−0.57, 8.09)	0.09	68	9.52	0.02	⊕⊕⊕○ Moderate &
HFrTMS	3	116	**5.39 (2.44, 8.34)**	0.0003	0	1.04	0.59	⊕⊕⊕○ Moderate *
iTBS	1	14	4.99 (−3.33, 13.31)	0.24	N/A	N/A	N/A	⊕⊕○○ Low *, #

MD: mean difference; CI: confidence interval; N/A: Not Applicable; * Limitations (risk of bias); ** Severe limitations (risk of bias); # Imprecision; ## Severe imprecision; $ Inconsistency; & Indirectness. “High”, “Moderate”, “Low and “Very low” explain these symbols, to be specific, ⊕⊕⊕⊕ for high, ⊕⊕⊕○ for moderate, ⊕⊕○○ for low and ⊕○○○ for very low.

## Data Availability

The data that support the findings of this study are available from the corresponding author upon reasonable request.

## Supplementary Materials

The following supporting information can be downloaded at: https://www.mdpi.com/article/10.3390/jcm11206162/s1. Part A. Search strategies; Part B. Characteristics of the included studies; Part C. Inclusion criteria, exclusion criteria, details of neurostimulation, efficacy outcomes, safety outcomes and conclusions of included studies; Part D. Catalogue of included studies [27,37,38,39,40,41,42,43,44,45,46,47,48,49,50,51,52,53,54,55,56,57,58,59,60,61,62,63,64,65,66,67,68,69,70,71,72,73,74,75,76,77,78,79,80,81,82,83,84,85,86,87,88,89,90,91,92,93,94,95,96,97,98,99,100,101,102,103,104,105,106,107,108,109,110,111,112,113,114,115,116,117,118,119,120,121,122,123]; Part E. Pair-wise forest plot; Part F. Pair-wise sensitivity analysis; Part G. The details of GRADE for pair-wise meta-analysis and network metanalysis; Part H. Results for ARAT; Part I. Results for BBT; Part J. Network meta-regression; Part K. Network global consistent analysis and sensitivity analysis; Part L. Network local consistent analysis and heterogeneity analysis of FMA-UE LFU; Part M. Network local consistent analysis and heterogeneity analysis of FMA-UE EOT; Part N. Network local consistent analysis and heterogeneity analysis of FMA-UE 1 month; Part O. Network local consistent analysis and heterogeneity analysis of FMA-UE 3 months; Part P. Risk of bias for included studies; Part Q. Network funnel plot.

## Abbreviations

AE = adverse event; ARAT = Action Research Arm Test; atDCS = anodal tDCS, BBT = Box and Block Test; CI = confidence interval; cNMES = cyclic NMES; CrI = credible interval; cTBS = continuous TBS; ctDCS = cathodal tDCS; DIC = deviance information criterion; drTMS = dual rTMS; dtDCS = dual tDCS; ENMES = EEG-trigger NMES; EOT =end of treatment; FES = functional electrical stimulation; FMA-UE = Fugl-Meyer Assessment-Upper Extremity; FMS = functional magnetic stimulation; GRADE = Grading of Recommendations Assessment, Development and Evaluation; HFrTMS = high frequency rTMS; iTBS = intermittent TBS; LFrTMS = low frequency rTMS; LFU = longest follow-up; MCS = motor cortex stimulation; MD = Mean difference; MID = minimal importance difference; NMA = Network Meta-analysis; NMES = neuromuscular electrical stimulation; nVNS = no-invasive VNS; PRISMA = Preferred Reporting Items for Systematic Reviews and Meta-Analyses; RCT = randomized controlled trial; rPMS = repetitive peripheral magnetic stimulation; rTMS = repetitive transcranial magnetic stimulation; SES = somatosensory electrical stimulation; SUCRA = surface under the curve ranking area; TBS = theta burst stimulation; tDCS = transcranial direct current stimulation; TENS = transcutaneous electrical nerve stimulation; VNS = vagus nerve stimulation.
