# Supplementary material for: Efficacy of Neurostimulations for Upper Extremity Function Recovery after Stroke: A Systematic Review and Network Meta-Analysis
Source: J Clin Med. 2022 Oct 19;11(20):6162. doi: 10.3390/jcm11206162 (PMC9605313; doi:10.3390/jcm11206162)
Supplement: Supplementary file 1 [file jcm-11-06162-s001.zip › jcm-1942754-supplementary.pdf]

Supplementary File

Efficacy of Neurostimulations for Upper Extremity Function Recovery after Stroke: Systematic Review and Network Meta-Analysis

Part	Legends for Supplementary Materials	Page
A.	Search strategies	2-3
B.	Characteristics of the included studies	4-43
C.	Inclusion criteria, exclusion criteria, details of neurostimulation, efficacy outcomes, safety outcomes and conclusions of included studies	44-212
D.	Catalogue of included studies	213-218
E.	Pair-wise forest plot	219-228
F.	Pair-wise sensitivity analysis	229-231
G.	The details of GRADE for pair-wise meta-analysis and network metanalysis	232-235
H.	Results for ARAT	236-238
I.	Results for BBT	239-242
J.	Network meta-regression	243-244
K.	Network global consistent analysis and sensitivity analysis	245
L.	Network local consistent analysis and heterogeneity analysis of FMA-UE LFU	246-255
M.	Network local consistent analysis and heterogeneity analysis of FMA-UE EOT	256-265
N.	Network local consistent analysis and heterogeneity analysis of FMA-UE 1 month	266-270
O.	Network local consistent analysis and heterogeneity analysis of FMA-UE 3 months	271-275
P.	Risk of bias for included studies	276-279
Q.	Network funnel plot	280-283

A. Search strategies

MEDLINE-1856

(((((((((((((((((((((((((((Strokes) OR (Cerebrovascular Accident)) OR (Cerebrovascular Accidents)) OR (CVA (Cerebrovascular Accident))) OR (CVAs (Cerebrovascular Accident))) OR (Cerebrovascular Apoplexy)) OR (Apoplexy, Cerebrovascular)) OR (Vascular Accident, Brain)) OR (Brain Vascular Accident)) OR (Brain Vascular Accidents)) OR (Vascular Accidents, Brain)) OR (Cerebrovascular Stroke)) OR (Cerebrovascular Strokes)) OR (Stroke, Cerebrovascular)) OR (Strokes, Cerebrovascular)) OR (Apoplexy)) OR (Cerebral Stroke)) OR (Cerebral Strokes)) OR (Stroke, Cerebral)) OR (Strokes, Cerebral)) OR (Stroke, Acute)) OR (Acute Stroke)) OR (Acute Strokes)) OR (Strokes, Acute)) OR (Cerebrovascular Accident, Acute)) OR (Acute Cerebrovascular Accident)) OR (Acute Cerebrovascular Accidents)) OR (Cerebrovascular Accidents, Acute) AND (((((((Extremities, Upper) OR (Upper Extremities)) OR (Membrum superius)) OR (Upper Limb)) OR (Limb, Upper)) OR (Limbs, Upper)) OR (Upper Limbs)) OR (Extremity, Upper) AND ((somatosensory electrical stimulation) OR (transcutaneous electrical nerve stimulation) OR (peripheral electrical stimulation) OR (functional electrical stimulation) OR (motor cortex stimulation) OR (neuromuscular electrical stimulation) OR (vagus nerve stimulation) OR (transcranial direct current stimulation) OR (transcranial magnetic stimulation) OR (theta burst stimulation) OR (peripheral magnetic stimulation) OR (function magnetic stimulation) OR (brain stimulation) OR neurostimulation) AND (random* AND control* AND trial)

Embase-605

((strokes OR (cerebrovascular AND accident) OR (cerebrovascular AND accidents) OR (cva AND cerebrovascular AND accident) OR (cvas AND cerebrovascular AND accident) OR (cerebrovascular AND apoplexy) OR (apoplexy, AND cerebrovascular) OR (vascular AND accident, AND brain) OR (brain AND vascular AND accident) OR (brain AND vascular AND accidents) OR (vascular AND accidents, AND brain) OR (cerebrovascular AND stroke) OR (cerebrovascular AND strokes) OR (stroke, AND cerebrovascular) OR (strokes, AND cerebrovascular) OR apoplexy OR (cerebral AND stroke) OR (cerebral AND strokes) OR (stroke, AND cerebral) OR (strokes, AND cerebral) OR (stroke, AND acute) OR (acute AND stroke) OR (acute AND strokes) OR (strokes, AND acute) OR (cerebrovascular AND accident, AND acute) OR (acute AND cerebrovascular AND accident) OR (acute AND cerebrovascular AND accidents) OR (cerebrovascular AND accidents, AND acute)) AND (extremities, AND upper OR (upper AND extremities) OR (membrum AND superius) OR (upper AND limb) OR (limb, AND upper) OR (limbs, AND upper) OR (upper AND limbs)) OR (extremity, AND upper)) AND (somatosensory AND electrical AND stimulation OR (transcutaneous AND electrical AND nerve AND stimulation) OR (peripheral AND electrical AND stimulation) OR (functional AND electrical AND stimulation) OR (motor AND cortex AND stimulation) OR (neuromuscular AND electrical AND stimulation) OR (vagus AND nerve AND stimulation) OR (transcranial AND direct AND current AND stimulation) OR (transcranial AND magnetic AND stimulation) OR (theta AND burst AND stimulation) OR (peripheral AND magnetic AND stimulation) OR (function AND magnetic AND stimulation) OR (brain AND stimulation) OR neurostimulation) AND random* AND control* AND trial

Cochrane library-511

(((((((((((((((((((((((((((Strokes) OR (Cerebrovascular Accident)) OR (Cerebrovascular Accidents)) OR (CVA (Cerebrovascular Accident))) OR (CVAs (Cerebrovascular Accident))) OR (Cerebrovascular Apoplexy)) OR (Apoplexy, Cerebrovascular)) OR (Vascular Accident, Brain)) OR (Brain Vascular Accident)) OR (Brain Vascular Accidents)) OR (Vascular Accidents, Brain)) OR (Cerebrovascular Stroke)) OR (Cerebrovascular Strokes)) OR (Stroke, Cerebrovascular)) OR (Strokes, Cerebrovascular)) OR (Apoplexy)) OR (Cerebral Stroke)) OR (Cerebral Strokes)) OR (Stroke, Cerebral)) OR (Strokes, Cerebral)) OR (Stroke, Acute)) OR (Acute Stroke)) OR (Acute Strokes)) OR (Strokes, Acute)) OR (Cerebrovascular Accident, Acute)) OR (Acute Cerebrovascular Accident)) OR (Acute Cerebrovascular Accidents)) OR (Cerebrovascular Accidents, Acute) in Title Abstract Keyword AND (((((((Extremities, Upper) OR (Upper Extremities)) OR (Membrum superius)) OR (Upper Limb)) OR (Limb, Upper)) OR (Limbs, Upper)) OR (Upper Limbs)) OR (Extremity, Upper) in Title Abstract Keyword AND (somatosensory electrical stimulation) OR (transcutaneous electrical nerve stimulation) OR (peripheral electrical stimulation) OR (functional electrical stimulation) OR (motor cortex stimulation) OR (neuromuscular electrical stimulation) OR (vagus nerve stimulation) OR (transcranial direct current stimulation) OR (transcranial magnetic stimulation) OR (theta burst stimulation) OR (peripheral magnetic stimulation) OR (function magnetic stimulation) OR (brain stimulation) OR neurostimulation in Title Abstract Keyword AND random* AND control* AND trial in Title Abstract Keyword - (Word variations have been searched)

Clinicaltrial.gov-179

((brain stimulation) OR (neurostimulation)) AND (upper) | Stroke

B. Characteristics of the Included Studies

Study And Publications	Countries And Centers	Included Sample Sizes	Included Treatment Groups (No. of participants)	Female（%）	Age, y, mean (SD)	Time of outcomes assessment (Months)
2016 Dawson et al. (NCT01669161) *Stroke*	UK 2	20	VNS + Rehabilitation (9) vs. Rehabilitation (11)	20.0%	59.44 ± 13.67	EOT
2017 Capone et al. (NCT03592745) *Neural Plasticity*	Italy 1	12	taVNS + Rehabilitation (7) vs. Rehabilitation (5)	41.7%	54.50 ± 6.18	EOT
2018 Kimberley et al. (NCT02243020) *Stroke*	USA UK 4	17	VNS + Rehabilitation (8) vs. Rehabilitation (9)	47.1%	59.76 ± 10.73	EOT, 1, 3
2020 Wu et al. (ChiCTR1800019635) *Neural Plasticity*	China 1	21	taVNS + Rehabilitation (10) vs. Rehabilitation (11)	38.1%	63.10 ± 10.15	EOT, 1, 3

2021 Dawson et al.<br>(NCT03131960)<br>*Lancet*	USA<br>UK<br>19	108	VNS + Rehabilitation (53) vs. Rehabilitation (55)	35.2%	60.10 ± 9.72	EOT, 3
2016 Amasyali et al.<br>(-)<br>*International Journal of Rehabilitation Research*	Turkey<br>1	15	EMG-triggered NMES + Rehabilitation (7) vs. Rehabilitation (8)	40.0%	58.80 ± 12.69	EOT, 3
2016 Wilson et al.<br>(NCT00142792)<br>*Neurorehabilitation and Neural Repair*	USA<br>3	122	EMG-triggered NMES + Rehabilitation (41) vs. Cyclic NMES + Rehabilitation (39) vs. SES + Rehabilitation (42)	41.8%	56.49 ± 13.88	EOT, 1, 3, 6
2016 Jeon et al.<br>(-)<br>*NeuroRehabilitation*	Korea<br>1	20	EMG-triggered Rehabilitation (10) vs. Cyclic NMES + Rehabilitation (10)	30.0%	53.8 ± 11.43	EOT
2013 Boyaci et al.<br>(-)<br>*International Journal of Rehabilitation Research*	Turkey<br>1	31	EMG-triggered NMES + Rehabilitation (11) vs. Cyclic NMES + Rehabilitation (10) vs. SES Rehabilitation (10)	61.9%	59.26 ± 11.69	EOT

2007 Hemmen et al.<br>(-)<br>*Clinical Rehabilitation*	Netherlands<br>2	27	EMG-triggered NMES + Rehabilitation (14) vs. Cyclic NMES + Rehabilitation (13)	40.7%	61.43 ± 12.29	EOT/3, 12
2008 de Kroon et al.<br>(-)<br>*Clinical Rehabilitation*	Netherlands<br>1	21	EMG-triggered NMES + Rehabilitation (11) vs. Cyclic NMES + Rehabilitation (10)	23.8%	58.92 ± 9.39	EOT/3
2017 Chuang et al.<br>(NCT01913509)<br>*Journal of NeuroEngineering and Rehabilitation*	China<br>2	38	EMG-triggered NMES + Rehabilitation (19) vs. SES + Rehabilitation (19)	34.2%	60.76 ± 10.84	EOT, 1
2015 McCabe et al.<br>(NCT01725659)<br>*Archives of Physical Medicine and Rehabilitation*	USA<br>1	23	Cyclic NMES + Rehabilitation (12) vs. Rehabilitation (11)	43.5%	NA	EOT
2014 Shimodozono et al.<br>(-)<br>*Brain Injury*	Japan<br>1	18	Cyclic NMES + Rehabilitation (9) vs. Rehabilitation (9)	16.7%	61.50 ± 12.14	EOT

2011 Shindo et al. (UMIN000001971) *Neurorehabilitation and Neural Repair*	Japan 1	20	Cyclic NMES + Rehabilitation (10) vs. Rehabilitation (10)	25.0%	58.05 ± 14.44	EOT
2015 Shen et al. (-) *CNS & Neurological Disorders - Drug Targets*	China 1	60	FES + Rehabilitation (30) vs. Cyclic NMES + Rehabilitation (30)	26.7%	60.30 ± 14.27	EOT
2017 Zhou et al. (-) *Journal of Rehabilitation Medicine*	China 1	36	FES + Rehabilitation (18) vs. Cyclic NMES + Rehabilitation (18)	30.6%	53.90 ± 12.26	EOT
2019 Zheng et al. (-) *Journal of Rehabilitation Medicine*	China 1	41	FES + Rehabilitation (21) vs. Cyclic NMES + Rehabilitation (20)	22.0%	62.39 ± 12.03	EOT
2012 Knutson et al. (-) *Neurorehabilitation and Neural Repair*	USA 1	17	FES + Rehabilitation (9) vs. Cyclic NMES + Rehabilitation (8)	47.1%	53.22 ± 10.96	EOT, 1 3

2016 Knutson et al.<br>(NCT00891319)<br>*Stroke*	USA<br>1	80	FES + Rehabilitation (40) vs.<br>Cyclic NMES + Rehabilitation (40)	36.3%	NA	EOT, 6
2020 Knutson et al.<br>(NCT01688856)<br>*American Journal of Physical Medicine & Rehabilitation*	USA<br>1	67	FES + Rehabilitation (56) vs.<br>Cyclic NMES + Rehabilitation (11)	35.8%	58 ± 12.5	EOT, 6
2006 Brown<br>(-)<br>*Neurosurgery*	USA<br>4	8	MCS + Rehabilitation (4) vs.<br>Rehabilitation (4)	37.5%	58.00 ± 16.21	EOT, 3
2008 Levy<br>(-)<br>*Journal of Neurosurgery*	USA<br>7	24	MCS + Rehabilitation (12) vs.<br>Rehabilitation (12)	37.5%	56.85 ± 13.48	EOT/1
2016 Levy<br>(-)<br>*Neurorehabilitation and Neural Repair*	USA<br>21	152	MCS + Rehabilitation (94) vs.<br>Rehabilitation (58)	40.8%	56.78 ± 11.05	EOT, 1, 3, 6

2008 Huang<br>(-)<br>*Neurosurgery*	USA<br>NR	24	MCS + Rehabilitation (12) vs. Rehabilitation (12)	37.5%	56.85 ± 13.48	EOT
2018 Ghaziani et al.<br>(NCT02250365)<br>*Neurorehabilitation and Neural Repair*	Denmark<br>1	102	SES + Rehabilitation (53) vs. Rehabilitation (49)	48.0%	71.48 ± 11.48	EOT, 6
2015 Fleming et al.<br>(ISRCTN 05542931)<br>*Neurorehabilitation and Neural Repair*	UK<br>5	33	SES + Rehabilitation (16) vs. Rehabilitation (17)	39.4%	61.42 ± 13.36	EOT, 3, 6
2016 Carrico et al.<br>(NCT02587234)<br>*Archives of Physical Medicine and Rehabilitation*	USA<br>1	19	SES + Rehabilitation (10) vs. Rehabilitation (9)	52.6%	55.68 ± 9.18	EOT, 1
2017 Takebayashi et al.<br>(UMIN000020927)<br>*frontiers in Neurology*	Japan<br>3	20	SES + Dual-tDCS + Rehabilitation (10) vs. Rehabilitation (10)	30.0%	59.30 ± 12.30	EOT

2018 Pan et al.<br>(NCT03277534)<br>*Scientific Reports*	China<br>1	12	SES + Rehabilitation (6) vs.<br>Rehabilitation (6)	8.3%	56.65 ± 13.78	EOT, 2, 3
2021 Alwhaibi<br>(-)<br>*Healthcare*	Egypt<br>1	40	SES + Rehabilitation (20) vs.<br>Rehabilitation (20)	52.5%	52.15 ± 5.26	EOT
2017 Jung<br>(-)<br>*NeuroRehabilitation*	Korea<br>1	46	SES + Rehabilitation (23) vs.<br>Rehabilitation (23)	52.2%	56.05 ± 8.61	EOT
2020 Yurdakul<br>(NCT04113369)<br>*Neurological Sciences*	Turkey<br>1	30	SES + Rehabilitation (15) vs.<br>Cyclic NMES + Rehabilitation (15)	56.7%	67.40 ± 12.99	EOT
2013 de Jong et al.<br>(NTR1748)<br>*Journal of Physiotherapy*	Netherlan<br>ds<br>3	46	Cyclic NMES + Rehabilitation<br>(23) vs.<br>Rehabilitation (23)	41.3%	57.50 ± 12.02	EOT

2019 Alisar (NCT03839316) *Journal of Stroke and Cerebrovascular Diseases*	Turkey 1	32	Dual-tDCS + Rehabilitation (16) vs. Rehabilitation (16)	59.4%	63.53 ± 11.27	EOT
2021 Chen (NCT04369235) *Journal of NeuroEngineering and Rehabilitation*	China 1	24	iTBS + Anodal-tDCS + Rehabilitation (12) vs. Rehabilitation (12)	29.2%	64.00 ± 14.61	EOT
2019 Bornheim (-) *Brain Stimulation*	Belgium 1	50	Anodal-tDCS + Rehabilitation (25) vs. Rehabilitation (25)	34.0%	62.98 ± 12.29	EOT, 1, 3, 6, 12
2019 Beaulieu (-) *Restorative Neurology and Neuroscience*	Canada 1	14	Dual-tDCS + Rehabilitation (7) vs. Rehabilitation (7)	28.6%	68.85 ± 10.02	EOT
2015 Ang (NCT01897025) *Archives of Physical Medicine and Rehabilitation*	Singapore 1	19	Dual-tDCS + Rehabilitation (10) vs. Rehabilitation (9)	26.3%	54.1 ± 10.6	EOT, 1

2016 Allman (NCT01414582) *Science Translational Medicine*	UK 1	24	Anodal-tDCS + Rehabilitation (11) vs. Rehabilitation (13)	29.2%	62.85 ± 11.71	EOT, 1, 3
2019 Jin (ChiCTR180002008) *Journal of the Neurological Sciences*	China 1	30	Dual-tDCS + Rehabilitation (20) vs. Rehabilitation (10)	20.0%	58.40 ± 8.08	EOT
2011 Hesse (-) *Neurorehabilitation and Neural Repair*	Germany and Italy 3	85	Anodal-tDCS + Rehabilitation (32) vs. Cathodal-tDCS + Rehabilitation (32) vs. Rehabilitation (32)	38.5%	64.97 ± 9.76	EOT, 3
2010 Lindenberg (-) *Neurology*	USA 1	20	Dual-tDCS + Rehabilitation (10) vs. Rehabilitation (10)	25.0%	58.75 ± 13.80	EOT
2020 Liao (NCT02827864) *Journal of NeuroEngineering and Rehabilitation*	China 1	28	Anodal-tDCS + Rehabilitation (20) vs. Rehabilitation (8)	25.0%	55.63 ± 8.61	EOT

2014 Lee<br>(-)<br>*Archives of Physical Medicine and Rehabilitation*	Korea<br>1	40	Cathodal-tDCS + Rehabilitation (20) vs. Rehabilitation (20)	51.2%	61.67 ± 12.50	EOT
2020 Gong<br>(ChiCTR2000034846)<br>*Clinical Rehabilitation*	China<br>1	60	LF-rTMS + Rehabilitation (15) vs. LF-rTMS + Cathodal-tDCS + Rehabilitation (15) vs. LF-rTMS + Anodal-tDCS + Rehabilitation (15) vs. Rehabilitation (15)	26.7%	62.11 ± 13.16	EOT, 1
2014 Fusco<br>(-)<br>*BioMed Research International*	Italy<br>1	11	Cathodal-tDCS + Rehabilitation (5) vs. Rehabilitation (6)	54.5%	58.36 ± 14.35	EOT, 1, 2, 3
2019 Edwards<br>(NCT03562663)<br>*Restorative Neurology and Neuroscience*	USA<br>2	69	Anodal-tDCS + Rehabilitation (34) vs. Rehabilitation (35)	39.0%	67.56 ± 9.89	EOT, 6
2021 Kim<br>(-)<br>*Journal of Stroke and Cerebrovascular Diseases*	Korea<br>1	30	Dual-tDCS + Rehabilitation (15) vs. Rehabilitation (15)	50.0%	60.27 ± 5.74	EOT

2019 Mazzoleni (NCT02496026) *IEEE Transactions on Neural Systems and Rehabilitation Engineering*	Italy 1	39	Anodal-tDCS + Rehabilitation (20) vs. Rehabilitation (19)	61.5%	68.10 ± 15.87	EOT
2020 Yao (ChiCTR1800019386) *Journal of NeuroEngineering and Rehabilitation*	China 1	40	Cathodal-tDCS + Rehabilitation (20) vs. Rehabilitation (20)	22.5%	64.60 ± 6.98	EOT
2014 Viana (-) *NeuroRehabilitation*	Brazil 1	20	Anodal-tDCS + Rehabilitation (10) vs. Rehabilitation (10)	20.0%	55.50 ± 10.96	EOT
2015 Triccas (NCT01405378) *NeuroRehabilitation*	UK 1	23	Anodal-tDCS + Rehabilitation (12) vs. Rehabilitation (11)	39.1%	63.40 ± 12.00	EOT, 3
2018 Shaheiwola (ChiCTR-ICR-15006108) *frontiers in Human Neuroscience*	China 1	30	Dual-tDCS + Rehabilitation (15) vs. Rehabilitation (15)	10.0%	50.60 ± 10.14	EOT

2019 Salazar (NCT02818608) *Annals of Physical and Rehabilitation Medicine*	Brazil 1	30	Dual-tDCS + Rehabilitation (15) vs. Rehabilitation (15)	33.3%	58.00 ± 13.44	EOT
2015 Rocha (NCT01879787) *Disability and Rehabilitation*	Brazil 1	21	Anodal-tDCS + Rehabilitation (7) vs. Cathodal-tDCS + Rehabilitation (7) vs. Rehabilitation (7)	28.6%	NR	EOT, 1
2017 Pavlova (-) *Restorative Neurology and Neuroscience*	Sweden 1	11	Anodal-tDCS + Rehabilitation (5) vs. Rehabilitation (6)	54.5%	60.91 ± 11.95	EOT, 2
2021 Gottlieb (-) *NeuroRehabilitation*	Germany 1	28	LF-rTMS + Rehabilitation (14) vs. Rehabilitation (14)	57.1%	63.18 ± 11.00	EOT
2020 Chiu (-) *Journal of Stroke and Cerebrovascular Diseases*	USA 1	29	Dual-rTMS + Rehabilitation (14) vs. Rehabilitation (15)	NA	NA	EOT, 1, 3

2021 Chen<br>(NCT03350087)<br>*Journal of NeuroEngineering and Rehabilitation*	China<br>1	23	iTBS + Rehabilitation (12) vs. Rehabilitation (11)	21.7%	51.77 ± 10.27	EOT
2010 Chang<br>(-)<br>*Journal of Rehabilitation Medicine*	Korea<br>1	28	HF-rTMS + Rehabilitation (18) vs. Rehabilitation (10)	39.3%	56.61 ± 12.21	EOT, 3
2020 Chen<br>(ChiCTR1800019757)<br>*Journal of International Medical Research*	China<br>1	35	LF-rTMS + Rehabilitation (16) vs. rPMS + Rehabilitation (19)	22.9%	50.79 ± 12.79	EOT
2020 Obayashia<br>(-)<br>*NeuroRehabilitation*	Japan<br>1	19	rPMS + Rehabilitation (10) vs. Rehabilitation (9)	31.6%	68.09 ± 12.39	EOT
2014 Krewer<br>(-)<br>*Archives of Rehabilitation Research and Clinical Translation*	Cermany<br>1	63	rPMS + Rehabilitation (31) vs. Rehabilitation (32)	39.7%	54.49 ± 12.90	EOT

2019 Chen<br>(NCT01947413)<br>*BMC Neurology*	China<br>1	22	iTBS + Rehabilitation (11) vs. Rehabilitation (11)	36.4%	52.75 ± 9.57	EOT
2016 Du<br>(-)<br>*European Journal of Neurology*	China<br>1	69	HF-rTMS + Rehabilitation (23) vs. LF-rTMS + Rehabilitation (23) vs. Rehabilitation (23)	34.8%	55.72 ± 11.60	EOT, 1, 3
2020 Miu<br>(-)<br>*Annals of Rehabilitation Medicine*	China<br>1	51	LF-rTMS + Rehabilitation (25) vs. Cathodal-tDCS + Rehabilitation (26)	60.0%	64.88 ± 11.51	EOT, 1
2017 Guan<br>(NCT03163758)<br>*CNS Neuroscience & Therapeutics*	China<br>1	42	HF-rTMS + Rehabilitation (21) vs. Rehabilitation (21)	28.6%	58.55 ± 10.93	EOT, 1, 3, 6, 12
2014 Galvao<br>(NCT01875536)<br>*Archives of Physical Medicine and Rehabilitation*	Brazil<br>1	20	LF-rTMS + Rehabilitation (10) vs. Rehabilitation (10)	35.0%	61.00 ± 10.19	EOT, 1

2013 Hsu (NCT01323881) *Brain Stimulation*	China 1	12	iTBS + Rehabilitation (6) vs. Rehabilitation (6)	33.3%	59.55 ± 7.88	EOT
2018 Harvey (NCT02089464) *Stroke*	USA 12	199	LF-rTMS + Rehabilitation (132) vs. Rehabilitation (67)	34.7%	58.70 ± 13.10	EOT, 1, 3, 6
2021 Kuzu (NCT04562415) *Journal of Stroke and Cerebrovascular Diseases*	Turkey 1	20	LF-rTMS + Rehabilitation (7) vs. cTBS + Rehabilitation (6) Rehabilitation (6)	40.0%	60.70 ± 9.53	EOT, 1
2020 Kim (KCT0001768) *Brain Science*	Korea 1	20	LF-rTMS + Rehabilitation (8) vs. Rehabilitation (12)	40.0%	64.10 ± 14.84	EOT
2020 Kim (NCT02082015) *Neurorehabilitation and Neural Repair*	Korea 3	73	LF-rTMS + Rehabilitation (36) vs. Rehabilitation (37)	31.5%	62.06 ± 12.15	EOT, 1

2015 Matsuura<br>(UMIN000016021)<br>*European Journal of Neurology*	Japan<br>1	20	LF-rTMS + Rehabilitation (10) vs. Rehabilitation (10)	45.0%	73.45 ± 9.75	EOT
2018 Long<br>(-)<br>*Journal of Stroke and Cerebrovascular Diseases*	China<br>1	62	LF-rTMS + Rehabilitation (21) vs. Dual-rTMS + Rehabilitation (21) vs. Rehabilitation (20)	24.2%	56.58 ± 9.00	EOT, 3
2014 Rose<br>(-)<br>*Stroke Research and Treatment*	USA<br>1	19	LF-rTMS + Rehabilitation (9) vs. Rehabilitation (10)	31.6%	64.65 ± 7.89	EOT
2019 Pinto<br>(NCT02208466)<br>*Neurorehabilitation and Neural Repair*	USA<br>1	27	LF-rTMS + Rehabilitation (9) vs. Rehabilitation (18)	40.7%	54.78 ± 12.65	EOT
2020 Meng<br>(-)<br>*Restorative Neurology and Neuroscience*	China<br>1	28	iTBS + LF-rTMS + Rehabilitation (10) vs. LF-rTMS + Rehabilitation (10) vs. Rehabilitation (8)	35.7%	53.89 ± 10.52	EOT

2015 Zheng<br>(UMIN000018819)<br>*Journal of Huazhong University of Science and Technology*	China<br>1	112	LF-rTMS + Rehabilitation (58) vs. Rehabilitation (54)	39.3%	65.79 ± 13.25	EOT, 1
2018 Watanabe<br>(UMIN000018819)<br>*Journal of the Neurological Sciences*	Japan<br>1	21	iTBS + Rehabilitation (8) vs. LF-rTMS + Rehabilitation (7) vs. Rehabilitation (6)	33.3%	71.64 ± 6.67	EOT/3
2020 Wang<br>(ChiCTR-IPR-17013580)<br>*Brain Stimulation*	China<br>1	45	HF-rTMS + Rehabilitation (15) vs. LF-rTMS + Rehabilitation (15) vs. Rehabilitation (15)	33.3%	59.57 ± 12.29	EOT
2017 Tosun<br>(-)<br>*Topics in Stroke Rehabilitation*	Turkey<br>1	25	LF-rTMS + Rehabilitation (9) vs. Cyclic NMES + LF-rTMS + Rehabilitation (7) vs. Rehabilitation (9)	44.0%	56.90 ± 11.23	EOT
2013 Sung<br>(-)<br>*Stroke*	China<br>1	54	iTBS + LF-rTMS + Rehabilitation (15) vs. iTBS + Rehabilitation (12) vs. LF-rTMS + Rehabilitation (13) vs. Rehabilitation (14)	24.1%	63.14 ± 12.10	EOT

2020 Sharma<br>(-)<br>*Archives of Rehabilitation Research and Clinical Translation*	India<br>1	96	LF-rTMS + Rehabilitation (47) vs. Rehabilitation (49)	30.2%	53.85 ± 14.17	EOT
2012 Seniów<br>(-)<br>*Neurorehabilitation and Neural Repair*	Poland<br>1	40	LF-rTMS + Rehabilitation (20) vs. Rehabilitation (20)	35.0%	63.45 ± 8.93	EOT, 3
2013 Abo<br>(-)<br>*International Journal of Stroke*	Japan<br>2	66	LF-rTMS + Rehabilitation (44) vs. Rehabilitation (22)	42.4%	58.57 ± 12.02	EOT
2008 Chan<br>(-)<br>*Neurorehabilitation and Neural Repair*	China<br>1	20	FES + Rehabilitation (10) vs. SES + Rehabilitation (10)	45.0%	45.50 ± 16.08	EOT

Study And Publications	Types of Stroke		Time after Stroke, Years, mean (SD)	Side of Hemiplegia (right/left)	FMA-UE baseline score, mean (SD)
	Ischemic	Hemorrhagic			
2016 Dawson et al. (NCT01669161) *Stroke*	VNS + Rehabilitation (9) vs. Rehabilitation (11)	VNS + Rehabilitation (0) vs. Rehabilitation (0)	1.75 ± 1.15	VNS + Rehabilitation (3/6) vs. Rehabilitation (4/7)	42.96 ± 9.15
2017 Capone et al. (NCT03592745) *Neural Plasticity*	taVNS + Rehabilitation (5) vs. Rehabilitation (3)	taVNS + Rehabilitation (2) vs. Rehabilitation (2)	6.13 ± 3.32	NA	26.94 ± 12.15
2018 Kimberley et al. (NCT02243020) *Stroke*	VNS + Rehabilitation (8) vs. Rehabilitation (9)	VNS + Rehabilitation (0) vs. Rehabilitation (0)	0.35 ± 09.81	VNS + Rehabilitation (7/1) vs. Rehabilitation (4/5)	33.15 ± 8.64
2020 Wu et al. (ChiCTR1800019635) *Journal of Clinical Neuroscience*	taVNS + Rehabilitation (10) vs. Rehabilitation (11)	taVNS + Rehabilitation (0) vs. Rehabilitation (0)	0.09 ± 0.03	taVNS + Rehabilitation (6/4) vs. Rehabilitation (8/3)	17.14 ± 4.31

2021 Dawson et al. (NCT03131960) *Lancet*	VNS + Rehabilitation (53) vs. Rehabilitation (55)	VNS + Rehabilitation (0) vs. Rehabilitation (0)	3.20 ± 2.45	VNS + Rehabilitation (25/28) vs. Rehabilitation (26/29)	35.06 ± 7.99
2016 Amasyali et al. (-) *International Journal of Rehabilitation Research*	EMG-triggered NMES+ Rehabilitation (7) vs. Rehabilitation (8)	EMG-triggered NMES+ Rehabilitation (0) vs. Rehabilitation (0)	0.49 ± 0.17	EMG-triggered NMES+ Rehabilitation (5/2) vs. Rehabilitation (7/1)	40.40 ± 17.20
2016 Wilson et al. (NCT00142792) *Neurorehabilitation and Neural Repair*	EMG-triggered NMES + Rehabilitation (31) vs. Cyclic NMES + Rehabilitation (30) vs. SES + Rehabilitation (37)	EMG-triggered NMES + Rehabilitation (10) vs. Cyclic NMES + Rehabilitation (9) vs. SES + Rehabilitation (5)	0.23 ± 0.20	EMG-triggered NMES + Rehabilitation (21/20) vs. Cyclic NMES + Rehabilitation (21/18) vs. SES + Rehabilitation (20/22)	28.68 ± 12.78
2016 Jeon et al. (-) *NeuroRehabilitation*	EMG-triggered NMES + Rehabilitation (2) vs. Cyclic NMES + Rehabilitation (4)	EMG-triggered NMES + Rehabilitation (8) vs. Cyclic NMES + Rehabilitation (6)	0.36 ± 0.14	EMG-triggered NMES + Rehabilitation (1/9) vs. Cyclic NMES + Rehabilitation (2/8)	18.30 ± 11.78

2013 Boyaci et al. (-) *International Journal of Rehabilitation Research*	EMG-triggered NMES + Rehabilitation (10) vs. Cyclic NMES + Rehabilitation (6) vs. SES + Rehabilitation (7)	EMG-triggered NMES + Rehabilitation (1) vs. Cyclic NMES + Rehabilitation (4) vs. SES + Rehabilitation (3)	0.56 ± 0.40	EMG-triggered NMES + Rehabilitation (5/6) vs. Cyclic NMES + Rehabilitation (6/4) vs. SES + Rehabilitation (6/4)	32.48 ± 15.20
2007 Hemmen et al. (-) *Clinical Rehabilitation*	NR	NR	0.15 ± 0.11	EMG-triggered NMES + Rehabilitation (6/7) vs. Cyclic NMES + Rehabilitation (9/4)	42.20 ± 8.44
2008 de Kroon et al. (-) *Clinical Rehabilitation*	EMG-triggered NMES + Rehabilitation (10) vs. Cyclic NMES + Rehabilitation (8)	EMG-triggered NMES + Rehabilitation (1) vs. Cyclic NMES + Rehabilitation (2)	1.84 ± 1.75	EMG-triggered NMES + Rehabilitation (3/8) vs. Cyclic NMES + Rehabilitation (3/7)	33.48 ± 10.30
2017 Chung et al. (NCT01913509) *Journal of NeuroEngineering and Rehabilitation*	EMG-triggered NMES + Rehabilitation (9) vs. SES + Rehabilitation (9)	EMG-triggered NMES + Rehabilitation (10) vs. SES + Rehabilitation (10)	0.63 ± 1.02	EMG-triggered NMES + Rehabilitation (10/9) vs. SES + Rehabilitation (7/12)	43.53 ± 18.77

2015 McCabe et al. (NCT01725659) *Archives of Physical Medicine and Rehabilitation*	NR	NR	NA	NR	23.20 ± 9.30
2014 Shimodozono et al. (-) *Brain Injury*	Cyclic NMES + Rehabilitation (7) vs. Rehabilitation (5)	Cyclic NMES + Rehabilitation (2) vs. Rehabilitation (4)	0.12 ± 0.04	Cyclic NMES + Rehabilitation (9) vs. Rehabilitation (9)	9.80 ± 4.83
2011 Shindo et al. (UMIN000001971) *Neurorehabilitation and Neural Repair*	Cyclic NMES + Rehabilitation (6) vs. Rehabilitation (8)	Cyclic NMES + Rehabilitation (4) vs. Rehabilitation (2)	0.10 ± 0.04	Cyclic NMES + Rehabilitation (4/6) vs. Rehabilitation (5/5)	23.70 ± 11.75
2015 Shen et al. (-) *CNS & Neurological Disorders - Drug Targets*	FES + Rehabilitation (21) vs. Cyclic NMES + Rehabilitation (18)	FES + Rehabilitation (9) vs. Cyclic NMES + Rehabilitation (12)	0.13 ± 0.09	FES + Rehabilitation (14/16) vs. Cyclic NMES + Rehabilitation (16/14)	21.75 ± 7.23

2017 Zhou et al.<br>(-)<br>*Journal of Rehabilitation Medicine*	FES + Rehabilitation (9) vs. Cyclic NMES + Rehabilitation (11)	FES + Rehabilitation (9) vs. Cyclic NMES + Rehabilitation (7)	0.20 ± 0.14	FES + Rehabilitation (12/6) vs. Cyclic NMES + Rehabilitation (8/10)	18.00 ± 8.66
2019 Zheng et al.<br>(-)<br>*Journal of Rehabilitation Medicine*	FES + Rehabilitation (18) vs. Cyclic NMES + Rehabilitation (17)	FES + Rehabilitation (3) vs. Cyclic NMES + Rehabilitation (3)	8.17d ± 2.22d	NR	13.85 ± 4.50
2012 Knutson et al.<br>(-)<br>*Neurorehabilitation and Neural Repair*	FES + Rehabilitation (8) vs. Cyclic NMES + Rehabilitation (7)	FES + Rehabilitation (1) vs. Cyclic NMES + Rehabilitation (1)	0.28 ± 0.14	FES + Rehabilitation (6/3) vs. Cyclic NMES + Rehabilitation (4/4)	32.64 ± 11.37
2016 Knutson et al.<br>(NCT00891319)<br>*Stroke*	FES + Rehabilitation (35) vs. Cyclic NMES + Rehabilitation (32)	FES + Rehabilitation (5) vs. Cyclic NMES + Rehabilitation (8)	1.70 ± 3.87	FES + Rehabilitation (20/20) vs. Cyclic NMES + Rehabilitation (19/21)	33.00 ± 13.46

2020 Knutson et al. (NCT01688856) *American Journal of Physical Medicine & Rehabilitation*	FES + Rehabilitation (50) vs. Cyclic NMES + Rehabilitation (9)	FES + Rehabilitation (6) vs. Cyclic NMES + Rehabilitation (2)	0.37 ± 0.28	FES + Rehabilitation (21/35) vs. Cyclic NMES + Rehabilitation (5/6)	31 ± 9.8
2006 Brown (-) *Neurosurgery*	MCS + Rehabilitation (4) vs. Rehabilitation (4)	MCS + Rehabilitation (0) vs. Rehabilitation (0)	2.35 ± 1.87	MCS + Rehabilitation (4/0) vs. Rehabilitation (2/2)	38.50 ± 7.25
2008 Levy (-) *Journal of Neurosurgery*	MCS + Rehabilitation (12) vs. Rehabilitation (12)	MCS + Rehabilitation (0) vs. Rehabilitation (0)	2.73 ± 1.95	MCS + Rehabilitation (5/7) vs. Rehabilitation (5/6/1)	32.45 ± 8.21
2016 Levy (-) *Neurorehabilitation and Neural Repair*	MCS + Rehabilitation (94) vs. Rehabilitation (58)	MCS + Rehabilitation (0) vs. Rehabilitation (0)	4.81 ± 5.34	MCS + Rehabilitation (60/34) vs. Rehabilitation (26/32)	37.60 ± 6.00

2008 Huang<br>(-)<br>*Neurosurgery*	MCS + Rehabilitation (12) vs. Rehabilitation (12)	MCS + Rehabilitation (0) vs. Rehabilitation (0)	2.73 ± 1.95	MCS + Rehabilitation (5/7) vs. Rehabilitation (5/6/1) (R/L/Dural)	32.40 ± 8.19
2018 Ghaziani et al.<br>(NCT02250365)<br>*Neurorehabilitation and Neural Repair*	SES + Rehabilitation (43) vs. Rehabilitation (37)	SES + Rehabilitation (10) vs. Rehabilitation (12)	19.35d ± 10.27d	SES + Rehabilitation (25/28) vs. Rehabilitation (28/21)	33.41 ± 19.94
2015 Fleming et al.<br>(ISRCTN 05542931)<br>*Neurorehabilitation and Neural Repair*	SES + Rehabilitation (13) vs. Rehabilitation (14)	SES + Rehabilitation (3) vs. Rehabilitation (3)	2.31 ± 2.55	SES + Rehabilitation (10/6) vs. Rehabilitation (9/8)	40.31 ± 9.56
2016 Carrico et al.<br>(NCT02587234)<br>*Archives of Physical Medicine and Rehabilitation*	SES + Rehabilitation (9) vs. Rehabilitation (8)	SES + Rehabilitation (1) vs. Rehabilitation (1)	NR	SES + Rehabilitation (4/6) vs. Rehabilitation (7/2)	8.00 ± 4.32

2017 Takebayashi et al. (UMIN000020927) *frontiers in Neurology*	SES + Dual-tDCS + Rehabilitation (6) vs. Rehabilitation (8)	SES + Dual-tDCS + Rehabilitation (4) vs. Rehabilitation (2)	2.91 ± 3.22	SES + Dual-tDCS + Rehabilitation (5/5) vs. Rehabilitation (5/5)	43.50 ± 8.74
2018 Pan et al. (NCT03277534) *Scientific Reports*	SES + Rehabilitation (2) vs. Rehabilitation (4)	SES + Rehabilitation (4) vs. Rehabilitation (2)	3.18 ± 1.68	SES + Rehabilitation (4/2) vs. Rehabilitation (3/3)	47.80 ± 2.26
2021 Alwhaibi (-) *Healthcare*	SES + Rehabilitation (13) vs. Rehabilitation (12)	SES + Rehabilitation (7) vs. Rehabilitation (8)	1.81 ± 0.22	NR	24.80 ± 2.82
2017 Jung (-) *NeuroRehabilitation*	SES + Rehabilitation (12) vs. Rehabilitation (15)	SES + Rehabilitation (11) vs. Rehabilitation (8)	1.16 ± 0.51	SES + Rehabilitation (10/13) vs. Rehabilitation (12/11)	42.00 ± 8.76

2020 Yurdakul (NCT04113369) *Neurological Sciences*	SES + Rehabilitation (15) vs. Cyclic NMES + Rehabilitation (15)	SES + Rehabilitation (0) vs. Cyclic NMES + Rehabilitation (0)	0.13 ± 0.11	SES + Rehabilitation (7/8) vs. Cyclic NMES + Rehabilitation (9/6)	55.27 ± 11.36
2013 de Jong et al. (NTR1748) *Journal of Physiotherapy*	Cyclic NMES + Rehabilitation (19) vs. Rehabilitation (18)	Cyclic NMES + Rehabilitation (4) vs. Rehabilitation (5)	0.12 ± 0.04	Cyclic NMES + Rehabilitation (12/11) vs. Rehabilitation (8/15)	NR
2019 Alisar (NCT03839316) *Journal of Stroke and Cerebrovascular Diseases*	Dual-tDCS + Rehabilitation (11) vs. Rehabilitation (13)	Dual-tDCS + Rehabilitation (5) vs. Rehabilitation (3)	1.09 ± 1.51	Dual-tDCS + Rehabilitation (10/6) vs. Rehabilitation (6/10)	31.56 ± 22.08
2021 Chen (NCT03350087) *Journal of NeuroEngineering and Rehabilitation*	iTBS + Anodal-tDCS + Rehabilitation (6) vs. Rehabilitation (2)	iTBS + Anodal-tDCS + Rehabilitation (6) vs. Rehabilitation (9)	0.54 ± 0.92	iTBS + Anodal-tDCS + Rehabilitation (5/7) vs. Rehabilitation (4/7)	39.26 ± 11.08

2019 Bornheim<br>(-)<br>*Brain Stimulation*	Anodal-tDCS + Rehabilitation (25) vs. Rehabilitation (25)	Anodal-tDCS + Rehabilitation (0) vs. Rehabilitation (0)	NR	Anodal-tDCS + Rehabilitation (25/0) vs. Rehabilitation (25/0)	36.30 ± 3.60
2019 Beaulieu<br>(-)<br>*Restorative Neurology and Neuroscience*	Dual-tDCS + Rehabilitation (4) vs. Rehabilitation (6)	Dual-tDCS + Rehabilitation (3) vs. Rehabilitation (1)	1.36 ± 1.11	Dual-tDCS + Rehabilitation (2/5) vs. Rehabilitation (2/5)	59.5 ± 5.06
2015 Ang<br>(NCT01897025)<br>*Archives of Physical Medicine and Rehabilitation*	Dual-tDCS + Rehabilitation (6) vs. Rehabilitation (8)	Dual-tDCS + Rehabilitation (4) vs. Rehabilitation (1)	2.84 ± 1.64	Dual-tDCS + Rehabilitation (5/5) vs. Rehabilitation (6/3)	34.02 ± 7.84
2016 Allman<br>(NCT01414582)<br>*Science Translational Medicine*	Anodal-tDCS + Rehabilitation (9) vs. Rehabilitation (9)	Anodal-tDCS + Rehabilitation (2) vs. Rehabilitation (4)	1.03 ± 0.69	Anodal-tDCS + Rehabilitation (8/3) vs. Rehabilitation (9/4)	37.56 ± 16.40

2019 Jin (ChiCTR180002008) *Journal of the Neurological Sciences*	Dual-tDCS + Rehabilitation (12) vs. Rehabilitation (8)	Dual-tDCS + Rehabilitation (8) vs. Rehabilitation (2)	0.40 ± 0.17	Dual-tDCS + Rehabilitation (12/8) vs. Rehabilitation (7/3)	49.27 ± 17.22
2011 Hesse (-) *Neurorehabilitation and Neural Repair*	Anodal-tDCS + Rehabilitation (32) vs. Cathodal-tDCS + Rehabilitation (32) vs. Rehabilitation (32)	Anodal-tDCS + Rehabilitation (0) vs. Cathodal-tDCS + Rehabilitation (0) vs. Rehabilitation (0)	25.67 ± 10.99d	Anodal-tDCS + Rehabilitation (14/18) vs. Cathodal-tDCS + Rehabilitation (15/17) vs. Rehabilitation (16/16)	6.5 ± 3.59
2010 Lindenberg (-) *Neurology*	Dual-tDCS + Rehabilitation (10) vs. Rehabilitation (10)	Dual-tDCS + Rehabilitation (0) vs. Rehabilitation (0)	0.68 ± 0.43	Dual-tDCS + Rehabilitation (4/6) vs. Rehabilitation (3/7)	39.0 ± 12.13
2020 Liao (NCT02827864) *Journal of NeuroEngineering and Rehabilitation*	Anodal-tDCS + Rehabilitation (13) vs. Rehabilitation (7)	Anodal-tDCS + Rehabilitation (7) vs. Rehabilitation (1)	0.50 ± 0.43	Anodal-tDCS + Rehabilitation (13/7) vs. Rehabilitation (8/0)	35.07 ± 10.63

2014 Lee<br>(-)<br>*Archives of Physical Medicine and Rehabilitation*	Cathodal-tDCS + Rehabilitation (12) vs. Rehabilitation (14)	Cathodal-tDCS + Rehabilitation (8) vs. Rehabilitation (6)	17.35 ± 6.40d	Cathodal-tDCS + Rehabilitation (11/9) vs. Rehabilitation (13/7)	36.65 ± 22.49
2020 Gong<br>(ChiCTR2000034846)<br>*Clinical Rehabilitation*	LF-rTMS + Rehabilitation (11) vs. LF-rTMS + Cathodal-tDCS + Rehabilitation (10) vs. LF-rTMS + Anodal-tDCS + Rehabilitation (11) vs. Rehabilitation (10)	LF-rTMS + Rehabilitation (4) vs. LF-rTMS + Cathodal-tDCS + Rehabilitation (5) vs. LF-rTMS + Anodal-tDCS + Rehabilitation (4) vs. Rehabilitation (5)	16.49 ± 5.55d	NR	27.45 ± 4.89
2014 Fusco<br>(-)<br>*BioMed Research International*	Cathodal-tDCS + Rehabilitation (5) vs. Rehabilitation (6)	Cathodal-tDCS + Rehabilitation (0) vs. Rehabilitation (0)	NR	Cathodal-tDCS + Rehabilitation (3/2) vs. Rehabilitation (2/4)	24.73 ± 24.00
2019 Edwards<br>(NCT03562663)<br>*Restorative Neurology and Neuroscience*	Anodal-tDCS + Rehabilitation (34) vs. Rehabilitation (35)	Anodal-tDCS + Rehabilitation (0) vs. Rehabilitation (0)	3.61 ± 4.65	NR	24.73 ± 3.63

2021 Kim<br>(-)<br>*Journal of Stroke and Cerebrovascular Diseases*	Dual-tDCS + Rehabilitation (8) vs. Rehabilitation (7)	Dual-tDCS + Rehabilitation (7) vs. Rehabilitation (8)	0.22 ± 0.04	Dual-tDCS + Rehabilitation (8/7) vs. Rehabilitation (7/8)	43.60 ± 2.35
2019 Mazzoleni<br>(NCT02496026)<br>*IEEE Transactions on Neural Systems and Rehabilitation Engineering*	Anodal-tDCS + Rehabilitation (13) vs. Rehabilitation (16)	Anodal-tDCS + Rehabilitation (7) vs. Rehabilitation (3)	NR	Anodal-tDCS + Rehabilitation (11/9) vs. Rehabilitation (11/8)	40.35 ± 19.59
2020 Yao<br>(ChiCTR1800019386)<br>*Journal of NeuroEngineering and Rehabilitation*	Cathodal-tDCS + Rehabilitation (20) vs. Rehabilitation (20)	Cathodal-tDCS + Rehabilitation (0) vs. Rehabilitation (0)	0.16 ± 0.09	Cathodal-tDCS + Rehabilitation (8/12) vs. Rehabilitation (10/10)	25.15 ± 15.73
2014 Viana<br>(-)<br>*NeuroRehabilitation*	Anodal-tDCS + Rehabilitation (9) vs. Rehabilitation (10)	Anodal-tDCS + Rehabilitation (1) vs. Rehabilitation (0)	0.64 ± 0.36	Anodal-tDCS + Rehabilitation (5/5) vs. Rehabilitation (7/3)	40.25 ± 16.50

2015 Triccas (NCT01405378) *NeuroRehabilitation*	Anodal-tDCS + Rehabilitation (9) vs. Rehabilitation (9)	Anodal-tDCS + Rehabilitation (3) vs. Rehabilitation (2)	0.38 ± 0.49	Anodal-tDCS + Rehabilitation (6/6) vs. Rehabilitation (6/5)	32.3 ± 16.6
2018 Shaheiwola (ChiCTR-ICR-15006108) *frontiers in Human Neuroscience*	NR	NR	NA	Dual-tDCS + Rehabilitation (7/8) vs. Rehabilitation (9/6)	17.05 ± 10.77
2019 Salazar (NCT02818608) *Annals of Physical and Rehabilitation Medicine*	Dual-tDCS + Rehabilitation (14) vs. Rehabilitation (11)	Dual-tDCS + Rehabilitation (1) vs. Rehabilitation (4)	0.49 ± 0.28	Dual-tDCS + Rehabilitation (7/8) vs. Rehabilitation (7/8)	27.77 ± 9.71
2015 Rocha (NCT01879787) *Disability and Rehabilitation*	NR	NR	0.58 ± 0.32	Anodal-tDCS + Rehabilitation (4/3) vs. Cathodal-tDCS + Rehabilitation (4/3) vs. Rehabilitation (3/4)	49.07 ± 6.68

2017 Pavlova<br>(-)<br>*Restorative Neurology and Neuroscience*	Anodal-tDCS + Rehabilitation (4) vs. Rehabilitation (2)	Anodal-tDCS + Rehabilitation (1) vs. Rehabilitation (4)	5.55 ± 9.94	Anodal-tDCS + Rehabilitation (3/2) vs. Rehabilitation (5/1)	29.73 ± 15.21
2021 Gottlieb<br>(-)<br>*NeuroRehabilitation*	LF-rTMS + Rehabilitation (13) vs. Rehabilitation (12)	LF-rTMS + Rehabilitation (1) vs. Rehabilitation (2)	0.12 ± 0.09	LF-rTMS + Rehabilitation (11/3) vs. Rehabilitation (9/5)	25.68 ± 21.18
2020 Chiu<br>(-)<br>*Journal of Stroke and Cerebrovascular Diseases*	Dual-rTMS + Rehabilitation (14) vs. Rehabilitation (15)	Dual-rTMS + Rehabilitation (0) vs. Rehabilitation (0)	NA	NA	NA
2021 Chen<br>(NCT03350087)<br>*Journal of NeuroEngineering and Rehabilitation*	iTBS + Rehabilitation (6) vs. Rehabilitation (2)	iTBS + Rehabilitation (6) vs. Rehabilitation (9)	0.54 ± 0.92	iTBS + Rehabilitation (5/7) vs. Rehabilitation (4/7)	39.26 ± 11.08

2010 Chang (-) *Journal of Rehabilitation Medicine*	HF-rTMS + Rehabilitation (18) vs. Rehabilitation (10)	HF-rTMS + Rehabilitation (0) vs. Rehabilitation (0)	13.44 ± 5.40	HF-rTMS + Rehabilitation (7/11) vs. Rehabilitation (6/4)	26.00 ± 17.05
2020 Chen (ChiCTR1800019757) *Journal of International Medical Research*	LF-rTMS + Rehabilitation (16) vs. rPMS + Rehabilitation (19)	LF-rTMS + Rehabilitation (0) vs. rPMS + Rehabilitation (0)	NR	LF-rTMS + Rehabilitation (7/9) vs. rPMS + Rehabilitation (10/9)	16.00 ± 13.55
2020 Obayashia (-) *NeuroRehabilitation*	rPMS + Rehabilitation (8) vs. Rehabilitation (8)	rPMS + Rehabilitation (2) vs. Rehabilitation (1)	0.14 ± 0.12	NR	16.68 ± 13.32
2014 Krewer (-) *Archives of Rehabilitation Research and Clinical Translation*	rPMS + Rehabilitation (28) vs. Rehabilitation (32)	rPMS + Rehabilitation (3) vs. Rehabilitation (0)	0.61 ± 1.47	rPMS + Rehabilitation (18/13) vs. Rehabilitation (15/17)	NA

2019 Chen (NCT01947413) *BMC Neurology*	iTBS + Rehabilitation (2) vs. Rehabilitation (3)	iTBS + Rehabilitation (9) vs. Rehabilitation (8)	NR	iTBS + Rehabilitation (5/6) vs. Rehabilitation (2/9)	31.68 ± 11.08
2016 Du (-) *European Journal of Neurology*	HF-rTMS + Rehabilitation (23) vs. LF-rTMS + Rehabilitation (23) vs. Rehabilitation (23)	HF-rTMS + Rehabilitation (0) vs. LF-rTMS + Rehabilitation (0) vs. Rehabilitation (0)	8.01d ± 3.89d	HF-rTMS + Rehabilitation (23) vs. LF-rTMS + Rehabilitation (23) vs. Rehabilitation (23)	24.82 ± 14.97
2020 Miu (-) *Annals of Rehabilitation Medicine*	LF-rTMS + Rehabilitation (19) vs. Cathodal-tDCS + Rehabilitation (18)	LF-rTMS + Rehabilitation (6) vs. Cathodal-tDCS + Rehabilitation (8)	NR	LF-rTMS + Rehabilitation (8/17) vs. Cathodal-tDCS + Rehabilitation (14/12)	NR
2017 Guan (NCT03163758) *CNS Neuroscience & Therapeutics*	HF-rTMS + Rehabilitation (21) vs. Rehabilitation (21)	HF-rTMS + Rehabilitation (0) vs. Rehabilitation (0)	4.30d ± 3.75d	HF-rTMS + Rehabilitation (10/11) vs. Rehabilitation (9/12)	39.15 ± 9.41

2014 Galvao (NCT01875536) *Archives of Physical Medicine and Rehabilitation*	LF-rTMS + Rehabilitation (9) vs. Rehabilitation (8)	LF-rTMS + Rehabilitation (1) vs. Rehabilitation (2)	4.45 ± 2.97	LF-rTMS + Rehabilitation (7/3) vs. Rehabilitation (3/7)	23.55 ± 12.41
2013 Hsu (NCT01323881) *Brain Stimulation*	iTBS + Rehabilitation (6) vs. Rehabilitation (6)	iTBS + Rehabilitation (0) vs. Rehabilitation (0)	21.40d ± 11.08d	iTBS + Rehabilitation (6) vs. Rehabilitation (6)	36.4 ± 11.08
2018 Harvey (NCT02089464) *Stroke*	LF-rTMS + Rehabilitation (103) vs. Rehabilitation (54)	LF-rTMS + Rehabilitation (29) vs. Rehabilitation (13)	NR	LF-rTMS + Rehabilitation (63/120) vs. Rehabilitation (31/58)	34.3 ± 12.3
2021 Kuzu (NCT04562415) *Journal of Stroke and Cerebrovascular Diseases*	LF-rTMS + Rehabilitation (7) vs. cTBS + Rehabilitation (6) Rehabilitation (6)	LF-rTMS + Rehabilitation (0) vs. cTBS + Rehabilitation (0) Rehabilitation (0)	1.26 ± 0.18	LF-rTMS + Rehabilitation (7) vs. cTBS + Rehabilitation (6) Rehabilitation (6)	17.70 ± 11.06

2020 Kim<br>(KCT0001768)<br>*Brain Science*	LF-rTMS + Rehabilitation (8) vs. Rehabilitation (12)	LF-rTMS + Rehabilitation 0) vs. Rehabilitation (0)	NR	LF-rTMS + Rehabilitation (3/5) vs. Rehabilitation (4/8)	39.90 ± 24.07
2020 Kim<br>(NCT02082015)<br>*Neurorehabilitation and Neural Repair*	LF-rTMS + Rehabilitation (36) vs. Rehabilitation (37)	LF-rTMS + Rehabilitation (0) vs. Rehabilitation (0)	NR	LF-rTMS + Rehabilitation (36) vs. Rehabilitation (37)	40.69 ± 17.74
2015 Matsuura<br>(UMIN000016021)<br>*European Journal of Neurology*	LF-rTMS + Rehabilitation (10) vs. Rehabilitation (10)	LF-rTMS + Rehabilitation (0) vs. Rehabilitation (0)	9.60d ± 4.13d	LF-rTMS + Rehabilitation (5/5) vs. Rehabilitation (5/5)	49. 94 ± 2.59
2018 Long<br>(-)<br>*Journal of Stroke and Cerebrovascular Diseases*	LF-rTMS + Rehabilitation (11) vs. Dual-rTMS + Rehabilitation (11) vs. Rehabilitation (10)	LF-rTMS + Rehabilitation (10) vs. Dual-rTMS + Rehabilitation (10) vs. Rehabilitation (10)	19.48d ± 2.67d	LF-rTMS + Rehabilitation (11/10) vs. Dual-rTMS + Rehabilitation (11/10) vs. Rehabilitation (11/9)	35.07 ± 4.69

2014 Rose<br>(-)<br>*Stroke Research and Treatment*	LF-rTMS + Rehabilitation (9) vs. Rehabilitation (10)	LF-rTMS + Rehabilitation (0) vs. Rehabilitation (0)	5.14 ± 4.02	LF-rTMS + Rehabilitation (3/6) vs. Rehabilitation (6/4)	39.18 ± 9.58
2019 Pinto<br>(NCT02208466)<br>*Neurorehabilitation and Neural Repair*	LF-rTMS + Rehabilitation (9) vs. Rehabilitation (18)	LF-rTMS + Rehabilitation (0) vs. Rehabilitation (0)	0.81 ± 0.67	LF-rTMS + Rehabilitation (6/3) vs. Rehabilitation (10/8)	26.3 ± 12.93
2020 Meng<br>(-)<br>*Restorative Neurology and Neuroscience*	iTBS + LF-rTMS + Rehabilitation (5) vs. LF-rTMS + Rehabilitation (7) vs. Rehabilitation (4)	iTBS + LF-rTMS + Rehabilitation (5) vs. LF-rTMS + Rehabilitation (3) vs. Rehabilitation (4)	0.10 ± 0.02	iTBS + LF-rTMS + Rehabilitation (5/5) vs. LF-rTMS + Rehabilitation (6/4) vs. Rehabilitation (4/4)	21.49 ± 6.10
2015 Zheng<br>(UMIN000018819)<br>*Journal of Huazhong University of Science and Technology*	LF-rTMS + Rehabilitation (34) vs. Rehabilitation (35)	LF-rTMS + Rehabilitation (24) vs. Rehabilitation (19)	19.01d ± 11.08d	LF-rTMS + Rehabilitation (32/23/3) vs. Rehabilitation (33/19/2)	NA

2018 Watanabe (UMIN000018819) *Journal of the Neurological Sciences*	iTBS + Rehabilitation (8) vs. LF-rTMS + Rehabilitation (7) vs. Rehabilitation (6)	iTBS + Rehabilitation (0) vs. LF-rTMS + Rehabilitation (0) vs. Rehabilitation (0)	NR	iTBS + Rehabilitation (4/4) vs. LF-rTMS + Rehabilitation (3/4) vs. Rehabilitation (5/1)	13.68 ± 11.08
2020 Wang (ChiCTR-IPR-17013580) *Brain Stimulation*	HF-rTMS + Rehabilitation (11) vs. LF-rTMS + Rehabilitation (10) vs. Rehabilitation (9)	HF-rTMS + Rehabilitation (4) vs. LF-rTMS + Rehabilitation (5) vs. Rehabilitation (6)	25.57d ± 16.30d	HF-rTMS + Rehabilitation (7/8) vs. LF-rTMS + Rehabilitation (8/7) vs. Rehabilitation (7/8)	19.82 ± 9.21
2017 Tosun (-) *Topics in Stroke Rehabilitation*	LF-rTMS + Rehabilitation (9) vs. Cyclic NMES + LF-rTMS + Rehabilitation (7) vs. Rehabilitation (9)	LF-rTMS + Rehabilitation (0) vs. Cyclic NMES + LF-rTMS + Rehabilitation (0) vs. Rehabilitation (0)	0.14 ± 0.13	LF-rTMS + Rehabilitation (6/3) vs. Cyclic NMES + LF-rTMS + Rehabilitation (3/4) vs. Rehabilitation (4/5)	25. 47 ± 15.66
2013 Sung (-) *Stroke*	iTBS + LF-rTMS + Rehabilitation (10) vs. iTBS + Rehabilitation (8) vs. LF-rTMS + Rehabilitation (8) vs. Rehabilitation (9)	iTBS + LF-rTMS + Rehabilitation (5) vs. iTBS + Rehabilitation (4) vs. LF-rTMS + Rehabilitation (5) vs. Rehabilitation (5)	0.67 ± 0.92	NR	29.06 ± 11.08

2020 Sharma<br>(-)<br>*Archives of Rehabilitation Research and Clinical Translation*	LF-rTMS + Rehabilitation (47) vs. Rehabilitation (49)	LF-rTMS + Rehabilitation (0) vs. Rehabilitation (0)	4.82d ± 1.41d	NR	45.25 ± 16.56
2012 Seniów<br>(-)<br>*Neurorehabilitation and Neural Repair*	LF-rTMS + Rehabilitation (19) vs. Rehabilitation (19)	LF-rTMS + Rehabilitation (1) vs. Rehabilitation (1)	0.11 ± 0.07	LF-rTMS + Rehabilitation (10/10) vs. Rehabilitation (13/7)	38.2 ± 14.33
2013 Abo<br>(-)<br>*International Journal of Stroke*	LF-rTMS + Rehabilitation (18) vs. Rehabilitation (11)	LF-rTMS + Rehabilitation (26) vs. Rehabilitation (11)	5.34 ± 4.10	LF-rTMS + Rehabilitation (25/19) vs. Rehabilitation (12/10)	47.7 ± 10.33
2008 Chan<br>(-)<br>*Neurorehabilitation and Neural Repair*	NR	NR	1.26 ± 1.18	FES + Rehabilitation (4/6) vs. SES + Rehabilitation (5/5)	19.10 ± 8.90

NR: Not reported; NA: Not achieve; EOT: End of treatment.

C. Inclusion criteria, exclusion criteria, details of neurostimulation, efficacy outcomes, safety outcomes and conclusions of included studies.

Trials	Brown et al. 2008<br>(——)
**Inclusion criteria**	Patients 20 to 75 years.<br>Ischemic infarct, either cortical or capsular, that occurred at least 4 months before enrollment and demonstrated on computerized tomography or magnetic resonance imaging.<br>An Upper Extremity Fugl-Meyer Assessment score between 20 and 50, inclusive sufficient to allow active wrist extension of at least 5 degrees.
**Exclusion criteria**	Another stroke preceded their index stroke and was associated with incomplete motor recovery.<br>There was a history of spinal cord injury, significant traumatic brain injury (such that associated with loss of consciousness and memory loss), or a subdural or epidural hematoma.<br>They had any history of seizures or were taking anticonvulsants to treat seizures.<br>There was any significant central nervous system disease state.<br>They were not considered candidates for surgery to implant the device.
**Details of neurostimulation**	At the beginning session each week of treatment, threshold for evoking gross movement in the contralateral hand was determined using stimulation parameters of 3 second trains of 50 Hz, 250 ms pulses starting at 1 mA and increasing until either movement was evoked or a maximum of 15 mA was reached. Stimulation was set at either 50% of movement threshold (if

	movement was evoked) or 6.5 mA (if no movement was evoked). Biphasic stimulation pulses were delivered with 250 ms first-phase durations and decaying exponential second phases.
**Efficacy outcomes**	Upper Extremity Fugl-Meyer Scale (UEFM)<br>Grip strength<br>Action Research Arm test (ARM)<br>9-hole Pegboard Test<br>Tapping speed<br>National Institutes of Health Stroke Scale (NIHSS)<br>Stroke Impact Scale (SIS)<br>Activities of daily living<br>Functional Independence Measure (FIM)<br>Neurological Function questionnaire<br>Mental status<br>Beck Depression Inventory
**Safety outcomes**	No patient deaths occurred. No patient demonstrated new neurological deficits during the period of assessment. There were no seizures during study participation for any patient, in either study group. Two complications occurred, both infectious.
**Conclusions**	This safety study demonstrates that cortical stimulation can be safely performed in apopulation of patients with cerebrovascular disease who are at risk for surgical morbidity. Preliminary motor assessment data also show that intermittent cortical stimulation delivered during periods of rehabilitation activity does enhance upper extremity functional recovery when compared with control groups of patients whoreceive only rehabilitation.

Trials	Huang et al 2008 (——)
**Inclusion criteria**	At least 4 months from a cortical or subcortical ischemic stroke, were at least 21 years of age, could comply with the rehabilitation therapy protocol, had moderate to moderately severe hemiplegia as defined by an UEFM score between 20 and 50.
**Exclusion criteria**	Patients with hemorrhagic stroke, seizure disorder, moderate to severe neglect, recent changes in any drugs that could impact the central nervous system (neurostimulants or spasticity agents) .
**Details of neurostimulation**	Bipolar stimulator output during therapy was programmed via telemetry to 50% of motor threshold or up to a maximum current of 6.5 mA for each investigational patient. Cortical stimulation was initiated 5 minutes prior to the start of each rehabilitation session and was terminated at the end of the session. In Protocol 1, the investigational patients received stimulation of 50 Hz with pulse duration of 250 μsec. In Protocol 2, investigational patients received 101 Hz stimulation with pulse duration of 250 μsec.
**Efficacy outcomes**	Upper Extremity Fugl-Meyer Scale (UEFM)<br>Arm Motor Ability Test (AMAT)<br>National Institutes of Health Stroke Scale (NIHSS)<br>Stroke Impact Scale (SIS)<br>Grip strength<br>Box and Block Test (B&B)<br>Mini-Mental Status Examination (MMSE)<br>Beck Depression Inventory (BDI)
**Safety outcomes**	No adverse events occurred in any investigational patient during cortical stimulation in either Protocol 1 (50 Hz) or

	Protocol 2 (101 Hz). One seizure occurred after device implantation but prior to the delivery of cortical stimulation. The participant was treated with valproic acid and completed the research study with no further events. Following the study, the anticonvulsant was discontinued without recurrent seizures. No other seizures occurred in any of the investigational patients. Other complications reported in the investigational groups were associated with device implantation/explanation and included two reports each of swelling after surgery, pain at the implant site, and headache. There was one report of bleeding at the incision site, fever, allergic reaction to surgical tape, and temporary mild tingling/numbness in the jaw area. There was one report of the temporary anesthesia-related events in treatment patients: unstable blood pressure, nausea/vomiting, and urinary retention. All complications were minor in nature and resolved quickly without any additional treatment.
**Conclusions**	Evidence from two small feasibility trials of cortical stimulation for stroke-related upper limb hemiplegia suggests that targeted bipolar stimulation to perilesional motor cortex given concurrently with intensive rehabilitation therapy may result in lasting improvement in affected upper limb motor control, in certain measures of impairment, and is not associated with serious adverse neurologic sequelae.

**Trials**	**Levy et al. 2008**<br>(——)

**Inclusion criteria**	Patient age ≥21 yrs.<br>Ischemic infarct, either cortical or capsular, that occurred ≥4 mos prior to enrollment, & demonstrated on computed tomography or MR imaging.<br>UEFM score between 20 & 50 inclusive, sufficient to allow active wrist extension of ≥5℃.
**Exclusion criteria**	Hemorrhagic stroke.<br>Stroke preceding index stroke associated w/ incomplete motor recovery.<br>Severe sensory deficit, or moderate to severe hemispatial neglect &/or anosognosia.<br>History of spinal cord injury, significant traumatic brain injury (such as associated w/ loss of consciousness & memory loss), or a subdural or epidural hematoma.<br>History of seizures or intake of anticonvulsants to treat seizuresany other significant central nervous system disease.<br>Not considered a candidate to undergo device implantation surgery.
**Details of neurostimulation**	For each assessment of stimulator output, the current level was adjusted in 3-second pulse trains to determine the minimum current required to elicit motor movement. The target stimulation level used during therapy was 50% of the movement threshold. If movement was not elicited during threshold testing, the stimulation level was set to a maximum of 6.5 mA, the maximum output current allowed under the investigational protocol. Stimulation was delivered at a pulse repetition frequency of either 50 or 101 Hz with a pulse duration of 250 msec.
**Efficacy**	Upper Extremity Fugl-Meyer Scale (UEFM)

**outcomes**	Arm Motor Ability Test (AMAT)<br>National Institutes of Health (NIH)<br>Beck Depression Inventory (BDI)<br>Modified Rankin Scale (MRS)
**Safety outcomes**	A secondarily generalized seizure occurred in 1 patient, 36 hours after implant surgery and prior to the delivery of CS. Another patient experienced a seizure 5 months after device explantation and ~ 12 months after the index stroke. This event was considered attributable to the patient's underlying stroke condition and disease state and not to the investigational device or study procedures. Other medical complications reported during the study were various anticipated surgery-related complications in patients in the treatment group. All complications were minor in nature and resolved quickly without any additional treatment.
**Conclusions**	This study demonstrates that CS can be safely performed in a population of patients with cerebrovascular disease who are at risk for surgical morbidity. Motor assessment data show that CS delivered during periods of active rehabilitation appears to enhance upper-extremity functional recovery when compared with control groups of patients who receive rehabilitation alone. Improvements in motor function seem to translate into improvement in ADLs and quality of life.

**Trials**	**Levy et al. 2016<br>(NCT00170716)**

**Inclusion criteria**	Male and female subjects at least 21 years old with moderate to moderately severe upper extremity hemiparesis due to an ischemic infarct that occurred ≥4 months prior to enrollment were considered for this study. Moderate to moderately severe impairment was defined as a score of 28 to 50 (inclusive) on the upper extremity Fugl-Meyer scale (UEFM).
**Exclusion criteria**	Subjects with a primary hemorrhagic stroke, severe sensory deficit, or moderate to severe hemispatial neglect and/or anosognosia; or a history of seizures were excluded from this study.
**Details of neurostimulation**	The IPG output current level was adjusted in 1-mA increments up to 13 mA at 50 Hz and 250 μs to determine the minimum current level required to elicit visible or palpable hand or arm muscle response (ie, MT). The stimulation amplitude used during rehabilitation therapy was 50% of the MT. If no muscle response could be induced during testing, the stimulator was set at 6.5 mA, which is 50% of maximum pulse generator output. These stimulation parameters were unchanged throughout the rehabilitation intervention. EECS was initiated approximately 5 minutes prior to the start of each rehabilitation session, and was discontinued at the end of each session.
**Efficacy outcomes**	Upper Extremity Fugl-Meyer Scale (UEFM)<br>Arm Motor Ability Test (AMAT)
**Safety outcomes**	There were 40 SAEs (anticipated SAEs = 11; unanticipated SAEs = 29) in 25investigational group subjects, and the likelihood of a patient in the investigational group having any SAE was 24% (95% CI 16% to 33%). A majority of these SAEs (77%) occurred either during implant (32%) or post-rehabilitation (45%). Eleven SAEs occurred in 9 subjects that

	were related to implantation or removal of the study device (Tables 5 and 6). Three of these events resulted in early surgical removal of the device, due to wound infection at device implant site, persistent headache after implant, and bleeding between skull and brain.
**Conclusions**	The Everest phase III trial was unsuccessful in attaining its primary efficacy endpoint at 4 weeks post-treatment. Cortical stimulation delivered during task-oriented upper limb rehabilitation did not result in significantly higher functional motor gains in investigational group patients when compared to the control group patients who underwent only rehabilitation.

**Trials**	**Amasyali et al. 2016**<br>(——)

**Inclusion criteria**	(i) They had had an ischemic stroke during the previous 12 months, (ii) were between 20 and 85 years old, (iii) they could understand simple verbal instructions (Mini-Mental State Examination score>21), (iv) had a Brunnstrom score between stage 2 and 5 for the hand, and (v) had a modified Ashworth scale score less than 3.
**Exclusion criteria**	No mention of it.
**Details of neurostimulation**	Pulse duration (200 μs), frequency (50 Hz), and other treatment parameters (1 s ramp up, 5 s biphasic stimulation, 1 s ramp down) were adjusted.
**Efficacy outcomes**	Upper limb Fugl-Meyer Assessment (FMA),<br>Wrist range of motion,<br>Grip force,<br>Box and Block Test (BBT).
**Safety outcomes**	No mention of it.
**Conclusions**	MT was more efficient in improving motor performance than physiotherapy alone and also MT may be more useful in improving hand skills compared with EMG-stim. The fact that, compared with CG, time since stroke in the MT group was 2 months earlier should not be ignored and could be one of the factors that contributed toward better recovery of the patients. Some advantages such as practice after discharge by the patients themselves without any observer have led to a greater increase in the popularity of this inexpensive modality in recent years.

**Trials**	**Boyaci et al. 2013**<br>(——)

**Inclusion criteria**	Post-stroke period ≥ 4 week<br>Between 18 and 80 years of age<br>The ability to understand and communicate<br>No visual or auditory defect<br>Adequately motivated and willing to participate<br>Medically stable condition<br>The ability to voluntarily extend the wrist
**Exclusion criteria**	Previous hemiparesis<br>Flaccid hemiplegia<br>Volitional wrist extension in synergy or in isolation with muscle grade ≥ 3/5<br>Spasticity > stage 3 according to the modified Ashworth scale<br>Deformity leading to a upper extremity dysfunction<br>Neurological comorbidity leading to an impaired upper extremity<br>Cardiac pacemaker<br>History of seizures within the previous 2 years<br>History of potentially fatal cardiac arrhythmia
**Details of neurostimulation**	EMG-triggered NMES: The sensitivity of the EMG biofeedback ranged from 0 to 100 mV. The settings for the electrical stimulation were 2-s ramp up, 10 s of symmetric biphasic stimulation at 50 Hz (mA 20–47, pulse width of 200 μs), and 2-s ramp down.<br>Cyclic NMES: The stimulation current amplitude was set to produce full wrist and finger extension with a duty cycle of 10 s on and 15 s off (symmetric biphasic stimulation at 50 Hz, 2-s ramp up and ramp down, 20–47 mA, pulse width 200 ms).
**Efficacy outcomes**	UE motor subscore of the Fugl-Meyer Motor Assessment (FMA)

	Functional Independence Measure (FIM)<br>Motor Activity Log (MAL)<br>The amount of use (AOU)<br>Quality of movement (QOM)<br>The modified Ashworth scale
**Safety outcomes**	NMES application was well tolerated and there were no complications or patient complaints. Side effects were not recorded.
**Conclusions**	The findings of the present study suggest that electrical neuromuscular therapy might be beneficial in rehabilitation programs because NMES (both active and passive) combined with neurophysiologic exercise treatment improved paretic UE motor function and decreased functional dependence in subacute and chronic stroke patients.

**Trials**	**Chuang et al. 2017**<br>**(NCT01913509)**

**Inclusion criteria**	(1) First-ever stroke with onset >3 months prior at time of recruitment; (2) at least mild intensity of hemiplegic shoulder pain with activity in the past 7 days(Numerical Rating Scale score ≥ 1); (3) no other neurological disorders, such as Parkinson's disease, epilepsy, multiple sclerosis, etc.; (4)adequate cognitive ability (Mini-Mental State Examination score ≥ 24).
**Exclusion criteria**	(1) Contraindications for electrical stimulation (e.g., metal implants, cardiac pacemaker); (2) pre-existing pathology of the shoulder, such as rotator cuff injury or tendonitis, frozen shoulder, etc.; (3) participation in any experimental rehabilitation or drug studies during the study period; (4) change of pain medication during the study period; (5) treatment of upper limb spasticity, including botulinum toxin injection or neurolytic or surgical procedures; (6) aphasia; and (7) severe cognitive deficits.
**Details of neurostimulation**	NMES: The range of intensities used to stimulate the muscles was 3–5 out of 10. The contraction-relaxation ratio of EMG-triggered NMES was adjusted progressively from 10/10 s to 30/10 s<br>TENS: The control group received TENS on the supraspinous fossa and posterior deltoid muscles of the painful shoulder, which was performed by a portable stimulator unit (SW320, Shining World Health Care Co., LTD., Taiwan) at a frequency of 30 Hz.
**Efficacy outcomes**	Vertical Numerical Rating Scale supplemented with a Faces Rating Scale (NRS-FRS)<br>The short form of the Brief Pain Inventory (BPI-SF)

	The upper-limb subscale of the Fugl-Meyer Assessment (FMA-UL)<br>Pain-free passive shoulder range of motion
**Safety outcomes**	No harms or unintended effects were reported in either group.
**Conclusions**	EMG-triggered NMES combined with bilateral arm training was better than TENS with bilateral arm training for reducing hemiplegic shoulder pain during movement, lessening the worst shoulder pain, and improving pain-free shoulder abduction and internal rotation for stroke patients with hemiplegic shoulder pain. Such improvements appear to be sustained beyond the immediate time frame of the treatment.

**Trials**	**Jong et al. 2013**

	**(NTR1748)**
**Inclusion criteria**	First-ever or recurrent stroke (except subarachnoid haemorrhages) between two and eight weeks post-stroke; age > 18 years; paralysis or severe paresis of the affected arm scoring 1–3 on there covery stages of Brunnstrom (1970); and no planned date of discharge within four weeks.
**Exclusion criteria**	Contraindications for electrical stimulation (eg. metal implants, cardiac pacemaker); preexisting impairments of the affected arm (pre-existing contracture was not an exclusion criterion); severe cognitive deficits and/or severe language comprehension difficulties, defined as < 3/4 correct verbal responses and/or < 3 correct visual graphic rating scale scores on the Ability Q (Turner Stokes and Rusconi 2003); and moderate to good arm motor control (> 18 points on the Fugl-Meyer Assessment arm score).
**Details of neurostimulation**	First, electrodes were placed over the motor point of the extensor digitorum communis muscle and the dorsal surface of the distal forearm. Two other electrodes were placed over the dorsal surface (pars posterior) of the deltoid muscle and over the infraspinatus/teres minor muscle(s). The electrical stimulatorb was programmed to deliver a symmetric bi-phasic wave pattern of 35 Hz and a pulse width of 300μs. Stimulation on and off time was 8 seconds with a 3 second ramp-up and ramp-down. For skin adaptation purposes the stimulation time was gradually increased from 10 to 45 min per session (with increments of 5 min every other day) during the first 13 days. Amplitudes never exceeded the individual's comfort level and skin checks were performed after every session.

	The basic stimulation (80Hz, pulse width 150µs) was just above the participants' sensation threshold, giving no muscle contraction but a tingling sensation at the most. Subsequently, the affected arm was positioned in shoulder internal rotation and ≤ 30 degrees of abduction.
**Efficacy outcomes**	The primary outcome measures were passive range of arm motion and pain in the hemiplegic shoulder. The secondary outcome measures were timing and severity of post-stroke shoulder pain, performance of real-life passive and basic daily active arm activities, hypertonia and spasticity, arm motor control and shoulder subluxation.
**Safety outcomes**	The most common residual impairments such as contractures, hypertonia, and spasticity and its associated secondary problems such as shoulder pain and restrictions in performance of daily life activities.
**Conclusions**	In people with poor arm motor control in the subacute phase after stroke, static stretch positioning combined with simultaneous NMES has no statistically significant effects on range of motion, shoulder pain, basic arm function, or activities of daily living.

Trials	Kroon et al. 2008<br>（——）
**Inclusion criteria**	(1) An interval of more than six months since unilateral supratentorial stroke (infarction or haemorrhage); (2) between 18 and 80 years of age; (3) impaired function of the upper extremity due to spastic paresis (spasticity was defined as a synergistic movement pattern or an Ashworth Score of 1 or more; paresis was defined as wrist extensor strength grade 4 or less (Medical Research Council); (4) voluntary extension of wrist (at least 10from resting position);(5)stable general health status; (6) written informed consent.
**Exclusion criteria**	(1) A cardiac pacemaker (on demand); (2) an epileptic seizure less than six months before the start of stimulation; (3) metal implants in the affected arm; (4) pre-existent functional limitations of the affected upper extremity; (5) serious contractures of shoulder, elbow or wrist (clinical assessment); (6) severe cognitive impairments or severe aphasia resulting in inability to understand the trial; (7) skin problems underneath the electrodes; (8) inadequate motor response to test stimulus; (9) not enough voluntary muscle contraction of wrist extensors to trigger stimulation; (10) no tolerance for surface stimulation. Criteria 8, 9 and 10 were assessed during a single test session with both modes of stimulation before inclusion and randomization.
**Details of neurostimulation**	In either mode biphasic pulses with a frequency of 35 Hz and pulse duration of 300 ms were administered for 6 seconds with 1-second ramp-up, 1-second ramp-down and 9-second stimulus off.
**Efficacy**	Action Research Arm test

**outcomes**	Fugl-Meyer Motor Assessment<br>Motricity Index.
**Safety outcomes**	Only in the first days of the treatment period, a few subjects experienced some temporary redness of the skin under the electrodes or pain during stimulation, both related to stimulation amplitude; others had shoulder complaints related to the position of shoulder and arm during stimulation or some muscular pain after stimulation. Apart from these temporary complaints, no adverse effects were reported.
**Conclusions**	The present study did not detect a significant difference between EMG-triggered and cyclic electrical stimulation with respect to improvement of motor function of the affected arm in chronic stroke.

Trials	Hemmen et al. 2007 (——)
**Inclusion criteria**	Central paresis of the arm/hand after a first-ever stroke, post-stroke period 3 weeks, active wrist muscle strength between grade 2 and 3 on the MRC (Medical Research Council) scale, fair cognitive level (Mini-Mental State Examination score 24),no additional severe rheumatologic, neurological or orthopaedic problems prior to the stroke, no pacemaker or severe cardiopulmonary complications, no history of epilepsy.
**Exclusion criteria**	Excluding one participant whose post-stroke time was 222 days
**Details of neurostimulation**	For the EMG-triggered feedback stimulation, the Danmeter Automove AM800 (Danmeter A/S, Odense,Denmark) was used in combination with Biostim CF5050 pre-wired, reusable surface electrodes (Biomedical Life Systems, Vista, CA, USA). For the conventional electrostimulation, the Automove AM800 was also used, but without the EMG-triggered feed back function. Optimal electrode location on the paretic forearm – where the wrist extensor muscles contracted clearly during application of either stimulation technique – had been established in a pilot study prior to this study. EMG/stimulation electrodes were placed over the wrist extensor muscles. The reference electrode was placed at an electrically neutral position near the elbow joint. Each subject sat in front of a table.
**Efficacy outcomes**	The upper extremity-related part of the Brunnstrom Fugl-Meyer test<br>the Action Research Arm test
**Safety outcomes**	Four participants from the reference group failed to complete the three-month training programme due to medical complications,

	which included recurrent stroke, severe pain due to shoulder–hand syndrome or lumbar spine disease. At one-year follow-up, one participant from the experimental group was excluded due to stroke recurrence. No spasticity was present in any of the participants.
**Conclusions**	EMG-triggered feedback stimulation did not lead to more arm–hand function improvement relative to conventional electro stimulation. However, in contrast to many clinical reports, a significant improvement was still observed in both groups nine months after treatment ceased.

Trials	Jeon et al. 2016 (——)
**Inclusion criteria**	1) Had no pathological disease in the shoulder joint before the onset, 2) has shoulder subluxation of 9.5 mm or greater (Hall, Dudgeon, & Guthrie, 1995), 3) diagnosed with stroke and has the MMT Grade 2 higher, 4) less than 6 months from the onset, 5) Mini-Mental Status Examination (MMSE) score of 21 or higher, 6) has no cardiac pacemaker or metal transplant, 7) understands the objective of this study and consents to participate in the study, 8) is not participating in an experiment similar to this study.
**Exclusion criteria**	No mention of it
**Details of neurostimulation**	The threshold for electric stimulation was set according to the maximum contraction intensity of each patient. Symmetric biphasic pulse was set at 35 Hz, single pulse of 200μs, phase duration of 10 seconds, rest period of 10 seconds, ramp up of 5 seconds, and ramp down of 5 seconds. The stimulation intensity ranged between 10~30 mA.
**Efficacy outcomes**	The degree of shoulder subluxation which had been confirmed by X-ray, muscleactivation of the supraspinatus and posterior deltoid muscles by electromyography, pain by the Visual Analogue Scale(VAS), and hand function by the Fugl-Meyer Assessment (FMA)
**Safety outcomes**	No mention of it
**Conclusions**	Task-oriented electromyography-triggered stimulation improved shoulder subluxation, muscle activation, pain and upper extremity function. These results suggest that task-

	oriented electromyography-triggered stimulation is effective and beneficial for individuals with subacute stroke.

Trials	Wilson et al. 2016 (NCT00142792)
**Inclusion criteria**	(1) Medically stable; (2) non-hospitalized adults within 6 months of stroke; (3) age 21 to 89 years; (4) required to have intact skin on the hemiparetic arm; (5) adequate cognition to participate (follow 3-step commands, recall 2 of 3 items at 30 minutes); (6) full passive range of motion of the wrist, thumb, index, and long finger metacarpophalangeal joints of the affected limb; (7) less than full strength of the affected limb; (8) a score less than 12 out of 14 points on the hand portion of the upper-extremity section of the Fugl-Meyer Assessment (FMA); (9) detectable, volitionally activated EMG signals from the wrist or finger extensors (extensor carpi radialis [ECR] or extensor digitorum communis [EDC] muscles); (10) the ability to tolerate NMES for wrist and finger extension; and (11) the ability to set up and operate the assigned NMES device independently or with assistance.
**Exclusion criteria**	(1) Insensate affected arm; (2) uncompensated hemineglect, as measured by testing for extinction on double simultaneous stimulation (visual or tactile) or by drawing a clock face; (3) history of cardiac arrhythmia with hemodynamic instability; (4) premorbid upper motor neuron lesion affecting the hemiparetic arm; (5) uncontrolled seizure disorder; (6) metacarpophalangeal joint or wrist pain to palpation or with extension; (7) implanted stimulator (such as cardiac pacemaker); and (8) pregnancy.
**Details of neurostimulation**	Stimulation was delivered via a biphasic waveform, with a pulse duration of 300 µs. Pulse frequency was adjusted to participant comfort but kept between a range of 20 and 40 Hz. The duty cycle was initially set at 5 s on, 20 s off. The off time was

	decreased approximately every 3 days by 5 s, until the duty cycle was 5 s on and 5 s off.<br>The stimulation intensity was set above sensory threshold but below motor threshold. Stimulation frequency was set at 30 Hz.
**Efficacy outcomes**	The upper-extremity section of the FMA<br>The ability of a hemiparetic upper limb to execute specific activities<br>The functional ability component of the Arm Motor Ability Test (AMAT)
**Safety outcomes**	No mention of it
**Conclusions**	This multisite RCT demonstrated equal, statistically significant increases in upper-extremity motor impairment in subacute stroke survivors with upper-limb hemiparesis, with an 8-week treatment with cyclic NMES, EMG-triggered NMES, and sensory stimulation. There was no difference between groups based on the stimulation treatment paradigm, and treatment effects were negligible. Merely triggering NMES with EMG signal is not enough to improve on the outcomes achieved with nontriggered cyclic NMES or sensory stimulation. There was no evidence that time poststroke influenced response to treatment.

Trials	Carrico et al. 2016 (NCT02587234)
**Inclusion criteria**	Recruitment targeted subjects with mild-to-moderate UE motor deficit after a single ischemic stroke. BMild-tomoderate[was defined according to standard eligibility criteria for CIT(ie, able to extend the affected metacarpophalangeal and interphalangeal joints at least 10°; and the wrist,20°). Targeted subjects were adults (ie, 18 years of age and older) at least 12mo from stroke onset. Targeting this phase helped mitigate the potential confound of spontaneous motor recovery, which usually occurs within the first 12 mo after stroke onset.
**Exclusion criteria**	(a) History of carpal tunnel syndrome and documented peripheral neuropathy; (b) within 3 mo of recruitment, addition or change in the dosage of drugs known to exert detrimental effects on motor recovery; and (c) aphasia or cognitive deficit severe enough to preclude informed consent
**Details of neurostimulation**	EMG activity was amplified and filtered (bandpass, 10-3000 Hz). The stimulus intensity was adjusted to elicit small compound muscle action potentials of approximately 50 to 100 μV without the absence of visible muscle movements.<br>An identical protocol was implemented except that the amplitude was set to 0 V.
**Efficacy outcomes**	Wolf Motor Function Test (WMFT)<br>The Fugl-Meyer Assessment Scale (FMA; UE motor score only)<br>The Action Research Arm Test (ARAT)
**Safety outcomes**	No treatment complications or serious adverse events occurred during the study.
**Conclusions**	All outcome measures in this study reflected improvement in behavioralmeasures of UE activity for both groups after

	intervention. However, significantly more improvement was evident for the active PNS group compared with the sham PNS group. Overall, the results of this study provide a strong rationale for a full-scale investigation of the effects of PNS paired with a modified form of CIT for stroke survivors with chronic, mild-to-moderate UE hemiparesis.

**Trials**	**Fleming et al. 2015 (ISRCTN 05542931)**

**Inclusion criteria**	Age >65 years, unilateral upper limb weakness, physically able to participate (including being ambulant and able to negotiate a flight of stairs with assistance), completed upper limb rehabilitation and the presence of motor evoked potentials (MEPs) in response to Trans-cranial Magnetic Stimulation (TMS) with the muscles at rest or pre-activated.
**Exclusion criteria**	Contraindications to TMS such as epilepsy or seizures, cardiacpace makers or metal implants in the head, severe spasticity (Modified Ashworth Scale22≥4), dysphasia or cognitive dysfunction sufficient to limit ability to provide informed consent.
**Details of neurostimulation**	The stimulator delivered bursts of 10 Hz stimulation at 50% duty cycle (500 ms on and off).
**Efficacy outcomes**	Action Research Arm Test (ARAT)<br>Upper limb Fugl-Meyer Assessment (FM)<br>The Motor Activity Log (MAL)<br>Goal Attainment Scale (GAS)<br>MEP Analysis
**Safety outcomes**	There were no serious adverse effects. Minor ones included dermatitis (n=11) at the site of the active SS electrodes which resolved spontaneously (n=9) or with prescribed steroid cream (n=2) and mild shoulder pain (sham n=2, active n=1). One participant developed short-term nausea and light-headedness during TMS which was discontinued (remaining in the study without TMS).
**Conclusions**	SS combined with TST induces short term improvements in function, as measured by ARAT, compared with TST alone. However, the mechanisms underlying the effect of SS remain unknown.

**Trials**	**Ghaziani et al. 2018 (NCT02250365)**

**Inclusion criteria**	(a) Age ⩾18 years, (b) residence in the hospital's catchment area for stroke rehabilitation, (c) acute stroke confirmed by magnetic resonance imaging (MRI) or computed tomography scan, and (d) arm paresis (<66 points on the upper extremity section of the Fugl-Meyer Assessment, subscales A-D).
**Exclusion criteria**	(a) Contraindications to ESS (pacemaker, skin impairment), (b) inability to initiate the ESS within 7 days post-stroke due to medical or logistical issues, (c) cognitive dysfunctions or poor Danish communication skills that limited the ability to provide informed consent, (d) severe pre-stroke disability (modified Rankin Scale score = 5) (e) incomplete recovery of the affected arm after a previous stroke, and (f) participation in other biomedical intervention trials within the past 3 months.
**Details of neurostimulation**	The intervention group received suprasensory ESS delivered in continuous mode (pulse width = 250 µs, frequency = 10 Hz).<br>The placebo ESS consisted of suprasensory ESS delivered in intermittent mode (active stimulation intervals of 3 seconds delivered in loops of 2.5 minutes, pulse width = 250 µs, frequency = 10 Hz),
**Efficacy outcomes**	Box and Block Test (BBT); Fugl-Meyer Assessment (FMA), grip strength, pinch strength, perceptual threshold of touch, pain, and Modified Rankin Scale (MRS)
**Safety outcomes**	Adverse reactions (itch) to ESS were reported in 1 patient in each group and alleviated by using allergy friendly electrodes. The percentage of complications during the hospitalization was similar in both groups. Likewise, the percentage of recurrent stroke was comparable; 6% in the low-dose group and 4% in the high-dose group. The ESS intervention had no effect on the discharge destination or the participants' residence at follow-up.

**Conclusions**	Providing the present ESS protocol prior to arm training was equally beneficial as arm training alone. These results are valid for patients with mild-to-moderate stroke and moderate arm impairments. We cannot exclude benefits in patients with other characteristics, in other time intervals post-stroke or using a different ESS protocol.

**Trials**	**Pan et al. 2018 (NCT03277534)**

**Inclusion criteria**	(1) First-ever cerebral cortical region involved chronic stroke, onset over a month, (2) able to perform active thumb flexion on the affected side with the scores of manual muscle test at least two points, and(3) at stable medical condition for intervention confirmed by a specialized physician.
**Exclusion criteria**	(1) History of other neurological disorders, (2) cognitive impairment (Mini-Mental State Examination score<24, MMSE), (3) unable to follow instructions, (4) contraindications of ES, and (5) under 20 years old.
**Details of neurostimulation**	The subjects in ES group received 1 millisecond-rectangular pulse at 100Hz with a 20-second on 20-second of cycle. The total ES intervention time is 40minutes and the intensity was set at the highest tolerable level without pain or muscle twitch.
**Efficacy outcomes**	EEG, EMG and Fugl-Meyer Assessment (FMA)
**Safety outcomes**	No mention of it
**Conclusions**	Chronic stroke survivors who received eight weeks of ES prior to functional training can facilitate corticomuscular functional connectivity and hence improve upper limb function. Additionally, they bring important insights into the timing of neural and behavioral changes as well as support further studies applying peripheral electrical stimulation.

**Trials**	**Takebayashi et al. 2017 (UMIN000020927)**

**Inclusion criteria**	Age, 20–90 years; and with a first stroke in chronic stage (>180 days from stroke onset)
**Exclusion criteria**	Bilateral or brain stem infarct or hemorrhage; voluntary extension of the metacarpophalangeal and interphalangeal joints of three or more fingers ≤10° or voluntary wrist extension ≤20°; severe impairment in balance or walking, indicated by the need for assistance for standing, walking or using the toilet; substantial use of the affected upper extremity before the intervention, indicated by a score of >2.5 points on the amount of use (AOU) scale of the Motor Activity Log (MAL); clear signs of dementia or cognitive disorder, indicated by a score <24 points in the Mini Mental State Examination; severe aphasia or apraxia, preventing the patient from participating in the activities involved in the study intervention; presence of another uncontrolled medical condition or severe end-stage disease; and severe contraction in the area of the shoulder, elbow, wrist, or fingers.
**Details of neurostimulation**	Trains of electrical stimulation (20 Hz, on/off duty cycle 150/150μs; pulse duration, 300 μs) were applied at 1 Hz for 10 min.
**Efficacy outcomes**	Fugl-Meyer Assessment<br>Amount of use (AOU)<br>Quality of movement (QOM) scores,<br>Motor Activity Log
**Safety outcomes**	No adverse events were identified in either the treatment or control group.
**Conclusions**	Compared to behavioral and motor rehabilitation alone, non-invasive stimulation with dual-t DCS and PNMES followed by

	behavioral and motor treatment provides greater effectiveness to enhance the recovery of motor function and real-world use of the affected upper extremity in patients with chronic stroke. Therefore, in chronic stroke patients, the novel pre-treatment based on dual-t DCS and PNMES may enhance the therapeutic benefit of CIMT.

**Trials**	**Alwhaibi et al. 2021 (healthcare9050614)**

**Inclusion criteria**	Aged 40–65 years, diagnosed with first-time ischemic/hemorrhagic stroke by their treating neurologists and confirmed by MRI scans
**Exclusion criteria**	Intolerance to TENS acupoints; UE sensory deficits attributable to non-stroke pathology, such as diabetes or peripheral neuropathy; pre-stroke orthopedic or neurological injury that caused the soma to sensory or motor impairment of the affected UE; presence of receptive (sensory) or expressive (motor) aphasia; impaired vision; presence of visuospatial or unilateral spatial neglects; uncontrolled post-stroke seizures; or participation in another experimental rehabilitation project during the duration of treatment.
**Details of neurostimulation**	Patients received 20 min of TENS acupoints (100 Hz, 0.2 ms, square pulses, 2 to 3 times the sensory threshold).
**Efficacy outcomes**	The Fugl-Meyer Assessment of the upper extremity (FMA-UE)<br>The box and block test (BBT)<br>Quantitative electroencephalogram (QEEG)
**Safety outcomes**	No mention of it
**Conclusions**	TST only or combined with TENS acupoints can be considered an effective method for improving motor function of the affected UE in chronic stroke patients, both being equally effective. However, TST combined with TENS acupoints proved better in improving brain plasticity in chronic stroke patients.

**Trials**	**Jung et al. 2017**<br>(——)

**Inclusion criteria**	(1) Hemiparesis caused by stroke, (2) presentation with mild to moderate motor and/or sensory deficits at the upper extremity, (3) chronic stage at least six months after stroke, (4) Mini-Mental State Examination (MMSE)scores was above 26, (5) unimpaired visual and vestibular function, and (6) the ability to under-stand and follow the experiment protocol.
**Exclusion criteria**	Patients with preexisting neurological disorders, progressive disease, traumatic brain injury, cardiopulmonary complications or other concurrent medical conditions were excluded.
**Details of neurostimulation**	Electrical stimulation (two times the sensory threshold, 100 Hz, 200 us) was applied to muscle belly of triceps and wrist extensors using a two channel TENS machine.
**Efficacy outcomes**	IEMG, AROM, muscle strength and FMA-UE
**Safety outcomes**	No mention of it
**Conclusions**	TRT Combined with TENS can improve paretic muscle activity in upper limb paresis, highlighting the benefits of somatosensory stimulation from TENS. TEN Sallowed retention of training effects, and that further efforts to integrate TENS into motor rehabilitation may contribute to consolidation of the positive effects of treatments based on practice of motor tasks.

**Trials**	**Yurdakul et al. 2020 (NCT04113369)**

**Inclusion criteria**	(1) Thromboembolic stroke, (2) ≤ 6 months of stroke, (3) right hand dominancy, (4) left hemiplegia, and (5) Brunnstrom stage ≥ 3 of recovery for upper extremity and hand.
**Exclusion criteria**	(1) Myopathy, tendinopathy, or peripheral neuropathy of the upper extremities; (2) auditory, cognitive, or speech disorder that impairs communication; (3) a history of fracture or arthrodesis in the upper limb; (4) contracture or severe spasticity (Ashworth scale ≥ 2) in forearm muscles; and (5) severe cardiovascular disorders.
**Details of neurostimulation**	The patients in the study group received 30 min of electrical stimulation to their nonparetic forearm on the wrist flexors by using an intermittent maximum strength program (6 s of tetanic contraction (a high-frequency pulsed current with 85-pps frequency, 250-μs pulse width, monopolar pulse rhythm, 1.5-s current rise time, 4-s steady time, and 0.5-s fall time) and 10 s of relaxation (a low-frequency pulsed current with 4-pps frequency, 250-μs pulse width, monopolar pulse rhythm, 0.5-s current rise time, 9-s steady time, and 0.5-s fall time)) along with 5 min of warmup program before the stimulation (5-pps frequency, 200-μs pulse width, 1.5-s current rise time, and 1.5-s current fall time) and 5 min of cool-down program (3-pps frequency, 200-μs pulse width, 1.5-s current rise time, and 1.5-s current fall time) after the stimulation using a Genesy 600 electrostimulation device.<br>The TENS group patients received 30 min of conventional antalgic TENS (100-pps frequency, 50-μs pulse width, bipolar pulse rhythm, 1.5-s current rise time, and 1.5-s current fall time) as controls with the same device to their forearm.

**Efficacy outcomes**	The Fugl–Meyer motor function assessment for upper extremity (FMA-UE)<br>Functional independence measure (FIM)<br>Brunnstrom staging of recovery for hand,<br>Maximum and mean wrist flexion force,<br>Wrist extension force (extension max and extension mean) of paretic untrained limb
**Safety outcomes**	Before the treatment, three patients in the TENS group and one in the EMS group had grade 1 spasticity, and after the treatment, none of the patients demonstrated spasticity.
**Conclusions**	Although stroke patients in both EMS and TENS groups improved similarly in terms of clinical assessments, the maximum and mean wrist flexor strengths of the paretic limb increased more with adding EMS to the non-paretic homologous muscles. We demonstrated a beneficial effect of upper extremity, cross-education strength training via EMS on muscle strength inpatients with subacute stroke. We believe that cross-education should be considered for patients with sub-acute stroke with unilateral weakness as an adjunct to conventional treatment methods.

**Trials**	**Capone et al. 2017**<br>(——)

**Inclusion criteria**	(a) First-ever, ischemic or haemorrhagic stroke at least 1 year earlier; (b)hand function impairment; (c) and ability to give informed consent and comprehend instructions.
**Exclusion criteria**	(a) Previous surgical intervention on vagus nerve; (b) low hearth rate (<60 bpm); (c) cognitive impairment or any substantial decrease in alertness, language reception, or attention that might interfere with understanding instructions for motor testing; (d) apraxia; (e) excessive pain in any joint of the paretic extremity; (f) advanced liver, kidney, cardiac, or pulmonary disease; (g) history of significant alcohol or drug abuse; (h) depression or use of neuropsychotropic drugs such as antidepressants or benzodiazepines; (i) and pregnancy.
**Details of neurostimulation**	VNS was delivered as trains lasting 30 s and composed by 600 pulses (intratrain pulse frequency = 20 Hz; pulse duration = 0.3 ms) repeated every 5 min for 60 min.
**Efficacy outcomes**	Change in upper extremity Fugl–Meyer assessment (FMA) score
**Safety outcomes**	After intervention, there were no adverse events.
**Conclusions**	Our pilot study confirms that VNS is feasible and safe in stroke patients and can produce a slight clinical improvement in association to robotic rehabilitation. Compared to traditional, invasive stimulation, t VNS seems to be safer and more tolerable.

**Trials**	**Dawson et al. 2016 (NCT01669161)**

**Inclusion criteria**	1. History of unilateral supra tentorial ischemic stroke that occurred at least 6 months prior, 2. Age > 18 years and < 80 years, 3. Right or left sided weakness of upper extremity
**Exclusion criteria**	1. Hemorrhagic stroke, 2. Any deficits in language or attention that interferes with reasonable study participation, 3. Presence of significant apraxia, 4. Profound Sensory loss, 5. Active major neurological or psychiatric diagnosis that would likely interfere with study protocol including alcohol or drug abuse
**Details of neurostimulation**	A 500-ms burst of VNS was delivered to the VNS group during each movement (the rehabilitation-only group did not have a device implanted). Each simulation consisted of fifteen 0.8-mA, constant current, charge balanced pulses (100-μs pulse width, 30-Hz frequency).
**Efficacy outcomes**	The main efficacy assessment was change in FMA-UE score between the main baseline assessment and the first post-therapy visit. Secondary end points were changes in ARAT, Box and Block, 9-hole peg test, and grip strength. We also assessed whether participants improved by the minimum clinically significant difference in the FMA-UE and ARAT scores. This was defined as ≥6 points in the FMA-UE score and ≥5 points in the ARAT score.
**Safety outcomes**	There were 22 AEs in the 8 participants in the VNS group (11 of which were adverse device effects in 5 participants) when compared with 10 AEs in 3 participants in the rehabilitation-only group. Two participants had a serious AE in the VNS group, none of which were serious device effects. One participant had 4 serious AEs in the rehabilitation group. No AEs were related to the rehabilitation therapy itself.

**Conclusions**	VNS paired with rehabilitation therapy is feasible in adults with arm weakness ≥6 months after ischemic stroke. It also seems to be acceptably safe for further study.

**Trials**	**Kimberley et al. 2018 (NCT02243020)**

**Inclusion criteria**	1. History of ischemic stroke that occurred at least 4 months prior to enrollment, but not more than 24 months prior, 2. Age >30 years and <80 years, 3. Right or left sided weakness of upper extremity, 4. UEFM score within designated range, 5. At least 10 degrees of wrist extension, 10°of thumb abduction/extension, and at least 10°of extension in at least 2 additional digits
**Exclusion criteria**	1. History of hemorrhagic stroke, 2. Any deficits in language or cognitive functioning that hinders participation, for example, (aphasia) or unable to follow 2 step commands, 3. Significant sensory loss, 4. Presence of ongoing dysphagia or aspiration difficulties, 5. Active major neurological or psychiatric diagnosis that would likely interfere with study protocol including prior history of brain lesions (including dementia, etc.), and current alcohol abuse, drug abuse, or epilepsy. 6. Subject receiving any therapy (medication or otherwise) at study entry that would interfere with VNS (e.g. drugs that interfere with neurotransmitter mechanisms). Additionally, no psychoactive medications - including nicotine - may be used during the acute study. 7. Prior injury to vagus nerve - either bilateral or unilateral (e.g., injury during carotid endarterectomy), 8. Severe depression, 9. Not considered candidate for a device implant surgery (history of adverse reactions to anesthetics, poor surgical candidate in surgeon's opinion, etc.), 10. Any other implanted device such as a pacemaker or other neurostimulator; any other investigational device or drug, 11. Medical or mental instability (diagnosis of personality disorder, psychosis, or substance abuse), 12. Pregnant or plan on becoming pregnant or breastfeeding during

	the study period, 13. Currently require, or likely to require, diathermy during the study duration, 14. Any health problem requiring surveillance with MRI imaging, 15. Active rehabilitation within 4-weeks prior to therapy, 16. Botox injections or any other non-study active rehabilitation of the upper extremity 4-weeks prior to and during therapy, 17. Severe spasticity of the upper limb
**Details of neurostimulation**	Participants were randomized to either active VNS (0.8 mA) or control VNS (0.0 mA).
**Efficacy outcomes**	Change in Upper Extremity Fugl-Meyer (UEFM), Wolf Motor Function Test (WMFT; time and functional), Box and Block Test (BBT), Nine-Hole Peg Test, Stroke Impact Scale, and Motor Activity Log.
**Safety outcomes**	There were 3 serious adverse events related to implantation surgery, including 1 implantation wound infection requiring treatment with intravenous antibiotics but resolved; 1 case of shortness of breath and dysphagia, likely because of intubation, which recovered; and 1 case of hoarseness because of vocal cord palsy. There were no serious adverse events reported as associated with stimulation.
**Conclusions**	Rehabilitation paired with VNS is an acceptably safe and feasible intervention for the treatment of upper limb weakness after ischemic stroke. The study demonstrated sufficient safety, feasibility, and potential efficacy to support a larger pivotal trial.

**Trials**	**Dawson et al. 2021** (NCT03131960)

**Inclusion criteria**	1. History of unilateral supratentorial ischemic stroke that occurred at least 9 months but not more than ten 10 years prior to enrollment. 2. Age >22 years and <80 years. 3. FMA-UE score of 20 to 50 (inclusive of 20 and 50). 4. Ability to communicate, understand , and give appropriate consent. Subjects should be able to follow two-step commands. 5. Right- or left-sided weakness of upper extremity. 6. Active wrist flexion/extension; active abduction/extension of thumb and at least two additional digits.
**Exclusion criteria**	1. History of hemorrhagic stroke, 2. Presence of ongoing dysphagia or aspiration difficulties. 3. Subject receiving medication that may significantly interfere with the actions of VNS on neurotransmitter systems at study entry. A list of excluded medications will be provided to Investigators. 4. Prior injury to vagus nerve, either bilateral or unilateral (e.g., injury during carotid endarterectomy). 5. Severe or worse depression (Beck Depression Scale > 29) (Beck et al.., 1961), 6. Unfavorable candidacy for device implant surgery (e.g., history of adverse reactions to anesthetics, poor surgical candidate in surgeon's opinion, etc.), 7. Current use of any other stimulation device, such as a pacemaker or other neurostimulator; current use of any other investigational device or drug. 8. Medical or mental instability (diagnosis of personality disorder, psychosis, or substance abuse) that would prevent subject from meeting protocol timeline. 9. Pregnancy or plans to become pregnant or to breastfeed during the study period.<br>10. Current requirement, or likely future requirement, of diathermy during the study duration. 11. Active rehabilitation within 4 weeks prior to consent. 12. Botox injections or any

	other non-study active rehabilitation of the upper extremity within 4 weeks prior to therapy through the post-30 day visit (Visit 6). 13. Severe spasticity of the upper limb (Modified Ashworth ≥3) (Bohannon and Smith, 1987). 14. Significant sensory loss. Sensory loss will be measured using the Upper Extremity sensory section of the Fugl Meyer Assessment of Physical Performance. The assessment addresses light touch (2 items) and proprioception (4 items).The highest points attained is 12; subjects with scores less than 6 will be excluded from the study.
**Details of neurostimulation**	The VNS group received 0·8 mA, 100 µs, 30 Hz stimulation pulses, lasting 0·5 s.<br>The control group received 0 mA pulses.
**Efficacy outcomes**	The primary outcome was change in FMA-UE score from baseline to the first day following completion of inclinic therapy.The secondary outcomes measures were clinically meaningful response on FMA-UE score at day 90; change in day 90 WMFT-Functional score relative to pretreatment (baseline); and change in day 90 FMA-UE score relative to baseline.Tertiary outcome measures were the MAL score, SIS score, SS-QOL score, and EQ-5D score.
**Safety outcomes**	43 (81%) of 53 patients in the VNS group and 42 (76%) of 55 patients in the control group had at least one adverse event (total of 163 events vs 171 events, appendix p 6). There were no deaths. There were three severe adverse events in two (4%) participants in the VNS group (one each of urinary tract infection, hyponatraemia, and insomnia) and two severe adverse events in two (4%) participants in the control group (one headache and one syncope). None of the severe events were

	reported as related to the trial device. 21 (40%) participants in the VNS group and 24 (55%) in the control group reported an adverse event assessed as either possibly, probably, or definitely related to device implantation. These events were mostly due to postoperative pain. 13 (25%) of 53 participants in the VNS group and nine (16%) of 55 participants in the control group reported an adverse event assessed as either possibly, probably, or definitely related to device use. The number of events, the number of participants reporting at least one event, and the number of severe events were similar in both groups (appendix p 7). There were no unexpected adverse events or serious adverse events reported associated with the device. There was one case of vocal cord palsy following device implantation in a control participant, which resolved after 5 weeks.
**Conclusions**	Participants with arm impairment, an average of 3 years after ischaemic stroke, who received rehabilitation showed clinically meaningful improvements in impairment and function that were 2–3 times greater with vagus nerve stimulation compared with sham stimulation. Improvements with paired vagus nerve stimulation therapy were also reflected in quality-of-life measures. Vagus nerve stimulation combined with rehabilitation is a novel strategy to help people achieve improvement in arm and hand function after stroke.

**Trials**	**Wu et al. 2020 (CTR1800019635)**

**Inclusion criteria**	(1) First time ischemia stroke; (2) in the acute or sub-acute phase of stroke (between 0.5 and 3 months post onset); (3) single upper limb motor function impairment; and (4) ability to follow instructions, with no obvious cognitive impairment.
**Exclusion criteria**	(1) Hemorrhagic stroke, which lead to the heterogeneity of lesion etiology; (2) advanced cardiac, pulmonary, liver, kidney dysfunction or blood system diseases; (3) malignant tumors or infectious diseases; (4) other neurologic or musculoskeletal diseases that could interfere with the assessments of this study; (5) low heart rate (<60 bpm); (6) previous surgical intervention on the vagus nerve; and (7) Botox injections or any other non-study active rehabilitation of the upper extremity 12 weeks prior to or during therapy.
**Details of neurostimulation**	The parameters were selected as follows: 600 pulses (intratrain pulse frequency = 20 Hz; pulse duration = 0:3 ms), lasting 30 seconds each time, stimulating once every 5 minutes.
**Efficacy outcomes**	The upper limb Fugl-Meyer assessment (FMA-U), the Wolf motor function test (WMFT), the Functional Independence Measurement (FIM), and Brunnstrom stage.
**Safety outcomes**	Only one adverse event was noted during the whole procedure. One patient in the taVNS group developed skin redness at the point of contact of the auricle skin electrodes after the third treatment, which returned to normal 6 hours later. There were no unpleasant sensations or other discomforts.
**Conclusions**	TaVNS appeared to be beneficial to the recovery of upper limb motor function in sub-acute ischemia stroke patients and without obvious adverse effects. Future studies are needed to confirm the optimum timing and ideal stimulation parameters and unveil the mechanisms of action of this innovative approach.

**Trials**	**McCabe et al. 2015 (NCT01725659)**

**Inclusion criteria**	(1) Persistent (>1y), upper-extremity impairment; (2) at least a trace muscle contraction in the wrist extensors; (3) single unilateral stroke; (4) mobility and function sufficient for independent performance of activities (eg, toileting, eating lunch during the treatment days); (5)stable medical condition; (6) no other prior neurologic condition; (7) ability to follow 2-step commands.
**Exclusion criteria**	(——)
**Details of neurostimulation**	The stimulation parameters were as follows: 300-millisecond pulse width, 40Hz, and amplitude varied according to subject tolerance.
**Efficacy outcomes**	Arm Motor Ability Test (AMAT)<br>FM coordination scale
**Safety outcomes**	No adverse events occurred as a result of participation in the study.
**Conclusions**	Severely impaired stroke survivors with persistent (>1y) upper-extremity dysfunction can make clinically and statistically significant gains in coordination and functional task performance in response to robotics plus ML, FES plus ML, and ML alone in an intensive and long-duration intervention; no group differences were found.

**Trials**	**Shimodozono et al. 2014**<br>(——)

**Inclusion criteria**	(1) A new, single CT-confirmed stroke of 3–13 weeks duration; (2) severe arm impairment, FMA20; (3) an ability to follow simple directions.
**Exclusion criteria**	(1) Contraindication for ES, such as a pacemaker implant; (2) arm contractures or pain; (3) preexisting arm impairment; (4) cerebellar lesion; (5) unstable medical status; (6) perceptual, apraxic or cognitive deficits that would prevent adequate study participation; (7) inability to provide informed consent.
**Details of neurostimulation**	The stimulation pulse was asymmetrical biphasic waveform, with a pulse width of 250 ms and frequency of 20 Hz.
**Efficacy outcomes**	Fugl-Meyer Assessment Scale (FMA)<br>active range of motion (ROMs)<br>the modified Ashworth scale (MAS)
**Safety outcomes**	No adverse treatment effects, such as burns, skin allergic responses or muscle fatigue, were noted.
**Conclusions**	RFE under NMES is feasible in clinical settings and may be more effective than conventional rehabilitation in lessening arm impairment after sub-acute stroke.

**Trials**	**Shindo et al. 2011 (UMIN000001971)**

**Inclusion criteria**	(a) First time unilateral supratentorial stroke, (b) time from stroke onset within 60 days at hospitalization, (c) age 20 to 80 years, (d) muscle activities in the affected EDC detectable with surface electrodes, (e) patients who could not fully extend their paretic fingers and could not extend their paretic fingers individually, (f) passive range of motion greater than 0° for the affected wrist extension and −10° for metacarpopharyngeal joint extension, (g) Mini-Mental State Examination score >23.
**Exclusion criteria**	(a) Severe cognitive deficits such as unilateral spatial neglect or aphasia that preclude HANDS therapy, (b) severe proprioceptive deficits or pain in the paretic upper extremity, (c) pacemaker or other implanted stimulator, (d) seizure history, (e) other serious medical conditions.
**Details of neurostimulation**	The IVES is a portable, noninvasive (surface), closed loop EMG-controlled, single channel neuromuscular electrical stimulator. A pair of electrodes for EMG detection and stimulation (30 × 12 mm) placed 5 mm apart, and one electrode (30 × 30 mm) for reference and stimulation were placed on the affected EDC muscle. The stimulator continually adjusts its stimulation intensity in proportion to the amplitude of the voluntary EMG activities. The maximum stimulus intensity was set at a level tolerable by the patients and at which extension of four fingers besides the thumb to 0° was achieved during the voluntary finger extension attempt. The patients also wore a wrist splint and carried a portable IVES in a waist-bag for 8 hours. Their intact upper extremity was not restrained. The control group wore the same wrist splint as the HANDS group for 8 hours a day in addition to the standard rehabilitation program described below. Both groups were instructed to use

	their affected hand as much as possible while wearing the HANDS system or the splint in their activities of daily living.
**Efficacy outcomes**	Fugl-Meyer Assessment (FMA)<br>Action Research Arm Test (ARAT)<br>Motor Activity Log (MAL)<br>Modified Ashworth Scale (MAS)
**Safety outcomes**	The interventions were not associated with any obvious adverse effects such as shoulder pain.
**Conclusions**	HANDS therapy in addition to conventional therapy may improve hand function inpatients with moderate to severe hand impairment during early rehabilitation.

**Trials**	**Chan et al. 2008**
	(——)

**Inclusion criteria**	(1) No skin allergy to electric stimulation/electrodes, (2) a score of "0" in the finger mass extension subitem of the Fugl–Meyer Assessment (FMA), (3) able to follow simple commands, (4) 6 weeks after onset of stroke, (5) first episode of stroke, (6) Glasgow Coma Scale = 15/15.
**Exclusion criteria**	(1) Severe dysphasia (either expressive or comprehensive) with inadequate communication, (2) any additional medical or psychological condition affecting their ability to comply with the study protocol, (3) history of other neurological diseases and psychiatric disorder, including alcoholism and substance abuse.
**Details of neurostimulation**	FES: The stimulation frequency was 40 Hz, and the pulse width was 200 μs.<br>Control: the intensity of the electric stimulation did not trigger any muscle movement (they only had a slight sensation of electric stimulation).
**Efficacy outcomes**	Functional Test for the Hemiplegic Upper Extremity (FTHUE)<br>Fugl-Meyer assessment (FMA)<br>Functional Independence Measure (FIM)<br>Modified Ashworth Scale values (MAS)
**Safety outcomes**	(——)
**Conclusions**	FES with bilateral upper limb training could improve motor functions in chronic stroke patients. There could be a benefit of having daily activity tasks training with a synchronized FES system. Our research supports the development of further studies using larger samples with long-term follow-up to reach a more confident conclusion about this method of rehabilitation.

**Trials**	**Knutson et al. 2011**
	(——)

**Inclusion criteria**	(1) ≤6 months from first hemorrhagic or non-hemorrhagic stroke, (2) finger extensor strength ≤4/5 (Medical Research Council scale), (3) sufficient active shoulder and elbow movement to volitionally position the hand in the workspace, (4) functional hand opening in response to electrical stimulation, (5) ability to follow 3-stage commands, (6) available caregiver to assist with the stimulator if needed.
**Exclusion criteria**	(1) Had intramuscular botulinum toxin injections in any upper limb muscle within 3 (2) months preceding study entry, (3) edema or absent sensation of the affected forearm or hand, (4) uncompensated hemineglect, (5) severe shoulder or hand pain, (6) evere depression, (7) insufficient passive range of motion of the wrist or fingers to allow a functional degree of hand opening.
**Details of neurostimulation**	The stimulators used in this study delivered biphasic rectangular current pulses; the pulse frequency was set at 35 Hz, and the pulse amplitude was set at 40 mA for all but 1 participant who required 60 mA.
**Efficacy outcomes**	Maximum voluntary finger extension angle ,<br>finger movement control (tracking test),<br>the upper limb portion of the Fugl-Meyer motor assessment<br>Box and Blocks Test (BBT)<br>Functional Ability component of the Arm Motor Abilities Test (AMAT)
**Safety outcomes**	（——）
**Conclusions**	In spite of major sources of potential bias, the finding that CCFES was superior to cyclic NMES suggests that we need to optimize the CCFES treatment and confirm the preliminary findings here in a properly powered trial.

Trials	Knutson et al. 2016 (NCT00891319)

**Inclusion criteria**	(1) >6 months from hemorrhagic or ischemic stroke; (2) unilateral finger extensor paresis indicated by a score of ≤4 of 5 on the manual muscle test (Medical Research Council scale); (3) sufficient active shoulder and elbow movement to volitionally position the paretic hand in the workspace for table-top task practice; (4) hand opening elicited by electrical stimulation of the paretic finger and thumb extensors without pain; (5) no intramuscular botulinum toxin injection of any upper limb muscle in the previous 3 months; (6) not receiving concomitant occupational therapy.
**Exclusion criteria**	(1) Insufficient passive range of motion of the wrist, fingers, or thumb to allow functional hand opening (2) Severe shoulder or hand pain (unable to position hand in workspace without pain) (3) Uncontrolled seizure disorder (4) Insensate to touch on forearm and/or hand (5) Uncompensated hemineglect (6) History of cardiac arrhythmias with hemodynamic instability (7) Cardiac pacemaker or other implanted electronic system (8) Diagnosis of Parkinson's Disease, spinal cord injury, traumatic brain injury, or multiple sclerosis (9) Intramuscular botulinum toxin injection of any upper limb musclein the previous 3 months (10) Pregnant
**Details of neurostimulation**	For each participant, surface electrodes were positioned over the forearm finger and thumb extensors to produce hand opening. Up to 3 electrodes were used, each delivering pulses of electric current with a pulse frequency of 35 Hz and amplitude of 40 mA. The strength of muscle contraction was modulated with pulse duration (0–250 µs).
**Efficacy outcomes**	Box and Block Test (BBT)<br>Arm Motor Abilities Test (AMAT)

	upper extremity Fugl–Meyer assessment (UEFM)
**Safety outcomes**	There were no serious, unexpected, study-related adverse events. However, 1participant frequently had headaches after FTP sessions, 3 participants had skin irritation from the electrodes, and 1 participant found the stimulation transiently uncomfortable.
**Conclusions**	In patients with chronic (>6 months) severe to moderate hand impairment after stroke, 12 weeks of CCFES therapy improved manual dexterity at 6 months post treatment more than an equivalent dose of cNMES. The advantage of CCFES over cNMES was greatest in participants who were <2 years post stroke and who had moderate rather than severe hand impairment.

**Trials**	**Knutson et al. 2020 (NCT01688856)**

**Inclusion criteria**	(1) Age ≥ 21 and ≤ 80, (2) ≤ 2 years of first clinical hemorrhagic or nonhemorrhagic stroke, (3) Skin intact on hemiparetic arm and hand, (4) Able to follow 3-stage commands, (5) Able to recall 2 of 3 items after 30 minutes, (6) Medically stable, (7) Finger extensor paresis indicated by a score of ≤ 4 out of 5 on the manual muscle test (Medical Research Council scale), (8) Adequate movement of shoulder and elbow to position the paretic hand in the workspace for table-top task practice, (9) Caregiver available to assist with device daily - OR - able to independently don elbow cuff on unaffected arm, (10) Full volitional elbow extension/flexion and hand opening/closing of unaffected limb, (11) Upper extremity hand section of Upper Extremity Fugl-Meyer (UEFM)≥ 1 AND ≤ 11/14, (12) Unable to simultaneously fully extend the elbow and fully open the hand toward tabletop object with arm unsupported (i.e. cannot voluntarily achieve the maximum passive range of motion (PROM) available), (13) Functional PROM (minimal resistance) at shoulder, elbow, wrist, and hand simultaneously on affected side (i.e., there exists enough PROM to reach and acquire table-top objects), (14) Able to hear and respond to stimulator cues, (15) While relaxed, surface NMES of finger extensors and thumb extensors and/or abductors produces a functional degree of hand opening without pain, (16) While relaxed with the forearm supported with a mobile arm support, surface NMES of elbow extensors (triceps) produces functional elbow extension without pain. (17) Patient must be able to sit unassisted in an armless straight-back chair for the duration of the screening portion of the eligibility assessment.

**Exclusion criteria**	(1) Co-existing neurological condition other than prior stroke involving the hemiparetic upper limb (e.g., peripheral nerve injury, Parkinson's Disease, Spinal Cord Injury, Traumatic Brain Injury, Multiple Sclerosis). (2) Severely impaired cognition and communication. (3) Uncontrolled seizure disorder, (4) History of cardiac arrhythmias with hemodynamic instability, (5) Cardiac pacemaker or other implanted electronic device, (6) Pregnant, (7) IM Botox injections in any UE muscle in the last 3 months, (8) Insensate arm, forearm, or hand, (9) Uncompensated hemi-neglect (extinguishing to double simultaneous stimulation), (10) Severe shoulder or hand pain, (11) Severe depression on Beck Depression Inventory (BDI) (score>=13 on BDI-fast screen)
**Details of neurostimulation**	A custom-built stimulator generated biphasic rectangular current pulses. A pulse frequency of 35 Hz was used. The default pulse amplitude was 40 mA; 60 mA was used in cases where 40 mA was inadequate to produce hand opening (3 participants) or elbow extension (6 participants). For each electrode, a maximum pulse width up to 255 μsec was determined as that which produced maximum finger or thumb opening, or elbow extension (if applicable), without discomfort.
**Efficacy outcomes**	Box and Blocks Test (BBT)<br>reachable workspace (RW)<br>UEFM score<br>Stroke Upper Limb Capacity Scale (SULCS)<br>Arm Motor Abilities Test (AMAT)<br>Motor Activity Log (MAL)
**Safety outcomes**	There were no serious, unexpected, study-related adverse events. Non-serious study-related or possibly related adverse

	events included shoulder pain during or after the RW assessment in nine participants (in three of these the shoulder pain may have been exacerbated by recent falls and in one by rheumatoid arthritis); numbness and tingling in both hands while at rest in one A + H CCFES participant; abdominal pain during RW assessment in two participants, each of whom had a history of abdominal aneurism or hernia; stimulation was transiently uncomfortable in one participant.
**Conclusions**	Adding contralaterally controlled elbow extension to hand CCFES does not improve upon gains in hand dexterity, but it further reduces upper limb impairment and improves RW measured in the laboratory. However, these additional benefits may not be large enough to be perceived by stroke survivors when they are performing activities of daily living at home.

**Trials**	**Shen et al. 2015**
	（——）

**Inclusion criteria**	(1) ≤3 months from first hemorrhagic or non-hemorrhagic stroke; (2) 20 to 80 years old; (3) unilateral onset; (4) in a stable condition; (5) manual muscle grade of 3 or less for finger extensors; (6) upper extremity Brunnstrom stage: II-IV; (7) functional hand opening without pain produced by stimulation through surface electrodes; (8) able to operate the stimulator properly.
**Exclusion criteria**	Had intramuscular botulinum toxin injections in any upper extremity muscle preceding study entry or at any time during the study, loss of sensation in the affected forearm or hand, uncompensated hemi-neglect or hemianopsia, insufficient passive range of motion of the wrist or fingers to allow a functional degree of hand opening or their motion dysfunction was caused by other reasons before onset.
**Details of neurostimulation**	The stimulus current was biphasic wave with a pulse duration of 200μs and a frequency of 60Hz. The electrical stimulation lasted for 20min per session, 5 sessions per week for 3 weeks.
**Efficacy outcomes**	Upper extremity part of the Fugl-Meyer assessment (FMA)<br>Upper limb motricity index (MI)<br>Functional test for the hemiplegic upper extremity (FTHUE-HK)<br>Active range of motion (AROM)
**Safety outcomes**	（——）
**Conclusions**	In the current study, CCFES shows better motor function recovery for the paralyzed limbs than the standard NMES method in post-stroke patients.

**Trials**	**Zheng et al. 2019**
	（——）

**Inclusion criteria**	(i) diagnosed with stroke using computed tomography (CT) or magnetic resonance imaging (MRI); (ii) stable vital signs 48 h post-stroke; (iii) single-side injury; (iv) age 20–80 years; (v) within 15 days post-stroke; (vi) Brunnstrom recovery stage of III or less; (vii) score of Fugl Meyer assessment (FMA) for upper extremity ≤22; and (viii) no active WD detected.
**Exclusion criteria**	(i) progressive stroke with non-stable condition; (ii) stroke-like symptoms due to subdural haematoma, tumour, encephalitis or trauma; (iii) unable to follow treatment instructions due to severe cognitive and communication deficiency; (iv) implanted with a pacemaker; and (v) no informed consent.
**Details of neurostimulation**	The stimulators used in this study delivered biphasic rectangular current pulses; the pulse frequency was set at 35 Hz, and the pulse amplitude was set at 40 mA.<br>The intensity of the electrical stimulation to WD of the paretic side was determined by the strength of contralateral forearm extensor muscles contraction. Subjects were asked to voluntarily extend their unaffected wrist to 10% of ROM or less and maintain that position without moving.
**Efficacy outcomes**	active ROM for WD measured with a goniometer<br>Manual Muscle Testing<br>Fugl-Meyer assessment (FMA)<br>Jebsen Hand Function Test (JHFT)<br>Modified Barthel Index (mBI)
**Safety outcomes**	Nine out of the total of 50 dropped out due to secondary cerebral haemorrhage (1 in the CCFES group and 2 in the NMES group), compressive lumbar vertebrae bone fracture after falling off a bed (1 in the CCFES group), secondary cerebral infarction (1 in

	the CCFES group and 2 in the NMES group), serious pulmonary infection (1 in the CCFES group and 1 in the NMES group).
**Conclusions**	CCFES signifcantly shortened the time for regaining wrist dorsiflexion, and improved the upper extremity function and general health of patients with early-phase stroke. CCFES therefore has potential as a clinical intervention.

**Trials**	**Zhou et al. 2017**

	(——)
**Inclusion criteria**	(i) first haemorrhagic or non-haemorrhagic stroke 2 weeks to 6 months prior to the study; (ii) lesions located in the territory of the middle cerebral artery (MCA), including the subcortical regions of the corona radiata, internal capsule, and the basal ganglia (mainly the putamen and the globus pallidus, as well as the overlying cortex, diagnosed using either computed tomography (CT) or magnetic resonance imaging (MRI); (iii) a Brunnstrom score between stages I and IV for the upper extremities; (iv) ability to understand and follow simple verbal instructions; (v) visible hand opening in response to NMES; and (vi) ability to sit unsupported for 40 min.
**Exclusion criteria**	(i) severe heart, liver, kidney or infectious diseases; (ii) lesions in the cerebellum or brainstem; (iii) a history of other neurological diseases or psychiatric disorders; (iv) shoulder-hand syndrome; (v) uncompensated hemineglect; (vi) intramuscular botulinum toxin injections in any upper-extremity muscles within 3 months; (vii) severe cognitive disorders (Mini-Mental State Examination score ≤24) (16); or (viii) severe depression (Hamilton Rating Scale for Depression >24)
**Details of neurostimulation**	CCFES: The modulation range of pulse duration and frequency are 150–750 μs and 23–60 Hz, respectively, the maximum/minimum compliance voltage is ±34 V, and the maximum output peak current is up to 30 mA. Before each training session, the current amplitude was first determined and set to elicit maximum wrist extension or hand opening without pain using a stimulation train with a pulse duration of 750 μs and a frequency of 60 Hz.

	Cyclic NMES: The stimulator delivered biphasic rectangular current pulses at a frequency of 50 Hz with a pulse duration of 500 μs. The stimulation current was set to elicit maximum wrist extension and hand opening in a range up to 40 mA. The time for ramping up and down was 1 s each, and a duty cycle of 5 s on and 5 s off was applied.
**Efficacy outcomes**	Brunnstrom's stages<br>upper extremity components of the UE-FMA<br>motor status scale (MSS)<br>sEMG ratio of the ECU/EDC<br>active range of wrist/finger extension
**Safety outcomes**	（——）
**Conclusions**	In the present group of subacute stroke patients, the results favour EMGB over cyclic NMES for augmenting the recovery of volitional wrist and finger motion.

Trials	Alisar et al. 2019 (NCT03839316)
**Inclusion criteria**	(1) Age 18-75 years (2) first time stroke sufferer (3) stroke of vascular aetiology as determined by computerised tomography or magnetic resonance imaging (4) at least 3 months since stroke onset (5) mini mental state examination score ≥23 (6) stable medical condition.
**Exclusion criteria**	(1) Presence of sensory aphasia/neglect/significant hearing or visual loss/significant spasticity (modified Ashworth scale grade 3-4) (2) history of epilepsy/brain tumour/cranial surgery (3) presence of a pacemaker/intracranial metallic implant.
**Efficacy outcomes**	Fugl-Meyer Upper Extremity (FMUE)<br>Functional Independence Measure (FIM)<br>Brunnstrom Stages of Stroke Recovery (BSSR)
**Details of neurostimulation**	Dual-tDCS: Bihemispheric tDCS was applied by placing the anode on the C3 motor point of the ipsilesional hemisphere and the cathode on the C4 motor point of the contralesional hemisphere. Anodal tDCS stimulates ipsilesional motor cortex excitability whereas cathodal tDCS inhibits contralesional motor cortex excitability. The stimulator was then activated and current increased to 2 mA and continued for 30 minutes during the OT session.<br>Control: The electrodes were placed using the same method in the sham group and the stimulator switched on and current increased until the patient felt the typical "tingling" sensation on their scalp for a duration of 30 seconds.
**Safety outcomes**	None of the patients reported any side effects of treatment.
**Conclusions**	Upper extremity motor function in hemiplegic stroke patients improves when bihemispheric tDCS is used alongside

	conventional PT and OT. The improvement in functionality is greater in chronic stroke patients.

Trials	Allman et al. 2016 (NCT01414582)
Inclusion criteria	(1) Participant is willing and able to give informed consent for participation in the study，(2) Male or Female, aged 18 - 80 years，(3) Patients should be at least six months post first symptomatic stroke affecting motor function of the hand.
Exclusion criteria	(1) Anyone who does not have adequate understanding of verbal and written information in English, sufficient to complete any of the safety screening forms. (2) Anyone who has a previous history of epilepsy, febrile convulsions as a child or recurrent fainting fits. Likewise, anyone who has a significant family history of epilepsy would be excluded as all these conditions carry a theoretical risk of increasing susceptibility to seizures. (3) Any person who has a history of drug abuse or a previous history of a neurological or psychiatric illness, or has a history of neurosurgical procedure is excluded as they may be at increased risk of epilepsy and data collected may be influenced by their condition. (4) Patients on some prescription medications such as anti-depressants may be excluded as they may be at an increased risk of seizures. (5) Anyone who is currently taking or who has taken anti-malarial treatment in the last 72 hours. (6) Pregnant women are excluded as a precaution as there is no data on the effect on maternal cranial stimulation on the fetus. (7) Any metallic implant in the neck, head, or eye and anyone with any implanted electrical devices would be excluded as there is a risk of heating with both TMS and TDCS stimulation. (8) Anyone with any metal implants or implantable device would be excluded as indicated by the MRI safety screening form. People who suffer with claustrophobia as they are unable to tolerate the

	scanner. (9) Patients who have had more than one stroke. Patients who have had a stroke will also be excluded if they have limited communication in the form of aphasia or a history of dementia.
**Details of neurostimulation**	Anodal-tDCS: The electrodes were connected to a DC stimulator (Eldith GmbH), which was controlled by the treating researcher. For anodal stimulation, the current was ramped up over 10s, held at a constant 1 mA for 20 min, and then ramped down over 10s.<br>Control: For sham stimulation, the current was ramped up over 10s and then immediately switched off.
**Efficacy outcomes**	Upper ExtremityFugl-Meyer Assessment (UEFM)<br>Action Research Arm Test (ARAT)<br>Wolf Motor Function Test (WMFT)
**Safety outcomes**	(——)
**Conclusions**	We report long-term improvements in upper limb ability in patients receiving repeated sessions of anodal tDCS to the ipsilesional motor cortex compared to the sham-treated group when tDCS was paired with motor training. We also found that these clinical improvements were associated with increased activation of ipsilesional motor cortical areas.

Trials	Ang et al. 2015 (NCT01897025)
**Inclusion criteria**	(1) Aged 21 to 70 years (2) had their first-ever subcortical stroke at least 9 months before recruitment, (3) with moderate to severe impairment of upper extremity function (subscore of the Fugl-Meyer Motor Assessment [FMMA], 11-45)
**Exclusion criteria**	(1) A history of seizures, (2) major depression, (3) implants that may be triggered, moved, or heated by electrical current (eg, intracranial shunts, pacemakers, metalcranial implants).
**Details of neurostimulation**	Dual-tDCS: Direct current was applied for 20 minutes using a saline-soaked pair of surface sponge electrodes from a battery-operated constant current stimulatorc at an intensity of 1mA with the anode placed over the M1 motor cortex of the ipsilesional hemisphere and the cathode placed over the contralesional M1.<br>Control: For the sham intervention, the current was applied only for the fifirst 30 seconds of the 20 minutes to give the sensation of the stimulation.
**Efficacy outcomes**	Upper extremity Fugl-Meyer Motor Assessment（FMMA）
**Safety outcomes**	(——)
**Conclusions**	The results suggest a role for tDCS in facilitating MI in stroke. The facilitation of MI may translate to enhanced sensorimotor integration and the efficacy of MI-BCI as a tool for motor recovery after stroke. It may also avail MI-BCI to stroke patients who initially fail the screening test for the ability to operate MI-BCI.

Trials	Beaulieu et al. 2019<br>(——)
**Inclusion criteria**	(i) Aged 18 years old and up; (ii) supratentorial stroke >6 months (i.e. chronic phase) prior to study entry; (iii) level of sensorimotor recovery at the affected upper limb ≥35/66 on the Fugl-Meyer Stroke Assessment [FMA] scale.
**Exclusion criteria**	(i) Important resistance to passive stretching in the affected shoulder extensors, elbows flexors, wrist flexors or finger flexors; (ii) major somatosensory deficit; (iii) unilateral spatial neglect; (iv) pain intensity at the affected upper limb≥4/10 on the Visual Analog Pain Scale; (v) apraxia; (vi) neurological disorder other than a stroke; (vii) concomitant orthopaedic problems at the affected upper limb; (viii) attention or comprehension deficits;(ix) any contraindication to transcranial magnetic stimulation and/or tDCS.
**Details of neurostimulation**	Dual-tDCS: Saline-soaked 5X7cm electrodes were used in a bilateral tDCS (Soterix Medical, USA) montage (Marquez et al., 2015a), with the active anode electrode placed over ipsilesional M1 and the cathode electrode placed symmetrically to the anode electrode, over the contralesional M1. The tDCS was applied for the first 20 minutes of each of the 12 PRT sessions at an intensity of 2 mA (with progressive ramp up/down).<br>Control: For the sham-tDCS group, the tDCS was applied for the first 30 seconds only, to ensure an appropriate blinding of subjects.
**Efficacy outcomes**	Fugl-meyer assessment (FMA)<br>Wolf Motor Function Test (WMFT)<br>Box and Block Test (BBT)<br>Grip strength

	Motor Activity Log (MAL)<br>Modified Ashworth Scale (MAS)
**Safety outcomes**	For both groups, the most reported tDCS-related symptom was skin tingling. The tingling intensity was always rated 'mild' and was noted by the participants as 'possibly' to 'probably' related to tDCS (median = 'possibly')Only one participant in the real-tDCS group reported the perception of mild skin burning during the 4th and the 8th sessions, which was 'possibly' related to tDCS according to the subject. No other symptom was reported.
**Conclusions**	TDCS did not result in superior efficacy than sham tDCS, as both groups showed similar sensorimotor improvements of the affected upper limb post training.

Trials	Bornheim et al. 2019 (——)
**Inclusion criteria**	(1) Aged between 18 and 80 years old, (2) presenting their first ever symptomatic ischemic stroke confirmed by CT or MRI
**Exclusion criteria**	(1) Inability to sign or understand the consent form, (2) one "yes" in the high and relatively high risk sections of the TSST (such as implants in their body that may be triggered or heated by electrical current, CNS-active medication, nicotine, alcohol or other substance use), (3) hemineglect
**Details of neurostimulation**	Anodal-tDCS: The electrodes, both 25 cm2 , were placed on their head with the anode placed over the primary motor cortex of the lesioned side and the cathode over the contralesional eye (C3/Fp2 or C4/Fp1) (this model is the most frequently used in stroke research. The electrodes were attached using a neoprene EEG cap, to deliver either a continuous current or no current and at a rate of 5 times per week for 4 weeks. tDCS lasted 20 min at 1 mA, with a 15 s ramp up and ramp down.<br>Control: Sham tDCS consisted of a 15 s ramp up followed by a 15 s ramp down of the current.
**Efficacy outcomes**	Wolf Motor Function Test (WMFT)<br>Semmes Weinstein Monofilament Test (SWMT)<br>Upper Extremity section (UEFM)<br>Lower Extremity section (LEFM)<br>Somatosensory section of the Fugl Meyer Test<br>Tardieu Spasticity Scale<br>Stroke Impact Scale (SIS)<br>Hospital Anxiety and Depression Scale (HADS)<br>Barthel Index

**Safety outcomes**	All patients (n=50) tolerated the treatment program, the side effects being similar between sham and the treatment group.
**Conclusions**	tDCS seems to be an effective adjuvant to conventional rehabilitation techniques. If applied in the acute stages of stroke, functional recovery is not only accelerated, but improved, and results are maintained up to one-year post stroke.

Trials	Chen et al. 2021 (NCT04369235)
**Inclusion criteria**	(1) Aged≥20 years; (2) unilateral cerebral stroke with hemiplegia and Brunnstrom stage IV or V; (3) adequate understanding of verbal/written information and physically able to complete the motor learning of functional tasks with the affected hand.
**Exclusion criteria**	(1) Lower motor neuron impairment, (2) unstable autonomic nervous system, (3) extremely sensitive to electrical stimulation and could not tolerate it, (4) contractures in the upper extremity or limitations of joint motion, (5) severe spasticity, (6) myositis ossifcans, (7) a history of arrhythmia, (8) a medical electronic device implant, such as a pacemaker, (9) decubitus or scalp wounds, (10) metal head or neck implants, (11) severe cognitive dysfunction or active psychiatric diseases, such as schizophrenia or dissociative identity disorder, (12) a history of seizures or organic brain disease, (13) severe traumatic brain injury, (14) drug or alcohol abuse, (15) a malignant tumor or an autoimmune rheumatic disease, such as systemic lupus erythematosus, rheumatoid arthritis, or ankylosing spondylitis.
**Details of neurostimulation**	iTBS + Anodal-tDCS: Participants in the real NIBS group received a current intensity of 1mA (DC) combined 1.5mA (iTBS) for 20 min, and the anode electrode was placed over the primary motor cortex (M1) scalp location (C3 or C4 according to the EEG 10/20 system) of the afected hemisphere, and the cathode was placed on the contralateral back of the shoulder region. For each stimulation, current intensity was ramped up to 1 mA over 30s at the beginning, applied for 20 min and then ramped down to 0mA over 30s at the end of stimulation.

	Control: For the sham NIBS group, participants received an intervention as the active tDCS+iTBS group except that the stimulation intensity current was ramped up to 1mA over 30s and then the current was ramped down to 0mA over 30s throughout the entire process while the electrode sponges were kept on the participants' scalp for 20 min to mimic real stimulation.
**Efficacy outcomes**	Fugl-meyer assessment- upper extremity (FMA-UE)<br>Jebsen-taylor hand function test (JTT)<br>Finger- to- nose test (FNT)<br>Statistical analysis
**Safety outcomes**	No patient in either group experienced any adverse events during or after the tDCS+iTBS treatment.
**Conclusions**	Patients who underwent the combined ipsilesional tDCS-iTBS stimulation with conventional rehabilitation exhibited greater impacts than did patients who underwent sham stimulation-conventional rehabilitation instatistically significant clinical responses of the total JTT time and FNT after the stroke. Preliminary results of upper limb functional recovery suggest that tDCS-iTBS combined with a conventional rehabilitation intervention may be a promising strategy to enhance therapeutic benefits in future clinical settings.

Trials	Edwards et al. 2019 (NCT03562663)
**Inclusion criteria**	a) A first unilateral ischemic lesion; b) cognitive function sufficient to understand the experiments and follow instructions; c) Motor Power score 1-4/5 (neither hemiplegicnor fully recovered motor function in the muscles of the shoulder and elbow and wrist).
**Exclusion criteria**	Potential study candidates identified were ineligible to enroll if they had participated in upper limb robotic training within the prior six months.
**Details of neurostimulation**	Anodal-tDCS: Participants received stimulation for 20 minutes while seated (immediately prior to robotic motor training), with the anode centered 5 cm lateral to the vertex, and the cathode on the contralateral supraorbital area. Participants in the Robot-Sham group had a comparable set-up to Robot-tDCS, with the stimulation comprising a 30 sec current ramp to 2 mA, then 30 sec ramp down to 0 mA, repeated after 20 mins.<br>Control: Participants in the Robot-Sham group had a comparable set-up to Robot-tDCS, with the stimulation comprising a 30 sec current ramp to 2 mA, then 30 sec ramp down to 0 mA, repeated after 20 mins.
**Efficacy outcomes**	Fugl-Meyer (FM)<br>Medical Research Council motor power score(MRC)<br>Wolf Motor Function Test<br>Barthel Index (BI)<br>Stroke Impact Scale (SIS)
**Safety outcomes**	The Robot Sham and Robot tDCS interventions were well tolerated. Minor adverse effects were reported in 16 of 82 patients as follows; headache1/1 (Robot Sham/Robot tDCS),

	burning sensation 0/1, sleepiness 1/1, tingling 2/5,redness 0/2, trouble concentrating 0/2.
**Conclusions**	1. Robot-assisted arm training is an effective form of rehabilitation for chronic post-stroke hemiparesis, 2. Supplementary ipsilesional-anode tDCS as performed in our study sample although well tolerated, did not augment training effects, and 3. Residual corticospinal integrity (motor evoked potential presence) in the chronic stroke hemiparetic arm, can be a predictor of clinically important response to intensive arm rehabilitation.

Trials	Fusco et al. 2014 (——)
**Inclusion criteria**	(1) Subjects affected by a first ever stroke, age between 18 and 83 years; (2) an occurrence of ischemic stroke in the territory of middle cerebral artery, as revealed by a magnetic resonance or computerized tomography scan performed before the enrollment; (3) the stroke event occurred within 30 days from the starting of the protocol; (4) no history of moderate to severe cognitive impairment, as evaluated by a neuropsychologist.
**Exclusion criteria**	(1) Inability to perform a motor rehabilitation training; (2) the presence of multiple foci of ischemia or a hemorrhagic stroke; (3) the presence in the patient's history of a previous stroke or global cerebral ischemia; (4) a diagnosis of a major psychiatric disorders or epilepsy; (5) a history of tumor independently from location; (6) the presence of pacemaker; (7) uncontrolled arrhythmias or nonstabilized hearth diseases; (8) dementia or severe aphasia that could have compromised the collaboration for the procedures.
**Details of neurostimulation**	In the EG, tDCS was performed using cathodal montage. The session of cathodal or sham tDCS had a duration of 10 minutes. The Eldith DC Stimulator tDCS model (NeuroConn, Ilmenau, Germany) was supplied to 1.5 mA of electric intensity, by 2 gel-sponge electrodes with a surface area of 35 cm^2 (5 cm × 7 cm), embedded in a saline-soaked solution. Consequently, we delivered a current density of 0.043 mA/cm2 (intensity of 1.5 mA, electrode area 35 cm2). Stimulation was preceded by few seconds during which the current increased gradually to the selected intensity (fade-in phase), eliciting transient sensations that disappeared in several seconds and followed by the same

	few seconds during which current was progressively reduced (fade-out phase). The active electrode was positioned in the primary motor cortex area in the contralateral affected hemisphere (C3/C4according to the International classification system of EEG electrodes placement). The reference electrode was positioned in a noncephalic side, above the right shoulder, contralateral to the electric circuit of the heart. In this way, only the area below the active electrode was stimulated, focusing the passage of current into the selected treatment area.
**Efficacy outcomes**	Canadian Neurological Scale (CNS)<br>Barthel Index(BI)<br>9-hole peg test (9HPT)<br>dynamometers for pinch and grasp forces<br>Upper Limb Fugl-Meyer Scale(UL-FM)<br>Timed Up and Go Test(TUG)<br>6-Minute Walking Test(6MWT)<br>10-Meter Walking Test (10MWT)<br>Rivermead Mobility Index(RMI)<br>Functional ambulation Classification(FAC)
**Safety outcomes**	Some patients of both groups reported the insurgence of upper limb pain during rehabilitation and it limited the quantity of exercises performed for increasing hand forces.
**Conclusions**	An early applied cathodal electrical stimulation did not lead to a higher functional improvement in respect of traditional rehabilitation in patients affected by stroke in subacute phase during their period of inpatient rehabilitation.

Trials	Gong et al. 2020 (ChiCTR2000034846)
**Inclusion criteria**	(1) The duration of within 30 days and the onset of the first-ever stroke, (2) the ipsilesional areas of middle cerebral artery(3) recorded Motor Evoked Potentials of the affected side.
**Exclusion criteria**	(1) Patients with limb dysfunction caused by other nervous system diseases and a history of epilepsy, (2) severe cognitive(3) mental disorders
**Details of neurostimulation**	rTMS: MagproR30 magnetic stimulation instrument produced by Magventure Company in Denmark were applied. Previous study showed that the compensation of the affected hemisphere was not obvious in the early stage of stroke, which manifested by excessive excitement on the contralateral hemisphere. Furthermore, motor symptoms could be improved after the treatment of 1Hz rTMS by activating the nerves of the poststroke cortical motor network.17 Thus, the lowfrequency (1Hz) rTMS was selected for subacute stroke patients. The stimulation time of each sequence was 30 seconds, the interval time was 2 seconds, a total of 40 sequences (a total of 1200 pulses), five times a week, continuous treatment for four weeks. According to previous study and methods, we applied rTMS single pulses of stimulation to evaluate the resting Motion Threshold, amplitude of Motor Evoked Potentials and Central Motor Conduction Time of affected and unaffected hemispheres.<br>tDCS: We adopt the transcranial direct current therapeutic instrument of IS200 produced by Sichuan Intelligent Electronic Industry Co. Ltd. According to evidence-based guidelines on the therapeutic use of tDCS, the stimulation site on the body surface of the anode was the motor area of the affected side, and the

	cathode was located in the contralateral shoulder. In the tDCS cathodic stimulation protocol, the stimulation site on the body surface of the cathode was the contralateral precentral gyrus motor area, and the anode was located in the contralateral shoulder. The stimulation intensity of direct current stimulation was 1.0~2.0MA, 20minutes each time, once a day, 5 times a week, continuous treatment for four weeks as a course of treatment.<br>Control: The patients of sham group only get routine motor training.
**Efficacy outcomes**	Fugl-Meyer Assessment upper limb and lower limb scale, (FMA)<br>National Institutes of Health Stroke Scale<br>Barthel Index Scale
**Safety outcomes**	Except for two transient headaches on the head at the beginning of the stimulation, no adverse effects were observed in this study, such as seizures.
**Conclusions**	1Hz rTMS combined with anode tDCS stimulation protocol could be apreferable rehabilitative strategy for motor recovery in subacute stroke patients.

**Trials**	**Hesse et al. 2011**

	**(NCT 00407667)**
**Inclusion criteria**	(1) Age 18 to 79 years, (2) first supratentorial ischemic stroke with a stroke interval of 3 to 8 weeks duration,(3) participation in a comprehensive inpatient rehabilitation program.
**Exclusion criteria**	(1) Receding epileptic seizures, (2) an electroencephalogram with elevated cortical excitability, (3) sensitive scalp skin, (4) metallic implants within the brain, (5) previous brain neurosurgery.
**Details of neurostimulation**	tDCS was applied via saline-soaked surface sponge electrodes (35 cm^2), connected to a constant current stimulator (Siemens Therapie, Neuroton 827, Munich, Germany). In group A patients (anodal group), the anodal electrode was placed over the presumed hand area of the lesioned hemisphere (C3 position according to the 10-20 system) and the cathodal electrode was placed above the contralateral orbit (C4 position). In group B patients (cathodal group), the cathodal electrode was placed over the presumed hand area of the nonlesioned hemisphere (C3 position) and the anodal electrode was placed above the contralateral orbit (C4 position). In group C patients (sham group), the positions of the electrodes either followed that of group A or group B patients; the order changed consecutively from patient to patient. The panel of the stimulator was invisible for the patients, in group A and B the intensity was set to 2 mA and in group C to 0 mA.
**Efficacy outcomes**	Fugl-Meyer motor assessment score (FMS)<br>Box and Block test (BBT)<br>Modified Ashworth score (MAS)
**Safety outcomes**	85 patients completed the whole study. Relevant side effects did not occur; an uncomfortable tingling under the electrodes (n =

	12, evenly distributed in all 3groups), and transient headaches (n = 4, known history of headaches) did not interrupt the intervention.
**Conclusions**	Neither anodal nor cathodal trans cranial direct current stimulation enhanced the effect of bilateral arm training in this exploratory trial of patients with cortical involvement and severe weakness. Unilateral hand training and up regulation of the nonlesioned hemisphere might also be tried in this population.

**Trials**	**Jin et al. 2019**<br>**(ChiCTR180002008)**

**Inclusion criteria**	1) Adults (aged ≥18 years old) who had experienced their first stroke more than six months ago; 2) upper extremity impairment ≥ second level in the Functional Test for the Hemiparetic Upper Extremity (FTHUE); 3) medically stable; 4) Mini-Mental State Examination (MMSE) score ≥ 21, to ensure the participant could understand the instructions and give consent; 5) not participating in other clinical, drug, or research studies at the same time; 6) passed the safety screening for tDCS.
**Exclusion criteria**	Those who had severe health conditions that required intensive medical care, such as heart failure, pneumonia, a poor nutritional state, or contraindications of tDCS, such as a cardiac pacemaker, cancer, bleeding tendencies, pregnancy, metal implants, a history of seizures, etc., were excluded from the study.
**Details of neurostimulation**	Dual-tDCS: In this study, we used the Soterix Medical 1 × 1 tDCS Low-Intensity Stimulator, and a 1 mA tDCS current was delivered through two 35 cm × 35 cm saline-soaked surface sponge electrodes for 30 min, 5 days per week, for 2 weeks. With references to previous studies and recommendations for tDCS studies in stroke, the cathodal electrode was placed over the primary motor cortex (M1) of the contralesional hemisphere (C3 position, according to the 10/20 EEG system), and with the anodal electrode placed above the ipsilateral M1 position. A head band was used to attach the electrodes. Initially, the current ramped up gradually to 1 mA in 30 s., in order to avoid adverse sensations. The 30-min training duration did not include the 30 s. ramp up time at the start of stimulation and an approximately 30 s. ramp down time at the end of stimulation.

	Control: With regard to the sham condition, the sham button of the device was switched on so that only an approximately 30 s. ramp up and an approximately 30 s. ramp down was delivered.
**Efficacy outcomes**	Fugl Meyer Assessment-Upper Extremity Subscore (FMA-UE)<br>Action Research Arm Test (ARAT)<br>Box and Block Test (BBT)
**Safety outcomes**	None of the patients reported discomfort or severe side effects.
**Conclusions**	The priming effect of tDCS was important to facilitate motor recovery in MT. Significant improvements after concurrent-tDCS and MT in only one motor function test were found when compared with prior-tDCS or sham-tDCS and MT but could not be seen in other outcomes. The concurrent-tDCS seems to be more advantageous and time efficient in the context of clinical trials combining with other kinds of motor rehabilitation training which should be considered in future application.

Trials	Kim et al. 2021 (——)
Inclusion criteria	(1) Patients who agreed to participate in the study; (2) adults aged 20 to 90 years old; (3) patients with chronic stroke (>180 days from stroke onset); (4) patients with no cognitive impairment(>MMSE-K 24)
Exclusion criteria	(1) Patients with infarction or hemorrhage in the bilateral hemisphere or brainstem area; (2) spontaneous extension of the metacarpophalangeal joints of the hand and the interphalangeal joints less than 10° and less than 20°, respectively; (3) serious impairment in balancing or walking; (4) uncontrolled health conditions other than stroke or serious terminal illness; (5) patients with severe stiffness in the shoulders, elbows, wrists or fingers.
Details of neurostimulation	The equipment used for dual tDCS is The Brain Driver v2.1 tDCS (TheBrain Driver v2.1, The Brain Driver, USA), which has the advantage of being portable. For the electrode attachment site, the anodal electrode is the primary motor cortex on the affected side (point C3 or C4 according to the 10-20 system), and the cathodal electrode is the primary motor cortex on unaffected side (point C4 or C3 according to the 10-20 system). and the stimulation protocol applied a constant current of 1 intensity for 20 min.
Efficacy outcomes	Fugle-Meyer Assessment (FMA)<br>Motor Activity Log (MAL)
Safety outcomes	(——)

**Conclusions**	This study has clinical significance in that it presents the possibility of convergence intervention that considers the therapeutic efficiency in clinical practice.
**Trials**	**Lee et al. 2014**<br>**(——)**
**Inclusion criteria**	(1) Had their first stroke within 1 month prior to enrollment, (2) their motor power of the affected shoulder was considered greater than a poor grade (ie, patients could not perform movements against gravity, but they demonstrated complete range of motion when the pull of gravity was eliminated).
**Exclusion criteria**	(1) Patients with contraindications to brain stimulation, previous history of brain neurosurgery or epilepsy, or metallic implants in the brain. (2) Patients who had severe cognitive impairments or aphasia, which made them unable to understand the instructions given by therapists. (3) Patients who could not complete the VR protocol because of poor sitting balance, severely damaged eyesight, or hemispatial neglect.
**Details of neurostimulation**	tDCS was delivered using a Phoresor II Auto Model PM850a via 2 conductive rubber electrodes that were placed in salinesoaked sponges (5.0*5.0cm^2). The cathodal electrode was placed over the hand area of the unaffected motor cortex, whereas the anodal electrode was placed above the contralateral orbit of the eye. To determine the optimal cortical stimulation site representing the hand area, we recorded the motor evoked potential in the unaffected first dorsal interossei muscles using transcranial magnetic stimulation.b The stimulation site was the site where the lowest excitatory threshold, shortest latency, and largest average amplitude in the resting state were recorded. The

	tDCS intensity was 2mA, and the duration of the stimulation application was 20 minutes.
**Efficacy outcomes**	Modified Ashworth Scale, (MAS)<br>manual muscle test (MMT),<br>Manual Function Test (MFT),<br>UE part of the Fugl-Meyer Scale (FMS),<br>Box and Block Test (BBT)<br>Korean-Modified Barthel Index (K-MBI)
**Safety outcomes**	No major adverse effects associated with tDCS or VR were reported by any of the patients who completed all 15 consecutive sessions.
**Conclusions**	In this pilot study, the combination of brain stimulation using tDCS and peripheral arm training using VR could provide additional benefits to the training of UE recovery after stroke over those benefits achieved with either intervention alone. This combination therapy is also safe and easy to apply in clinical settings. We suggest that this therapy is a promising strategy that promotes an improvement of UE recovery in patients with subacute stroke.

Trials	Liao et al. 2020 (NCT02827864)
**Inclusion criteria**	(1) A first ever unilateral stroke, (2) Age above 18 years old, (3) stroke onset more than 6 months, (4) Fugl-Meyer assessment (FMA) scores between 20 and 56, indicating moderate to mild impairments, (5) no severe muscle spasticity at the paretic arm at all joints (the Modified Ashworth Scale scores < 3), (6) adequate cognitive function to follow instructions (the Mini Mental State Examination ≥24).
**Exclusion criteria**	(1) participation in any drug or rehabilitation projects/experiments in the past 6 months, (2) had Botulinum toxin injections in the past 3 months, (3) severe vision or visual perception impairments (e.g., neglect and poor visual field) as assessed by the National Institutes of Health Stroke Subscale, (4) concomitant neurologic, neuromuscular or orthopedic conditions such as brain tumor and Parkinson's disease, (5) any contradictions to NIBS.
**Details of neurostimulation**	A battery-driven direct current stimulator (neuroConn GmbH, Ilmenau, Germany) was used for delivering anodal tDCS. The anode electrode was placed over the iM1, which was the C3/C4 location of the international 10–20 electroencephalogram (EEG) electrode system; while the cathode electrode was placed on the contralesional supraorbital area. The duration of tDCS was 20 min. The size of the electrodes was 35 cm^2 , and the stimulation intensity was 2 mA, resulting in a current density of 0.057 mA/cm^2, which was well within the current safety limit. For the sham tDCS, the stimulation intensity was first ramped up to 2 mA in 15 s and then ramped down to 0 within the next 30 s.
**Efficacy**	Nottingham Extended Activities of Daily Living (NEADL)

**outcomes**	Fugl-Meyer assessment scale of upper extremity (FMA)
**Safety outcomes**	(——)
**Conclusions**	Potential timing-dependent effects of tDCS with MT on daily function and motor control of paretic hand in chronic stroke patients. Sequentially applying tDCS prior to MT could enhance ADL/IADL function more than applying tDCS concurrently with MT or sham stimulation. Hand movement efficiency also improved in the sequentially combined group.

Trials	Lindenberg et al. 2010 (NCT00792428)
Inclusion criteria	(1) Occurrence of ischemic stroke in the territory of the medial cerebral artery at least 5 months prior to enrollment; (2) no previous or subsequent strokes; (3) Medical Research Council (MRC) strength grade of 3/5 in extensor muscles of the affected upper extremity in the acute phase with at least 15 degrees of active wrist dorsiflexion at enrollment; (4) no additional neurologic or psychiatric disorders; (5) no concurrent use of CNS-affecting drugs.
Exclusion criteria	(——)
Details of neurostimulation	Direct current was delivered using a Phoresor® II autostimulator (IOMED, Salt Lake City, UT) through 2 saline-soaked surface gel sponge electrodes (16.3 cm^2 active area).4 Real stimulation consisted of 30 minutes of 1.5 mA direct current with the anode placed over the ipsilesional and the cathode over the contralesional motor cortex (C3 and C4 of the international 10 – 20 EEG electrode system). For sham stimulation, the same electrode positions were used. The current was ramped up to 1.5 mA and slowly decreased over 30 seconds to ensure the typical initial tingling sensation
Efficacy outcomes	Upper Extremity Fugl-Meyer Scale (UEFM)<br>Wolf Motor Function Test (WMFT)
Safety outcomes	No adverse effects were observed by the investigators or reported by the patients except a mild tingling sensation at the site of the electrodes at the beginning of the stimulation, which is a common finding across different studies.

**Conclusions**	The combination of bihemispheric tDCS and peripheral sensorimotor activities improved motor functions in chronic stroke patients that outlasted the intervention period. This novel approach may potentiate cerebral adaptive processes that facilitate motor recovery after stroke.

Trials	Mazzoleni et al. 2019 (NCT02496026)
**Inclusion criteria**	(i) Persons affected by first supratentorial stroke, whose onset time is 25 ± 7 days; (ii) upper limb hemiparesis; (iii) cognitive and speech abilities sufficient to understand instructions and toprovide informed consent; (iv) absence of intense pain due to passive wrist mobilization assessed byVAS <3 (range 0-10); (v) ability to provide written informed consent.
**Exclusion criteria**	(i) Previous epilepsy seizures, (ii) severe EEG abnormalities, (iii) previous neurosurgery interventions including metallic elements, (iv) anticonvulsant medications, (v) inability to keep sitting posture, (vi) other current severe medical problems.
**Details of neurostimulation**	The HDC kit (Newronika Srl, Milano, Italy) was used to deliver tDCS. It consists of a module for programming the stimulation parameters, a module for the stimulation and a kit of electrodes. Two ellipsoidal electrodes (area: 35 cm^2, dimensions: 5 cm × 7 cm) consisting in a conductive-rubber enclosed in a perforated sponge pocket saturated with an electrolytes solution (NaCl 0.9%) were used. The anodal electrode was placed on primary motor area (M1) of the affected hemisphere, whilst the cathodal electrode was placed on the controlateral orbit. M1 was localized considering that it corresponds to C3/C4 location according to the International 10-20 System for EEG. Based on the electrodes dimension and placement, the primary motor area is stimulated by means of 2mA direct current. In order to identify M1 areas and to keep the electrodes in the right location during the stimulation, an ergonomic cap embedded with metal connections placed according to the International 10-20 System

	(MindCap, Newronika Srl, Milano, Italy) was put on the patients head.
**Efficacy outcomes**	Upper extremityof the Fugl-Meyer Assessment Scale (FM/ue), Shoulder-elbow of the Fugl-Meyer Assessment Scale(FM/se) Wrist of the Fugl-Meyer Assessment Scale(FM/w) Modified Ashworth Scale on the wrist (MAS/w) Motricity Index (MI) Box and Block test (B&B)
**Safety outcomes**	(——)
**Conclusions**	The findings in this study showed that the combination of wrist robotic training and tDCS can be used as a safe and effective method for rehabilitation. However, based on the specific intervention no additional effects of tDCS combined with wrist robot-assisted training in comparison with wrist robot-assisted training only were observed in subacute stroke patients. Our results could be due to the lack of HD-tDCS stimulation. In addition, the recovery process of patients undergoing robot-assisted training can be evaluated by means of clinical outcome measures and kinematic parameters.

Trials	Pavlova et al. 2017 (——)
**Inclusion criteria**	(1) First ever stroke, (2) time from stroke more than six months, (3) an ability to voluntarily produce at least some whole hand grip force according to clinical examination.
**Exclusion criteria**	(1) Any other neurological disorder, (2) ongoing psychiatric disorder, (3) multiple stroke locations, (4) a history of epileptic seizures, (5) cardiac pacemakers or metallic implants in the head and neck, (6) electric devices in the body, (7) inability to follow instructions, (8) use of serotoninergic or dopaminergic drugs, (9) drug or alcohol addiction.
**Details of neurostimulation**	Bipolar stimulation was delivered by a batterydriven electrical stimulator (Magstim Company, Whiteland, Dyfed, UK) through a pair of rectangular rubber electrodes (6 × 2.5 cm). Two sessions of 0.5 mA (0.028 mA/cm2) tDCS of the primary motor cortex were performed daily (weekdays) during a four week period. Stimulation sessions continued for 20 minutes separated by a 20 minutes interval. Ramp duration was 10 seconds. A comparable stimulation protocol has been proposed to induce late phase plasticity (Monte-Silva et al., 2013). In sham stimulation experiments, tDCS was delivered for 30 s. Location of the primary motor cortex was determined with transcranial magnetic stimulation (TMS).Motor cortex tDCS was applied with the electrode long axis positioned in the medio-lateral direction with 45 degrees angle, to target the motor cortex. In all cases, the return electrode (5 × 7 cm) was placed above the contralateral orbit. Subjects were seated in a comfortable chair.
**Efficacy outcomes**	Upper Extremity Fugl-Meyer Scale (UEFM)<br>Wolf motor function test (WMFT),

	Box and Blocks test (BBT)
	Maximal grip force measure
**Safety outcomes**	No adverse effects were observed in any of the participants.
**Conclusions**	Four-week visuo-motor training combined with tDCS showed no difference between the Active and Sham groups in the total UE FMA score, which may be explained by heterogeneity of the degree of recovery in the Active group. However, the shoulder-elbow FMA sub-score improved significantly more in the Active compared to the Sham group, which deserves further study.

Trials	Rocha et al. 2015 (NCT01879787)
**Inclusion criteria**	(i) aged at 40–75 years, (ii) able to understand verbal commands, (iii) able to perform some movement of active extension with the paretic wrist (against gravity).
**Exclusion criteria**	(i) Had spasticity scores at the wrist of more than ‘‘3’’ according to the Ashworth scale, (ii) pain of more than ‘‘4’’ on the visual analogue pain scale, (iii) a history of neurological or psychiatric disease, a history of seizures, a cardiac pacemaker, previous surgery involving metallic implants in the skull (cochlear implants, aneurysm clips, and brain electrodes), (iv) having already received mCIMT or tDCS treatment.
**Details of neurostimulation**	During tDCS sessions, the patients were seated in a comfortable chair with head and arm rests. Continuous direct current was applied through a clinical microcurrent stimulator (Soterix, New York, NY) using a pair of saline-soaked surface sponge electrodes (surface 35 cm2). For anodal and sham stimulation, the anode electrode was placed over the primary motor cortex (M1; C3 or C4 according to EEG 10/20 system) of the affected hemisphere and the cathode was placed above supra-orbital region. For cathodal tDCS, the cathode was positioned over M1 of the unaffected hemisphere and the anode was placed above the supra-orbital region. tDCS was administered with a current strength of 1 mA for 13 (anodal tDCS) or 9 min (cathodal tDCS), which had been demonstrated to enhance cortical excitability or inhibition, respectively, lasting for 1 h after the end of stimulation. Sham tDCS was performed by current flow for 30 s, a method shown to achieve a good level of blinding.
**Efficacy**	Fugl–Meyer assessment (FMA)

**outcomes**	Motor activity log (MAL)
**Safety outcomes**	(——)
**Conclusions**	The ipsilesional anodal tDCS has greater impact than the contralesional cathodal tDCS in augmenting clinical responses of mCIMT in rehabilitation after stroke, but both types of stimulation seem to be superior to the sham stimulation.

Trials	Salazar et al. 2019 (NCT02818608)
**Inclusion criteria**	(1) Individuals with ischemic or hemorrhagic chronic stroke confirmed by head CT or MRI at least 6 months before recruitment, (2) who were male or female, (3) aged 18 to 80 years, (4) with moderate (32-47/66), (5) severe hemiparesis (9-31/66) according to the Fugl–Meyer score, (6) have minimal cognitive ability on the Mini Mental State Examination
**Exclusion criteria**	(1) Presented shoulder pain, (2) adhesive capsulitis or glenohumeral luxation, (3) any contraindications for electrical stimulation
**Details of neurostimulation**	Individuals allocated to the tDCS plus FES group received bi-cephalic tDCS and FES at the same time. tDCS electrodes were placed on the participant's head at the M1 area (C3 and C4) according to the electroencephalogram system. Anode electrodes were positioned over the ipsilesional M1 and cathodes over the contralesional M1. tDCS was delivered by a TCT neurostimulator (Research Version) developed by TransCranial Research Ltd. (Hong Kong, China) via a pair of 55cm saline-soaked sponge surface electrodes . The applied current was set to deliver mA bi-cephalic tDCS , with a relative current density of 0.08 mA/m2, for 30 min. The ShamtDCS modality involved the same electrode montage used for active tDCS. The stimulation stopped after a ramp-up and ramp-down period of 30 s each to provide an equivalent scalp sensation.
**Efficacy outcomes**	Movement cycle time<br>Mean reaching phase velocity<br>Mean returning phase velocity<br>Peak velocity

**Safety outcomes**	No serious adverse effects occurred during the treatment.
**Conclusions**	Concurrent tDCS and FES induced a slight improvement in reaching motor performance and handgrip strength of individuals with chronic hemiparesis and moderate to severe UL impairment.

Trials	Shaheiwola et al. 2018 (ChiCTR-ICR-15006108)
**Inclusion criteria**	(1) Age between 35 and 70 years; (2) cerebral hemorrhage or cerebral infraction for the first time; (3) confirmed by head CT or MRI; (4) at least 6 months since stroke onset and an ipsilateral arm Brunnstrom recovery at stages 0–3; (5) conscious and able to communicate; (6) able to sign informed consent himself/herself or with the help of his/her immediate family member.
**Exclusion criteria**	(1) Sequelae after lacunar cerebral infraction; (2) peripheral neuropathy in upper limbs; (3) unconsciousness, sensory aphasia or mental disorders, that may lead to failures in coordinating examination and treatment; (4) history of seizure. (5) serious illnesses, such as heart, liver or kidney diseases, or serious coagulation disorders; (6) history of cognitive disorder, neuropsychiatric disorder, drug or alcohol abuse; (7) organ failure, carcinoma or terminal stroke that seriously affect quality of life beyond hand dysfunction; (8) inability to complete basic course, to persist treatment, or difficult to follow-up; (9) with metal implants or skull defect; (10) existence of skin rash, allergy or wounds at the locations where stimulation electrodes would be placed.
**Details of neurostimulation**	The active tDCS protocol (intensity: 2.0 mA, time of ramp-up: 10 s, time of ramp-down: 10 s) and the sham protocol were programmed by a dedicated computer software package and saved on the device ahead of the usage. Regular parameters of tDCS were chosen based on pilot study prior to the experiment.
**Efficacy outcomes**	Wolf Motor Function Test (WMFT)<br>Modified Ashworth Scale (MAS)

	Non-Invasive Assessment of Muscles European Community project (SENIAM)
**Safety outcomes**	All subjects went through the complete experiment without dropouts. The feedback from subjects showed that the combined protocol was well tolerated by all subjects without any adverse effect.
**Conclusions**	The proposed protocol can facilitate improvements in upper extremity motor abilities in severe chronic stroke patients and is more beneficial than the protocol with FES therapy alone. Our results showed efficacy of the new paradigm with combined intervention in both the central nervous system and the peripheral nervous system.

Trials	Triccas et al. 2015 (NCT01405378)
**Inclusion criteria**	(1) 18 years and above; (2) had a confirmed clinical diagnosis of stroke by a neurologist or stroke specialist, no previous history of another stroke, were >2 weeks post-stroke, (3) had upper and forearm and hand paresis (Medical Research Council scale for muscle strength >2) with minimal spasticity allowed (Modified Ashworth scale ≤2) and partial shoulder flexion with gravity. (4) have good sitting balance and ability to provide informed consent.
**Exclusion criteria**	(1) Impaired gross cognitive function (<24 on the Mini-Mental State Examination (Folstein, Folstein, & McHugh, 1975), (2) any another neurological condition apart from stroke, (3) shoulder pain resulting from shoulder flexion beyond 90º, (4) epilepsy, (5) implants in the brain, (6) previous brain neurosurgery, (7) metal implants in the skull or brain including cochlear implants, (8) medications that influence cortical excitability, (9) previous adverse effects when stimulated with tDCS and pregnancy.
**Details of neurostimulation**	Direct current was transferred by 35 cm2 (7 × 5 cm) rubber electrodes surrounded by saline-soaked pair of surface sponge electrodes using adhesive bandages. The anode was applied over the M1 area of the affected hemisphere and the cathode was positioned on the contralateral supra-orbital region. For the sham stimulation current faded-in and faded-out over 10 seconds at the beginning and end of the 20 minutes in a ramp-like fashion. Stimulation amplitude was 1 mA.
**Efficacy outcomes**	Fugl-Meyer Assessment (FMA)<br>Action Research Arm Test (ARAT)

	Motor Activity Log- 28 (MAL)<br>Stroke Impact Scale (3.0) (SIS)
**Safety outcomes**	After four intervention sessions, a participant with chronic stroke dropped out of the trial due to a skin reaction after receiving four real tDCS sessions, therefore 22 participants completed the trial.
**Conclusions**	No significant differences were found between the real and sham tDCS groups. RT is potentially a more effective for people with sub-acute stroke, influencing UL impairment, function, activities and stroke impact.

Trials	Viana et al. 2014 (——)
**Inclusion criteria**	(1) Had sustained a unilateral stroke within the last six months, (2) were older than 21 years, (3) had residual weakness and/or spasticity of the paretic UL, were able to hold the Wii controller with their paretic hand, (4) had no cognitive deficits, as determined by the scores on the Mini-mental state exam (Lourenc¸o & Veras, 2006).
**Exclusion criteria**	(1) Had histories of seizure, (2) cerebral aneurysm, (3) prior surgery involving metallic implants.
**Details of neurostimulation**	For priming the cortical neural networks to receive the VRT (Bolognini et al., 2009), the anodal tDCS (2 mA) was applied for 13 minutes. Continuous direct currents were transferred via a pair of saline-soaked surface sponge electrodes (surface of 35 cm2) and delivered by a clinical current stimulator (Striat, IBRAMED, Brazil), with a maximum output of 20 mA. The anodal electrode was positioned over the primary motor cortex (M1) (C3 or C4, international 10–20 system) of the affected hemisphere and the cathodal electrode above the contra lateral orbit. For the sham-tDCS, the current flflow was terminated after 30 seconds.
**Efficacy outcomes**	Fugl-Meyer assessment (FMA)<br>Wolf motor function test (WMFT)<br>Modifified Ashworth scale (MAS)<br>Brazilian version of the Stroke Specifific Quality of Life Scale (SSQOL Brazil)<br>Minimal clinically important differences (MCID)
**Safety outcomes**	No adverse events were reported by any of the participants.

**Conclusions**	These findings support that tDCS, combined with VRT therapy, should be investigated and clarified further.

Trials	Yao et al. 2020 (ChiCTR1800019386)
**Inclusion criteria**	(1) Aged 18–80 years; (2) had a first-ever ischemic stroke (silent infarct is allowed) as diagnosed by computed tomography or magnetic resonance imaging image scans; (3) had their first ischemic stroke between 2 weeks to 12 months; (4) can induce motor evoked potential (MEP) of contralesional first dorsalinterossei muscle (FDI) using Transcranial magnetic stimulation.
**Exclusion criteria**	(1) Intracranial or orbital metallic implants, pacemakers or artificial cochlea; (2) previous seizure history; (3) previous history of brain neurosurgery or cerebral trauma; (4) aphasia, unilateral neglect or cognitive deficits (Mini-Mental State Examination score < 20); (5) refused to sign informed consent.
**Details of neurostimulation**	The electrical stimulation device was a transcranial direct current stimulation model IS300 manufactured by Sichuan Intelligent Company of China. Its two conductive rubber electrodes were placed in a saline-soaked sponge (5 × 7 cm 2) when used. The cathodal electrode was placed over the patients' scalp which corresponded to the primary motor cortex (M1) of the unaffected hemisphere, and the region was determined by the induction of stable MEP response in the FDI using transcranial magnetic stimulation. The reference electrode was placed above the contralateral supraorbital region. The current of the experimental group was constant 2 mA for 20 min. For the control group, the current was rapidly increased to 2 mA in the beginning and then slowly tapered down to 0. At the end of the experiment, the current again rapidly ramp up to 2 mA and then slowly ramp-down to 0.

**Efficacy outcomes**	Fugl-Meyer Upper Extremity (FM-UE)<br>Action Research Arm Test (ARAT)<br>Barthel Index (BI)
**Safety outcomes**	4 patients reported tingling sensation in experimental group, lasting up to 2 min after the start of the experiment, and 1 patient reported itching sensation during cathodal stimulation in experimental group, lasting up to 1 min after the end of real stimulation
**Conclusions**	C-tDCS combined with VR can reduce motor impairment, improve function, increase ADL in the affected upper limb in patients with subacute or chronic ischemic stroke than VR alone.

Trials	Abo et al 2013 (——)
**Inclusion criteria**	(1) Brunnstrom stage for hand-fingers of 4 or 5 (ability subjectively to flex and extend all fingers of the affected upper limb in full range of motion); (2) age at the intervention between 18 and 90 years; (3) time between the onset of stroke and intervention of more than 12 months; (4) history of a single stroke only (no bilateral cerebrovascular lesion); (5) no cognitive impairment with a pretreatment Mini Mental State Examination score of more than 26;(6) clinical confirmation of a plateau state, representing no score increase in the Fugl-Meyer Assessment (FMA) in the latest three months;(7) no active physical or mental illness requiring medical management; (8) no history of seizure within one-year preceding the intervention; (9) no documented epileptic discharge on pretreatment electroencephalogram; (10) no current use of antiepileptic medications for the prevention of seizure; (11) no pathological conditions known to be contraindications for rTMS in the guidelines suggested by Wassermann.
**Exclusion criteria**	(——)
**Details of neurostimulation**	LF-rTMS was delivered using a 70-mm fifigure-8 coil and MagProR30 stimulator (MagVenture Company, Farum, Denmark). Each rTMS session consisted of 1200 pulses of 1 Hz rTMS applied to the nonlesional hemisphere over the primary motor area, lasting 20 min. The optimal site of stimulation on the skull was defifined as the location where the largest motor evoked potentials in the first dorsal interosseous (FDI) muscle of the unaffected upper limb was elicited on surface

	electromyography. The resting motor threshold (MT) of the FDI muscle of the unaffected upper limb was defifined as the minimum stimulus intensity that produced a minimal motor evoked response (about 50 μV in at least five of 10 trials) of the muscle at rest. According to the measured resting MT level, the intensity of stimulation was set at 90% of resting MT of the FDI muscle.
**Efficacy outcomes**	The primary outcome measures for this study included the mean change in the FMA score and the mean change in WMFT log performance time. The secondary outcome measure was the change in FAS of WMFT.
**Safety outcomes**	None of the patients experienced any pathological symptoms or any deterioration of motor function in the upper limb during hospitalization.
**Conclusions**	The results of the 15-day rehabilitative protocol showed the superiority of NEURO relative to constraint induced movement therapy; NEURO improved the motion of the whole upper limb and resulted in functional improvement in activities of daily living.

Trials	Chang et al. 2010<br>(——)
**Inclusion criteria**	(i) First-ever cerebral infarction, (ii) post-onset duration of less than one month, and (iii) mild to severe motor deficits of the contralesional upper limb.
**Exclusion criteria**	(i) Any clinically significant or unstable medical disorder, (ii) any neuropsychiatric comorbidity other than stroke, (iii) direct injury to the primary motor cortex, (iv) complete internal carotid artery occlusion, (v) seizure, or (vi) an intracranial metallic implant.
**Details of neurostimulation**	Over a two-week period, patients received 10 sessions of rTMS, which were applied to the primary motor cortex of the affected hemisphere using a Magstim Rapid2® stimulator with two booster modules. Real rTMS involved 50 trains at 10 Hz for 5 s and 90% RMT applied through the coil over the target motor cortex area corresponding to the paretic hand. For subjects who showed no MEP response in the affected hemisphere, the hot spot and RMT were taken by the mirror image of the unaffected hemisphere. A total of 1000 pulses was delivered with a 55-s inter-train interval consisting of 50 s of motor training and 5 sec of rest. Stimulation was applied to the motor cortex by holding the figure-of-8 coil tangentially to the skull. rTMS protocols used in the present study were in accordance with safety guidelines for rTMS applications. As with real rTMS, control rTMS was performed with the coil held at 90º to the scalp using the same stimulation parameters (duration, time, frequency).
**Efficacy outcomes**	The arm score in the Motricity Index (MI-A)<br>The upper limb score in the Fugl-Meyer assessment (FMA-UL)<br>Grip strength

	The Box and Block test (BBT)<br>The leg score in MI (MI-L)<br>The lower limb score in FMA (FMA-LL)<br>Functional Ambulatory Category (FAC)<br>Modified Barthel index (MBI)
**Safety outcomes**	All patients completed their rTMS sessions; no adverse side-effects were reported during the course of the experiment using consecutive multi-session high-frequency rTMS with subthreshold intensity.
**Conclusions**	Results from this study demonstrated that consecutive multisession high-frequency rTMS with subthreshold intensity in the affected hemisphere during the subacute stage of stroke was safe, and might provide an additional beneficial effect on recovery of upper limb motor function in patients with stroke. With regard to upper limb motor function, significant interaction was observed between time and type of intervention, as measured by MI-A. Thus, real rTMS might provide additional improvement in motor function of the affected upper limb immediately following rTMS, and could also facilitate recovery of motor function up to 3 months after stroke.

**Trials**	**Chen et al. 2019 (NCT01947413)**
**Inclusion criteria**	(1) Aged between 30 and 70 years; (2) first-ever ischemic or haemorrhagic stroke; (3) onset ≥6 months; (4) unilateral cerebral stroke with hemiplegia or hemiparesis.
**Exclusion criteria**	(1) brainstem or cerebellar stroke; (2) active psychiatric diseases; (3) severe psychological impairments, such as mental retardation, autism, or severe communication problems; (4) cognitive impairments that may interfere with understanding instructions; (5) progressive disorders, such as neurodegenerative disease; (6) an active medical condition, such as infection; (7) a history of seizure or aneurysm; (8) metal head implants.
**Details of neurostimulation**	Real or sham iTBS was delivered at the same time of day for 5 consecutive days per week for 2 weeks. iTBS gave a 2-s train of bursts, which contained three 50-Hz pulses repeated every 200 ms (i.e., 5 Hz) at an intensity of 80% AMT, every 10 s for 20 times (600 pulses in total) .
**Efficacy outcomes**	Modified Ashworth Scale (MAS)<br>Fugl-Meyer Assessment Upper Extremity (FMA-UE)<br>Action Research Arm Test (ARAT)<br>Box and Block test (BBT)<br>Motor Activity Log (MAL)
**Safety outcomes**	There were no adverse events throughout the study course.
**Conclusions**	I TBS is a non-invasive and safe neuromodulatory stimulation technique that may be a beneficial adjunct to neurorehabilitation for patients with chronic stroke who have relatively low potential for motor recovery.

Trials	Chen et al. 2020 (ChiCTR1800019757)
**Inclusion criteria**	1) Sub-acute stroke patients (cerebral infarction) with upper extremity hemiparesis; 2) 18 to 65 years old; and 3) able to follow the therapist's instructions.
**Exclusion criteria**	1) Cerebral hemorrhage; 2) a history of epilepsy or recent administration of anti-epileptic drugs; 3) medically unstable, such as a severe cardiopulmonary situation, severe liver and kidney dysfunction, or malignant tumor; 4) severe cognitive dysfunction or aphasia with mental illness; 5) severe visual and auditory problems; 6) electronic and/or magnetic implants; and 7) spasticity of the affected upper extremity, with Modified Ashworth Scale >2.
**Details of neurostimulation**	For the FMS group, stimulation was delivered via a parabolic coil (MMC-140 coil, Magpro R30 stimulator, Magventure, Farum, Denmark), at a frequency of 30 Hz, and with an intensity of approximately 20% to 40% of its maximal output without generating local pain. The extensor muscles of the upper extremity and shoulder muscles were sequentially stimulated while patients simultaneously contracted; this was repeated five times for each site. Occupational therapy was conducted immediately after FMS. For the LF-rTMS group, stimulation was delivered via a slightly bent butterfly coil (MCF-B70 Butterfly Coil, Magventure) on the contralesional M1 area, at 45 to the sagittal direction, at an intensity of 90% of the resting motor threshold (RMT), with 1 Hz frequency and 1,500 pulses in total. The RMT was evaluated before therapy using a previously reported method.14 Briefly, the electromyography signal was monitored using disposable Ag/AgCl electrodes. The

	active electrode was attached to the skin overlying the contralesional abductor pollicis brevis, while the reference electrode was placed over the adjacent joint. The RMT was determined as the minimum TMS intensity that generated at least fifive motor evoked potentials of 50 mV peak-to-peak amplitude per 10 consecutive stimuli.
**Efficacy outcomes**	Fugl–Meyer Assessment for upper extremity (FMA-UE)<br>Barthel Index (BI)
**Safety outcomes**	No adverse effects of therapy were reported.
**Conclusions**	FMS improves paretic upper extremity function and leads to better recovery of motor activity than LF-rTMS. FMS may be a novel modality to improve motor function.

Trials	Chen et al. 2021 (NCT03350087)
Inclusion criteria	(1) Frst ever cerebral stroke; (2) under stable condition; (3) unilateral hemiplegia or hemiparesis due to unilateral cerebral stroke; (4) Brunnströmstage of the afected upper limb≥3, and (5) 30 to 70 years of age.
Exclusion criteria	(1) Brainstem or cerebellar stroke; (2) history of seizure, brain aneurysm or arteriovenous malformation;(3) active psychiatric disease; (4) progressive neurodegenerative disease impairing cognitive function; (5) communicated disorders such as aphasia; (6) severe or active medical problems such as cardiac disease or pneumonia; (7) heavy metal implant; (8) pregnancy, (9) severe visual impairment; and (10) inability to follow instructions.
Details of neurostimulation	An iTBS session comprised 2-s train of bursts, containing three pulses at 50 Hz, repeated at intervals of 200 ms, every 10 s for 20 times (a total of 600 pulses). Learning from studies on spaced TBS, two sessions of iTBS were applied with a 10-to-15-min break for a total of 1200 pulses to consolidate and induce longer-lasting changes in cortical excitability. Therefore, two sessions of iTBS with 600 pulses with a 10-min break to have 1200 pulses in total were given to enhance the modulation efects.
Efficacy outcomes	Fugl-Meyer Assessment-Upper Extremity (FMA-UE)<br>Modifed Ashworth Scale Upper-Extremity (MAS-UE)<br>Action Research Arm Test (ARAT)<br>Nine Hole Peg Test (NHPT)<br>Box and Block Test (BBT)<br>Motor Activity Log (MAL)<br>Stroke Impact Scale (SIS)<br>Wilcoxon signed-rank tests

	Mann–Whitney U tests
**Safety outcomes**	All patients could tolerate the intervention without significant adverse effects throughout the study.
**Conclusions**	Applying iTBS over the ipsilesional hemisphere had augmented efficacy on VCT in reducing spasticity, increasing actual use of the affected upper limb, and improving participation in daily life. Additionally, no patients experienced significant acute side effects after receiving iTBS in all patients. In conclusion, iTBS may be a promising and safe treatment option as an adjuvant therapy that could augment the therapeutic effects of neurorehabilitation in stroke patients. A further larger-scale study iswarranted to verify the results.

Trials	Chiu et al. 2020 (——)
**Inclusion criteria**	1) Chronic stable ischemic stroke (>3 months from acute event); 2) Persistent unilateral weakness involving at least the upper extremity verified by clinical neurological examination; and 3) Age 18 to 80 years.
**Exclusion criteria**	1) Seizure history or epileptogenic activity on screening electroencephalogram; 2) Any active unstable medical condition; 3) Pregnancy, schizophrenia, bipolar disorder, alcoholism, or substance abuse; 4) Any condition precluding MRI; and 5) Botulinum toxin use within 2 months.
**Details of neurostimulation**	Contralesional PMC was identified by locating the activation of the hand motor area in the precentral gyrus in an fMRI scan involving gripping movements of the normal hand. On the ipsilesional side, microstimulators were placed on the lateral premotor cortical (LPC) site 5 cm anterior to the international 10-20 system electroencephalographic (EEG) electrode locus C3 or C4, and the supplementary motor cortical (SMC) site 15% of the nasion-to-inion distance anterior to EEG locus Cz. In addition, two microstimulators were placed on ipsilesional sites surrounding the infarct lesion on the PMC and the postcentral gyrus 4 cm apart. In subjects who had a subcortical infarct and intact PMC, the microstimulator pair was placed over the MRI-localized precentral gyrus 1 cm and 4 cm lateral to midline. The stimulus protocol was programmed to a Bluetooth-enabled microcontroller operated by smartphone. Treatment consisted of 40-minute sessions of TRPMS stimulation each day 5 times per week for a total of 20 sessions over 4 weeks. The subject sat in a relaxed position during stimulation. Stimulus pulse duration

	was 100 msec and frequency 0.2 Hz on the contralesional side. Stimulus duration and frequency on the ipsilesional side (perilesional/PMC, LPC and SMC) were 25 ms and 5 Hz respectively. The strength of the stimuli was the maximum generated by the TRPMS device prototype and known to modulate muscle activity and cortical excitability upon stimulation of the motor representation of the muscle.
**Efficacy outcomes**	1) Fugl-Meyer motor arm score;<br>2) Action Research Arm Test (ARAT) score;<br>3) Grip strength using a hand grip dynamometer;<br>4) Key pinch strength using a pinch dynamometer;<br>5) Timed Up and Go (TUG) gait velocity;<br>6) National Institutes of Health Stroke Scale (NIHSS).
**Safety outcomes**	All Adverse events were judged unlikely to be treatment related.
**Conclusions**	Multifocal bilateral TRPMS was safe and showed significant fMRI changes suggestive of functional reorganization of cortical circuits in patients with chronic ischemic stroke.

Trials	Du et al. 2016 (ChiCTR-IOR-14005394)
**Inclusion criteria**	Hemiplegic after the first-ever stroke with onset ranging from 3 to 30 days, and stroke lesions located within the middle cerebral artery territory verified by a computed tomography or magnetic resonance imaging scan.
**Exclusion criteria**	Other neurological diseases, prior administration of tranquilizer, severe aphasia or cognitive impairment, and contraindications for transcranial magnetic stimulation.
**Details of neurostimulation**	Each patient received rTMS daily for five consecutive days. Patients in the high-frequency stimulation group received rTMS as follows: 3 Hz, 10 s, intertrain interval 10 s, 40 trains, total of 1200 pulses at 80–90% rMT on the affected hemisphere. For the low-frequency stimulation group, patients received rTMS as follows: 1 Hz, 30 s, intertrain interval 2 s, 40 trains, total of 1200 pulses at 110–120% rMT on the unaffected hemisphere. The sham group received rTMS with the same parameters (noise, time and frequency) as the 1-Hz rTMS group over the unaffected hemisphere but with the coil rotated 90° away from the scalp so that no current was induced in the brain.
**Efficacy outcomes**	Fugl–Meyer Assessment (FMA)<br>Medical Research Council (MRC) scale<br>National Institutes Health Stroke Scale (NIHSS)<br>Barthel Index (BI) scale<br>Modified Rankin Scale (mRS)
**Safety outcomes**	No adverse effects were observed except for three transient headaches and one tingling sensation on the head at the beginning of the stimulation.

**Conclusions**	Significant motor improvements in real rTMS groups compared with the sham rTMS group under conventional physical and medical therapy at an early stage of stroke. The clinical effects persisted beyond the intervention by at least 3 months, and were accompanied by motor cortex excitability changes.

Trials	Galvao et al. 2014 (——)
**Inclusion criteria**	(1) Stroke onset 6 months; (2) muscle tone at the wrist with a modified Ashworth scale (MAS) score between 1+and 3; (3) age between 30 and 75 years; and (4) absence of cognitive impairment, as determined by the Mini-Mental State Examination.
**Exclusion criteria**	(1) A history of seizure or cerebral aneurysm, (2) used antispasticity drugs within 6months before enrollment, (3) previous surgery involving metallic implants, (4) unstable vital signs, (5) other neurological diseases, and (6) aphasia.
**Details of neurostimulation**	The participants were seated in a comfortable chair with headrest and armrests. The rTMS of the motor cortex was performed with a 70-mm fifigure-8 coil attached to a magnetic stimulator. Before the start of rTMS, the rest motor threshold (MT) from the contralateral first dorsal interosseous muscle was determined. The MT was defifined as the lowest single-pulse TMS intensity required to produce a motor-evoked potential amplitude larger than 50mV (confirmed by surface electromyography) in more than 5 of 10 trials from the first dorsal interosseous muscle. The stimulation site was the site defined for the MT determination. We used the same stimulation parameters used previously by Valle et al to reduce the spasticity in children with cerebral palsy: a frequency of 1Hz on the uninjured hemisphere by stroke and 1500 pulses with an intensity of 90% of MT. Each patient received 1 session of active/sham rTMS per day, always before physical therapy, for a total of 10 sessions. For sham rTMS, a coil disconnected from the stimulator unit was held over the scalp while a second coil,

	connected with the stimulator, was positioned behind the patient's head, without touching the scalp. Thus, no current was induced in the brain, but the patients were exposed to acoustic stimulation (from the second coil).
**Efficacy outcomes**	Modified Ashworth scale (MAS)<br>Upper-extremity Fugl-Meyer assessment (UE-FMA)<br>Functional Independence Measure (FIM)<br>Range of motion<br>Stroke-specific quality-of-life scale (SSQOL)
**Safety outcomes**	No adverse events were reported by any of the participants.
**Conclusions**	Inhibitory rTMS over the unaffected hemisphere in association with physiotherapy reduces the spastic hypertonia in the upper limb, and the effect was maintained for at least 4 weeks after the rTMS sessions. rTMS is a noninvasive and painless neuromodulatory tool that may become an integral part of spasticity management with the practice of physiotherapy in the future.

Trials	Gottlieb et al. 2021 (——)
Inclusion criteria	(i) Age ranging between 18 and 80 years; (ii)first-ever unilateral cerebral ischemic or hemorrhagic stroke in the territory of the middle cerebral artery;(iii) at least two weeks delay between stroke onset and study enrollment; (iv)functional restriction of an upper extremity due to hemiparesis or hemiplegia; (v) sensory perception at the fingertips of the affected hand; (vi) ability to give written informed consent.
Exclusion criteria	Patients with epilepsy, previous seizures and medications lowering the seizure threshold. The presence of contraindications for performing MRI scans.
Details of neurostimulation	rTMS was performed using a PowerMAG Research 100 stimulator (Mag & More, Munich, Germany) with an 80 mm fifigure-of-eigh6t coil. In each rTMS session, 1200 pulses were applied over the contralesional M1 (“motor hotspot” of the hand area), delivered in a single train with a rate of 1 Hz. Stimulation intensity was 100% of the resting motor threshold, which was determined for each participant before the rTMS treatment commenced. The resting motor threshold was defined as the lowest stimulator intensity that produced motor evoked potentials with peak-to-peak amplitude of at least 50 V in the contralateral abductor pollicis brevis muscle in five out of ten trials. Ten rTMS sessions were employed over a period of 12 days (5 sessions per week).
Efficacy outcomes	Modified Ashworth Scale (MAS)<br>The Fugl-Meyer-Assessment for the upper extremity (UE-FAM)<br>A resting-state fMRI

**Safety outcomes**	Five patients reported a mild adverse event within 24 h after the rTMS session (headache: n = 4, pain in contralateral hand: n = 1). One patient with headache needed a low dose of an analgetic. However, the frequency of reported adverse events was equal in both groups.
**Conclusions**	Changes in functional connectivity in patients receiving inhibitory rTMS over the contralesional motor cortex suggest that processes of neuronal plasticity are stimulated.

Trials	Guan et al. 2017 (NCT03163758)
Inclusion criteria	(1) Modified Ashworth Scale (MAS) stroke patients within 1 week after onset with unilateral cerebral subcortex lesion in the middle cerebral artery territory detected by diffusion weighted image, (2) right-handed, (3) without memory loss or intelligence disorder, (4) never suffered stroke before.
Exclusion criteria	(i) a history of stroke or cerebral small vessel disease, (ii) cognitive impairment (Mini-Mental State Examination score ≤24); (iii) a history of serious lung and heart diseases, liver and renal failure diseases or malignant tumors; and (iv) any MRI contraindications.
Details of neurostimulation	All patients received consecutive 10-day rTMS or sham rTMS. We used a Medtronic MagPro type magnetic stimulation device (Medtronic, Minneapolis, MN, USA) and a figure-eight coil (MC-B70, Medtronic). Regarding the safety threshold suggested by the International Federation of Clinical Neurophysiology (IFCN) and related studies,17 our protocol used 5 Hz rTMS applied to the ipsilesional M1 with a stimulation intensity set at 120% of the MT data of the contralateral side M1 because the MTs on lesion side were changed because of motor function destroyed since stroke. The treatment involved 50 trains of 20 pulses with 2-second intertrain intervals daily. In the rTMS treatment group, coils were placed tangent to the scalp, while in the sham group, coils were placed perpendicular to the scalp. The patient wore a 10-20 system EEG cap for scalp location and earplugs to protect their hearing.
Efficacy outcomes	National Institutes of Health Stroke Scale (NIHSS),<br>Barthel Index (BI),

	Fugl-Meyer Assessment Upper Limb/Lower Limb (FMA-UL/LL),<br>modified Rank Score (mRS),<br>resting motor threshold (RMT)
**Safety outcomes**	(——)
**Conclusions**	rTMS facilitates motor recovery of acute stroke patients, and the effect can last to 1 month, except the function improvement on upper extremities could last for 1 year. A single course of rTMS in the acute stage may induce the improvement of upper extremities function lasted for 1 year.

**Trials**	**Harvey et al. 2018**

	**(NCT02089464)**
**Inclusion criteria**	(1) ≥ 18 years of age, (2) An ischemic or hemorrhagic stroke suffered 3-12 months prior to the study; (3) no other known brain abnormalities by history; (4) A one-sided stroke resulting in upper extremity paresis, (4) A Chedoke-McMaster Stroke Assessment arm stage and hand stage of 3-6 for the affected limb
**Exclusion criteria**	(——)
**Details of neurostimulation**	The NBT treatment protocol delivered 1 Hz frequency stimulation over the identified optimal cortical location for EDC. We chose EDC because prognosis for functional hand use in hemiplegia is best for those who can achieve active finger extension. 4 At each visit motor threshold for the target site was determined prior to initiating rTMS treatment. The treatment group received 900 active TMS pulses per session at 110% motor threshold while the sham group received 900 sham TMS pulses per session. The pulse dose was selected to remain well within published safety guidelines. 5 The sham condition was delivered using a sham TMS coil with identical external physical characteristics as the active TMS coil. Internally, the sham coil was based on a typical figure-ofeight design but with wiring wound in a way that both wings of the figure eight turn in the same direction. This results in the magnetic fields generated by the wings to counteract each other such that the resulting E-field generated by the coil is less than 30% in intensity and of a very different in shape than that of the active coil. The sham coil delivers no stimulating E-field at the target site for active TMS stimulation, but instead provides weak E-fields in a wide doughnut-shape region around the target site. This weak circular

	E-field causes identical scalp and muscle sensations as the active coil, but avoids any direct target site stimulation.
**Efficacy outcomes**	Upper extremity Fugl-Meyer (UEFM)<br>Action Research Arm Test (ARAT)<br>Wolf Motor Function Test (WMFT)
**Safety outcomes**	Of the safety population (n=196), a total of 26 serious adverse eventsoccurred in 18 participants. Rates within treatment arms were not significantly different. The most common treatment-related adverse events were arm and hand pain, spasm or myalgia (n=14), head discomfort (n=11), and paresthesia (n=6). These were transient and resolved within 24 hours of onset.
**Conclusions**	Among patients 3 to 12 months post-stroke, goal-oriented motor rehabilitation improved motor function 6 months after end of treatment. There was no difference between the active and sham repetitive trans cranial magnetic stimulation trial arms.

**Trials**	**Hsu et al. 2013**<br>**(NCT-01323881)**

**Inclusion criteria**	Patients with first-ever, subacute (between 2 and 4 weeks after stroke onset),monohemispheric ischemic stroke within the middle cerebral artery (MCA) territory, and mild-to-moderate hand paresis (the motor arm score of the NIHSS = 1-3 out of 4, where 0 = no drift or weakness, 4 = no movement). Infarcts were documented by brain diffusion-weighted imaging using a 3T MRI scanner (Discovery MR750, GE Magnetic Resonance System) within 3 days of stroke onset.
**Exclusion criteria**	Being over 75 years of age, total MCA or M1 infarction, motor arm score of NIHSS = 0 or 4, history of seizures or epileptiform discharges on routine electroencephalogram (EEG), pregnancy, aphasia, apraxia, concomitant neurological diseases or other severe medical diseases (e.g., sepsis, malignancy, hepatic or renal failure), and undetectable aMT from the paretic ECR.
**Details of neurostimulation**	iTBS consists of a brief train of basic theta bursts (5 Hz) lasting for 2 s (10 bursts, each burst containing three pulses of 50 Hz) given every 10 s for 40 trains (a total of 1200 pulses, iTBS1200) at 80% the intensity of the aMT. The aMT was defined as the minimum intensity needed to evoke MEPs greater than 200 in more than 5 out of 10 trials while the subject was maintaining a voluntary contraction at about 20% of the maximum, as monitored by visual feedback. The TBS was performed using a Magstim Rapid2 simulator (Magstim Co., Whitland, UK) with a 70-mm figure-ofeight coil and surface electromyography (EMG) recording system (Synergy EMG/EP system using AgeAgCl electrodes and a bandpass filter from 3 Hz to 10 kHz).
**Efficacy outcomes**	National Institutes of Health Stroke Scale (NIHSS) ,<br>Modified Rankin Scale (MRS)<br>Upper Extremity Fugl-Meyer Scale (UEFM)

	Action Research Arm Test (ARAT)<br>Affected aMT and MEPs from ECR
**Safety outcomes**	The self-reports of adverse effects following iTBS1200 included transient local pain/mild ipsilateral headache (2/6) and discomfort/mild tingling sensation (1/6), but not syncope, paresthesia, dizziness, hearing changes, or fatigue (0/6), suggesting the iTBS1200 was well tolerated throughout the intervention period.
**Conclusions**	Repetitive sessions of iTBS1200 over ipsilesional M1 of subacute stroke patients are safe and the potential benefits encourage a larger trial to determine the efficacy in stroke patients.

**Trials**	**Kim et al. 2020 (NCT02082015)**

**Inclusion criteria**	Patients were eligible for inclusion in the study if they displayed unilateral upper limb hemiparesis in Brunnstrom hand stage rating of 3 to 5 within 90 days after first-ever ischemic stroke onset confirmed by magnetic resonance imaging (MRI) and were aged between 20 and 80 years.
**Exclusion criteria**	Exclusion criteria were hemorrhagic or recurrent stroke, previous history of traumatic brain injury, seizure or cerebrovascular surgery, need for intensive care due to severe complications of stroke, metallic materials in the body (eg, pacemakers, cochlear implants, aneurysm clips), pregnant or lactating women, those who were likely to become pregnant but did not agree to appropriate contraception during the trial, skin lesions around the contralesional M1 which interfered with rTMS, or those who could not regularly receive occupational or physical therapy.
**Details of neurostimulation**	Patients were seated comfortably in a chair during the rTMS. The resting motor threshold (RMT) for the first dorsal interosseous muscle of the unaffected side was measured over the contralesional M1. RMT was defined as the minimum stimulation intensity needed to evoke a response of at least 50 μV in at least 5 of 10 consecutive stimulations. In each session, contralesional M1 was stimulated through an 88-mm-diameter figure-of-eight coil powered by ALTMS (Remed, Korea), with a frequency of 1 Hz and intensity of 100% of the patient's RMT measured in the contralesional M1 for 30 minutes, achieving 1800 stimuli per session. The stimulation intensity and duration were determined based on previous studies of low-frequency rTMS9 and safety guideline.6 For the sham rTMS, the coil was

	positioned perpendicular to the scalp on contralesional M1 with the same intensity and frequency as that in the real rTMS.
**Efficacy outcomes**	Box and Block Test (BBT)<br>upper extremity FMA score;<br>Finger Tapping Test (FTT);<br>hand grip,<br>pinch grip,<br>lateral prehension, and three jaw chuck strength;<br>Brunnstrom hand and arm stage ratings;<br>modified Ashworth Scale (MAS) in the elbow, wrist, and finger flexors;<br>Korean version of Modified Barthel Index (K-MBI)
**Safety outcomes**	Of the safety population (n = 76), a total of 111 adverse events occurred in 48 participants and were more numerous among real rTMS (71 events in real rTMS [64%] vs 40 events in sham rTMS [36%]; P = .038). Most of the events were mild (67 [94%] in real rTMS and 38 [95%] in sham rTMS) and only one serious adverse event (ischemic stroke recurrence) occurred in real rTMS but was determined to be unrelated to the intervention. There were 7 adverse device events (ADEs; 18%) for real rTMS and 2 (5%) for sham rTMS, with no statistically significant difference (P = .154). No serious and unexpected ADEs were noted.
**Conclusions**	This study does not support the superiority of 2-week low frequency rTMS on the contralesional M1 over sham rTMS for upper limb motor recovery when combined with task oriented occupational therapy among patients within 3 months post-stroke. Stroke lesion location may have to be considered in

	further clinical trials using low-frequency rTMS for motor recovery

Trials	Kim et al. 2020 (KCT0001768)
Inclusion criteria	(1) Age older than 19 years; (2) incipient CI confirmed by computed tomography or magnetic resonance imaging examination; (3)CI that resulted in motor and sensory disorders within 1 month before enrolment; (4) could undergo rehabilitation therapy after hospitalization at the Department of Physical and Rehabilitation Medicine of Chonnam National University Hospital; (5) Modified Rankin scale (mRS) score of 2–4; (6) voluntarily signed an informed consent form.
Exclusion criteria	(1) Prior history of brain lesion (e.g., stroke, serious mental illness, loss of consciousness accompanied by head trauma, brain surgery, or seizure disorder); (2) presence of other serious illnesses (e.g., cancer, Alzheimer's disease, epilepsy, head trauma, or cerebral palsy); (3) transient ischemic attack; (4) contraindications to electromagnetic stimulation (e.g., metal implants in the brain, implanted electronic devices in the body such as non-detachable ferromagnetic metals, metal-sensitive implants less than 30 cm away from the brain such as cochlear implants, pacemakers, aneurysm clips or coils, stents, bullet fragments, deep brain stimulation, vagus nerve stimulators, jewelry, or hairpins); (5) continuous convulsion symptoms; (6) previous craniectomy or shunt surgery; (7) increased intracranial pressure symptoms such as headache, vomiting, or nausea; (8) seizure disorder or epilepsy after CI; (9) prior history of stroke accompanied by a clear clinical sign; (10) contraindications to SA (e.g., scalp scarring, inflammation from scalpinjury, infection in the treatment region, inability to stop blood flow due toclotting disturbances such as hemophilia, serious unusual

	response after acupuncture treatment); (11) pregnant or breastfeeding; (12) disagreement with informed consent; (13) scheduled for surgery within 2 weeks.
**Details of neurostimulation**	The rTMS was conducted as follows: a 70 mm fifigure-8 coil and a Magstim Rapid stimulator (Magstim Co., Dyfed, UK) were used to deliver 1 Hz of rTMS to the skull of the contralesional hemisphere at the site that elicited the largest motor-evoked potentials (MEPs) in the first dorsal interosseous (FDI) muscle of the unaffected upper limb. One LF-rTMS session consisted of 1200 pulses and lasted for 20 min. Stimulation intensity was set to 80% of the motor threshold of the FDI muscle, which was defifined as the lowest intensity of stimulation that provoked MEPs. All patients sat in a reclining wheelchair and were asked to relax as much as possible with their heads strapped to a headrest.
**Efficacy outcomes**	Fugl–Mayer assessment (FMA)<br>National Institutes of Health Stroke Scale (NIHSS)<br>Modified Barthel index (MBI)<br>Functional independent measurement (FIM)<br>K-MMSE score<br>ASHA-NOMS score<br>European Quality of Life-5 Dimensions (EQ-5D),<br>Modifified Ashworth scale (MAS)<br>Hand grip strength test<br>MEPs<br>Modified Rankin Scale (MRS)<br>9 hole peg test (9HPT)
**Safety outcomes**	No adverse events that were related to the intervention occurred in this study.

**Conclusions**	First, LF-rTMS over the contralesional hemisphere may have long-term therapeutic effects on upper extremity motor-function recovery and on improving activities of daily living. Second, simultaneous application of SA and LF-rTMS did not show the positive synergistic effects of SA and rTMS on motor-function recovery, cognitive function, activities of daily living, walking, quality of life, and stroke severity.

Trials	Krewer et al. 2014 (——)
**Inclusion criteria**	(1) Hemiparesis caused by a stroke or a traumatic brain injury; (2) spasticity of an upper extremity, with a score of 1 to 3 on the Tardieu Scale; (3) ages between 18 and 75 years.
**Exclusion criteria**	(1) Metal implant in the head or within the stimulation area; (2) medical implanted devices (cardiac pacemaker, cochlea implant, or medication pumps); (3) pregnancy; (4) comorbidity with other neurodegenerative disorders or other neurologic, orthopedic disorders; (5) increased intracranial pressure; (6) unstable fractures of the paretic upper extremity.
**Details of neurostimulation**	The rpMS consisted of 5000 stimuli at a stimulation frequency of 25Hz, a train duration of 1 second, and an intertrain interval of 2 seconds. Intensity was individually set at 10% above the level that evoked a wrist or elbow movement taken at rest. Stimuli were distributed consistently among extensors and flexors of the upper and lower arm. Magnetic stimuli were generated by Signal software (Signal for Windowsa), and the digital outputs were fed through an analogue-digital converter (Micro 1401 mk IIa) into the magnetic stimulator (P-Stim 160b). The P-Stim 160 magnetic stimulator generated double cosine pulses with a magnetic induction of maximally 1 tesla. Two butterflfly magnetic coils (diameter, 2 100mm) were used for interventions: an active coil, connected with the stimulator, producing typical discharge noises; and a nonactive, nonconnected passive coil. Patients in the rpMS group were treated with the active coil. The same procedure was followed in the control group, but the nonactive coil was used; the active coil produced the typical noise on the side.

**Efficacy outcomes**	Modified Tardieu Scale (MTS)<br>Fugl-Meyer assessment (FMA)<br>Barthel Index<br>Hamilton Depression Scale-7<br>Patient questionnaire
**Safety outcomes**	(——)
**Conclusions**	Therapy with rpMS increases sensory function in patients with severe limb paresis. The magnetic stimulation, however, has limited effect on spasticity and no effect on motor function.

Trials	Kuzu et al. 2021 (NCT04562415)
**Inclusion criteria**	(1) Ischemic stroke with a disease interval of 6 months to 2 years, (2) aged between 18-80 years, (3) Mini mental test score 24, (4) upper limb Brunnstrom recovery stage 3 to 5, (5) upper extremity (elbow, wrist and finger) spasticity level Modified Ashworth Scale (MAS) 1+ to 3.
**Exclusion criteria**	(1) Hemorrhagic stroke, (2) history of epilepsy, (3) a cardiac pacemaker,(4) pregnancy, (5) history of previous stroke or ischemic attack,(6) neurological diseases other than stroke, (7) metallic implant in brain or scalp (including cochlear implant), (8) previous brain surgery, (9) orthopedic disease that prevents upper extremity movements, (10) poor general health (due to heart failure, chronic obstructive pulmonary disease, severe infection, etc.), (11) diagnosis of malignancy, (12) injections of botulinum toxin in the last 6 months for the affected upper extremity, (13) drug or dosage changes of anti-spasticity medications in the last 1 month periods
**Details of neurostimulation**	In the real rTMS group, 10 sessions of non-lesional hemispheric upper extremity motor area (M1) were targeted and inhibitor rTMS was applied with a frequency of 1 Hz consisting of 1200 pulses for 20 min. In the real cTBS group, 10 sessions of non-lesional hemispheric upper extremity motor area (M1) were targeted and cTBS was administered for a total of 600 pulses consisting of 3 burst stimulations of 50 Hz repeated every 200 ms for 40 s. Sham cTBS was applied in the same protocol but using sham coil.
**Efficacy outcomes**	Modified Ashworth Scale (MAS),<br>Upper Extremity Fugl-Meyer Motor FunctionScale, (UEFM)

	Functional Independence Measure, (FIM)<br>Motor Activity Log-28<br>Brunnstrom upper extremity and hand motor recovery stage
**Safety outcomes**	In our study, no side effects were observed in any of the patients during and after TMS applications.
**Conclusions**	In this study, it was observed that real cTBS or real rTMS combined with PT provided improvement on upper extremity motor functions and daily living activities in chronic ischemic stroke patients, but improvement in spasticity was limited.

Trials	Long et al. 2018 (——)
**Inclusion criteria**	1) First-ever stroke within the last 6 months; 2) a clinical diagnosis of cerebral subcortical infarction (CI) in the territory of the middle cerebral artery or supratentorial intracerebral hemorrhage (ICH) without invasion into the cerebral cortex confirmed with computed tomography (CT) or magnetic resonance imaging (MRI); 3) at intervention between 30 and 80 years; 4) moderate motor and sensory deficit of the upper limb; 5) no cognitive impairment, as indicated by a Mini Mental State Examination score of greater than 26; 6) no active physical or mental illness requiring medical management; 7) no history of seizure within the two years preceding the intervention; 8) no current use of antiepileptic medications for prevention of seizure; 9) no pathological conditions known to be contraindications for rTMS, as specified in the guidelines (Rossi et al., 2009).
**Exclusion criteria**	(1) Patients with relevant aphasia or cognitive impairments, which might interfere with the understanding of instructions for motor testing. (2) participants had apraxia; (3) neglect; (4) visual field deficits; (5) coexistent psychiatric, general neurological, medical or orthopedicillnesses; (6) accepted contraindications for TMS (e.g., pacemakers, metallic objects in the head, or a history of epilepsy).
**Details of neurostimulation**	For the LF-rTMS group, 1 Hz rTMS was performed over the contralesional hemisphere hotspot at 90% of RMT for a total of 1000 pulses. Sham stimulation over the upsilesional M1 cortex was performed with the coil held at an angle of 90◦ to the

	ipsilesional scalp to reproduce the noise associated with the 10 Hz stimulus.<br>For the LF-HF combining group, the patients first received 1000 pulses of 1 Hz LF-rTMS over the contralesional M1. Then, 10 Hz rTMS was applied to the ipsilesional hemisphere motor hotspot at 90% of RMT for 10 seconds followed by an interstimulus interval of 50 seconds. A total of 1000 pulses of 10 Hz rTMS were applied.<br>The sham group received sham stimulation at the same sites in the same order as the LF-HF rTMS group. The coil was held at an angle of 90◦ to the scalp to reproduce the noise of 1 Hz or 10 Hz stimulus for 1000 pulses.
**Efficacy outcomes**	Fugl-Meyer Assessment (FMA)<br>Wolf Motor Function Test (WMFT)
**Safety outcomes**	No significant change in vital signs was observed throughout the intervention. No patient complained of pain or discomfort immediately after rTMS or during the follow-up visits. No patient experienced any pathologic symptoms, such as seizure or deterioration of motor function in the upper limb. All of the three protocols were well tolerated in the post-stroke patients.
**Conclusions**	The results indicated that both LF- and LF-HF rTMS were effective in promoting upper limb motor recovery in patients with acute stroke. Combining HF- and LF-rTMS protocol in the present study is tolerable and more beneficial for motor improvement than the unilateral use of LF-rTMS alone.

Trials	Matsuura et al. 2015 (UMIN000016021)
**Inclusion criteria**	(i) Had a stroke lesion within one hemisphere as verified by magnetic resonance imaging; (ii) showed mild to moderate motor impairment in one hand with a score equal to or less than 63 for the Fugl-Meyer assessment (FMA) and could extend at least 20 at the wrist for MRCP measurement; and (iii) started the rTMS session within 30 days after stroke onset. Patients with any neuropsychiatric comorbidity other than stroke and with contraindication to rTMS, i.e. patients with metal within the brain, a cardiac pacemaker, pregnant or with a history of seizure, were excluded.
**Exclusion criteria**	(——)
**Details of neurostimulation**	The stimulation was performed based on the method of Fregni et al., at a rate of 1 Hz, and with an intensity of 100% motor threshold for 20 min (1200 pulses) per day. The mean stimulus intensity was 43.6% (30%–65%) of the maximum level for the stimulator output. Sham rTMS was performed by placing the coil perpendicularly to the scalp to reproduce the noise of a 1 Hz stimulus and tactile sensation on the scalp without cortical stimulation.
**Efficacy outcomes**	Fugl-Meyer assessment (FMA)<br>Purdue Pegboard Test (PPT)<br>grip strength before and after rTMS sessions in a blinded fashion.
**Safety outcomes**	All patients completed their rTMS sessions and did not report any adverse effects.

**Conclusions**	Our findings suggest that low-frequency rTMS to the contralesional motor cortex facilitates functional recovery of paretic limbs in acute stroke patients through enhancing the the neuronal activity of ipsilesional motor and pre-motor areas.

Trials	Meng et al. 2020 (——)
**Inclusion criteria**	(1) Ischemic or hemorrhagic lesion within 1 hemisphere documented by computed tomography (CT) or magnetic resonance imaging (MRI); (2) 30 to 60 days after the first-ever stroke; (3) aged 30 to 70 years old; (4) an National Institute of Health Stroke Scale (NIHSS) of 1 to 15 points at enrollment (Vanacker et al., 2016).
**Exclusion criteria**	(1) A large area of cerebral infarct or hemorrhage in the frontal and/or temporal lobe; (2) major circulatory, respiratory disease, neurological disease/deficit other than stroke; (3) severe limb orthopedic conditions; (4) recent use of drugs that alter cortical excitability (e.g. sedation, antidepressants); (5) aphasia, spatial neglect, visual field deficit, or emotional problems; (6) history of seizure attack, dementia, cognitive impairment, or other neurodegenerative diseases; (7) TMS contraindications (e.g., pacemakers or metallic objects in the head).
**Details of neurostimulation**	We performed transcranial magnetic stimulation using rTMS with a 125-mm figure-of-eight coil; 1 Hz rTMS trains consisting of 1200 pulses at 100% of rMT were applied over the motor representation of the abductor brevis pollicis in the contralesional M1. iTBS was performed at 60–80% of rMT over the ipsilesional M1, consisting of bursts containing three pulses at 50 Hz repeated at 200-ms intervals for 2 seconds (i.e., at 5 Hz). A 2-second train of iTBS was repeated every 10 seconds for 1200 pulses together (Lin et al., 2019). The initial intensity of iTBS was 60% rMT and increased 5% rMT daily until 80% rMT unless the patient had discomforts like headache or nausea.Once there were uncomfortable symptoms, the intensity

	would not change until the patient could tolerate. Because many patients had a low motor function and could not complete active motor thresh old (AMT) measurement, we chose not to measure AMT and instead used rMT as a reference for stimulation intensity (Pedapati et al., 2015). For sham rTMS, the coil was held at 45◦, touching the skull not with the center but with the rim opposite the handle.
**Efficacy outcomes**	Upper extremity Fugl-Meyer Assessment (UEFMA) and Barthel Index (BI) by an independent assessor at two visits before and after rTMS sessions. Electrophysiological measures included corticomotor excitability, probing maximal amplitude, and latency of MEP from paretic abductor brevis pollicis (ABP), extensor digitorum communis (EDC) and biceps brachii
**Safety outcomes**	The self-reports of adverse effects following stimulation included light nausea (Group A: 1 case; Group B:1 case) and mild headache (Group A: 2 cases, Group B:1 case), which disappeared within 5 minutes after stimulation. And they gradually developed tolerance within the fourth treatment. No other adverse effects like a mild tingling sensation, syncope, paresthesia, dizziness, hearing changes, or fatigue appeared, suggesting that iTBS 1200 was well tolerated throughout the intervention period.
**Conclusions**	The combining of 1 Hz rTMS and iTBS protocol in the present study is tolerable and more beneficial for motor improvement than the single use of 1 Hz rTMS in patients with subacute stroke.

Trials	Miu et al. 2020 (——)
**Inclusion criteria**	Patients with recent stroke aged >18 years admitted to the rehabilitation unit of an extended care hospital were recruited. All stroke diagnoses were ascertained by neuroimaging. The inclusion criteria were subjects with ischemic or hemorrhagic stroke who could follow commands. Only participants who could read, write, and communicate in Chinese were recruited.
**Exclusion criteria**	(1) motor, sensory, or global aphasia; (2) inability to reach and grasp a dowel at least once; (3) conditions contraindicated to rTMS/tDCS, such as known seizure and had taken drugs known to lower the seizure threshold; and (4) having an intracranial metallic implant. Patients with stroke onset of >1 month were also excluded. Information collected included basic demographics, side of hemiparesis, and date of stroke onset.
**Details of neurostimulation**	Single-pulse TMS was used to measure non-lesioned motor cortex excitability. Stimulation was administered using a PowerMAG 100 clinical stimulator (MAG and More GmbH, Munich, Germany) with air-cooled coil and evoked responses were measured from the abductor pollicis brevis (APB) on the non-paretic side. The "hot spot" was identified as the optimal scalp position for eliciting the largest motor evoked potential (MEP) from the non-affected primary motor cortex (M1) representing the APB area consistently. Resting motor threshold was determined by systemically reducing the stimulus intensity over M1. Motor threshold is defined as the lowest intensity level required to induce MEP with peak-to-peak amplitude of at least 50 μV in 5 out of 10 consecutive trials [23]. rTMS was then

	applied at 1 Hz at 90% of MT to the non-lesioned hemisphere extensor digitorum communis M1 for 20 minutes.<br>tDCS was administered via Soterix medical 1×1 tDCS. A saline-soaked cathodal electrode (5 cm×7 cm) was applied on the scalp overlying the unaffected motor cortex, while the anodal electrode was placed on the contralateral forehead above the orbit. The current used was 1.5 mA, and the duration of stimulation was 20 minutes
**Efficacy outcomes**	Motor Assessment Scale (MAS)<br>modified Ashworth Scale (AS)<br>Fugl-Meyer arm score test (FM)<br>Nine-Hole Peg Test (9HPT)<br>modified Barthel Index (mBI)
**Safety outcomes**	(——)
**Conclusions**	Both interventions produce a statistically significant improvement in upper limb function. There was no statistically significant difference between the two intervention methods with respect to motor performance. It is suggested that a larger study may help to clarify the superiority of either methods.

Trials	Obayashia et al. 2020 (——)
**Inclusion criteria**	(1) Medically stable; (2) hospitalized adults within two weeks of stroke; (3) age20–89 years; (4) intact skin on the hemiparetic arm; (5) adequate cognition to participate; (6) first-ever, severe UE paresis (stroke impairment assessment set (SIAS): knee-mouth test range 1–3; finger function test 1a - 3).
**Exclusion criteria**	(1) Past history of stroke, brain injury or brain tumor; (2)metal implant in head or within stimulation area;(3) dysphasia; (4) history of cardiac arrhythmia with hemodynamic instability; (5) comorbidity with neurodegenerative diseases and mental disorders; (6) uncontrolled seizure disorder; (7) disturbed consciousness; (8) implanted stimulator (such as cardiac pacemaker); (9) pregnancy.
**Details of neurostimulation**	rPMS was applied to upper limb muscles including extensor digitorum communis (EDC), extensor carpi radialis (ECR), flflexor digitorum superfificialis (FDS), triceps brachii, biceps brachii, and anterior or middle head of deltoid. We used a peripheral magnetic stimulator (Pathleader, IFG, Sendai, Japan) for rPMS treatment. This round coil stimulator generated biphasic 350 sec with magnetic gradients of up to 15 kT/sec, thus producing repetitive contraction-relaxation cycles that enhance proprioceptive input from target muscles. We applied an intermittent ON/OFF stimulation protocol consisting of three times 10 consecutive stimulations for each muscle per session, where one stimulation (ON) was continued for 2 sec with a frequency of 30 Hz and intensity of 70% MSO (maximal stimulator output: corresponding to 0.65 Tesla), and then the stimulation intervals (OFF period) were set at 2 sec. Deltoid,

	triceps brachii, ECR and EDC were mandatory target muscles for rPMS. Additional application of rPMS to flexor muscles (biceps brachii and FDS) was given only to patients with flflaccid muscle tone. The stimulation session was conducted with patients sitting on a chair in front of a table.
**Efficacy outcomes**	Upper-extremity(UE)<br>Fugl-Meyer Motor Assessment Scale (FMA)<br>Wolf motor function test (WMFT)<br>functional ability scale (FAS)<br>box and block test (BBT)
**Safety outcomes**	There were no adverse events such as pain or discomfort.
**Conclusions**	This is the first report to describe beneficial effects of rPMS on severe UE dysfunction during early acute phase of stroke when applying rPMS to the whole UE. These findings would contribute to the justification for specific treatment parameters to maximize severe UE recovery after stroke. In addition, we strongly desire that our results might lead to the establishment of a definitive approach to the improvement of severe upper extremity (UE) paresis immediately after stroke onset.

**Trials**	**Pinto et al. 2019 (NCT02208466)**
**Inclusion criteria**	（1）Adults with hemiparesis/hemiplegia due to ischemic stroke within the previous 2 years, (2) upper extremity Fugl-Meyer (FMA) motor score >11 and <56, (3) pre-stroke disability score <3 on Modified Rankin Scale (mRS).
**Exclusion criteria**	Unable to understand instructions, TMS contraindications,concurrent medical condition likely to worsen functional status within 6 months, score of 24 or higher on Hamilton Depression Rating Scale (HDRS), joint or paretic extremity pain, pregnancy, recent intake of SSRI or any medication likely to interact with SSRIs (5-week washout period was required before enrollment in either case).
**Details of neurostimulation**	Low-frequency rTMS was applied at 100% rMT intensity, 1 Hz, 1200 pulses as a single, continuous train lasting 20 minutes. rMT and "hot spot" were confirmed on each stimulation day, and each day's stimulation intensity was used for the low-frequency rTMS protocol. We used the same swim cap for each subject to mark the "hot spot" location on each stimulation session. We used MagPro stimulators (MagPro G3 Main, X100+Option Model; serial number: 1099).<br>For sham-rTMS, parameters were identical except the coil was flipped over to the sham side and an electrode (Ambu Neuroline Surface Electrodes) was placed over the forehead to deliver a small current mimicking stimulation sensations; this electrode was also used in active rTMS but without a current (Smith JE1, Peterchev AV.MagVenture 2017 Cool-coil system: user manual US 501-1282).
**Efficacy**	JTHF and FMA

**outcomes**	Modified Ashworth Scale (MAS),<br>Beck's Depression Inventory (BDI),<br>Mini-Mental State Examination (MMSE),<br>Medication diary,<br>Antidepressant side effect,<br>rTMS side effects questionnaires.
**Safety outcomes**	No severe adverse events occurred, and there were no significant differences in all adverse events between groups
**Conclusions**	Combined fluoxetine and low-frequency rTMS treatment of the unaffected hemisphere improved motor function in stroke beyond the effects of fluoxetine alone and placebo. Fluoxetine may have had a relatively detrimental effect, leading to increased intracortical facilitation and decreased motor function improvements compared with placebo. Further mechanistic stroke trials are needed to clarify the effects of both treatments.

Trials	Rose et al. 2014 (——)
**Inclusion criteria**	(1) Stroke onset >6 months prior, (2) passive range of motion of paretic UE within functional limits, (3) Upper Extremity Fugl-Meyer Motor (UEFM-M) assessment shoulder/elbow subcomponent score between 15–25 and (4) 18–90 years of age.
**Exclusion criteria**	(1) History of epilepsy, brain tumor, learning disorder, intellectual or developmental disabilities, drug or alcohol abuse, dementia, major head trauma, or major psychiatric illness, (2) history or radiographic evidence of arterio-venous malformation, intracortical hemorrhage, subarachnoid hemorrhage, or bilateral cerebrovascular disease, (3) history of implanted pacemaker or medication pump, metal plate in skull, or metal objects in the eye or skull, (4) use of medications known to lower seizure threshold, (5) pregnancy, (6) pain in paretic UE that would interfere with movement, (7) inability to follow 3-step instructions, (8) orthopedic condition in back or upper extremity, (9) impaired corrected vision that would alter kinematics of reaching, (10) previous exposure to rTMS.
**Details of neurostimulation**	rTMS was delivered using a Magstim Rapid2 Stimulator (Magstim Company, Whitland, UK) with an air-cooled figure-8 coil (70 mm in diameter per loop) over the contralesional M1, focused over the optimal spot for stimulating the extensor carpi radialis (ECR) muscle. The ECR is important in attaining hand position for reach to grasp, and pointing to and touching objects in the environment, all of which are foundational movements for a functional upper extremity. First, the “hot spot,” the optimal scalp position for consistently eliciting the largest motor evoked potential (MEP) from the contralesional M1 representation

	corresponding to ECR, was determined. Next, resting motor threshold (rMT) was determined by decreasing the stimulus intensity over the hot spot. rMT was defined as the lowest stimulator output that elicited MEPs with a peak to-peak amplitude of 50 V in 6 of 10 trials. The center of the figure-8 coil was positioned tangentially over the "hot spot," and oriented perpendicular to the central sulcus for optimal stimulation of the underlying tissue. A total of 1200 pulses were delivered as a single 1 Hz train, at 100% of rMT, established at each session. These stimulation parameters have been used in previous studies and fall within the current recommended safety guidelines for rTMS. Sham-rTMS was delivered using a placebo coil that looks like and imitates the sound of a real coil.
**Efficacy outcomes**	Wolf Motor Function Test (WMFT)<br>Grip, Lateral Pinch (LP),<br>Palmar Pinch (PP),<br>3-Jaw Chuck (3JC) force,<br>Upper Extremity Fugl-Meyer Scale (UEFM)<br>Action Research Arm Test (ARAT)<br>Semmes-Weinstein Monofilaments<br>Modified Ashworth Scale (MAS)<br>Motor Activity Log (MAL)<br>Late-Life Functioning Disability Index (LLFDI)
**Safety outcomes**	Administration of low-frequency rTMS for 20 minutes, 4 times/week followed by 1 hour of affected UE functional task practice was both feasible and well-tolerated by all participants with no adverse events from either aspect of the intervention.
**Conclusions**	Although the contralesional hemisphere revealed somewhat greater intracortical inhibition following the intervention,

	changes in this variable alone were not sufficient to affect larger joint functional UE movements. Disinhibition of the contralesional cortex is one factor contributing to impaired UE motor ability following stroke. The primary stroke insultand the resultant decreased descending drive to the UE motor neuronal pools are not directly remediated by contralesional low-frequency rTMS and thus may be greater contributors to limb paresis. Although inter-hemispheric balance may be reestablished through down-regulation of the hyper-excitable contralesional hemisphere by rTMS, further examination of its role in addressing post-stroke UE impairment is needed.

Trials	Senióws et al. 2012 (——)
**Inclusion criteria**	(a) Male and female patients in the age range of 20 to 75 years; (b) first hemispheric stroke documented by computed tomography or magnetic resonance imaging; (c) time from onset of symptoms ≤3 months; (d) moderate paresis of the affected upper extremity (National Institutes of Health Stroke Scale [NIHSS]—motor arm score of 1 to 3).
**Exclusion criteria**	(a) Other concomitant neurological or psychiatric illnesses; (b) history of head injury with loss of consciousness; (c) use of a drug that could have an effect on cortical excitability; (d) severe aphasia, apraxia, dementia, perception disorders, and other serious medical conditions.
**Details of neurostimulation**	Repetitive transcranial magnetic stimulation was performed by a single investigator (KW), using a Magstim Rapid Stimulator (Magstim Company, Whitland, UK) equipped with an air-cooled figure-of-eight coil (each loop 70 mm in diameter). The coil was placed tangentially to the scalp over the hand area of the primary motor cortex. The exact site of stimulation was defined as the location where stimulation of a slightly suprathreshold intensity elicited the largest motor evoked potential (MEP) in the first dorsal interosseous (FDI) muscle. The placement of the coil was maintained by marking the actual point on the skin of the head. Magnetic stimulation was applied at 90% of the resting motor threshold (rMT) at 1 Hz frequency. A total of 1800 pulses were generated during each session. Dosing parameters were based on previous studies. The rMT was determined in each subject once before treatment and was defined as the minimum stimulus intensity able to elicit MEP of

	at least 50 mV in at least 5 of 10 consecutive stimulations. MEPs were recorded in a belly–tendon (respectively, cathode–anode) montage on the skin overlying the FDI muscle of the unaffected hand. The FDI muscle is relatively large, easily distinguishable from surrounding muscles, and enables close-spaced electrode placement. It is also routinely used in accordance with clinical guidelines. The electromyography signal was not amplified, filtered, or digitized for further analysis.<br>Sham stimulation was performed with a coil that imitates the sound of a real TMS coil. The stimulation parameters were chosen in accordance with the current safety guidelines for rTMS.
**Efficacy outcomes**	Wolf Motor Function Test (WMFT)<br>the upper extremity part of the FuglMeyer Motor Assessment (FMA)<br>National Institutes of Health Stroke Scale (NIHSS)
**Safety outcomes**	(——)
**Conclusions**	Our study suggests that suppression of contralesional motor cortex activity by rTMS is not yet an evidence-based method during early neurorehabilitation for patients such as those in our trial. Larger trials that stratify participants, perhaps, based on individual patterns of cortical activation or by degree of hand motor function, may be warranted.

Trials	Sharma et al. 2020<br>(CTRI/ 2016/02/006620)
Inclusion criteria	(1) Patients aged 18-75 years, (2) with first ever acute ischemic stroke, (3) within last 15 days documented by computed tomography or magnetic resonance imaging scan of the head, (4) National Institutes of Health Stroke Scale (NIHSS) of 4-20.
Exclusion criteria	(1) Participants who were medically unstable, (2) pregnant, (3)had coexistent brain lesions (tumor, infection), (4) comatose, (5) mechanical ventilation, (6) history of epilepsy, (7) having any surgical implant or pacemaker.
Details of neurostimulation	Low frequency rTMS was performed using Magstim Rapid stimulator equipped with air cooled fifigure of 8 coil (70mm), ie, biphasic pulse was used for rTMS. The resting motorevoked potential (MEP) was ascertained using an electromyogram, recording from the abductor pollicis brevis in keeping with the International Federation of Clinical Neurophysiology recommendations. The coil was placed tangentially to the scalp over the hand area of the primary motor cortex to calculate hot spot. Hot spot was defined as the location on the scalp where stimulation of a slightly supra threshold intensity elicited the largest MEP in the abductor pollicis brevis muscle. After the hot spot was identifified, resting motor threshold was determined using the lowest stimulus intensity to produce MEP of >50mV peak to peak amplitude in 5 of 10 subsequent trails. If no MEP was obtained at the time of hot spot calculation in the affected ipsilesion M1, then the hotspot was defifined as the symmetric location to the contralesion M1. The stimulation parameters were chosen in accordance with the safety guidelines for rTMS. Total 750 pulses, 75 trains using low frequency (1 Hz) with inter

	train interval of 45 seconds at calculated intensity of 110% resting motor threshold (Fc3/Fc4), was administered to the randomized patient by a qualifified technician in the Institute TMS Laboratory. Localization was done using 10-20 electroencephalogram (EEG) method. Sham rTMS pulses were administered using the same stimulation parameters.
**Efficacy outcomes**	mBI score<br>functional independence score (mBI) >90,<br>Fugl-Meyer Assessment of upper extremity,<br>Fugl-Meyer Assessment of lower extremity,<br>Modified Rankin Scale (MRS),<br>National Institutes of Health Stroke Scale (NIHSS)
**Safety outcomes**	One participant in the real TMS arm developed seizure 18 hours after the fourth session and about 1 hour after waking from sleep in the morning. He was treated with phenytoin. The event was reported to the institute ethics committee and later published. The participant did not receive any more TMS sessions. No other adverse events were reported in the participants.
**Conclusions**	In first ever subacute ischemic stroke participants, 1-Hz low-frequency rTMS on contralesional motor cortex along with conventional physical therapy caused significant change in mBI score. Hence, rTMS should be used as part of standard of care in stroke rehabilitation.

**Trials**	**Sung et al. 2013**<br>**(——)**
**Inclusion criteria**	(1)Ischemic or hemorrhagic lesion within 1 hemisphere, as verified by MRI; (2) 3 to 12 months after the first-ever stroke; (3) an MRC-strength grade of ≤3/5 in the finger flexor of the paretic upper extremity at enrollment; (4) no history of seizure attack, dementia, cognitive impairment, or other neurodegenerative diseases; (5) absence of aphasia, spatial neglect, visual field deficit, or emotional problems.
**Exclusion criteria**	(——)
**Details of neurostimulation**	We performed transcranial magnetic stimulation using Magstim Rapid (Magstim) with a 70-mm figure-of-eight coil; 1-Hz rTMS trains consisting of 600 pulses at 90% of rMT were applied over the motor representation of the first dorsal interosseus in the contralesional M1. iTBS was performed at 80% of active motor threshold over the ipsilesional M1, consisting of bursts containing 3 pulses at 50 Hz repeated at 200-ms intervals for 2 seconds (ie, at 5 Hz). A 2-second train of iTBS was repeated every 10 seconds for 190 seconds and 600 pulses. We used a placebo coil (Magstim) for sham stimulation, which delivered <5% of the magnetic output with audible click-on discharge.
**Efficacy outcomes**	Wolf Motor Function Test<br>Upper extremity FuglMeyer Assessment (FMA)<br>Finger flexor MRC Scale<br>Simple reaction time task (RT)<br>Index finger tapping task (FT)<br>Bilateral corticomotor excitability,<br>Probing rMT,

	Maximal amplitude,<br>Latency of MEP,<br>Motor map area
**Safety outcomes**	All of the patients demonstrated good tolerance for the rTMS sessions, with no adverse side effects observed during the conditioning and assessments.
**Conclusions**	Our clinical trials established an extended timeframe during which conditioning could be safely continued and produced more favorable outcomes in facilitating motor performanceand ameliorating inter hemispheric imbalance than those obtained from single-course rTMS modulation alone.

Trials	Tosun et al. 2017<br>(——)
**Inclusion criteria**	(1) First-ever ischemic stroke (2) adults (aged > 18 years old), (3) stroke onset < 6 months; (4) minimum 10 degrees of volitional flexion and extension of fingers and wrist in the affected limb, (5) absence of cognitive impairment or severe aphasia that made it impossible to follow the verbal instructions during treatments or fMRI.
**Exclusion criteria**	Medically unstable or had concomitant neurodegenerative disorders, other intracranial diseases, significant medical or psychiatric illness,<br>motor apraxia, contraindications to brain stimulation or NMES (seizures, medication-resistant epilepsy, presence of a cardiac pacemaker or implanted medication pump implanted metal devices in the head), contractures of the upper limb.
**Details of neurostimulation**	NMES was delivered using a portable surface neuromuscular stimulator (BTL TENS 15 Neurostimulator, Domino SRL, Italy). The surface electrodes were arranged over the following muscles: wrist extensors and extensor digitorum communis. The stimulation pulse was a symmetrical biphasic waveform with a pulse width of 180 μs and frequency of 50 Hz. The intensity of the electrical current was adjusted to produce the extension of the wrist and fingers. Each patient received a total of 20 sessions in 4 weeks (5d/w).<br>A TAMAS device (Remed, Korea) with a figure-8 coil was used for rTMS. The participants were seated in a comfortable chair with headrest and armrests, and were told to rest both hands and upper limbs on top of their thighs. The resting motor threshold (RMT) was determined from the observation of maximum

	contralateral index finger flexion achieved in more than 5 of 10 single-pulse stimulations in TMS and TMS + NMES groups. The stimulation site was the site defined for the RMT determination. A frequency of 1 Hz, 1200 pulses with an intensity of 90% of RMT was delivered to the unaffected hemisphere for 20 min. Each patient received a total of 10 sessions over 2 weeks (5d/w) before physical therapy sessions.
**Efficacy outcomes**	BRSs, Upper Extremity Fugl-Meyer Scale (UEFM), and UE-MI, MAS score, Barthel Index (BI), BRS
**Safety outcomes**	No adverse events were reported by any of the participants, all participants completed the trial.
**Conclusions**	LF-rTMS with or without NMES seems to facilitate the motor recovery in the paretic hand of patients with acute/subacute ischemic stroke. TMS or the combination of TMS +NMES may be a promising additional therapy in upper limb motor training. Further studies with larger numbers of patients are needed to establish their effectiveness in upper limb motor rehabilitation of stroke.

**Trials**	**Wang et al. 2020 (ChiCTR-IPR-17013580)**
**Inclusion criteria**	(1) First-ever unilateral ischemic or hemorrhagic stroke in the territory of the middle cerebral artery within two weeks to 3 months of enrollment; (2) age 30-85 years; (3) initial Fugl-Meyer Motor Assessment (FMA) score <50/100 with severe motor impairment; (4) ability to provide written informed consent.
**Exclusion criteria**	(1) History of seizures; (2) severe general impairment or concomitant diseases; (3) severe aphasia or cognitive impairment that could prevent informed consent or interfere with the study's behavioral measurements; (4) any contraindications to TMS (e.g., metal implants in the brain or use of a pacemaker); (5) use of benzodiazepines or antidepressants; (6) use of muscle relaxants such as baclofen or tizanidine or injections with Botox for post-stroke spasticity.
**Details of neurostimulation**	Patients allocated to the HF group were treated as follows: 10 Hz stimulation for 1 s per session, with a 10 s interval between sessions, 100 sessions per treatment, totaling 1000 pulses at 100% resting motor threshold (rMT) over M1 of the unaffected hemisphere. The coil was positioned tangentially to the scalp, with the handle pointing posteriorly. The difference between this program and the usual high-frequency stimulation of the affected side is the change in the stimulation site to the unaffected cortex.<br>Patients in the LF group were treated as follows: 1 Hz stimulation for 10 s, with a 3 s interval between sessions, 100 sessions per treatment, totaling 1000 pulses at 100% rMT on M1 of the unaffected hemisphere.

	For sham stimulation, the coil was still centered on the same scalp position with the same parameter as the HF group, but it was rotated 90 so that scalp contact and discharging noise were similar to the 10 Hz stimulus, but minimal current flow was induced in the brain .
**Efficacy outcomes**	Fugl-Meyer assessment (FMA)<br>Barthel Index (BI)<br>motor evoked potential (MEP) amplitude and latency<br>central motor conduction time (CMCT)<br>Surface electromyography (SEMG)
**Safety outcomes**	(——)
**Conclusions**	HF-rTMS over the contralesional cortex was superior to low-frequency rTMS and sham stimulation in promoting motor recovery in patients with severe hemiplegic stroke by acting on contralesional cortex plasticity.

Trials	Watanabe et al. 2018 (UMIN000018819)
Inclusion criteria	(1) A clinical diagnosis of newly developed isolated unilateral capsular infarction confirmed by magnetic resonance imaging (MRI); (2) the possibility of starting the study intervention (rTMS) within 7 days after stroke onset; (3) age ≥ 20 years; (4) Brunnstrom Stage I–III and a modified Ashworth Scale (MAS) score of 0–2 in the affected upper limb; (5) nointravascular or other surgical intervention, (6) no use of tissue plasminogen activator; (7) no disturbance of consciousness; (8) no apparent cognitive deficit; (9) no serious general complication requiring intensive medical management; (10) no use of drugs with seizure threshold lowering potential such as antidepressants; (11) no contraindication for rTMS, such an implantable cardiac pacemaker or a history of seizures.
Exclusion criteria	(——)
Details of neurostimulation	In the iTBS group patients, iTBS (for a total of 600 pulses) was applied daily to the motor hand area of the primary motor cortex on the affected side at an intensity of 80% RMT for 10 days. In the 1-Hz stimulation group patients, low-frequency 1 Hz stimulation (for a total of 1200 pulses) was applied daily to the motor hand area of the primary motor cortex on the unaffected side at an intensity of 110% RMT for 10 days. In the sham stimulation group patients, iTBS (for a total of 600 pulses) was applied daily across a 10-cm-thick plastic board to the motor hand area of the primary motor cortex on the affected side at an intensity of 80% RMT for 10 days.
Efficacy	Fugl-Meyer Assessment (FMA),

**outcomes**	Stroke Impairment Assessment Set (SIAS),<br>Modified Ashworth Scale (MAS),<br>grip strength,<br>MEP amplitude in the FDI muscle
**Safety outcomes**	All 21 patients completed the study, and no complication occurred. No patients had symptoms suggestive of seizure.
**Conclusions**	Results of our study suggest that ipsilesional iTBS and contralesional 1-Hz stimulation applied during the acute phase of stroke are equally safe and that they enhance motor recovery in patients with post-stroke hemiparesis. However, the aspects of motor recovery enhanced by these stimulation protocols differ: ipsilesional iTBS improves movement, whereas contralesional 1-Hz stimulation reduces spasticity.

Trials	Zheng et al. 2015 (——)
**Inclusion criteria**	(1) The primary diagnosis of stroke with magnetic resonance imaging (MRI) orcomputerized tomography (CT) scans, (2) between 1 week and 4 weeks post-stroke at the time of participation, (3) an age between 40 and 80 years, (4) mild-to-moderate motor upper-limb deficits (Brunnstrom proximal upper-limb stage ≥III), (5) ability to execute at least 20 degrees of active shoulder flexion and abduction against gravity, (6) the ability to follow instructions and engage in study procedures, (7) ability to give informed consent.
**Exclusion criteria**	(1) The presence of serious diseases of the liver, kidney, hematological system or endocrine system, (2) psychiatric disorders, (3) severe cognitive impairment defined as <20 on Mini Mental State Examination, (4) orthopedic impairment, (5) limiting mobility substantially or causing pain in the affected arm, (6) visual disorders limiting the ability to comply with treatment regimen.
**Details of neurostimulation**	rTMS was applied using a 70-mm figure-of-eight coil and a Magstim Rapid 2 stimulator (Magstim Co., UK). One Hz rTMS was applied to the contralesional hemisphere over the primary motor area. The coil was placed tangentially to the scalp, with the handle pointing 45° postero-laterally. The optimal site of stimulation on the skull was defined as the location where the largest motor evoked potentials (MEPs) in unaffected first dorsal in terosseous (FDI) muscle were elicited on surface electromyography. Each rTMS session consisted of 1800 pulses, and a stimulus intensity of 90% of the resting motor threshold

	(rMT), lasting for 30 min, 6 times per week, for a total of 24 sessions.<br>For the sham stimulation, we placed the sham coil at the same place that was used for the motor cortex stimulation, and the same stimulation parameters were measured.
**Efficacy outcomes**	Upper limb score in the Fugl-Meyer assessment (U-FMA)<br>Wolf motor function test (WMFT)<br>Modified Barthel index (MBI)<br>SF-36 Quality of Life (QOL)
**Safety outcomes**	No vital adverse reactions were observed in any patients throughout the in-patient intervention. The mild adverse reactions in the experimental group included dizziness (2 cases), abnormal sleep (1 case), nausea (1 case) and nonspecific neck pain (1 case), and those in the control group included dizziness (1 case), headache (1 case), difficult concentration (1 case) and anxiety (1 case).
**Conclusions**	The results suggested the combined use of LF rTMS with VR training could effectively improve the upper limb function, the living activity, and the quality of life in patients with hemiplegia following subacute stroke, which may provide a better rehabilitation treatment for subacute stroke.

D. Catalogue of included studies

1. Dawson J, Pierce D, Dixit A, Kimberley TJ, Robertson M, Tarver B, Hilmi O, McLean J, Forbes K, Kilgard MP, Rennaker RL, Cramer SC, Walters M, Engineer N. Safety, Feasibility, and Efficacy of Vagus Nerve Stimulation Paired With Upper-Limb Rehabilitation After Ischemic Stroke. Stroke. 2016 Jan;47(1):143-50. doi: 10.1161/STROKEAHA.115.010477. Epub 2015 Dec 8. PMID: 26645257; PMCID: PMC4689175.

2. Capone F, Miccinilli S, Pellegrino G, Zollo L, Simonetti D, Bressi F, Florio L, Ranieri F, Falato E, Di Santo A, Pepe A, Guglielmelli E, Sterzi S, Di Lazzaro V. Transcutaneous Vagus Nerve Stimulation Combined with Robotic Rehabilitation Improves Upper Limb Function after Stroke. Neural Plast. 2017;2017:7876507. doi: 10.1155/2017/7876507. Epub 2017 Dec 10. PMID: 29375915; PMCID: PMC5742496.

3. Kimberley TJ, Pierce D, Prudente CN, Francisco GE, Yozbatiran N, Smith P, Tarver B, Engineer ND, Alexander Dickie D, Kline DK, Wigginton JG, Cramer SC, Dawson J. Vagus Nerve Stimulation Paired With Upper Limb Rehabilitation After Chronic Stroke. Stroke. 2018 Nov;49(11):2789-2792. doi: 10.1161/STROKEAHA.118.022279. PMID: 30355189.

4. Wu D, Ma J, Zhang L, Wang S, Tan B, Jia G. Effect and Safety of Transcutaneous Auricular Vagus Nerve Stimulation on Recovery of Upper Limb Motor Function in Subacute Ischemic Stroke Patients: A Randomized Pilot Study. Neural Plast. 2020 Aug 1;2020:8841752. doi: 10.1155/2020/8841752. PMID: 32802039; PMCID: PMC7416299.

5. Dawson J, Liu CY, Francisco GE, Cramer SC, Wolf SL, Dixit A, Alexander J, Ali R, Brown BL, Feng W, DeMark L, Hochberg LR, Kautz SA, Majid A, O'Dell MW, Pierce D, Prudente CN, Redgrave J, Turner DL, Engineer ND, Kimberley TJ. Vagus nerve stimulation paired with rehabilitation for upper limb motor function after ischaemic stroke (VNS-REHAB): a randomised, blinded, pivotal, device trial. Lancet. 2021 Apr 24;397(10284):1545-1553. doi: 10.1016/S0140-6736(21)00475-X. PMID: 33894832; PMCID: PMC8862193.

6. Amasyali SY, Yaliman A. Comparison of the effects of mirror therapy and electromyography-triggered neuromuscular stimulation on hand functions in stroke patients: a pilot study. Int J Rehabil Res. 2016 Dec;39(4):302-307. doi: 10.1097/MRR.0000000000000186. PMID: 27437723.

7. Wilson RD, Page SJ, Delahanty M, Knutson JS, Gunzler DD, Sheffler LR, Chae J. Upper-Limb Recovery After Stroke: A Randomized Controlled Trial Comparing EMG-Triggered, Cyclic, and Sensory Electrical Stimulation. Neurorehabil Neural Repair. 2016 Nov;30(10):978-987. doi: 10.1177/1545968316650278. Epub 2016 May 24. PMID: 27225977; PMCID: PMC5048487.

8. Jeon S, Kim Y, Jung K, Chung Y. The effects of electromyography-triggered electrical stimulation on shoulder subluxation, muscle activation, pain, and function in persons with stroke: A pilot study. NeuroRehabilitation. 2017;40(1):69-75. doi: 10.3233/NRE-161391. PMID: 27792019.

9. Boyaci A, Topuz O, Alkan H, Ozgen M, Sarsan A, Yildiz N, Ardic F. Comparison of the effectiveness of active and passive neuromuscular electrical stimulation of hemiplegic upper extremities: a randomized, controlled trial. Int J Rehabil Res. 2013 Dec;36(4):315-22. doi: 10.1097/MRR.0b013e328360e541. PMID: 23579106.

10. Hemmen B, Seelen HA. Effects of movement imagery and electromyography-triggered feedback on arm hand function in stroke patients in the subacute phase. Clin Rehabil. 2007 Jul;21(7):587-94. doi: 10.1177/0269215507075502. PMID: 17702700.

11. de Kroon JR, IJzerman MJ. Electrical stimulation of the upper extremity in stroke: cyclic versus EMG-triggered stimulation. Clin Rehabil. 2008 Aug;22(8):690-7. doi: 10.1177/0269215508088984. PMID: 18678569.

12. Chuang LL, Chen YL, Chen CC, Li YC, Wong AM, Hsu AL, Chang YJ. Effect of EMG-triggered neuromuscular electrical stimulation with bilateral arm training on hemiplegic shoulder pain and arm function after stroke: a randomized controlled trial. J Neuroeng Rehabil. 2017 Nov 28;14(1):122. doi: 10.1186/s12984-017-0332-0. PMID: 29183339; PMCID: PMC5706163.

13. McCabe J, Monkiewicz M, Holcomb J, Pundik S, Daly JJ. Comparison of robotics, functional electrical stimulation, and motor learning methods for treatment of persistent upper extremity dysfunction after stroke: a randomized controlled trial. Arch Phys Med Rehabil. 2015 Jun;96(6):981-90. doi: 10.1016/j.apmr.2014.10.022. Epub 2014 Nov 15. Erratum in: Arch Phys Med Rehabil. 2020 Apr;101(4):730. PMID: 25461822.

14. Shimodozono M, Noma T, Matsumoto S, Miyata R, Etoh S, Kawahira K. Repetitive facilitative exercise under continuous electrical stimulation for severe arm impairment after sub-acute stroke: a randomized controlled pilot study. Brain Inj. 2014;28(2):203-10. doi: 10.3109/02699052.2013.860472. Epub 2013 Dec 4. PMID: 24304090.

15. Shindo K, Fujiwara T, Hara J, Oba H, Hotta F, Tsuji T, Hase K, Liu M. Effectiveness of hybrid assistive neuromuscular dynamic stimulation therapy in patients with subacute stroke: a randomized controlled pilot trial. Neurorehabil Neural Repair. 2011 Nov-Dec;25(9):830-7. doi: 10.1177/1545968311408917. Epub 2011 Jun 10. PMID: 21666139.

16. Shen Y, Yin Z, Fan Y, Chen CF, Dai W, Yi W, Li Y, Zhang W, Zhang Y, Bian R, Huang Y, Machado S, Yuan TF, Shan C. Comparison of the Effects of Contralaterally Controlled Functional Electrical Stimulation and Neuromuscular Electrical Stimulation on Upper Extremity Functions in Patients with Stroke. CNS Neurol Disord Drug Targets. 2015;14(10):1260-6. doi: 10.2174/1871527315666151111122457. PMID: 26556084.

17. Zhou YX, Xia Y, Huang J, Wang HP, Bao XL, Bi ZY, Chen XB, Gao YJ, Lü XY, Wang ZG. Electromyographic bridge for promoting the recovery of hand movements in subacute stroke patients: A randomized controlled trial. J Rehabil Med. 2017 Aug 31;49(8):629-636. doi: 10.2340/16501977-2256. PMID: 28792587.

18. Zheng Y, Mao M, Cao Y, Lu X. Contralaterally controlled functional electrical stimulation improves wrist dorsiflexion and upper limb function in patients with early-phase stroke: A randomized controlled trial. J Rehabil Med. 2019 Feb 1;51(2):103-108. doi: 10.2340/16501977-2510. PMID: 30671586.

19. Knutson JS, Harley MY, Hisel TZ, Hogan SD, Maloney MM, Chae J. Contralaterally controlled functional electrical stimulation for upper extremity hemiplegia: an early-phase randomized clinical trial in subacute stroke patients. Neurorehabil Neural Repair. 2012 Mar-Apr;26(3):239-46. doi: 10.1177/1545968311419301. Epub 2011 Aug 29. PMID: 21875892; PMCID: PMC3526819.

20. Knutson JS, Gunzler DD, Wilson RD, Chae J. Contralaterally Controlled Functional Electrical Stimulation Improves Hand Dexterity in Chronic Hemiparesis: A Randomized Trial. Stroke. 2016 Oct;47(10):2596-602. doi: 10.1161/STROKEAHA.116.013791. Epub 2016 Sep 8. PMID: 27608819; PMCID: PMC5039083.

21. Knutson JS, Makowski NS, Harley MY, Hisel TZ, Gunzler DD, Wilson RD, Chae J. Adding Contralaterally Controlled Electrical Stimulation of the Triceps to Contralaterally Controlled Functional Electrical Stimulation of the Finger Extensors Reduces Upper Limb Impairment and Improves Reachable Workspace but not Dexterity: A Randomized Controlled Trial. Am J Phys Med Rehabil. 2020 Jun;99(6):514-521. doi: 10.1097/PHM.0000000000001363. PMID: 32167957.

22. Brown JA, Lutsep HL, Weinand M, Cramer SC. Motor cortex stimulation for the enhancement of recovery from stroke: a prospective, multicenter safety study. Neurosurgery. 2006 Mar;58(3):464-73. doi: 10.1227/01.NEU.0000197100.63931.04. PMID: 16528186.

23. Levy R, Ruland S, Weinand M, Lowry D, Dafer R, Bakay R. Cortical stimulation for the rehabilitation of patients with hemiparetic stroke: a multicenter feasibility study of safety and efficacy. J Neurosurg. 2008 Apr;108(4):707-14. doi: 10.3171/JNS/2008/108/4/0707. PMID: 18377250.

24. Levy RM, Harvey RL, Kissela BM, Winstein CJ, Lutsep HL, Parrish TB, Cramer SC, Venkatesan L. Epidural Electrical Stimulation for Stroke Rehabilitation: Results of the Prospective, Multicenter, Randomized, Single-Blinded Everest Trial. Neurorehabil Neural Repair. 2016 Feb;30(2):107-19. doi: 10.1177/1545968315575613. Epub 2015 Mar 6. PMID: 25748452.

25. Huang M, Harvey RL, Stoykov ME, Ruland S, Weinand M, Lowry D, Levy R. Cortical stimulation for upper limb recovery following ischemic stroke: a small phase II pilot study of a fully implanted stimulator. Top Stroke Rehabil. 2008 Mar-Apr;15(2):160-72. doi: 10.1310/tsr1502-160. PMID: 18430685.

26. Ghaziani E, Couppé C, Siersma V, Søndergaard M, Christensen H, Magnusson SP. Electrical Somatosensory Stimulation in Early Rehabilitation of Arm Paresis After Stroke: A Randomized Controlled Trial. Neurorehabil Neural Repair. 2018 Oct;32(10):899-912. doi: 10.1177/1545968318799496. Epub 2018 Sep 25. PMID: 30251591.

27. Fleming MK, Sorinola IO, Roberts-Lewis SF, Wolfe CD, Wellwood I, Newham DJ. The effect of combined somatosensory stimulation and task-specific training on upper limb function in chronic stroke: a double-blind randomized controlled trial. Neurorehabil Neural Repair. 2015 Feb;29(2):143-52. doi: 10.1177/1545968314533613. Epub 2014 May 6. PMID: 24803495.

28. Carrico C, Chelette KC 2nd, Westgate PM, Salmon-Powell E, Nichols L, Sawaki L. Randomized Trial of Peripheral Nerve Stimulation to Enhance Modified Constraint-Induced Therapy After Stroke. Am J Phys Med Rehabil. 2016 Jun;95(6):397-406. doi: 10.1097/PHM.0000000000000476. PMID: 26945226; PMCID: PMC4873453.

29. Takebayashi T, Takahashi K, Moriwaki M, Sakamoto T, Domen K. Improvement of Upper Extremity Deficit after Constraint-Induced Movement Therapy Combined with and without Preconditioning Stimulation Using Dual-hemisphere Transcranial Direct Current Stimulation and Peripheral Neuromuscular Stimulation in Chronic Stroke Patients: A Pilot Randomized Controlled Trial. Front Neurol. 2017 Oct 30;8:568. doi: 10.3389/fneur.2017.00568. PMID: 29163334; PMCID: PMC5670104.

30. Pan LH, Yang WW, Kao CL, Tsai MW, Wei SH, Fregni F, Chen VC, Chou LW. Effects of 8-week sensory electrical stimulation combined with motor training on EEG-EMG coherence and motor function in individuals with stroke. Sci Rep. 2018 Jun 15;8(1):9217. doi: 10.1038/s41598-018-27553-4. PMID: 29907780; PMCID: PMC6003966.

31. M Alwhaibi R, Mahmoud NF, M Zakaria H, M Ragab W, Al Awaji NN, Y Elzanaty M, R Elserougy H. Therapeutic Efficacy of Transcutaneous Electrical Nerve Stimulation Acupoints on Motor and Neural Recovery of the Affected Upper Extremity in Chronic Stroke: A Sham-Controlled Randomized Clinical Trial. Healthcare (Basel). 2021 May 20;9(5):614. doi: 10.3390/healthcare9050614. PMID: 34065465; PMCID: PMC8160996.

32. Jung K, Jung J, In T, Kim T, Cho HY. The influence of Task-Related Training combined with Transcutaneous Electrical Nerve Stimulation on paretic upper limb muscle activation in patients with chronic stroke. NeuroRehabilitation. 2017;40(3):315-323. doi: 10.3233/NRE-161419. PMID: 28339404.

33. Yurdakul OV, Kilicoglu MS, Rezvani A, Kucukakkas O, Eren F, Aydin T. How does cross-education affects muscles of paretic upper extremity in subacute stroke survivors? Neurol Sci. 2020 Dec;41(12):3667-3675. doi: 10.1007/s10072-020-04506-2. Epub 2020 Jun 6. PMID: 32506358.

34. de Jong LD, Dijkstra PU, Gerritsen J, Geurts AC, Postema K. Combined arm stretch positioning and neuromuscular electrical stimulation during rehabilitation does not improve range of motion, shoulder pain or function in patients after stroke: a randomised trial. J Physiother. 2013 Dec;59(4):245-54. doi: 10.1016/S1836-9553(13)70201-7. PMID: 24287218.

35. Alisar DC, Ozen S, Sozay S. Effects of Bihemispheric Transcranial Direct Current Stimulation on Upper Extremity Function in Stroke Patients: A randomized Double-Blind Sham-Controlled Study. J Stroke Cerebrovasc Dis. 2020 Jan;29(1):104454. doi: 10.1016/j.jstrokecerebrovasdis.2019.104454. Epub 2019 Nov 4. PMID: 31699572.

36. Chen SC, Yang LY, Adeel M, Lai CH, Peng CW. Transcranial electrostimulation with special waveforms enhances upper-limb motor function in patients with chronic stroke: a pilot randomized controlled trial. J Neuroeng Rehabil. 2021 Jun 30;18(1):106. doi: 10.1186/s12984-021-00901-8. PMID: 34193179; PMCID: PMC8244182.

37. Bornheim S, Croisier JL, Maquet P, Kaux JF. Transcranial direct current stimulation associated with physical-therapy in acute stroke patients - A randomized, triple blind, sham-controlled study. Brain Stimul. 2020 Mar-Apr;13(2):329-336. doi: 10.1016/j.brs.2019.10.019. Epub 2019 Oct 31. PMID: 31735645.

38. Beaulieu LD, Blanchette AK, Mercier C, Bernard-Larocque V, Milot MH. Efficacy, safety, and tolerability of bilateral transcranial direct current stimulation combined to a resistance training program in chronic stroke survivors: A double-blind, randomized, placebo-controlled pilot study. Restor Neurol Neurosci. 2019;37(4):333-346. doi: 10.3233/RNN-190908. PMID: 31227673.

39. Ang KK, Guan C, Phua KS, Wang C, Zhao L, Teo WP, Chen C, Ng YS, Chew E. Facilitating effects of transcranial direct current stimulation on motor imagery brain-computer interface with robotic feedback for stroke rehabilitation. Arch Phys Med Rehabil. 2015 Mar;96(3 Suppl):S79-87. doi: 10.1016/j.apmr.2014.08.008. PMID: 25721551.

40. Allman C, Amadi U, Winkler AM, Wilkins L, Filippini N, Kischka U, Stagg CJ, Johansen-Berg H. Ipsilesional anodal tDCS enhances the functional benefits of rehabilitation in patients after stroke. Sci Transl Med. 2016 Mar 16;8(330):330re1. doi: 10.1126/scitranslmed.aad5651. Epub 2016 Mar 16. PMID: 27089207; PMCID: PMC5388180.

41. Jin M, Zhang Z, Bai Z, Fong KNK. Timing-dependent interaction effects of tDCS with mirror therapy on upper extremity motor recovery in patients with chronic stroke: A randomized controlled pilot study. J Neurol Sci. 2019 Oct 15;405:116436. doi: 10.1016/j.jns.2019.116436. Epub 2019 Aug 29. PMID: 31493725.

42. Hesse S, Waldner A, Mehrholz J, Tomelleri C, Pohl M, Werner C. Combined transcranial direct current stimulation and robot-assisted arm training in subacute stroke patients: an exploratory, randomized multicenter trial. Neurorehabil Neural Repair. 2011 Nov-Dec;25(9):838-46. doi: 10.1177/1545968311413906. Epub 2011 Aug 8. PMID: 21825004.

43. Lindenberg R, Renga V, Zhu LL, Nair D, Schlaug G. Bihemispheric brain stimulation facilitates motor recovery in chronic stroke patients. Neurology. 2010 Dec 14;75(24):2176-84. doi: 10.1212/WNL.0b013e318202013a. Epub 2010 Nov 10. PMID: 21068427; PMCID: PMC3013585.

44. Liao WW, Chiang WC, Lin KC, Wu CY, Liu CT, Hsieh YW, Lin YC, Chen CL. Timing-dependent effects of transcranial direct current stimulation with mirror therapy on daily function and motor control in chronic stroke: a randomized controlled pilot study. J Neuroeng Rehabil. 2020 Jul 20;17(1):101. doi: 10.1186/s12984-020-00722-1. PMID: 32690032; PMCID: PMC7370428.

45. Lee SJ, Chun MH. Combination transcranial direct current stimulation and virtual reality therapy for upper extremity training in patients with subacute stroke. Arch Phys Med Rehabil. 2014 Mar;95(3):431-8. doi: 10.1016/j.apmr.2013.10.027. Epub 2013 Nov 14. PMID: 24239790.

46. Gong Y, Long XM, Xu Y, Cai XY, Ye M. Effects of repetitive transcranial magnetic stimulation combined with transcranial direct current stimulation on motor function and cortex excitability in subacute stroke patients: A randomized controlled trial. Clin Rehabil. 2021 May;35(5):718-727. doi: 10.1177/0269215520972940. Epub 2020 Nov 23. PMID: 33222502.

47. Fusco A, Assenza F, Iosa M, Izzo S, Altavilla R, Paolucci S, Vernieri F. The ineffective role of cathodal tDCS in enhancing the functional motor outcomes in early phase of stroke rehabilitation: an experimental trial. Biomed Res Int. 2014;2014:547290. doi: 10.1155/2014/547290. Epub 2014 May 5. PMID: 24895588; PMCID: PMC4026962.

48. Edwards DJ, Cortes M, Rykman-Peltz A, Chang J, Elder J, Thickbroom G, Mariman JJ, Gerber LM, Oromendia C, Krebs HI, Fregni F, Volpe BT, Pascual-Leone A. Clinical improvement with intensive robot-assisted arm training in chronic stroke is unchanged by supplementary tDCS. Restor Neurol Neurosci.

2019;37(2):167-180. doi: 10.3233/RNN-180869. PMID: 30932903.

49. Kim SH. Effects of Dual Transcranial Direct Current Stimulation and Modified Constraint-Induced Movement Therapy to Improve Upper-Limb Function after Stroke: A Double-Blinded, Pilot Randomized Controlled Trial. J Stroke Cerebrovasc Dis. 2021 Sep;30(9):105928. doi: 10.1016/j.jstrokecerebrovasdis.2021.105928. Epub 2021 Jul 10. PMID: 34256199.

50. Mazzoleni S, Tran VD, Dario P, Posteraro F. Effects of Transcranial Direct Current Stimulation (tDCS) Combined With Wrist Robot-Assisted Rehabilitation on Motor Recovery in Subacute Stroke Patients: A Randomized Controlled Trial. IEEE Trans Neural Syst Rehabil Eng. 2019 Jul;27(7):1458-1466. doi: 10.1109/TNSRE.2019.2920576. Epub 2019 Jun 3. PMID: 31170077.

51. Yao X, Cui L, Wang J, Feng W, Bao Y, Xie Q. Effects of transcranial direct current stimulation with virtual reality on upper limb function in patients with ischemic stroke: a randomized controlled trial. J Neuroeng Rehabil. 2020 Jun 15;17(1):73. doi: 10.1186/s12984-020-00699-x. PMID: 32539812; PMCID: PMC7296643.

52. Viana RT, Laurentino GE, Souza RJ, Fonseca JB, Silva Filho EM, Dias SN, Teixeira-Salmela LF, Monte-Silva KK. Effects of the addition of transcranial direct current stimulation to virtual reality therapy after stroke: a pilot randomized controlled trial. NeuroRehabilitation. 2014;34(3):437-46. doi: 10.3233/NRE-141065. PMID: 24473248.

53. Triccas LT, Burridge JH, Hughes A, Verheyden G, Desikan M, Rothwell J. A double-blinded randomised controlled trial exploring the effect of anodal transcranial direct current stimulation and uni-lateral robot therapy for the impaired upper limb in sub-acute and chronic stroke. NeuroRehabilitation. 2015;37(2):181-91. doi: 10.3233/NRE-151251. PMID: 26484510.

54. Shaheiwola N, Zhang B, Jia J, Zhang D. Using tDCS as an Add-On Treatment Prior to FES Therapy in Improving Upper Limb Function in Severe Chronic Stroke Patients: A Randomized Controlled Study. Front Hum Neurosci. 2018 Jun 19;12:233. doi: 10.3389/fnhum.2018.00233. PMID: 29970994; PMCID: PMC6018756.

55. Salazar AP, Cimolin V, Schifino GP, Rech KD, Marchese RR, Pagnussat AS. Bi-cephalic transcranial direct current stimulation combined with functional electrical stimulation for upper-limb stroke rehabilitation: A double-blind randomized controlled trial. Ann Phys Rehabil Med. 2020 Jan;63(1):4-11. doi: 10.1016/j.rehab.2019.05.004. Epub 2019 May 31. PMID: 31158553.

56. Rocha S, Silva E, Foerster Á, Wiesiolek C, Chagas AP, Machado G, Baltar A, Monte-Silva K. The impact of transcranial direct current stimulation (tDCS) combined with modified constraint-induced movement therapy (mCIMT) on upper limb function in chronic stroke: a double-blind randomized controlled trial. Disabil Rehabil. 2016;38(7):653-60. doi: 10.3109/09638288.2015.1055382. Epub 2015 Jun 10. PMID: 26061222.

57. Pavlova EL, Lindberg P, Khan A, Ruschkowski S, Nitsche MA, Borg J. Transcranial direct current stimulation combined with visuo-motor training as treatment for chronic stroke patients. Restor Neurol Neurosci. 2017;35(3):307-317. doi: 10.3233/RNN-160706. PMID: 28506002.

58. Gottlieb A, Boltzmann M, Schmidt SB, Gutenbrunner C, Krauss JK, Stangel M, Höglinger GU, Wallesch CW, Rollnik JD. Treatment of upper limb spasticity with inhibitory repetitive transcranial magnetic stimulation: A randomized placebo-controlled trial. NeuroRehabilitation. 2021;49(3):425-434. doi: 10.3233/NRE-210088. PMID: 34542038.

59. Chiu D, McCane CD, Lee J, John B, Nguyen L, Butler K, Gadhia R, Misra V, Volpi JJ, Verma A, Helekar SA. Multifocal transcranial stimulation in chronic ischemic stroke: A phase 1/2a randomized trial. J Stroke Cerebrovasc Dis. 2020 Jun;29(6):104816. doi: 10.1016/j.jstrokecerebrovasdis.2020.104816. Epub 2020 Apr 19. PMID: 32321651.

60. Chen YH, Chen CL, Huang YZ, Chen HC, Chen CY, Wu CY, Lin KC. Augmented efficacy of intermittent theta burst stimulation on the virtual reality-based cycling training for upper limb function in patients with stroke: a double-blinded, randomized controlled trial. J Neuroeng Rehabil. 2021 May 31;18(1):91. doi: 10.1186/s12984-021-00885-5. PMID: 34059090; PMCID: PMC8166006.

61. Chang WH, Kim YH, Bang OY, Kim ST, Park YH, Lee PK. Long-term effects of rTMS on motor recovery in patients after subacute stroke. J Rehabil Med. 2010 Sep;42(8):758-64. doi: 10.2340/16501977-0590. PMID: 20809058.

62. Chen X, Liu X, Cui Y, Xu G, Liu L, Zhang X, Jiang K, Li Z. Efficacy of functional magnetic stimulation in improving upper extremity function after stroke: a randomized, single-blind, controlled study. J Int Med Res. 2020 Jun;48(6):300060520927881. doi: 10.1177/0300060520927881. PMID: 32495667; PMCID: PMC7273768.

63. Obayashi S, Takahashi R. Repetitive peripheral magnetic stimulation improves severe upper limb paresis in early acute phase stroke survivors. NeuroRehabilitation. 2020;46(4):569-575. doi: 10.3233/NRE-203085. PMID: 32508342; PMCID: PMC7458515.

64. Krewer C, Hartl S, Müller F, Koenig E. Effects of repetitive peripheral magnetic stimulation on upper-limb spasticity and impairment in patients with spastic

hemiparesis: a randomized, double-blind, sham-controlled study. Arch Phys Med Rehabil. 2014 Jun;95(6):1039-47. doi: 10.1016/j.apmr.2014.02.003. Epub 2014 Feb 19. PMID: 24561057.

65. Chen YJ, Huang YZ, Chen CY, Chen CL, Chen HC, Wu CY, Lin KC, Chang TL. Intermittent theta burst stimulation enhances upper limb motor function in patients with chronic stroke: a pilot randomized controlled trial. BMC Neurol. 2019 Apr 25;19(1):69. doi: 10.1186/s12883-019-1302-x. PMID: 31023258; PMCID: PMC6485156.

66. Du J, Tian L, Liu W, Hu J, Xu G, Ma M, Fan X, Ye R, Jiang Y, Yin Q, Zhu W, Xiong Y, Yang F, Liu X. Effects of repetitive transcranial magnetic stimulation on motor recovery and motor cortex excitability in patients with stroke: a randomized controlled trial. Eur J Neurol. 2016 Nov;23(11):1666-1672. doi: 10.1111/ene.13105. Epub 2016 Jul 18. PMID: 27425785.

67. Doris Miu KY, Kok C, Leung SS, Chan EYL, Wong E. Comparison of Repetitive Transcranial Magnetic Stimulation and Transcranial Direct Current Stimulation on Upper Limb Recovery Among Patients With Recent Stroke. Ann Rehabil Med. 2020 Dec;44(6):428-437. doi: 10.5535/arm.20093. Epub 2020 Dec 31. PMID: 33440091; PMCID: PMC7808788.

68. Guan YZ, Li J, Zhang XW, Wu S, Du H, Cui LY, Zhang WH. Effectiveness of repetitive transcranial magnetic stimulation (rTMS) after acute stroke: A one-year longitudinal randomized trial. CNS Neurosci Ther. 2017 Dec;23(12):940-946. doi: 10.1111/cns.12762. Epub 2017 Oct 2. PMID: 28971620; PMCID: PMC6492666.

69. Barros Galvão SC, Borba Costa dos Santos R, Borba dos Santos P, Cabral ME, Monte-Silva K. Efficacy of coupling repetitive transcranial magnetic stimulation and physical therapy to reduce upper-limb spasticity in patients with stroke: a randomized controlled trial. Arch Phys Med Rehabil. 2014 Feb;95(2):222-9. doi: 10.1016/j.apmr.2013.10.023. Epub 2013 Nov 12. PMID: 24239881.

70. Hsu YF, Huang YZ, Lin YY, Tang CW, Liao KK, Lee PL, Tsai YA, Cheng HL, Cheng H, Chern CM, Lee IH. Intermittent theta burst stimulation over ipsilesional primary motor cortex of subacute ischemic stroke patients: a pilot study. Brain Stimul. 2013 Mar;6(2):166-74. doi: 10.1016/j.brs.2012.04.007. Epub 2012 May 12. PMID: 22659021.

71. Harvey RL, Edwards D, Dunning K, Fregni F, Stein J, Laine J, Rogers LM, Vox F, Durand-Sanchez A, Bockbrader M, Goldstein LB, Francisco GE, Kinney CL, Liu CY; NICHE Trial Investigators *. Randomized Sham-Controlled Trial of Navigated Repetitive Transcranial Magnetic Stimulation for Motor Recovery in Stroke. Stroke. 2018 Sep;49(9):2138-2146. doi: 10.1161/STROKEAHA.117.020607. PMID: 30354990.

72. Kuzu Ö, Adiguzel E, Kesikburun S, Yaşar E, Yılmaz B. The Effect of Sham Controlled Continuous Theta Burst Stimulation and Low Frequency Repetitive Transcranial Magnetic Stimulation on Upper Extremity Spasticity and Functional Recovery in Chronic Ischemic Stroke Patients. J Stroke Cerebrovasc Dis. 2021 Jul;30(7):105795. doi: 10.1016/j.jstrokecerebrovasdis.2021.105795. Epub 2021 Apr 20. PMID: 33887662.

73. Kim JH, Han JY, Song MK, Park GC, Lee JS. Synergistic Effects of Scalp Acupuncture and Repetitive Transcranial Magnetic Stimulation on Cerebral Infarction: A Randomized Controlled Pilot Trial. Brain Sci. 2020 Feb 7;10(2):87. doi: 10.3390/brainsci10020087. PMID: 32046150; PMCID: PMC7071610.

74. Kim WS, Kwon BS, Seo HG, Park J, Paik NJ. Low-Frequency Repetitive Transcranial Magnetic Stimulation Over Contralesional Motor Cortex for Motor Recovery in Subacute Ischemic Stroke: A Randomized Sham-Controlled Trial. Neurorehabil Neural Repair. 2020 Sep;34(9):856-867. doi: 10.1177/1545968320948610. Epub 2020 Aug 18. PMID: 32807013.

75. Matsuura A, Onoda K, Oguro H, Yamaguchi S. Magnetic stimulation and movement-related cortical activity for acute stroke with hemiparesis. Eur J Neurol. 2015 Dec;22(12):1526-32. doi: 10.1111/ene.12776. Epub 2015 Jul 15. PMID: 26177235.

76. Long H, Wang H, Zhao C, Duan Q, Feng F, Hui N, Mao L, Liu H, Mou X, Yuan H. Effects of combining high- and low-frequency repetitive transcranial magnetic stimulation on upper limb hemiparesis in the early phase of stroke. Restor Neurol Neurosci. 2018;36(1):21-30. doi: 10.3233/RNN-170733. PMID: 29439359.

77. Rose DK, Patten C, McGuirk TE, Lu X, Triggs WJ. Does inhibitory repetitive transcranial magnetic stimulation augment functional task practice to improve arm recovery in chronic stroke? Stroke Res Treat. 2014;2014:305236. doi: 10.1155/2014/305236. Epub 2014 Aug 13. PMID: 25197611; PMCID: PMC4147256.

78. Bonin Pinto C, Morales-Quezada L, de Toledo Piza PV, Zeng D, Saleh Vélez FG, Ferreira IS, Lucena PH, Duarte D, Lopes F, El-Hagrassy MM, Rizzo LV, Camargo EC, Lin DJ, Mazwi N, Wang QM, Black-Schaffer R, Fregni F. Combining Fluoxetine and rTMS in Poststroke Motor Recovery: A Placebo-Controlled Double-Blind Randomized Phase 2 Clinical Trial. Neurorehabil Neural Repair. 2019 Aug;33(8):643-655. doi: 10.1177/1545968319860483. Epub 2019 Jul 9. PMID: 31286828; PMCID: PMC6688938.

79. Meng Y, Zhang D, Hai H, Zhao YY, Ma YW. Efficacy of coupling intermittent theta-burst stimulation and 1 Hz repetitive transcranial magnetic stimulation to enhance upper limb motor recovery in subacute stroke patients: A randomized controlled trial. Restor Neurol Neurosci. 2020;38(1):109-118. doi:

10.3233/RNN-190953. PMID: 32039879.

80. Zheng CJ, Liao WJ, Xia WG. Effect of combined low-frequency repetitive transcranial magnetic stimulation and virtual reality training on upper limb function in subacute stroke: a double-blind randomized controlled trail. J Huazhong Univ Sci Technolog Med Sci. 2015 Apr;35(2):248-254. doi: 10.1007/s11596-015-1419-0. Epub 2015 Apr 16. PMID: 25877360.

81. Watanabe K, Kudo Y, Sugawara E, Nakamizo T, Amari K, Takahashi K, Tanaka O, Endo M, Hayakawa Y, Johkura K. Comparative study of ipsilesional and contralesional repetitive transcranial magnetic stimulations for acute infarction. J Neurol Sci. 2018 Jan 15;384:10-14. doi: 10.1016/j.jns.2017.11.001. Epub 2017 Nov 9. PMID: 29249365.

82. Wang Q, Zhang D, Zhao YY, Hai H, Ma YW. Effects of high-frequency repetitive transcranial magnetic stimulation over the contralesional motor cortex on motor recovery in severe hemiplegic stroke: A randomized clinical trial. Brain Stimul. 2020 Jul-Aug;13(4):979-986. doi: 10.1016/j.brs.2020.03.020. Epub 2020 Apr 2. PMID: 32380449.

83. Tosun A, Türe S, Askin A, Yardimci EU, Demirdal SU, Kurt Incesu T, Tosun O, Kocyigit H, Akhan G, Gelal FM. Effects of low-frequency repetitive transcranial magnetic stimulation and neuromuscular electrical stimulation on upper extremity motor recovery in the early period after stroke: a preliminary study. Top Stroke Rehabil. 2017 Jul;24(5):361-367. doi: 10.1080/10749357.2017.1305644. Epub 2017 Mar 22. PMID: 28327054.

84. Sung WH, Wang CP, Chou CL, Chen YC, Chang YC, Tsai PY. Efficacy of coupling inhibitory and facilitatory repetitive transcranial magnetic stimulation to enhance motor recovery in hemiplegic stroke patients. Stroke. 2013 May;44(5):1375-82. doi: 10.1161/STROKEAHA.111.000522. Epub 2013 Mar 26. PMID: 23532011.

85. Sharma H, Vishnu VY, Kumar N, Sreenivas V, Rajeswari MR, Bhatia R, Sharma R, Srivastava MVP. Efficacy of Low-Frequency Repetitive Transcranial Magnetic Stimulation in Ischemic Stroke: A Double-Blind Randomized Controlled Trial. Arch Rehabil Res Clin Transl. 2020 Jan 11;2(1):100039. doi: 10.1016/j.arrct.2020.100039. Erratum in: Arch Rehabil Res Clin Transl. 2020 Jun 20;2(4):100069. PMID: 33543068; PMCID: PMC7853333.

86. Seniów J, Bilik M, Leśniak M, Waldowski K, Iwański S, Członkowska A. Transcranial magnetic stimulation combined with physiotherapy in rehabilitation of poststroke hemiparesis: a randomized, double-blind, placebo-controlled study. Neurorehabil Neural Repair. 2012 Nov-Dec;26(9):1072-9. doi: 10.1177/1545968312445635. Epub 2012 May 15. PMID: 22588639.

87. Abo M, Kakuda W, Momosaki R, Harashima H, Kojima M, Watanabe S, Sato T, Yokoi A, Umemori T, Sasanuma J. Randomized, multicenter, comparative study of NEURO versus CIMT in poststroke patients with upper limb hemiparesis: the NEURO-VERIFY Study. Int J Stroke. 2014 Jul;9(5):607-12. doi: 10.1111/ijs.12100. Epub 2013 Sep 9. PMID: 24015934.

88. Chan MK, Tong RK, Chung KY. Bilateral upper limb training with functional electric stimulation in patients with chronic stroke. Neurorehabil Neural Repair. 2009 May;23(4):357-65. doi: 10.1177/1545968308326428. Epub 2008 Dec 12. PMID: 19074684.

E. Pair-wise forest plot

FMA-UE LFU

Study or Subgroup	Group 1 Mean	Group 1 SD	Group 1 Total	Group 2 Mean	Group 2 SD	Group 2 Total	Weight	Mean Difference IV, Random, 95% CI
**1.1.1 VNS vs. control**								
VNS_2016 Dawson	8.7	5.8	9	3	6.1	11	13.5%	5.70 [0.47, 10.93]
VNS_2018 Kimberley	9.5	9.6	8	3.8	9.4	9	4.5%	5.70 [-3.35, 14.75]
VNS_2021 Dawson	5.8	6	53	2.8	5.2	55	82.0%	3.00 [0.88, 5.12]
**Subtotal (95% CI)**			**70**			**75**	**100.0%**	**3.49 [1.56, 5.41]**
Heterogeneity: Tau² = 0.00; Chi² = 1.12, df = 2 (P = 0.57); I² = 0%								
Test for overall effect: Z = 3.56 (P = 0.0004)								
**1.1.2 MCS vs. control**								
MCS_2006 Brown	10	8.9	4	1.9	7.75	4	3.8%	8.10 [-3.47, 19.67]
MCS_2008 Huang	4.54	6.08	12	-0.08	5.4	12	19.6%	4.62 [0.02, 9.22]
MCS_2008 Levy	5.5	4.4	12	1.9	4.4	12	28.8%	3.60 [0.08, 7.12]
MCS_2016 Levy	4.6	6.3	94	3.8	7.1	58	47.9%	0.80 [-1.43, 3.03]
**Subtotal (95% CI)**			**122**			**86**	**100.0%**	**2.63 [0.32, 4.95]**
Heterogeneity: Tau² = 1.62; Chi² = 4.19, df = 3 (P = 0.24); I² = 28%								
Test for overall effect: Z = 2.23 (P = 0.03)								
**1.1.3 SES vs. control**								
SES_2015 Fleming	2.8	4.7	16	1.4	6.8	17	5.8%	1.40 [-2.57, 5.37]
SES_2016 Carrico	12.5	6.28	10	6.58	7.17	9	2.6%	5.92 [-0.17, 12.01]
SES_2017 Jung	6	1.94	23	3.4	1.75	23	40.5%	2.60 [1.53, 3.67]
SES_2018 Ghaziani	17.5	4.3	53	16.6	4	49	25.4%	0.90 [-0.71, 2.51]
SES_2018 Pan	5.09	5.71	6	3.83	5.58	6	2.4%	1.26 [-5.13, 7.65]
SES_2021 Alwhaibi	3.2	2.39	20	2.4	3.1	20	23.3%	0.80 [-0.92, 2.52]
**Subtotal (95% CI)**			**128**			**124**	**100.0%**	**1.73 [0.73, 2.73]**
Heterogeneity: Tau² = 0.34; Chi² = 6.47, df = 5 (P = 0.26); I² = 23%								
Test for overall effect: Z = 3.40 (P = 0.0007)								
**1.1.4 ENMES vs. control**								
NMES_2016 Amasyali	13.71	16.48	7	11.55	16.61	8	100.0%	2.16 [-14.62, 18.94]
**Subtotal (95% CI)**			**7**			**8**	**100.0%**	**2.16 [-14.62, 18.94]**
Heterogeneity: Not applicable								
Test for overall effect: Z = 0.25 (P = 0.80)								
**1.1.5 ENMES vs. SES**								
NMES vs. SES_2013 Boyaci	8	15.25	11	1	19.63	10	11.9%	7.00 [-8.14, 22.14]
NMES vs. SES_2016 Wilson	9.7	14.67	41	10.8	14.89	42	67.3%	-1.10 [-7.46, 5.26]
NMES vs. SES_2017 Chuang	4.37	18.8	19	1.31	17.07	19	20.9%	3.06 [-8.36, 14.48]
**Subtotal (95% CI)**			**71**			**71**	**100.0%**	**0.73 [-4.49, 5.95]**
Heterogeneity: Tau² = 0.00; Chi² = 1.14, df = 2 (P = 0.57); I² = 0%								
Test for overall effect: Z = 0.27 (P = 0.78)								
**1.1.6 cMNES vs. control**								
NMES_2011 Shindo	12.2	5.3	10	5.5	6	10	34.9%	6.70 [1.74, 11.66]
NMES_2013 Jong	12	17.79	23	7	16.19	23	16.2%	5.00 [-4.83, 14.83]
NMES_2013 Shimodozono	18.5	11.9	9	9.7	8.41	9	17.0%	8.80 [-0.72, 18.32]
NMES_2015 McCabe	8.8	7.3	14	9.9	7.37	13	31.8%	-1.10 [-6.64, 4.44]
**Subtotal (95% CI)**			**56**			**55**	**100.0%**	**4.30 [-0.38, 8.97]**
Heterogeneity: Tau² = 9.88; Chi² = 5.41, df = 3 (P = 0.14); I² = 45%								
Test for overall effect: Z = 1.80 (P = 0.07)								
**1.1.7 atDCS vs. control**								
tDCS_2011 Hesse	15.4	16.73	32	14.3	15.38	32	10.0%	1.10 [-6.77, 8.97]
tDCS_2014 Viana	9.3	5.7	10	7.5	7.1	10	14.3%	1.80 [-3.84, 7.44]
tDCS_2015 Rocha	13.2	3.3	7	5.6	11	7	9.0%	7.60 [-0.91, 16.11]
tDCS_2015 Triccas	7.18	16.34	12	7.09	16.3	11	4.6%	0.09 [-13.26, 13.44]
tDCS_2016 Allman	9.28	15.18	11	6.73	16.86	13	4.9%	2.55 [-10.27, 15.37]
tDCS_2017 Pavlova	1.83	18.29	5	2.32	18.27	6	1.9%	-0.49 [-22.19, 21.21]
tDCS_2019 Bornheim	18.66	13.64	25	7	14.8	25	9.9%	11.66 [3.77, 19.55]
tDCS_2019 Edwards	6.7	4.4	41	8.6	5.1	41	24.0%	-1.90 [-3.96, 0.16]
tDCS_2019 Mazzoleni	12	8.5	20	15.74	13.75	19	11.1%	-3.74 [-10.96, 3.48]
tDCS_2020 Liao	5.49	12.25	23	3.75	9.87	11	10.3%	1.74 [-5.95, 9.43]
**Subtotal (95% CI)**			**186**			**175**	**100.0%**	**1.64 [-1.50, 4.77]**
Heterogeneity: Tau² = 9.53; Chi² = 16.44, df = 9 (P = 0.06); I² = 45%								
Test for overall effect: Z = 1.02 (P = 0.31)								
**1.1.8 ctDCS vs. control**								
tDCS_2011 Hesse	15.6	13.13	32	14.3	15.38	32	25.0%	1.30 [-5.71, 8.31]
tDCS_2014 Fusco	8.34	28.8	7	9.904	23.04	7	1.6%	-1.56 [-28.89, 25.76]
tDCS_2014 Lee	10.3	5	20	8.7	10.8	20	45.1%	1.60 [-3.62, 6.82]
tDCS_2015 Rocha	7.6	3.8	7	5.6	11	7	16.5%	2.00 [-6.62, 10.62]
tDCS_2020 Yao	10.1	17.23	20	6.4	15.83	20	11.7%	3.70 [-6.55, 13.95]
**Subtotal (95% CI)**			**86**			**86**	**100.0%**	**1.78 [-1.72, 5.29]**
Heterogeneity: Tau² = 0.00; Chi² = 0.22, df = 4 (P = 0.99); I² = 0%								
Test for overall effect: Z = 1.00 (P = 0.32)								
**1.1.9 dtDCS vs. control**								
tDCS_2010 Lindenberg	6.1	12.5	10	1.1	11.6	10	2.2%	5.00 [-5.57, 15.57]
tDCS_2015 Ang	5	4.4	10	5.4	5.7	9	11.5%	-0.40 [-5.02, 4.22]
tDCS_2018 Shaheiwola	8.5	9.35	15	4.6	13.04	15	3.7%	3.90 [-4.22, 12.02]
tDCS_2019 Beaulieu	5.71	4.54	7	2.71	3.2	7	14.5%	3.00 [-1.11, 7.11]
tDCS_2019 Jin	1.55	17.13	20	0.9	18.24	10	1.3%	0.65 [-12.92, 14.22]
tDCS_2019 Salazar	1.4	9.14	15	1.78	10.36	15	5.0%	-0.38 [-7.37, 6.61]
tDCS_2020 Alisar	9.12	19.38	16	1.74	23.55	16	1.1%	7.38 [-7.56, 22.32]
tDCS_2021 Kim	4.4	2.82	15	3.14	2.81	15	60.6%	1.26 [-0.75, 3.27]
**Subtotal (95% CI)**			**108**			**97**	**100.0%**	**1.48 [-0.09, 3.05]**
Heterogeneity: Tau² = 0.00; Chi² = 2.86, df = 7 (P = 0.90); I² = 0%								
Test for overall effect: Z = 1.85 (P = 0.06)								
**1.1.10 ENMES vs. cNMES**								
NMES vs. SES_2013 Boyaci	5	14.05	10	8	15.25	11	8.0%	-3.00 [-15.53, 9.53]
NMES vs. SES_2016 Wilson	9.7	14.67	41	11.8	13.74	39	32.6%	-2.10 [-8.33, 4.13]
NMES_2008 Kroon	5	5.9	10	2.8	6	11	48.7%	2.20 [-2.89, 7.29]
NMES_2016 Jeon	1.8	12.11	10	3.1	12.74	10	10.6%	-1.30 [-12.19, 9.59]
**Subtotal (95% CI)**			**71**			**71**	**100.0%**	**0.01 [-3.55, 3.56]**
Heterogeneity: Tau² = 0.00; Chi² = 1.43, df = 3 (P = 0.70); I² = 0%								
Test for overall effect: Z = 0.00 (P = 1.00)								

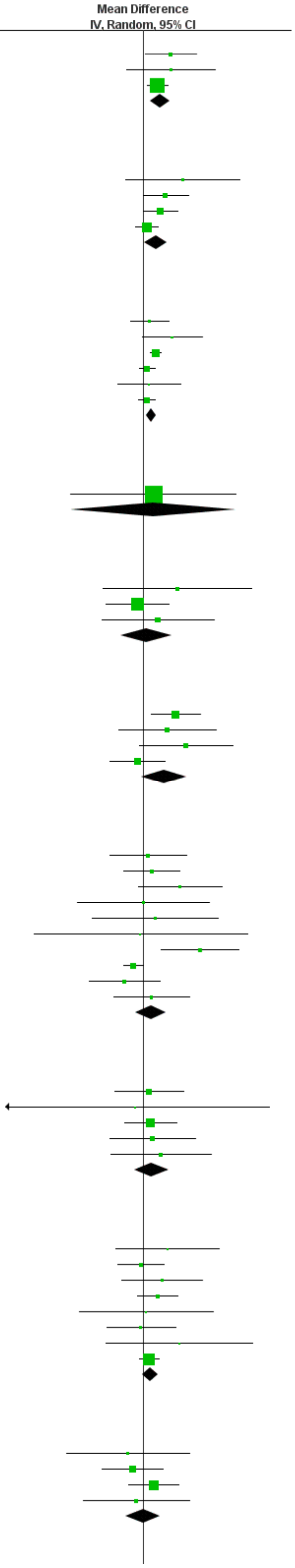

**1.1.11 FES vs. cNMES**								
FES vs. NMES_2011 Knutson	13.6	7.57	9	8.5	7.22	8	9.3%	5.10 [-1.93, 12.13]
FES vs. NMES_2014 Shen	7.6	1.4	30	4.9	1.4	30	23.8%	2.70 [1.99, 3.41]
FES vs. NMES_2016 Knutson	2.4	5.27	32	2.2	4.69	40	20.6%	0.20 [-2.13, 2.53]
FES vs. NMES_2017 Zhou	21.1	7.54	18	12.4	7.54	18	13.5%	8.70 [3.77, 13.63]
FES vs. NMES_2019 Zheng	16.38	4.06	21	8.1	4.53	20	19.7%	8.28 [5.64, 10.92]
FES vs. NMES_2020 Knutson	11	10.87	21	10.7	2.99	8	13.1%	0.30 [-4.79, 5.39]
**Subtotal (95% CI)**			**131**			**124**	**100.0%**	**4.01 [1.28, 6.73]**
Heterogeneity: $Tau^2 = 7.97$; $Chi^2 = 28.13$, df = 5 ($P < 0.0001$); $I^2 = 82\%$								
Test for overall effect: Z = 2.88 (P = 0.004)								
**1.1.12 cNMES vs. SES**								
NMES vs. SES_2013 Boyaci	5	14.05	10	1	19.63	10	8.2%	4.00 [-10.96, 18.96]
NMES vs. SES_2016 Wilson	11.8	13.74	39	10.8	14.89	42	47.3%	1.00 [-5.23, 7.23]
NMES vs.SES_2020 Yurdakul	6.67	11	15	4.8	6.34	15	44.5%	1.87 [-4.56, 8.30]
**Subtotal (95% CI)**			**64**			**67**	**100.0%**	**1.63 [-2.65, 5.92]**
Heterogeneity: $Tau^2 = 0.00$; $Chi^2 = 0.14$, df = 2 (P = 0.93); $I^2 = 0\%$								
Test for overall effect: Z = 0.75 (P = 0.46)								
**1.1.13 LFrTMS vs. control**								
rTMS vs. cTBS vs. R_2021 Kuzu	7.71	3.04	7	1.17	1.47	6	8.6%	6.54 [4.00, 9.08]
rTMS vs. iTBS vs, R_2018 Watanabe	25.11	9.92	7	13.86	4.39	6	3.0%	11.25 [3.10, 19.40]
rTMS vs. iTBS vs. R_2020 Meng	4.02	6.41	10	2.14	7.3	8	4.1%	1.88 [-4.55, 8.31]
rTMS vs. iTBS vs. rTMS+iTBS vs. R_2013 Sung	2.6	15.36	13	1.4	12.42	14	2.0%	1.20 [-9.39, 11.79]
rTMS vs. NMES+rTMS vs. R_2017 Tosun	22.2	13.41	9	4.7	19.11	9	1.0%	17.50 [2.25, 32.75]
rTMS_2012 Seniow	11.6	12.29	20	12.2	15	20	2.8%	-0.60 [-9.10, 7.90]
rTMS_2013 Abo	5.39	4.28	44	3.09	4.5	22	8.9%	2.30 [0.03, 4.57]
rTMS_2014 Galvao	4.8	4	10	7.5	8.4	10	4.6%	-2.70 [-8.47, 3.07]
rTMS_2014 Rose	4.6	4.5	9	3.9	4.74	10	6.4%	0.70 [-3.46, 4.86]
rTMS_2015 Matsuura	8.18	9.44	10	1.82	9.72	10	2.8%	6.36 [-2.04, 14.76]
rTMS_2015 Zheng	28.5	9.52	58	18.51	9.94	54	7.1%	9.99 [6.38, 13.60]
rTMS_2016 Du	22.56	12.73	23	14.36	12.19	23	3.5%	8.20 [1.00, 15.40]
rTMS_2018 Harvey	8.1	7.4	132	8.5	8.7	67	8.7%	-0.40 [-2.84, 2.04]
rTMS_2018 Long	6.29	5.2	21	4.85	3.21	20	8.4%	1.44 [-1.19, 4.07]
rTMS_2019 Pinto	8.11	11.07	9	6.87	6.77	10	2.8%	1.24 [-7.12, 9.60]
rTMS_2020 Gong	4.06	1.39	15	3.2	1.43	15	10.3%	0.86 [-0.15, 1.87]
rTMS_2020 Kim	13.18	15.72	8	5.83	11.3	12	1.5%	7.35 [-5.28, 19.98]
rTMS_2020 Sharma	3.73	15.69	47	2.21	16.85	49	4.0%	1.52 [-4.99, 8.03]
rTMS_2020 Wang	4.7	12.27	15	2.8	7.53	15	3.5%	1.90 [-5.39, 9.19]
rTMS_2020 Won-Seok	14.9	10.8	36	13.8	12.1	37	5.1%	1.10 [-4.16, 6.36]
rTMS_2021 Gottlieb	6.43	23.92	14	3.5	22.38	14	0.8%	2.93 [-14.23, 20.09]
**Subtotal (95% CI)**			**517**			**431**	**100.0%**	**2.99 [1.34, 4.63]**
Heterogeneity: $Tau^2 = 6.54$; $Chi^2 = 56.91$, df = 20 ($P < 0.0001$); $I^2 = 65\%$								
Test for overall effect: Z = 3.56 (P = 0.0004)								
**1.1.14 LFrTMS vs. LFrTMS+atDCS**								
rTMS_2020 Gong	4.06	1.39	15	4.4	2.67	15	100.0%	-0.34 [-1.86, 1.18]
**Subtotal (95% CI)**			**15**			**15**	**100.0%**	**-0.34 [-1.86, 1.18]**
Heterogeneity: Not applicable								
Test for overall effect: Z = 0.44 (P = 0.66)								
**1.1.15 LFrTMS vs. LFrTMS+ctDCS**								
rTMS_2020 Gong	4.06	1.39	15	4.07	1.73	15	100.0%	-0.01 [-1.13, 1.11]
**Subtotal (95% CI)**			**15**			**15**	**100.0%**	**-0.01 [-1.13, 1.11]**
Heterogeneity: Not applicable								
Test for overall effect: Z = 0.02 (P = 0.99)								
**1.1.16 LFrTMS+atDCS vs. control**								
rTMS_2020 Gong	4.4	2.67	15	3.2	1.43	15	100.0%	1.20 [-0.33, 2.73]
**Subtotal (95% CI)**			**15**			**15**	**100.0%**	**1.20 [-0.33, 2.73]**
Heterogeneity: Not applicable								
Test for overall effect: Z = 1.53 (P = 0.12)								
**1.1.17 LFrTMS+ctDCS vs. control**								
rTMS_2020 Gong	4.07	1.73	15	3.2	1.43	15	100.0%	0.87 [-0.27, 2.01]
**Subtotal (95% CI)**			**15**			**15**	**100.0%**	**0.87 [-0.27, 2.01]**
Heterogeneity: Not applicable								
Test for overall effect: Z = 1.50 (P = 0.13)								
**1.1.18 LFrTMS+atDCS vs. LFrTMS+ctDCS**								
rTMS_2020 Gong	4.4	2.67	15	4.07	1.73	15	100.0%	0.33 [-1.28, 1.94]
**Subtotal (95% CI)**			**15**			**15**	**100.0%**	**0.33 [-1.28, 1.94]**
Heterogeneity: Not applicable								
Test for overall effect: Z = 0.40 (P = 0.69)								
**1.1.19 iTBS+atDCS vs. control**								
tDCS+iTBS_2021 Chen	11.33	10.52	12	7	7.35	12	100.0%	4.33 [-2.93, 11.59]
**Subtotal (95% CI)**			**12**			**12**	**100.0%**	**4.33 [-2.93, 11.59]**
Heterogeneity: Not applicable								
Test for overall effect: Z = 1.17 (P = 0.24)								
**1.1.20 PNS+dtDCS vs. control**								
SES+dt_2017 Takebayashi	9.2	4.64	10	4.56	2.6	9	100.0%	4.64 [1.30, 7.98]
**Subtotal (95% CI)**			**10**			**9**	**100.0%**	**4.64 [1.30, 7.98]**
Heterogeneity: Not applicable								
Test for overall effect: Z = 2.72 (P = 0.006)								
**1.1.21 drTMS vs. control**								
rTMS_2018 Long	12.67	3.44	21	4.85	3.21	20	88.6%	7.82 [5.78, 9.86]
rTMS_2020 Chiu	2.34	33.47	14	8	30.33	15	11.4%	-5.66 [-28.96, 17.64]
**Subtotal (95% CI)**			**35**			**35**	**100.0%**	**6.28 [-2.12, 14.68]**
Heterogeneity: $Tau^2 = 19.64$; $Chi^2 = 1.28$, df = 1 (P = 0.26); $I^2 = 22\%$								
Test for overall effect: Z = 1.47 (P = 0.14)								
**1.1.22 iTBS vs. control**								
iTBS_2013 Hsu	22.2	17.15	6	12.5	7.1	6	11.3%	9.70 [-5.15, 24.55]
iTBS_2019 Chen	1.32	19.8	11	-2.97	21.48	11	8.4%	4.29 [-12.97, 21.55]
iTBS_2021 Chen (2)	3.59	15.85	13	6.09	17.63	11	13.7%	-2.50 [-16.02, 11.02]
rTMS vs. iTBS vs, R_2018 Watanabe	18.85	10.89	8	13.86	4.39	6	36.1%	4.99 [-3.33, 13.31]
rTMS vs. iTBS vs. rTMS+iTBS vs. R_2013 Sung	2	11.13	12	1.4	12.42	14	30.5%	0.60 [-8.45, 9.65]
**Subtotal (95% CI)**			**50**			**48**	**100.0%**	**3.10 [-1.90, 8.10]**
Heterogeneity: $Tau^2 = 0.00$; $Chi^2 = 1.93$, df = 4 (P = 0.75); $I^2 = 0\%$								
Test for overall effect: Z = 1.22 (P = 0.22)								
**1.1.23 HFrTMS vs. control**								
rTMS_2010 Chang	8	8.3	18	4.67	9.66	10	14.5%	3.33 [-3.78, 10.44]
rTMS_2016 Du	23.07	14.44	23	14.36	12.19	23	12.3%	8.71 [0.99, 16.43]
rTMS_2017 Guan	22.4	8.3	21	15.2	2.7	21	52.8%	7.20 [3.47, 10.93]
rTMS_2020 Wang	11.4	9.18	15	2.8	7.53	15	20.4%	8.60 [2.59, 14.61]
**Subtotal (95% CI)**			**77**			**69**	**100.0%**	**7.11 [4.40, 9.82]**
Heterogeneity: $Tau^2 = 0.00$; $Chi^2 = 1.49$, df = 3 (P = 0.68); $I^2 = 0\%$								
Test for overall effect: Z = 5.14 ($P < 0.00001$)								

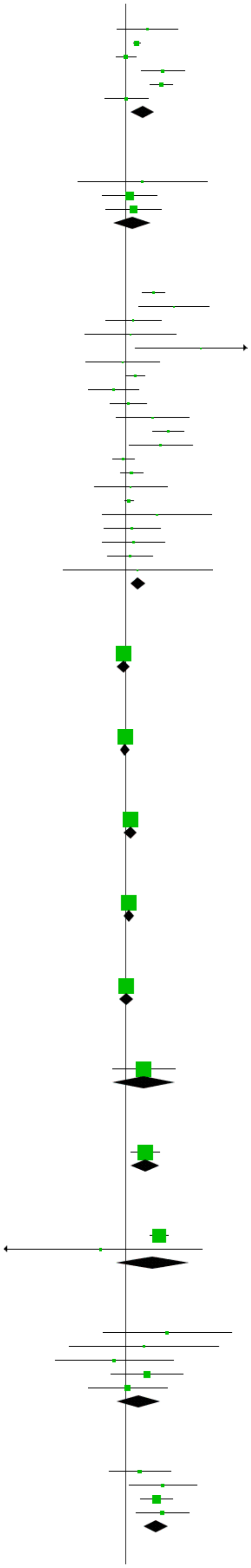

**1.1.24 LFrTMS vs. rPMS**								
rTMS vs. FMS_2020 Chen	10.5	12.82	16	15.21	14.04	19	100.0%	-4.71 [-13.62, 4.20]
**Subtotal (95% CI)**			**16**			**19**	**100.0%**	**-4.71 [-13.62, 4.20]**
Heterogeneity: Not applicable								
Test for overall effect: Z = 1.04 (P = 0.30)								
**1.1.25 LFrTMS vs. HFrTMS**								
rTMS_2016 Du	22.56	12.73	23	23.07	14.44	23	49.4%	-0.51 [-8.38, 7.36]
rTMS_2020 Wang	4.7	12.27	15	11.4	9.18	15	50.6%	-6.70 [-14.45, 1.05]
**Subtotal (95% CI)**			**38**			**38**	**100.0%**	**-3.64 [-9.71, 2.42]**
Heterogeneity: Tau² = 3.27; Chi² = 1.21, df = 1 (P = 0.27); I² = 17%								
Test for overall effect: Z = 1.18 (P = 0.24)								
**1.1.26 LFrTMS vs. ctDCS**								
rTMS vs. tDCS_2020 Miu	6.88	11.41	25	7.35	9.34	26	100.0%	-0.47 [-6.21, 5.27]
**Subtotal (95% CI)**			**25**			**26**	**100.0%**	**-0.47 [-6.21, 5.27]**
Heterogeneity: Not applicable								
Test for overall effect: Z = 0.16 (P = 0.87)								
**1.1.27 LFrTMS vs. cTBS**								
rTMS vs. cTBS vs. R_2021 Kuzu	7.71	3.04	7	4.14	1.68	7	100.0%	3.57 [1.00, 6.14]
**Subtotal (95% CI)**			**7**			**7**	**100.0%**	**3.57 [1.00, 6.14]**
Heterogeneity: Not applicable								
Test for overall effect: Z = 2.72 (P = 0.007)								
**1.1.28 cTBS vs. control**								
rTMS vs. cTBS vs. R_2021 Kuzu	4.14	1.68	7	1.17	1.47	6	100.0%	2.97 [1.26, 4.68]
**Subtotal (95% CI)**			**7**			**6**	**100.0%**	**2.97 [1.26, 4.68]**
Heterogeneity: Not applicable								
Test for overall effect: Z = 3.40 (P = 0.0007)								
**1.1.29 LFrTMS vs. drTMS**								
rTMS_2018 Long	6.29	5.2	21	12.67	3.44	21	100.0%	-6.38 [-9.05, -3.71]
**Subtotal (95% CI)**			**21**			**21**	**100.0%**	**-6.38 [-9.05, -3.71]**
Heterogeneity: Not applicable								
Test for overall effect: Z = 4.69 (P < 0.00001)								
**1.1.30 iTBS+LFrTMS vs. control**								
rTMS vs. iTBS vs. R_2020 Meng	8.69	5.84	10	2.14	7.3	8	66.1%	6.55 [0.33, 12.77]
rTMS vs. iTBS vs. rTMS+iTBS vs. R_2013 Sung	2.9	11.37	15	1.4	12.42	14	33.9%	1.50 [-7.19, 10.19]
**Subtotal (95% CI)**			**25**			**22**	**100.0%**	**4.84 [-0.22, 9.89]**
Heterogeneity: Tau² = 0.00; Chi² = 0.86, df = 1 (P = 0.35); I² = 0%								
Test for overall effect: Z = 1.88 (P = 0.06)								
**1.1.31 iTBS+LFrTMS vs. LFrTMS**								
rTMS vs. iTBS vs. R_2020 Meng	8.69	5.84	10	4.02	6.41	10	78.1%	4.67 [-0.70, 10.04]
rTMS vs. iTBS vs. rTMS+iTBS vs. R_2013 Sung	2.9	11.37	15	2.6	15.36	13	21.9%	0.30 [-9.84, 10.44]
**Subtotal (95% CI)**			**25**			**23**	**100.0%**	**3.71 [-1.04, 8.46]**
Heterogeneity: Tau² = 0.00; Chi² = 0.56, df = 1 (P = 0.46); I² = 0%								
Test for overall effect: Z = 1.53 (P = 0.13)								
**1.1.32 LFrTMS vs. iTBS**								
rTMS vs. iTBS vs, R_2018 Watanabe	25.11	9.92	7	18.85	10.89	8	49.6%	6.26 [-4.27, 16.79]
rTMS vs. iTBS vs. rTMS+iTBS vs. R_2013 Sung	2.6	15.36	13	2	11.13	12	50.4%	0.60 [-9.86, 11.06]
**Subtotal (95% CI)**			**20**			**20**	**100.0%**	**3.41 [-4.01, 10.83]**
Heterogeneity: Tau² = 0.00; Chi² = 0.56, df = 1 (P = 0.45); I² = 0%								
Test for overall effect: Z = 0.90 (P = 0.37)								
**1.1.33 LFrTMS+cNMES vs. control**								
rTMS vs. NMES+rTMS vs. R_2017 Tosun	12.7	13.16	7	4.7	19.11	9	100.0%	8.00 [-7.84, 23.84]
**Subtotal (95% CI)**			**7**			**9**	**100.0%**	**8.00 [-7.84, 23.84]**
Heterogeneity: Not applicable								
Test for overall effect: Z = 0.99 (P = 0.32)								
**1.1.34 LFrTMS+cNMES vs. LFrTMS**								
rTMS vs. NMES+rTMS vs. R_2017 Tosun	12.7	13.16	7	22.2	13.41	9	100.0%	-9.50 [-22.61, 3.61]
**Subtotal (95% CI)**			**7**			**9**	**100.0%**	**-9.50 [-22.61, 3.61]**
Heterogeneity: Not applicable								
Test for overall effect: Z = 1.42 (P = 0.16)								
**1.1.35 iTBS+LFrTMS vs.iTBS**								
rTMS vs. iTBS vs. rTMS+iTBS vs. R_2013 Sung	2.9	11.37	15	2	11.13	12	100.0%	0.90 [-7.63, 9.43]
**Subtotal (95% CI)**			**15**			**12**	**100.0%**	**0.90 [-7.63, 9.43]**
Heterogeneity: Not applicable								
Test for overall effect: Z = 0.21 (P = 0.84)								
**1.1.36 ctDCS vs. atDCS**								
tDCS_2011 Hesse	15.6	13.13	32	15.4	16.73	32	34.4%	0.20 [-7.17, 7.57]
tDCS_2015 Rocha	7.6	3.8	7	13.2	3.3	7	65.6%	-5.60 [-9.33, -1.87]
**Subtotal (95% CI)**			**39**			**39**	**100.0%**	**-3.61 [-9.01, 1.79]**
Heterogeneity: Tau² = 7.94; Chi² = 1.89, df = 1 (P = 0.17); I² = 47%								
Test for overall effect: Z = 1.31 (P = 0.19)								
**1.1.37 rPMS vs. control**								
rPMS_2014 Krewer	0.67	17.88	31	0	3.55	32	82.1%	0.67 [-5.74, 7.08]
rPMS_2020 Obayashia	23.7	15.16	10	17.5	15.33	9	17.9%	6.20 [-7.53, 19.93]
**Subtotal (95% CI)**			**41**			**41**	**100.0%**	**1.66 [-4.15, 7.47]**
Heterogeneity: Tau² = 0.00; Chi² = 0.51, df = 1 (P = 0.47); I² = 0%								
Test for overall effect: Z = 0.56 (P = 0.58)								
**1.1.38 nVNS vs. control**								
nVNS_2017 Capone	5.4	2.7	7	2.8	2.7	5	43.8%	2.60 [-0.50, 5.70]
nVNS_2020 Wu	7.4	1.78	10	4.18	4.24	11	56.2%	3.22 [0.48, 5.96]
**Subtotal (95% CI)**			**17**			**16**	**100.0%**	**2.95 [0.90, 5.00]**
Heterogeneity: Tau² = 0.00; Chi² = 0.09, df = 1 (P = 0.77); I² = 0%								
Test for overall effect: Z = 2.82 (P = 0.005)								
**1.1.39 FES vs. SES**								
FES vs. SES_2008 Chan	7.7	4.2	10	2.1	6.2	10	100.0%	5.60 [0.96, 10.24]
**Subtotal (95% CI)**			**10**			**10**	**100.0%**	**5.60 [0.96, 10.24]**
Heterogeneity: Not applicable								
Test for overall effect: Z = 2.36 (P = 0.02)								

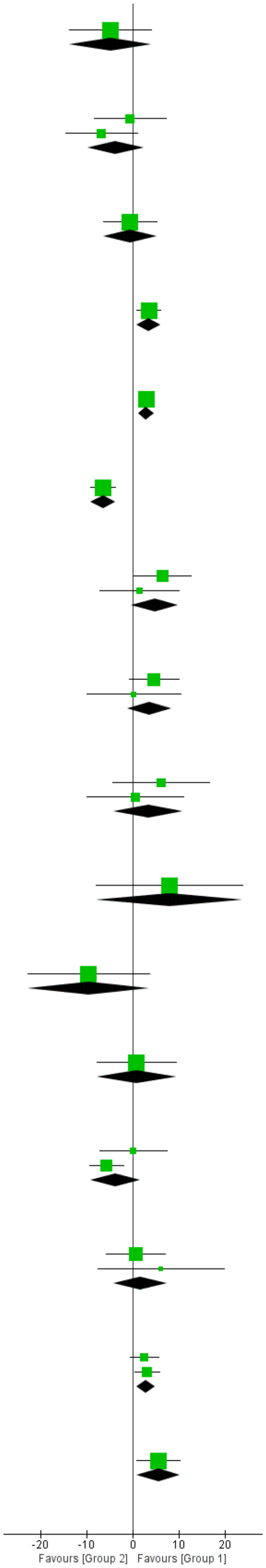

FMA-UE EOT

Study or Subgroup	Group 1 Mean	Group 1 SD	Group 1 Total	Group 2 Mean	Group 2 SD	Group 2 Total	Weight	Mean Difference IV, Random, 95% CI
**2.1.1 VNS vs. control**								
VNS_2016 Dawson	8.7	5.8	9	3	6.1	11	7.9%	5.70 [0.47, 10.93]
VNS_2018 Kimberley	7.62	8.93	8	5.33	9.19	9	2.9%	2.29 [-6.33, 10.91]
VNS_2021 Dawson	5	4.4	53	2.4	3.8	55	89.2%	2.60 [1.05, 4.15]
**Subtotal (95% CI)**			**70**			**75**	**100.0%**	**2.83 [1.37, 4.30]**
Heterogeneity: Tau² = 0.00; Chi² = 1.26, df = 2 (P = 0.53); I² = 0%								
Test for overall effect: Z = 3.79 (P = 0.0002)								
**2.1.2 nVNS vs. control**								
nVNS_2017 Capone	5.4	2.7	7	2.8	2.7	5	15.7%	2.60 [-0.50, 5.70]
nVNS_2020 Wu	6.9	1.85	10	3.18	1.17	11	84.3%	3.72 [2.38, 5.06]
**Subtotal (95% CI)**			**17**			**16**	**100.0%**	**3.54 [2.31, 4.77]**
Heterogeneity: Tau² = 0.00; Chi² = 0.42, df = 1 (P = 0.52); I² = 0%								
Test for overall effect: Z = 5.65 (P < 0.00001)								
**2.1.3 ENMES vs. control**								
NMES_2016 Amasyali	9.71	17.03	7	5.75	17.43	8	100.0%	3.96 [-13.51, 21.43]
**Subtotal (95% CI)**			**7**			**8**	**100.0%**	**3.96 [-13.51, 21.43]**
Heterogeneity: Not applicable								
Test for overall effect: Z = 0.44 (P = 0.66)								
**2.1.4 cNMES vs. control**								
NMES_2011 Shindo	12.2	5.3	10	5.5	6	10	39.7%	6.70 [1.74, 11.66]
NMES_2013 Shimodozono	18.5	11.9	9	9.7	8.41	9	23.0%	8.80 [-0.72, 18.32]
NMES_2015 McCabe	8.8	7.3	14	9.9	7.37	13	37.2%	-1.10 [-6.64, 4.44]
**Subtotal (95% CI)**			**33**			**32**	**100.0%**	**4.28 [-1.74, 10.30]**
Heterogeneity: Tau² = 17.34; Chi² = 5.37, df = 2 (P = 0.07); I² = 63%								
Test for overall effect: Z = 1.39 (P = 0.16)								
**2.1.5 ENMES vs. cNMES**								
NMES vs. SES_2013 Boyaci	8	15.25	11	5	14.05	10	15.1%	3.00 [-9.53, 15.53]
NMES vs. SES_2016 Wilson	4.9	14.57	41	7.1	13.06	39	64.8%	-2.20 [-8.26, 3.86]
NMES_2016 Jeon	3.1	12.74	10	1.8	12.11	10	20.0%	1.30 [-9.59, 12.19]
**Subtotal (95% CI)**			**62**			**59**	**100.0%**	**-0.71 [-5.59, 4.17]**
Heterogeneity: Tau² = 0.00; Chi² = 0.70, df = 2 (P = 0.70); I² = 0%								
Test for overall effect: Z = 0.29 (P = 0.77)								
**2.1.6 ENMES vs. SES**								
NMES vs. SES_2013 Boyaci	8	15.25	11	1	19.63	10	11.3%	7.00 [-8.14, 22.14]
NMES vs. SES_2016 Wilson	4.9	14.57	41	6.8	13.93	42	68.9%	-1.90 [-8.03, 4.23]
NMES vs. SES_2017 Chuang	4.06	19.07	19	1.63	16.89	19	19.8%	2.43 [-9.02, 13.88]
**Subtotal (95% CI)**			**71**			**71**	**100.0%**	**-0.04 [-5.13, 5.06]**
Heterogeneity: Tau² = 0.00; Chi² = 1.36, df = 2 (P = 0.51); I² = 0%								
Test for overall effect: Z = 0.01 (P = 0.99)								
**2.1.7 cNMES vs. SES**								
NMES vs. SES_2013 Boyaci	5	14.05	10	1	19.63	10	7.8%	4.00 [-10.96, 18.96]
NMES vs. SES_2016 Wilson	7.1	13.06	39	6.8	13.93	42	50.2%	0.30 [-5.58, 6.18]
NMES vs.SES_2020 Yurdakul	6.67	11	15	4.8	6.34	15	42.0%	1.87 [-4.56, 8.30]
**Subtotal (95% CI)**			**64**			**67**	**100.0%**	**1.25 [-2.92, 5.41]**
Heterogeneity: Tau² = 0.00; Chi² = 0.27, df = 2 (P = 0.88); I² = 0%								
Test for overall effect: Z = 0.59 (P = 0.56)								
**2.1.8 SES vs. control**								
SES_2015 Fleming	2.5	3.1	16	0.6	3.6	17	19.6%	1.90 [-0.39, 4.19]
SES_2016 Carrico	10.2	4.32	10	5.56	4.32	9	9.6%	4.64 [0.75, 8.53]
SES_2017 Jung	6	1.94	23	3.4	1.75	23	34.4%	2.60 [1.53, 3.67]
SES_2018 Ghaziani	10.7	10.03	53	12.4	14.64	49	6.6%	-1.70 [-6.61, 3.21]
SES_2018 Pan	-0.46	6.34	6	2.2	5.11	6	4.0%	-2.66 [-9.18, 3.86]
SES_2021 Alwhaibi	3.2	2.39	20	2.4	3.1	20	25.8%	0.80 [-0.92, 2.52]
**Subtotal (95% CI)**			**128**			**124**	**100.0%**	**1.70 [0.33, 3.07]**
Heterogeneity: Tau² = 1.12; Chi² = 9.02, df = 5 (P = 0.11); I² = 45%								
Test for overall effect: Z = 2.44 (P = 0.01)								
**2.1.9 MCS vs. control**								
MCS_2006 Brown	10.2	9.66	4	3.9	10.42	4	1.1%	6.30 [-7.62, 20.22]
MCS_2008 Huang	5.86	5.38	12	3.67	4.86	12	13.1%	2.19 [-1.91, 6.29]
MCS_2016 Lewy	3.5	5.2	94	3.4	4.7	58	85.8%	0.10 [-1.50, 1.70]
**Subtotal (95% CI)**			**110**			**74**	**100.0%**	**0.44 [-1.04, 1.93]**
Heterogeneity: Tau² = 0.00; Chi² = 1.55, df = 2 (P = 0.46); I² = 0%								
Test for overall effect: Z = 0.59 (P = 0.56)								
**2.1.10 FES vs. cNMES**								
FES vs. NMES_2011 Knutson	8.3	7.33	9	8.3	6.95	8	9.0%	0.00 [-6.79, 6.79]
FES vs. NMES_2014 Shen	7.6	1.4	30	4.9	1.4	30	23.3%	2.70 [1.99, 3.41]
FES vs. NMES_2016 Knutson	2.62	3.39	32	2.72	3.29	40	21.8%	-0.10 [-1.66, 1.46]
FES vs. NMES_2017 Zhou	21.1	7.54	18	12.4	7.54	18	12.8%	8.70 [3.77, 13.63]
FES vs. NMES_2019 Zheng	16.38	4.06	21	8.1	4.53	20	19.0%	8.28 [5.64, 10.92]
FES vs. NMES_2020 Knutson	7.42	7.38	21	6.29	4.41	8	14.1%	1.13 [-3.26, 5.52]
**Subtotal (95% CI)**			**131**			**124**	**100.0%**	**3.45 [0.86, 6.05]**
Heterogeneity: Tau² = 7.42; Chi² = 36.35, df = 5 (P < 0.00001); I² = 86%								
Test for overall effect: Z = 2.61 (P = 0.009)								
**2.1.11 FES vs. SES**								
FES vs. SES_2008 Chan	7.7	4.2	10	2.1	6.2	10	100.0%	5.60 [0.96, 10.24]
**Subtotal (95% CI)**			**10**			**10**	**100.0%**	**5.60 [0.96, 10.24]**
Heterogeneity: Not applicable								
Test for overall effect: Z = 2.36 (P = 0.02)								

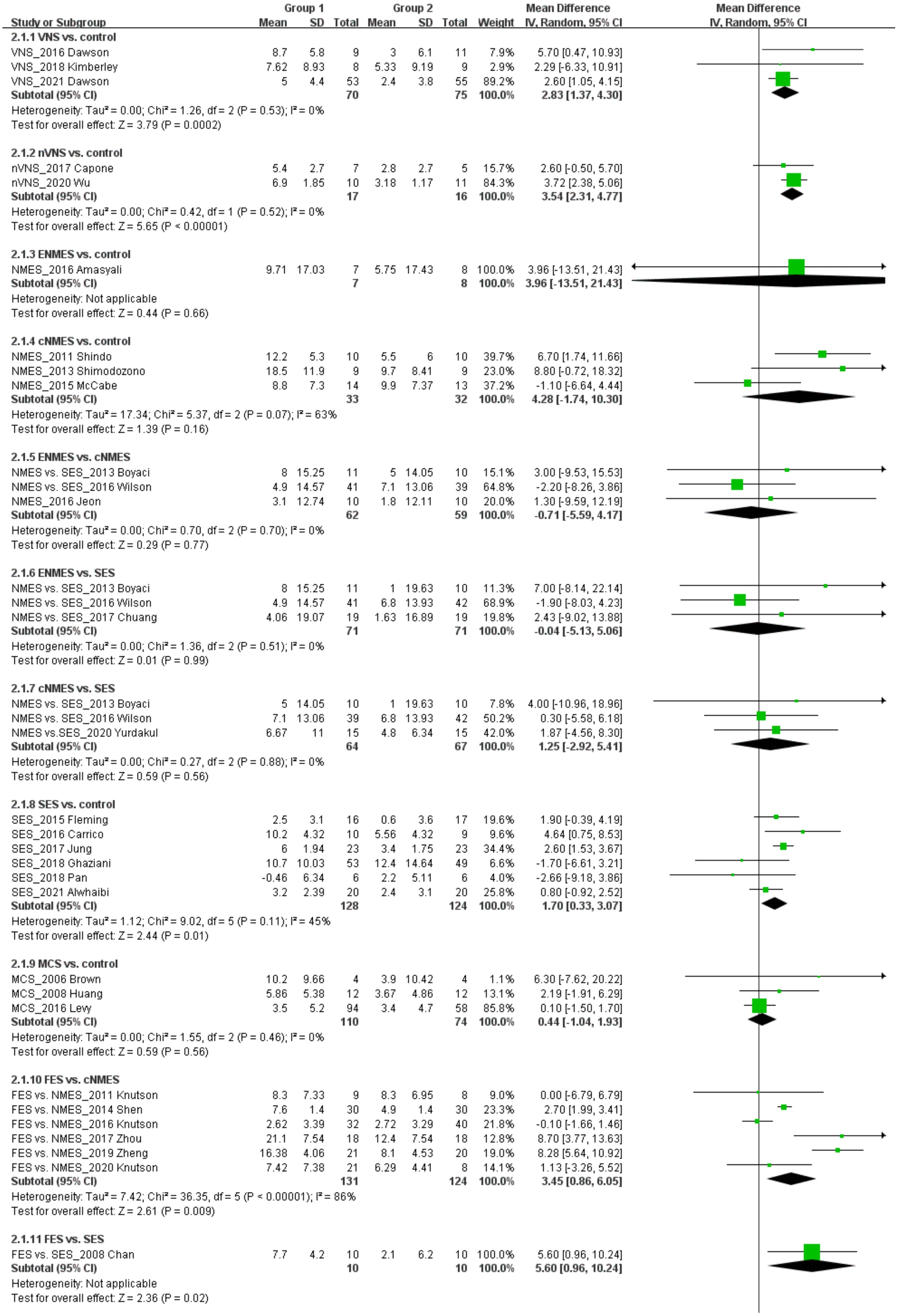

**2.1.12 dtDCS vs. control**								
tDCS_2010 Lindenberg	5.6	12.83	10	1.2	11.65	10	1.9%	4.40 [-6.34, 15.14]
tDCS_2015 Ang	0.9	3	10	2.8	4	9	21.3%	-1.90 [-5.11, 1.31]
tDCS_2018 Shaheiwola	8.5	9.35	15	4.6	13.04	15	3.3%	3.90 [-4.22, 12.02]
tDCS_2019 Beaulieu	5.71	4.54	7	2.71	3.2	7	12.9%	3.00 [-1.11, 7.11]
tDCS_2019 Jin	2.35	16.92	20	1.2	17.73	10	1.2%	1.15 [-12.11, 14.41]
tDCS_2019 Salazar	1.4	9.14	15	1.78	10.36	15	4.5%	-0.38 [-7.37, 6.61]
tDCS_2020 Alisar	9.12	19.38	16	1.74	23.55	16	1.0%	7.38 [-7.56, 22.32]
tDCS_2021 Kim	4.4	2.82	15	3.14	2.81	15	53.9%	1.26 [-0.75, 3.27]
**Subtotal (95% CI)**			**108**			**97**	**100.0%**	**0.95 [-0.53, 2.42]**
Heterogeneity: Tau² = 0.00; Chi² = 5.83, df = 7 (P = 0.56); I² = 0%								
Test for overall effect: Z = 1.25 (P = 0.21)								
**2.1.13 atDCS vs. ctDCS**								
tDCS_2011 Hesse	11.1	2.7	7	7.2	4.5	7	65.4%	3.90 [0.01, 7.79]
tDCS_2015 Rocha	11.3	12.93	32	11	9.28	32	34.6%	0.30 [-5.21, 5.81]
**Subtotal (95% CI)**			**39**			**39**	**100.0%**	**2.65 [-0.70, 6.01]**
Heterogeneity: Tau² = 0.55; Chi² = 1.09, df = 1 (P = 0.30); I² = 9%								
Test for overall effect: Z = 1.55 (P = 0.12)								
**2.1.14 atDCS vs. control**								
tDCS_2011 Hesse	11.3	12.93	32	11	13.36	32	7.9%	0.30 [-6.14, 6.74]
tDCS_2014 Viana	9.3	5.7	10	7.5	7.1	10	10.1%	1.80 [-3.84, 7.44]
tDCS_2015 Rocha	11.1	2.7	7	3.85	6.4	7	11.9%	7.25 [2.10, 12.40]
tDCS_2015 Triccas	8.73	16.13	12	7.73	15.11	11	2.2%	1.00 [-11.77, 13.77]
tDCS_2016 Allman	11.46	14.13	11	9.11	16.18	13	2.4%	2.35 [-9.78, 14.48]
tDCS_2017 Pavlova	4.66	18.29	5	3.49	18.38	6	0.8%	1.17 [-20.59, 22.93]
tDCS_2019 Bornheim	3.33	14.48	25	1.11	13.92	25	5.5%	2.22 [-5.65, 10.09]
tDCS_2019 Edwards	7	4	41	7.7	4.9	41	47.1%	-0.70 [-2.64, 1.24]
tDCS_2019 Mazzoleni	12	8.5	20	15.74	13.75	19	6.4%	-3.74 [-10.96, 3.48]
tDCS_2020 Liao	5.49	12.25	23	3.75	9.87	11	5.7%	1.74 [-5.95, 9.43]
**Subtotal (95% CI)**			**186**			**175**	**100.0%**	**0.80 [-1.10, 2.70]**
Heterogeneity: Tau² = 1.02; Chi² = 9.98, df = 9 (P = 0.35); I² = 10%								
Test for overall effect: Z = 0.83 (P = 0.41)								
**2.1.15 ctDCS vs. LFrTMS**								
rTMS vs. tDCS_2020 Miu	7.35	9.34	26	6.88	11.41	25	100.0%	0.47 [-5.27, 6.21]
**Subtotal (95% CI)**			**26**			**25**	**100.0%**	**0.47 [-5.27, 6.21]**
Heterogeneity: Not applicable								
Test for overall effect: Z = 0.16 (P = 0.87)								
**2.1.16 ctDCS vs. control**								
tDCS_2011 Hesse	11	9.28	32	11	13.36	32	28.9%	0.00 [-5.64, 5.64]
tDCS_2014 Fusco	3.73	28.94	7	4	21.89	7	1.3%	-0.27 [-27.15, 26.61]
tDCS_2014 Lee	10.3	5	20	8.7	10.8	20	33.8%	1.60 [-3.62, 6.82]
tDCS_2015 Rocha	7.2	4.5	7	3.85	6.4	7	27.3%	3.35 [-2.45, 9.15]
tDCS_2020 Yao	10.1	17.23	20	6.4	15.83	20	8.7%	3.70 [-6.55, 13.95]
**Subtotal (95% CI)**			**86**			**86**	**100.0%**	**1.78 [-1.25, 4.81]**
Heterogeneity: Tau² = 0.00; Chi² = 0.83, df = 4 (P = 0.93); I² = 0%								
Test for overall effect: Z = 1.15 (P = 0.25)								
**2.1.17 LFrTMS vs. drTMS**								
rTMS_2018 Long	5.1	6.54	21	6.58	4.01	21	100.0%	-1.48 [-4.76, 1.80]
**Subtotal (95% CI)**			**21**			**21**	**100.0%**	**-1.48 [-4.76, 1.80]**
Heterogeneity: Not applicable								
Test for overall effect: Z = 0.88 (P = 0.38)								
**2.1.18 drTMS vs. control**								
rTMS_2018 Long	6.58	4.01	21	1.05	3.26	20	99.2%	5.53 [3.30, 7.76]
rTMS_2020 Chiu	0.5	34.71	14	2.33	32.99	15	0.8%	-1.83 [-26.51, 22.85]
**Subtotal (95% CI)**			**35**			**35**	**100.0%**	**5.47 [3.25, 7.69]**
Heterogeneity: Tau² = 0.00; Chi² = 0.34, df = 1 (P = 0.56); I² = 0%								
Test for overall effect: Z = 4.82 (P < 0.00001)								
**2.1.19 HFrTMS vs. LFrTMS**								
rTMS_2016 Du	5.36	16.25	23	5.33	12.82	23	46.7%	0.03 [-8.43, 8.49]
rTMS_2020 Wang	11.4	9.18	15	4.7	12.27	15	53.3%	6.70 [-1.05, 14.45]
**Subtotal (95% CI)**			**38**			**38**	**100.0%**	**3.59 [-2.93, 10.11]**
Heterogeneity: Tau² = 5.10; Chi² = 1.30, df = 1 (P = 0.25); I² = 23%								
Test for overall effect: Z = 1.08 (P = 0.28)								
**2.1.20 iTBS vs. LFrTMS**								
rTMS vs. iTBS vs. rTMS+iTBS vs. R_2013 Sung	2	11.13	12	2.6	15.36	13	100.0%	-0.60 [-11.06, 9.86]
**Subtotal (95% CI)**			**12**			**13**	**100.0%**	**-0.60 [-11.06, 9.86]**
Heterogeneity: Not applicable								
Test for overall effect: Z = 0.11 (P = 0.91)								
**2.1.21 cTBS vs. LFrTMS**								
rTMS vs. cTBS vs. R_2021 Kuzu	3.29	1.7	7	5.57	2.51	7	100.0%	-2.28 [-4.53, -0.03]
**Subtotal (95% CI)**			**7**			**7**	**100.0%**	**-2.28 [-4.53, -0.03]**
Heterogeneity: Not applicable								
Test for overall effect: Z = 1.99 (P = 0.05)								
**2.1.22 rPMS vs. LFrTMS**								
rPMS vs TMS_2020 Chen	15.21	14.04	19	10.5	12.82	16	100.0%	4.71 [-4.20, 13.62]
**Subtotal (95% CI)**			**19**			**16**	**100.0%**	**4.71 [-4.20, 13.62]**
Heterogeneity: Not applicable								
Test for overall effect: Z = 1.04 (P = 0.30)								
**2.1.23 cNMES+LFrTMS VS. LFrTMS**								
rTMS vs. NMES+rTMS vs. R_2017 Tosun	12.7	13.16	7	22.2	13.41	9	100.0%	-9.50 [-22.61, 3.61]
**Subtotal (95% CI)**			**7**			**9**	**100.0%**	**-9.50 [-22.61, 3.61]**
Heterogeneity: Not applicable								
Test for overall effect: Z = 1.42 (P = 0.16)								
**2.1.24 cNMES+LFrTMS VS. control**								
rTMS vs. NMES+rTMS vs. R_2017 Tosun	12.7	13.16	7	4.7	19.11	9	100.0%	8.00 [-7.84, 23.84]
**Subtotal (95% CI)**			**7**			**9**	**100.0%**	**8.00 [-7.84, 23.84]**
Heterogeneity: Not applicable								
Test for overall effect: Z = 0.99 (P = 0.32)								
**2.1.25 LFrTMS+atDCS vs. LFrTMS**								
rTMS_2020 Gong	2.86	1.3	15	2.73	1.48	15	100.0%	0.13 [-0.87, 1.13]
**Subtotal (95% CI)**			**15**			**15**	**100.0%**	**0.13 [-0.87, 1.13]**
Heterogeneity: Not applicable								
Test for overall effect: Z = 0.26 (P = 0.80)								
**2.1.26 LFrTMS+ctDCS vs. LFrTMS**								
rTMS_2020 Gong	2.8	1.9	15	2.73	1.48	15	100.0%	0.07 [-1.15, 1.29]
**Subtotal (95% CI)**			**15**			**15**	**100.0%**	**0.07 [-1.15, 1.29]**
Heterogeneity: Not applicable								
Test for overall effect: Z = 0.11 (P = 0.91)								

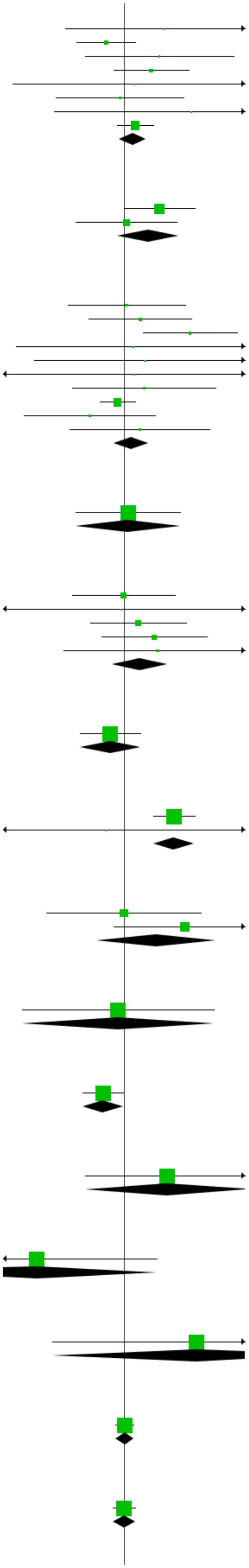

**2.1.27 iTBS+LFrTMS VS. LFrTMS**								
rTMS vs. iTBS vs. R_2020 Meng	8.69	5.84	10	4.02	6.41	10	78.1%	4.67 [-0.70, 10.04]
rTMS vs. iTBS vs. rTMS+iTBS vs. R_2013 Sung	2.9	11.37	15	2.6	15.36	13	21.9%	0.30 [-9.84, 10.44]
**Subtotal (95% CI)**			**25**			**23**	**100.0%**	**3.71 [-1.04, 8.46]**
Heterogeneity: Tau² = 0.00; Chi² = 0.56, df = 1 (P = 0.46); I² = 0%								
Test for overall effect: Z = 1.53 (P = 0.13)								
**2.1.28 LFrTMS vs. control**								
rTMS vs. cTBS vs. R_2021 Kuzu	5.57	2.51	7	1.17	1.47	6	13.5%	4.40 [2.20, 6.60]
rTMS vs. iTBS vs. R_2020 Meng	4.02	6.41	10	2.14	7.3	8	2.8%	1.88 [-4.55, 8.31]
rTMS vs. iTBS vs. rTMS+iTBS vs. R_2013 Sung	2.6	15.36	13	1.4	12.42	14	1.1%	1.20 [-9.39, 11.79]
rTMS vs. NMES+rTMS vs. R_2017 Tosun	22.2	13.41	9	4.7	19.11	9	0.5%	17.50 [2.25, 32.75]
rTMS_2012 Seniow	6.3	13.31	20	6.9	15	20	1.6%	-0.60 [-9.39, 8.19]
rTMS_2013 Abo	5.39	4.28	44	3.09	4.5	22	13.1%	2.30 [0.03, 4.57]
rTMS_2014 Galvao	5.4	6.2	10	8.6	5.9	10	3.9%	-3.20 [-8.50, 2.10]
rTMS_2014 Rose	4.6	4.5	9	3.9	4.74	10	5.8%	0.70 [-3.46, 4.86]
rTMS_2015 Matsuura	8.18	9.44	10	1.82	9.72	10	1.7%	6.36 [-2.04, 14.76]
rTMS_2016 Du	5.33	12.82	23	2.19	12.22	23	2.2%	3.14 [-4.10, 10.38]
rTMS_2018 Harvey	5.6	13.31	132	6.2	13.26	67	6.4%	-0.60 [-4.50, 3.30]
rTMS_2018 Long	5.1	6.54	21	1.05	3.26	20	8.8%	4.05 [0.91, 7.19]
rTMS_2019 Pinto	8.11	11.07	9	6.87	6.77	10	1.7%	1.24 [-7.12, 9.60]
rTMS_2020 Gong	2.73	1.48	15	2.06	0.89	15	23.6%	0.67 [-0.20, 1.54]
rTMS_2020 Kim	13.18	15.72	8	5.83	11.3	12	0.8%	7.35 [-5.28, 19.98]
rTMS_2020 Sharma	3.73	15.69	47	2.21	16.85	49	2.7%	1.52 [-4.99, 8.03]
rTMS_2020 Wang	4.7	12.27	15	2.8	7.53	15	2.2%	1.90 [-5.39, 9.19]
rTMS_2020 Won-Seok	10.3	7.4	36	9.8	8.7	37	7.0%	0.50 [-3.20, 4.20]
rTMS_2021 Gottlieb	6.43	23.92	14	3.5	22.38	14	0.4%	2.93 [-14.23, 20.09]
**Subtotal (95% CI)**			**452**			**371**	**100.0%**	**1.83 [0.69, 2.96]**
Heterogeneity: Tau² = 1.21; Chi² = 24.35, df = 18 (P = 0.14); I² = 26%								
Test for overall effect: Z = 3.16 (P = 0.002)								
**2.1.29 HFrTMS vs. control**								
rTMS_2010 Chang	3.72	8.28	18	2.31	9.64	10	14.4%	1.41 [-5.68, 8.50]
rTMS_2016 Du	5.36	16.25	23	2.19	12.22	23	11.1%	3.17 [-5.14, 11.48]
rTMS_2017 Guan	8.6	2.9	21	6.7	2.4	21	56.0%	1.90 [0.29, 3.51]
rTMS_2020 Wang	11.4	9.18	15	2.8	7.53	15	18.5%	8.60 [2.59, 14.61]
**Subtotal (95% CI)**			**77**			**69**	**100.0%**	**3.21 [0.17, 6.25]**
Heterogeneity: Tau² = 3.61; Chi² = 4.56, df = 3 (P = 0.21); I² = 34%								
Test for overall effect: Z = 2.07 (P = 0.04)								
**2.1.30 iTBS+LFrTMS VS. iTBS**								
rTMS vs. iTBS vs. rTMS+iTBS vs. R_2013 Sung	2.9	11.37	15	2	11.13	12	100.0%	0.90 [-7.63, 9.43]
**Subtotal (95% CI)**			**15**			**12**	**100.0%**	**0.90 [-7.63, 9.43]**
Heterogeneity: Not applicable								
Test for overall effect: Z = 0.21 (P = 0.84)								
**2.1.31 iTBS vs. control**								
iTBS_2013 Hsu	15.8	12	6	8.5	3.43	6	32.3%	7.30 [-2.69, 17.29]
iTBS_2019 Chen	1.32	19.8	11	-2.97	21.48	11	10.8%	4.29 [-12.97, 21.55]
iTBS_2021 Chen (2)	3.59	15.85	13	6.09	17.63	11	17.6%	-2.50 [-16.02, 11.02]
rTMS vs. iTBS vs. rTMS+iTBS vs. R_2013 Sung	2	11.13	12	1.4	12.42	14	39.3%	0.60 [-8.45, 9.65]
**Subtotal (95% CI)**			**42**			**42**	**100.0%**	**2.62 [-3.06, 8.29]**
Heterogeneity: Tau² = 0.00; Chi² = 1.62, df = 3 (P = 0.65); I² = 0%								
Test for overall effect: Z = 0.90 (P = 0.37)								
**2.1.32 cTBS vs. control**								
rTMS vs. cTBS vs. R_2021 Kuzu	3.29	1.7	7	1.17	1.47	6	100.0%	2.12 [0.40, 3.84]
**Subtotal (95% CI)**			**7**			**6**	**100.0%**	**2.12 [0.40, 3.84]**
Heterogeneity: Not applicable								
Test for overall effect: Z = 2.41 (P = 0.02)								
**2.1.33 rPMS vs. control**								
rPMS_2014 Krewer	-0.33	17.15	31	1.66	6.72	32	78.5%	-1.99 [-8.46, 4.48]
rPMS_2020 Obayashia	23.7	15.16	10	17.5	15.33	9	21.5%	6.20 [-7.53, 19.93]
**Subtotal (95% CI)**			**41**			**41**	**100.0%**	**-0.23 [-6.82, 6.37]**
Heterogeneity: Tau² = 3.54; Chi² = 1.12, df = 1 (P = 0.29); I² = 11%								
Test for overall effect: Z = 0.07 (P = 0.95)								
**2.1.34 SES+dtDCS vs. control**								
SES+dt_2017 Takebayashi	9.2	4.64	10	4.56	2.6	9	100.0%	4.64 [1.30, 7.98]
**Subtotal (95% CI)**			**10**			**9**	**100.0%**	**4.64 [1.30, 7.98]**
Heterogeneity: Not applicable								
Test for overall effect: Z = 2.72 (P = 0.006)								
**2.1.35 LFrTMS+atDCS vs. LFrTMS+ctDCS**								
rTMS_2020 Gong	2.86	1.3	15	2.8	1.9	15	100.0%	0.06 [-1.11, 1.23]
**Subtotal (95% CI)**			**15**			**15**	**100.0%**	**0.06 [-1.11, 1.23]**
Heterogeneity: Not applicable								
Test for overall effect: Z = 0.10 (P = 0.92)								
**2.1.36 LFrTMS+atDCS vs. control**								
rTMS_2020 Gong	2.86	1.3	15	2.06	0.89	15	100.0%	0.80 [0.00, 1.60]
**Subtotal (95% CI)**			**15**			**15**	**100.0%**	**0.80 [0.00, 1.60]**
Heterogeneity: Not applicable								
Test for overall effect: Z = 1.97 (P = 0.05)								
**2.1.37 LFrTMS+ctDCS vs. control**								
rTMS_2020 Gong	2.8	1.9	15	2.06	0.89	15	100.0%	0.74 [-0.32, 1.80]
**Subtotal (95% CI)**			**15**			**15**	**100.0%**	**0.74 [-0.32, 1.80]**
Heterogeneity: Not applicable								
Test for overall effect: Z = 1.37 (P = 0.17)								
**2.1.38 iTBS+atDCS vs. control**								
iTBS_2019 Chen	11.33	10.52	12	7	7.35	12	100.0%	4.33 [-2.93, 11.59]
**Subtotal (95% CI)**			**12**			**12**	**100.0%**	**4.33 [-2.93, 11.59]**
Heterogeneity: Not applicable								
Test for overall effect: Z = 1.17 (P = 0.24)								
**2.1.39 iTBS+LFrTMS vs. control**								
rTMS vs. iTBS vs. R_2020 Meng	8.69	5.84	10	2.14	7.3	8	66.1%	6.55 [0.33, 12.77]
rTMS vs. iTBS vs. rTMS+iTBS vs. R_2013 Sung	2.9	11.37	15	1.4	12.42	14	33.9%	1.50 [-7.19, 10.19]
**Subtotal (95% CI)**			**25**			**22**	**100.0%**	**4.84 [-0.22, 9.89]**
Heterogeneity: Tau² = 0.00; Chi² = 0.86, df = 1 (P = 0.35); I² = 0%								
Test for overall effect: Z = 1.88 (P = 0.06)								

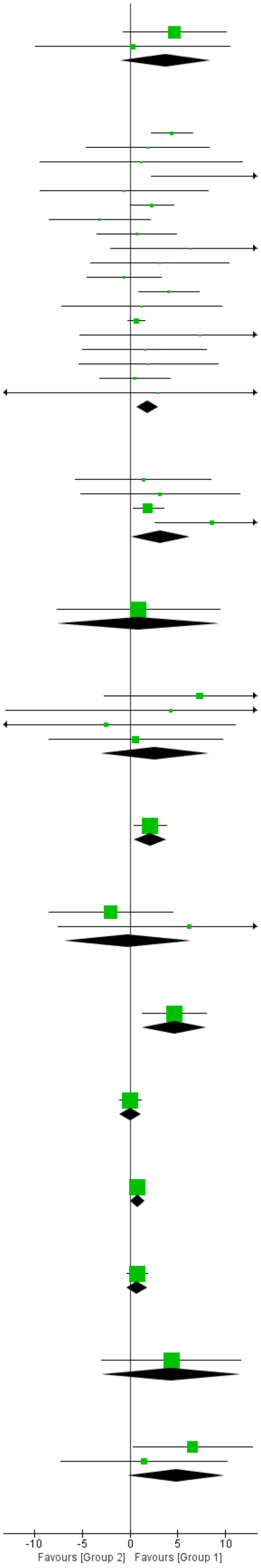

Test for subgroup differences: Chi² = 85.36, df = 38 (P < 0.0001), I² = 55.5%

FMA-UE 1 month

Study or Subgroup	Group 1 Mean	Group 1 SD	Group 1 Total	Group 2 Mean	Group 2 SD	Group 2 Total	Weight	Mean Difference IV, Random, 95% CI
**3.1.1 VNS vs. control**								
VNS_2018 Kimberley	8	8.44	8	5.77	9.73	9	100.0%	2.23 [-6.41, 10.87]
**Subtotal (95% CI)**			**8**			**9**	**100.0%**	**2.23 [-6.41, 10.87]**
Heterogeneity: Not applicable								
Test for overall effect: Z = 0.51 (P = 0.61)								
**3.1.2 nVNS vs. control**								
nVNS_2020 Wu	7.7	1.49	10	3.36	1.75	11	100.0%	4.34 [2.95, 5.73]
**Subtotal (95% CI)**			**10**			**11**	**100.0%**	**4.34 [2.95, 5.73]**
Heterogeneity: Not applicable								
Test for overall effect: Z = 6.14 (P < 0.00001)								
**3.1.3 ENMES vs. cNMES**								
NMES vs. SES_2016 Wilson	6.5	14.37	41	6.1	13.31	39	100.0%	0.40 [-5.67, 6.47]
**Subtotal (95% CI)**			**41**			**39**	**100.0%**	**0.40 [-5.67, 6.47]**
Heterogeneity: Not applicable								
Test for overall effect: Z = 0.13 (P = 0.90)								
**3.1.4 ENMES vs. SES**								
NMES vs. SES_2016 Wilson	6.5	14.37	41	8.4	14.45	42	77.2%	-1.90 [-8.10, 4.30]
NMES vs. SES_2017 Chuang	4.37	18.8	19	1.31	17.07	19	22.8%	3.06 [-8.36, 14.48]
**Subtotal (95% CI)**			**60**			**61**	**100.0%**	**-0.77 [-6.22, 4.68]**
Heterogeneity: Tau² = 0.00; Chi² = 0.56, df = 1 (P = 0.45); I² = 0%								
Test for overall effect: Z = 0.28 (P = 0.78)								
**3.1.5 cNMES vs. FES**								
FES vs. NMES_2011 Knutson	8	7.14	8	8.7	7.43	9	100.0%	-0.70 [-7.63, 6.23]
**Subtotal (95% CI)**			**8**			**9**	**100.0%**	**-0.70 [-7.63, 6.23]**
Heterogeneity: Not applicable								
Test for overall effect: Z = 0.20 (P = 0.84)								
**3.1.6 cNMES vs. SES**								
NMES vs. SES_2016 Wilson	6.1	13.31	39	8.4	14.45	42	100.0%	-2.30 [-8.35, 3.75]
**Subtotal (95% CI)**			**39**			**42**	**100.0%**	**-2.30 [-8.35, 3.75]**
Heterogeneity: Not applicable								
Test for overall effect: Z = 0.75 (P = 0.46)								
**3.1.7 SES vs. control**								
SES_2016 Carrico	12.5	6.28	10	6.58	7.17	9	100.0%	5.92 [-0.17, 12.01]
**Subtotal (95% CI)**			**10**			**9**	**100.0%**	**5.92 [-0.17, 12.01]**
Heterogeneity: Not applicable								
Test for overall effect: Z = 1.91 (P = 0.06)								
**3.1.8 MCS vs. control**								
MCS_2008 Huang	5.5	4.4	12	1.9	4.4	12	26.9%	3.60 [0.08, 7.12]
MCS_2008 Levy	4.3	5.3	94	4	4.3	58	47.2%	0.30 [-1.24, 1.84]
MCS_2016 Levy	5.62	4.57	12	2.05	4.57	12	25.8%	3.57 [-0.09, 7.23]
**Subtotal (95% CI)**			**118**			**82**	**100.0%**	**2.03 [-0.47, 4.54]**
Heterogeneity: Tau² = 2.84; Chi² = 4.71, df = 2 (P = 0.09); I² = 58%								
Test for overall effect: Z = 1.59 (P = 0.11)								
**3.1.9 dtDCS vs. control**								
tDCS_2015 Ang	5	4.4	10	5.4	5.7	9	100.0%	-0.40 [-5.02, 4.22]
**Subtotal (95% CI)**			**10**			**9**	**100.0%**	**-0.40 [-5.02, 4.22]**
Heterogeneity: Not applicable								
Test for overall effect: Z = 0.17 (P = 0.87)								
**3.1.10 atDCS vs. ctDCS**								
tDCS_2015 Rocha	13.2	3.3	7	7.6	3.8	7	100.0%	5.60 [1.87, 9.33]
**Subtotal (95% CI)**			**7**			**7**	**100.0%**	**5.60 [1.87, 9.33]**
Heterogeneity: Not applicable								
Test for overall effect: Z = 2.94 (P = 0.003)								
**3.1.11 atDCS vs. control**								
tDCS_2015 Rocha	13.2	3.3	7	5.6	11	7	39.8%	7.60 [-0.91, 16.11]
tDCS_2016 Allman	10.83	14.55	11	10.04	16.08	13	19.1%	0.79 [-11.47, 13.05]
tDCS_2019 Bornheim	12.66	14.6	25	4.89	15.58	25	41.1%	7.77 [-0.60, 16.14]
**Subtotal (95% CI)**			**43**			**45**	**100.0%**	**6.37 [1.00, 11.73]**
Heterogeneity: Tau² = 0.00; Chi² = 0.98, df = 2 (P = 0.61); I² = 0%								
Test for overall effect: Z = 2.33 (P = 0.02)								
**3.1.12 ctDCS vs. LFrTMS**								
rTMS vs. tDCS_2020 Miu	7.35	9.34	26	6.88	11.41	25	100.0%	0.47 [-5.27, 6.21]
**Subtotal (95% CI)**			**26**			**25**	**100.0%**	**0.47 [-5.27, 6.21]**
Heterogeneity: Not applicable								
Test for overall effect: Z = 0.16 (P = 0.87)								

Mean Difference
IV, Random, 95% CI

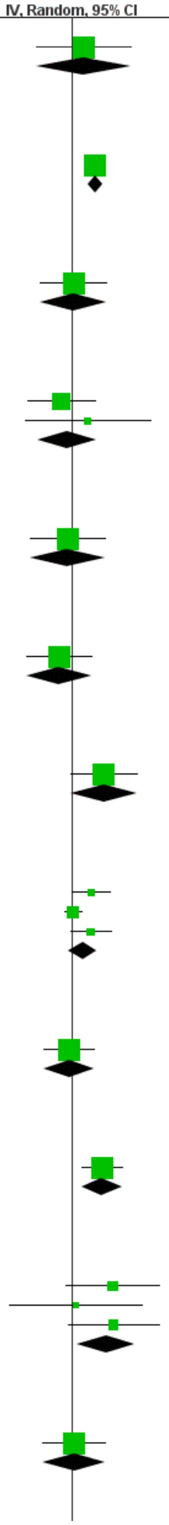

**3.1.13 ctDCS vs. control**								
tDCS_2014 Fusco	7.2	29.81	7	9.87	24.44	7	8.4%	-2.67 [-31.23, 25.89]
tDCS_2015 Rocha	7.6	3.8	7	5.6	11	7	91.6%	2.00 [-6.62, 10.62]
**Subtotal (95% CI)**			**14**			**14**	**100.0%**	**1.61 [-6.64, 9.86]**
Heterogeneity: Tau² = 0.00; Chi² = 0.09, df = 1 (P = 0.76); I² = 0%								
Test for overall effect: Z = 0.38 (P = 0.70)								
**3.1.14 drTMS vs. control**								
rTMS_2020 Chiu	3.84	33.81	14	4.66	33.49	15	100.0%	-0.82 [-25.33, 23.69]
**Subtotal (95% CI)**			**14**			**15**	**100.0%**	**-0.82 [-25.33, 23.69]**
Heterogeneity: Not applicable								
Test for overall effect: Z = 0.07 (P = 0.95)								
**3.1.15 LFrTMS vs. HFrTMS**								
rTMS_2016 Du	11.43	12.32	23	13.17	15.73	23	100.0%	-1.74 [-9.91, 6.43]
**Subtotal (95% CI)**			**23**			**23**	**100.0%**	**-1.74 [-9.91, 6.43]**
Heterogeneity: Not applicable								
Test for overall effect: Z = 0.42 (P = 0.68)								
**3.1.16 LFrTMS vs. cTBS**								
rTMS vs. cTBS vs. R_2021 Kuzu	7.71	3.04	7	4.14	1.68	7	100.0%	3.57 [1.00, 6.14]
**Subtotal (95% CI)**			**7**			**7**	**100.0%**	**3.57 [1.00, 6.14]**
Heterogeneity: Not applicable								
Test for overall effect: Z = 2.72 (P = 0.007)								
**3.1.17 LFrTMS+atDCS vs. LFrTMS**								
rTMS_2020 Gong	4.4	2.67	15	4.06	1.39	15	100.0%	0.34 [-1.18, 1.86]
**Subtotal (95% CI)**			**15**			**15**	**100.0%**	**0.34 [-1.18, 1.86]**
Heterogeneity: Not applicable								
Test for overall effect: Z = 0.44 (P = 0.66)								
**3.1.18 LFrTMS+ctDCS vs. LFrTMS**								
rTMS_2020 Gong	4.07	1.73	15	4.06	1.39	15	100.0%	0.01 [-1.11, 1.13]
**Subtotal (95% CI)**			**15**			**15**	**100.0%**	**0.01 [-1.11, 1.13]**
Heterogeneity: Not applicable								
Test for overall effect: Z = 0.02 (P = 0.99)								
**3.1.19 LFrTMS vs. control**								
rTMS vs. cTBS vs. R_2021 Kuzu	7.71	3.04	7	1.17	1.47	6	17.0%	6.54 [4.00, 9.08]
rTMS_2014 Galvao	4.8	4	10	7.5	8.4	10	11.9%	-2.70 [-8.47, 3.07]
rTMS_2015 Zheng	28.5	9.52	58	18.51	9.94	54	15.4%	9.99 [6.38, 13.60]
rTMS_2016 Du	11.43	12.32	23	4.88	12.02	23	10.1%	6.55 [-0.48, 13.58]
rTMS_2018 Harvey	6.3	13.31	132	6.3	14.5	67	14.5%	0.00 [-4.15, 4.15]
rTMS_2020 Gong	4.06	1.39	15	3.2	1.43	15	18.6%	0.86 [-0.15, 1.87]
rTMS_2020 Won-Seok	14.9	10.8	36	13.8	12.1	37	12.7%	1.10 [-4.16, 6.36]
**Subtotal (95% CI)**			**281**			**212**	**100.0%**	**3.28 [0.01, 6.55]**
Heterogeneity: Tau² = 14.72; Chi² = 41.15, df = 6 (P < 0.00001); I² = 85%								
Test for overall effect: Z = 1.97 (P = 0.05)								
**3.1.20 HFrTMS vs. control**								
rTMS_2016 Du	13.17	15.73	23	4.88	12.02	23	11.7%	8.29 [0.20, 16.38]
rTMS_2017 Guan	14.9	4.4	21	11.1	3.8	21	88.3%	3.80 [1.31, 6.29]
**Subtotal (95% CI)**			**44**			**44**	**100.0%**	**4.33 [1.49, 7.16]**
Heterogeneity: Tau² = 0.76; Chi² = 1.08, df = 1 (P = 0.30); I² = 7%								
Test for overall effect: Z = 2.99 (P = 0.003)								
**3.1.21 cTBS vs. control**								
rTMS vs. cTBS vs. R_2021 Kuzu	4.14	1.68	7	1.17	1.47	6	100.0%	2.97 [1.26, 4.68]
**Subtotal (95% CI)**			**7**			**6**	**100.0%**	**2.97 [1.26, 4.68]**
Heterogeneity: Not applicable								
Test for overall effect: Z = 3.40 (P = 0.0007)								
**3.1.22 LFrTMS+atDCS vs. LFrTMS+ctDCS**								
rTMS_2020 Gong	4.4	2.67	15	4.07	1.73	15	100.0%	0.33 [-1.28, 1.94]
**Subtotal (95% CI)**			**15**			**15**	**100.0%**	**0.33 [-1.28, 1.94]**
Heterogeneity: Not applicable								
Test for overall effect: Z = 0.40 (P = 0.69)								
**3.1.23 LFrTMS+atDCS vs. control**								
rTMS_2020 Gong	4.4	2.67	15	3.2	1.43	15	100.0%	1.20 [-0.33, 2.73]
**Subtotal (95% CI)**			**15**			**15**	**100.0%**	**1.20 [-0.33, 2.73]**
Heterogeneity: Not applicable								
Test for overall effect: Z = 1.53 (P = 0.12)								
**3.1.24 LFrTMS+ctDCS vs. control**								
rTMS_2020 Gong	4.07	1.73	15	3.2	1.43	15	100.0%	0.87 [-0.27, 2.01]
**Subtotal (95% CI)**			**15**			**15**	**100.0%**	**0.87 [-0.27, 2.01]**
Heterogeneity: Not applicable								
Test for overall effect: Z = 1.50 (P = 0.13)								

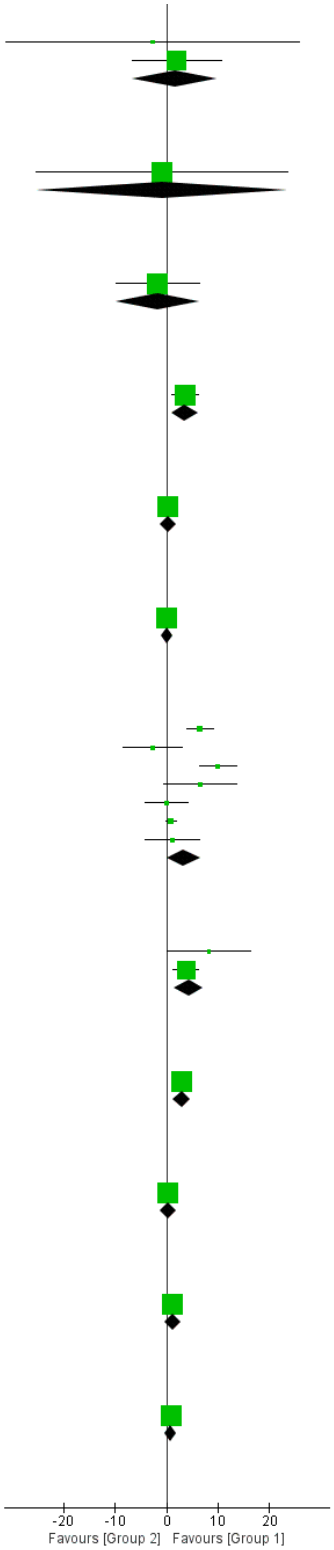

Test for subgroup differences: Chi² = 52.81, df = 23 (P = 0.0004), I² = 56.4%

FMA-UE 3 months

Study or Subgroup	Group 1 Mean	Group 1 SD	Group 1 Total	Group 2 Mean	Group 2 SD	Group 2 Total	Weight	Mean Difference IV, Random, 95% CI
**4.1.1 VNS vs. control**								
VNS_2018 Kimberley	9.5	9.6	8	3.8	9.4	9	5.2%	5.70 [-3.35, 14.75]
VNS_2021 Dawson	5.8	6	53	2.8	5.2	55	94.8%	3.00 [0.88, 5.12]
**Subtotal (95% CI)**			**61**			**64**	**100.0%**	**3.14 [1.08, 5.21]**
Heterogeneity: Tau² = 0.00; Chi² = 0.32, df = 1 (P = 0.57); I² = 0%								
Test for overall effect: Z = 2.98 (P = 0.003)								
**4.1.2 nVNS vs. control**								
nVNS_2020 Wu	7.4	1.78	10	4.18	4.24	11	100.0%	3.22 [0.48, 5.96]
**Subtotal (95% CI)**			**10**			**11**	**100.0%**	**3.22 [0.48, 5.96]**
Heterogeneity: Not applicable								
Test for overall effect: Z = 2.31 (P = 0.02)								
**4.1.3 ENMES vs. cNMES**								
NMES vs. SES_2016 Wilson	9.5	14.87	41	10.4	13.31	39	40.5%	-0.90 [-7.08, 5.28]
NMES_2008 Kroon	2.8	6	11	5	5.9	10	59.5%	-2.20 [-7.29, 2.89]
**Subtotal (95% CI)**			**52**			**49**	**100.0%**	**-1.67 [-5.60, 2.26]**
Heterogeneity: Tau² = 0.00; Chi² = 0.10, df = 1 (P = 0.75); I² = 0%								
Test for overall effect: Z = 0.83 (P = 0.40)								
**4.1.4 ENMES vs. SES**								
NMES vs. SES_2016 Wilson	9.5	14.87	41	10.5	13.93	42	100.0%	-1.00 [-7.20, 5.20]
**Subtotal (95% CI)**			**41**			**42**	**100.0%**	**-1.00 [-7.20, 5.20]**
Heterogeneity: Not applicable								
Test for overall effect: Z = 0.32 (P = 0.75)								
**4.1.5 ENMES vs. control**								
NMES_2007 Hemmen	8.7	7.11	14	11.9	8.47	13	88.9%	-3.20 [-9.12, 2.72]
NMES_2016 Amasyali	13.71	16.48	7	11.55	16.61	8	11.1%	2.16 [-14.62, 18.94]
**Subtotal (95% CI)**			**21**			**21**	**100.0%**	**-2.61 [-8.19, 2.98]**
Heterogeneity: Tau² = 0.00; Chi² = 0.35, df = 1 (P = 0.55); I² = 0%								
Test for overall effect: Z = 0.91 (P = 0.36)								
**4.1.6 cNMES vs. FES**								
FES vs. NMES_2011 Knutson	8.5	7.22	8	13.6	7.57	9	100.0%	-5.10 [-12.13, 1.93]
**Subtotal (95% CI)**			**8**			**9**	**100.0%**	**-5.10 [-12.13, 1.93]**
Heterogeneity: Not applicable								
Test for overall effect: Z = 1.42 (P = 0.16)								
**4.1.7 cNMES vs. SES**								
NMES vs. SES_2016 Wilson	10.4	13.31	39	10.5	13.93	42	100.0%	-0.10 [-6.03, 5.83]
**Subtotal (95% CI)**			**39**			**42**	**100.0%**	**-0.10 [-6.03, 5.83]**
Heterogeneity: Not applicable								
Test for overall effect: Z = 0.03 (P = 0.97)								
**4.1.8 SES vs. control**								
SES_2015 Fleming	2.5	4.2	16	2	4.9	17	80.9%	0.50 [-2.61, 3.61]
SES_2018 Pan	5.09	5.71	6	3.83	5.58	6	19.1%	1.26 [-5.13, 7.65]
**Subtotal (95% CI)**			**22**			**23**	**100.0%**	**0.65 [-2.15, 3.44]**
Heterogeneity: Tau² = 0.00; Chi² = 0.04, df = 1 (P = 0.83); I² = 0%								
Test for overall effect: Z = 0.45 (P = 0.65)								
**4.1.9 MCS vs. control**								
MCS_2006 Brown	10	8.9	4	1.9	7.75	4	7.8%	8.10 [-3.47, 19.67]
MCS_2008 Huang	6.33	5.24	12	2.57	5.43	12	33.5%	3.76 [-0.51, 8.03]
MCS_2016 Levy	4.8	5.5	94	4.6	5.4	58	58.6%	0.20 [-1.58, 1.98]
**Subtotal (95% CI)**			**110**			**74**	**100.0%**	**2.01 [-1.42, 5.45]**
Heterogeneity: Tau² = 4.42; Chi² = 3.81, df = 2 (P = 0.15); I² = 47%								
Test for overall effect: Z = 1.15 (P = 0.25)								
**4.1.10 atDCS vs. ctDCS**								
tDCS_2011 Hesse	15.4	16.73	32	15.6	13.13	32	100.0%	-0.20 [-7.57, 7.17]
**Subtotal (95% CI)**			**32**			**32**	**100.0%**	**-0.20 [-7.57, 7.17]**
Heterogeneity: Not applicable								
Test for overall effect: Z = 0.05 (P = 0.96)								
**4.1.11 atDCS vs. control**								
tDCS_2011 Hesse	15.4	16.73	32	14.3	15.38	32	39.9%	1.10 [-6.77, 8.97]
tDCS_2015 Triccas	7.18	16.34	12	7.09	16.3	11	13.9%	0.09 [-13.26, 13.44]
tDCS_2016 Allman	9.28	15.18	11	6.73	16.86	13	15.0%	2.55 [-10.27, 15.37]
tDCS_2019 Bornheim	14.44	14.48	25	5.77	17.49	25	31.2%	8.67 [-0.23, 17.57]
**Subtotal (95% CI)**			**80**			**81**	**100.0%**	**3.54 [-1.43, 8.51]**
Heterogeneity: Tau² = 0.00; Chi² = 1.92, df = 3 (P = 0.59); I² = 0%								
Test for overall effect: Z = 1.40 (P = 0.16)								
**4.1.12 ctDCS vs. control**								
tDCS_2011 Hesse	15.6	13.13	32	14.3	15.38	32	93.8%	1.30 [-5.71, 8.31]
tDCS_2014 Fusco	8.34	28.8	7	9.9	23.04	7	6.2%	-1.56 [-28.88, 25.76]
**Subtotal (95% CI)**			**39**			**39**	**100.0%**	**1.12 [-5.66, 7.91]**
Heterogeneity: Tau² = 0.00; Chi² = 0.04, df = 1 (P = 0.84); I² = 0%								
Test for overall effect: Z = 0.32 (P = 0.75)								
**4.1.13 LFrTMS vs. drTMS**								
rTMS_2018 Long	6.29	5.2	21	12.67	3.44	21	100.0%	-6.38 [-9.05, -3.71]
**Subtotal (95% CI)**			**21**			**21**	**100.0%**	**-6.38 [-9.05, -3.71]**
Heterogeneity: Not applicable								
Test for overall effect: Z = 4.69 (P < 0.00001)								

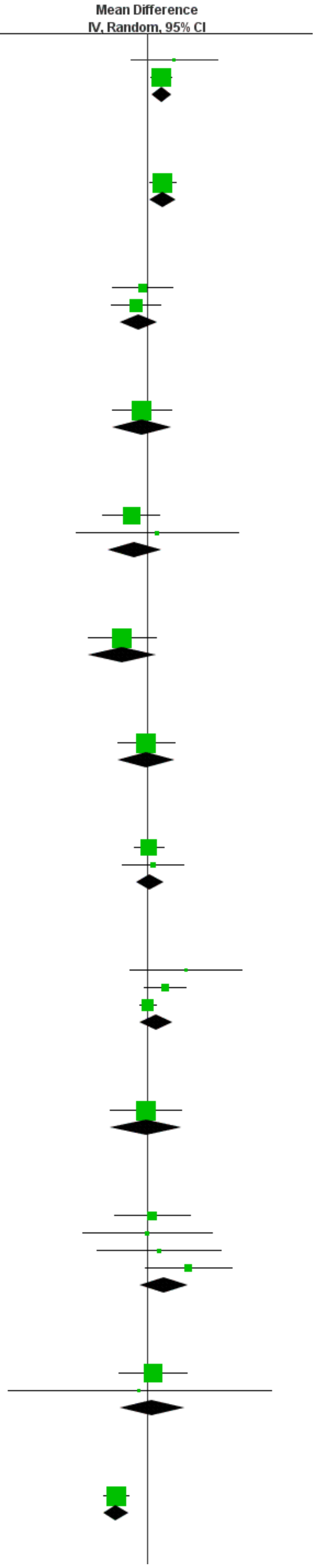

**4.1.14 drTMS vs. control**								
rTMS_2018 Long	12.67	3.44	21	4.85	3.21	20	97.5%	7.82 [5.78, 9.86]
rTMS_2020 Chiu	2.34	37.21	14	8	33.49	15	2.5%	-5.66 [-31.49, 20.17]
**Subtotal (95% CI)**			**35**			**35**	**100.0%**	**7.48 [3.35, 11.61]**
Heterogeneity: Tau² = 3.48; Chi² = 1.04, df = 1 (P = 0.31); I² = 4%								
Test for overall effect: Z = 3.55 (P = 0.0004)								
**4.1.15 LFrTMS vs. HFrTMS**								
rTMS_2016 Du	22.56	12.73	23	23.07	14.44	23	100.0%	-0.51 [-8.38, 7.36]
**Subtotal (95% CI)**			**23**			**23**	**100.0%**	**-0.51 [-8.38, 7.36]**
Heterogeneity: Not applicable								
Test for overall effect: Z = 0.13 (P = 0.90)								
**4.1.16 LFrTMS vs. iTBS**								
rTMS vs. iTBS vs, R_2018 Watanabe	25.11	9.92	7	18.85	10.89	8	100.0%	6.26 [-4.27, 16.79]
**Subtotal (95% CI)**			**7**			**8**	**100.0%**	**6.26 [-4.27, 16.79]**
Heterogeneity: Not applicable								
Test for overall effect: Z = 1.16 (P = 0.24)								
**4.1.17 LFrTMS vs. control**								
rTMS vs. iTBS vs, R_2018 Watanabe	25.11	9.92	7	13.86	4.39	6	16.5%	11.25 [3.10, 19.40]
rTMS_2016 Du	22.56	12.73	23	14.36	12.19	23	18.9%	8.20 [1.00, 15.40]
rTMS_2018 Harvey	7.1	13.44	132	7.6	13.37	67	29.9%	-0.50 [-4.44, 3.44]
rTMS_2018 Long	6.29	5.2	21	4.85	3.21	20	34.6%	1.44 [-1.19, 4.07]
**Subtotal (95% CI)**			**183**			**116**	**100.0%**	**3.76 [-0.57, 8.09]**
Heterogeneity: Tau² = 12.30; Chi² = 9.52, df = 3 (P = 0.02); I² = 68%								
Test for overall effect: Z = 1.70 (P = 0.09)								
**4.1.18 HFrTMS vs. control**								
rTMS_2010 Chang	8	8.3	18	4.67	9.66	10	17.3%	3.33 [-3.78, 10.44]
rTMS_2016 Du	23.07	14.44	23	14.36	12.19	23	14.6%	8.71 [0.99, 16.43]
rTMS_2017 Guan	20.4	7.6	21	15.2	3.5	21	68.1%	5.20 [1.62, 8.78]
**Subtotal (95% CI)**			**62**			**54**	**100.0%**	**5.39 [2.44, 8.34]**
Heterogeneity: Tau² = 0.00; Chi² = 1.04, df = 2 (P = 0.59); I² = 0%								
Test for overall effect: Z = 3.58 (P = 0.0003)								
**4.1.19 iTBS vs. control**								
rTMS vs. iTBS vs, R_2018 Watanabe	18.85	10.89	8	13.86	4.39	6	100.0%	4.99 [-3.33, 13.31]
**Subtotal (95% CI)**			**8**			**6**	**100.0%**	**4.99 [-3.33, 13.31]**
Heterogeneity: Not applicable								
Test for overall effect: Z = 1.17 (P = 0.24)								

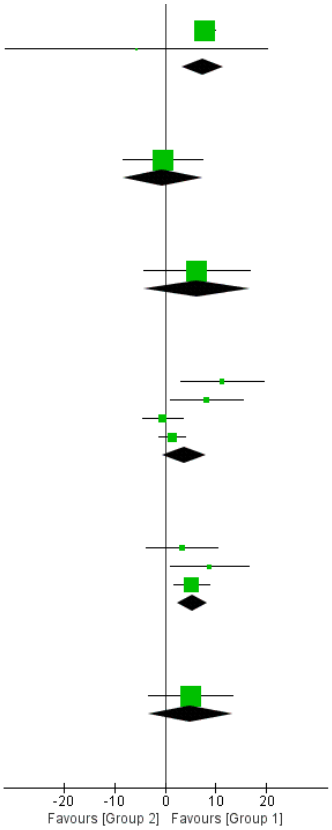

Test for subgroup differences: Chi² = 65.09, df = 18 (P < 0.00001), I² = 72.3%

F. Pair-wise sensitivity analysis

FMA-UE LFU LFrTMS sensitivity

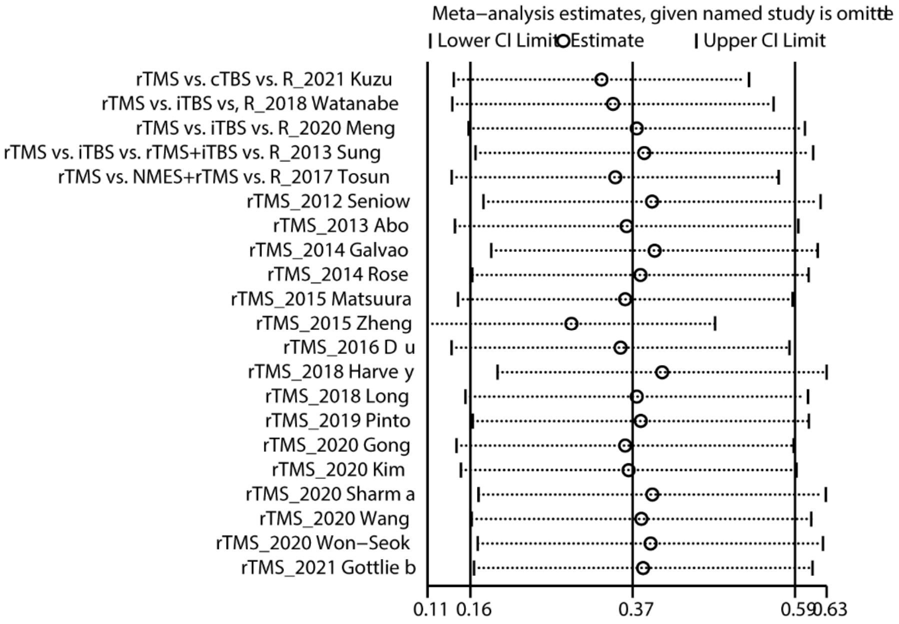

FMA-UE EOT cNMES sensitivity

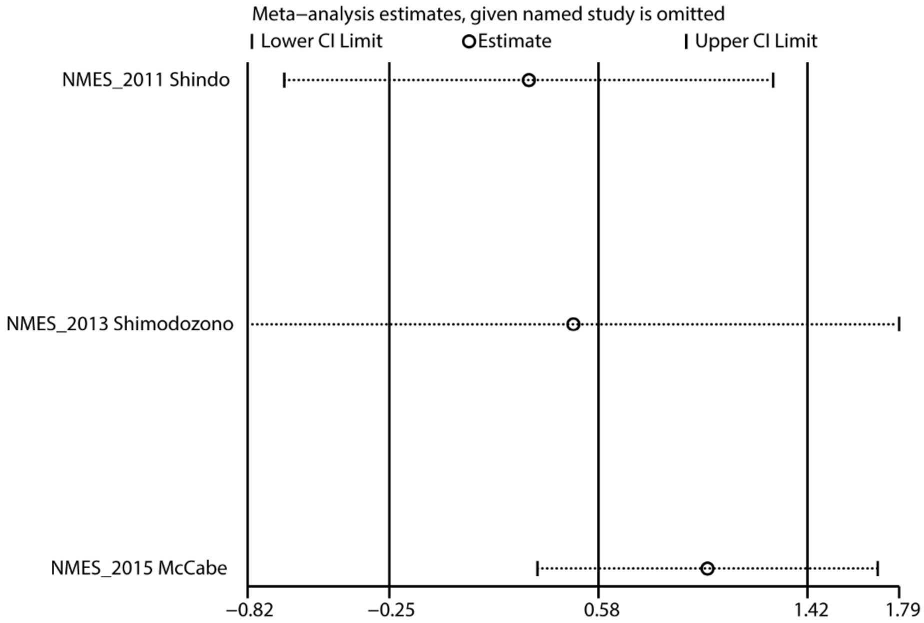

FMA-UE 1 month MCS sensitivity

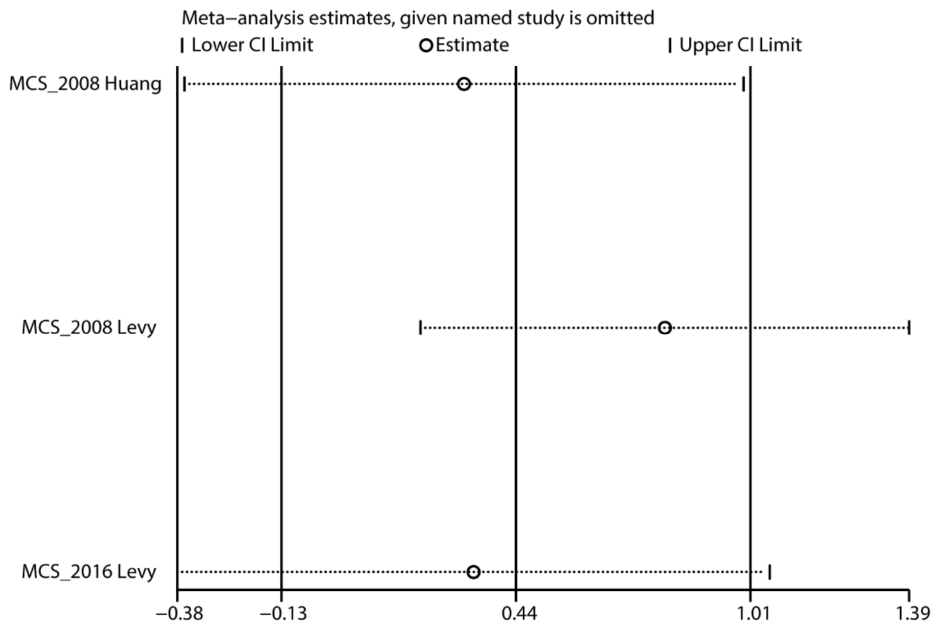

FMA-UE 1 month LFrTMS sensitivity

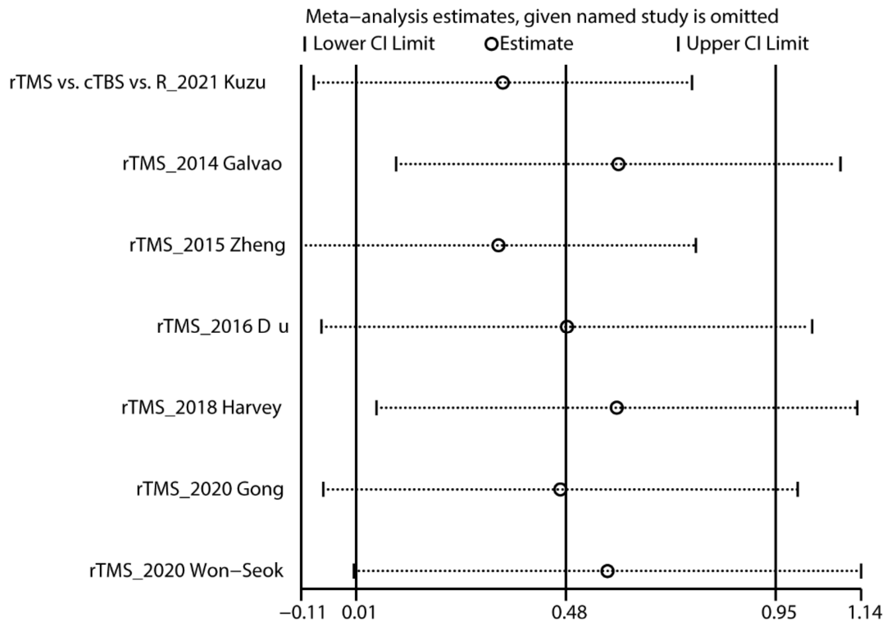

FMA-UE 3 month LFrTMS sensitivity

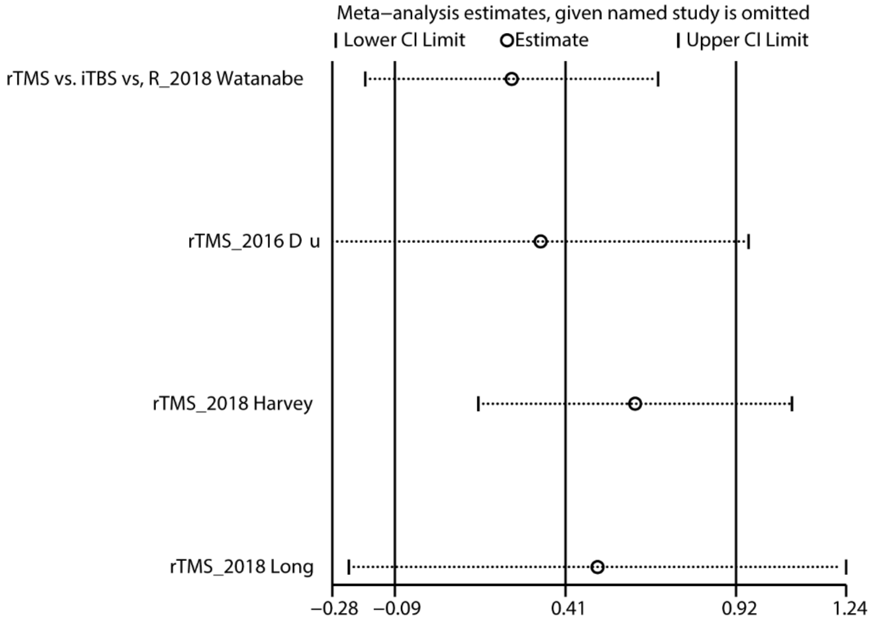

G. The details of GRADE for pair-wise meta-analysis and network meta-analysis

FMA-UE LFU	Direct	Indirect	Network
VNS vs. control	Moderate *	Not estimable ¶	Moderate
MCS vs. control	Very low **, #	Not estimable ¶	Very low
SES vs. control	Low *, #	Very low &, §	Low
ENMES vs. control	Very low *, ##	Low &	Low
ENMES vs. SES	Moderate #	Very low &	Moderate
cNMES vs. control	Low *, $	Very low &	Low
atDCS vs. control	Moderate $	Low &	Moderate
ctDCS vs. control	High	Very low &, §	High
dtDCS vs. control	High	Not estimable ¶	High
ENMES vs. cNMES	Moderate *	Very low &, #	Moderate
FES vs. cNMES	Low *, $	Low %, #	Low
cNMES vs. SES	Moderate *	Low &	Moderate
LFrTMS vs. control	Moderate $	Very low &, §	Moderate
LFrTMS vs. LFrTMS+atDCS	Moderate *	Moderate %	Moderate
LFrTMS vs. LFrTMS+ctDCS	Moderate *	Moderate %	Moderate
LFrTMS+atDCS vs. control	Moderate *	Low %, §	Moderate
LFrTMS+ctDCS vs. control	Moderate *	Moderate %	Moderate
LFrTMS+atDCS vs. LFrTMS+ctDCS	Moderate *	Moderate %	Moderate
iTBS+atDCS vs. control	Low *, #	Not estimable ¶	Low
SES+dtDCS vs. control	Moderate *	Not estimable ¶	Moderate
drTMS vs. control	Moderate #	Low %, §	Moderate
iTBS vs. control	High	Moderate %	High
HFrTMS vs. control	Moderate *	Low &	Moderate
LFrTMS vs. rPMS	Low *, #	Low &	Low
LFrTMS vs. HFrTMS	Low *, #	Low %, #	Low
LFrTMS vs. ctDCS	Low *, #	Moderate %	Moderate
LFrTMS vs. cTBS	High	Moderate %	High
cTBS vs. control	High	Moderate %	High
LFrTMS vs. drTMS	High	Moderate %	High
iTBS+LFrTMS vs. control	Moderate #	Low &	Moderate
iTBS+LFrTMS vs. LFrTMS	Moderate #	Very low &, §	Moderate
LFrTMS vs. iTBS	Moderate #	Very low &, #	Moderate
LFrTMS+cNMES vs. control	Very low *, ##	Very low &	Very low
LFrTMS+cNMES vs. LFrTMS	Very low *, ##	Very low &	Very low
iTBS+LFrTMS vs.iTBS	Low ##	Low &	Low
ctDCS vs. atDCS	Low #, $	Low %, ^	Low
rPMS vs. control	Low *, #	Very low &, #	Low
taVNS vs. control	Moderate *	Not estimable ¶	Moderate

FES vs. SES	Moderate #	Low &	Moderate
FES vs. control	Not estimable	Very low &	Very low

FMA-UE EOT	Direct	Indirect	Network
VNS vs. control	Moderate *	Not estimable ¶	Moderate
taVNS vs. control	Moderate *	Not estimable ¶	Moderate
ENMES vs. control	Very low *, ##	Very low &, §	Very low
cNMES vs. control	Very low *, #, $	Very low &, §	Low $$
ENMES vs. cNMES	Low *, #	Very low &, §, #	Low
ENMES vs. SES	Moderate #	Very low &, §, #	Moderate
cNMES vs. SES	Moderate #	Very low &	Moderate
SES vs. control	Low #, $	Very low &	Low
MCS vs. control	Low **	Not estimable ¶	Low
FES vs. cNMES	Very low **, $	Moderate %	Moderate
FES vs. SES	Moderate #	Very low &	High $$
dtDCS vs. control	High	Not estimable ¶	High
atDCS vs. ctDCS	High	Moderate ^	High
atDCS vs. control	High	High	High
ctDCS vs. LFrTMS	Low *, #	Moderate %	Moderate
ctDCS vs. control	High	Low &	High
LFrTMS vs. drTMS	High	Very low %, ##	High
drTMS vs. control	High	Moderate %	High
HFrTMS vs. LFrTMS	Low *, #	Low &	Low
iTBS vs. LFrTMS	Low ##	Moderate %	Moderate
cTBS vs. LFrTMS	Moderate #	Moderate %	Moderate
rPMS vs. LFrTMS	Low *, #	Very low &, §	Low
cNMES+LFrTMS VS. LFrTMS	Very low *, ##	Very low &	Very low
cNMES+LFrTMS VS. control	Very low *, ##	Very low &	Very low
LFrTMS+atDCS vs. LFrTMS	Moderate *	Low &	Moderate
LFrTMS+ctDCS vs. LFrTMS	Moderate *	Moderate %	Moderate
iTBS+LFrTMS VS. LFrTMS	Moderate #	Low &	Moderate
LFrTMS vs. control	Moderate #	Very low &	Moderate
HFrTMS vs. control	Low *, #	Low &	Low
iTBS+LFrTMS VS. iTBS	Low ##	Low %, #	Low
iTBS vs. control	Moderate #	Low &	Moderate
cTBS vs. control	Moderate #	Moderate %	Moderate
rPMS vs. control	Low *, #	Very low &, §	Low
SES+dtDCS vs. control	Moderate *	Not estimable ¶	Moderate
LFrTMS+atDCS vs. LFrTMS+ctDCS	Moderate *	Low &	Moderate
LFrTMS+atDCS vs. control	Low *, #	Moderate %	Moderate
LFrTMS+ctDCS vs. control	Moderate *	Low &	Moderate
iTBS+atDCS vs. control	Moderate #	Not estimable ¶	Moderate
iTBS+LFrTMS vs. control	Moderate #	Low &	Moderate

FES vs. control	Not estimable	Very low &, §	Very low

FMA-UE 1 month	Direct	Indirect	Network
VNS vs. control	Moderate #	Not estimable ¶	Moderate
taVNS vs. control	Moderate *	Not estimable ¶	Moderate
ENMES vs. cNMES	Low *, #	Low &	Low
ENMES vs. SES	Moderate #	Low &	Moderate
cNMES vs. FES	Low *, #	Not estimable ¶	Low
cNMES vs. SES	Low *, #	Low &	Low
SES vs. control	Moderate #	Not estimable ¶	Moderate
MCS vs. control	Very low **, $	Not estimable ¶	Very low
dtDCS vs. control	Low *, #	Not estimable ¶	Low
atDCS vs. ctDCS	High	Low %, #	High
atDCS vs. control	Moderate #	Moderate %	Moderate
ctDCS vs. LFrTMS	Low *, #	Very low &, #, §	Low
ctDCS vs. control	Moderate #	Very low &, #	Moderate
drTMS vs. control	Low ##	Not estimable ¶	Low
LFrTMS vs. HFrTMS	Low *, #	Very low &, #, §	Low
LFrTMS vs. cTBS	High	Low &	High
LFrTMS+atDCS vs. LFrTMS	Moderate *	Low &	Moderate
LFrTMS+ctDCS vs. LFrTMS	Moderate *	Low &	Moderate
LFrTMS vs. control	Low #, $	Very low &, #	Low
HFrTMS vs. control	High	Low &	High
cTBS vs. control	High	Low &	High
LFrTMS+atDCS vs. LFrTMS+ctDCS	Moderate *	Moderate %	Moderate
LFrTMS+atDCS vs. control	Moderate *	Low &	Moderate
LFrTMS+ctDCS vs. control	Moderate *	Low &	Moderate
FES vs. control	Not estimable	Not estimable ¶	Very low
ENMES vs. control	Not estimable	Moderate %	Moderate
cNMES vs. control	Not estimable	Low &	Low

FMA-UE 3 month	Direct	Indirect	Network
VNS vs. control	High	Not estimable ¶	High
taVNS vs. control	Low *, #	Not estimable ¶	Low
ENMES vs. cNMES	Moderate *	Low &	Moderate
ENMES vs. SES	Low *, #	Low &	Low
ENMES vs. control	Low *, #	Low &	Low
cNMES vs. FES	Low *, #	Not estimable ¶	Low
cNMES vs. SES	Low *, #	Very low &, #	Low
SES vs. control	Moderate *	Low &	Moderate
MCS vs. control	Very low **, &	Not estimable ¶	Very low
atDCS vs. ctDCS	Moderate #	Very low %, ##	Moderate
atDCS vs. control	High	Moderate %	High

ctDCS vs. control	Moderate #	Moderate %	Moderate
LFrTMS vs. drTMS	High	Very low %, ##	High
drTMS vs. control	High	Moderate %	High
LFrTMS vs. HFrTMS	Low *, #	Moderate %	Moderate
LFrTMS vs. iTBS	Low *, #	Low &	Low
LFrTMS vs. control	Moderate &	Low &	Moderate
HFrTMS vs. control	Moderate *	Low &	Moderate
iTBS vs. control	Low *, #	Low &	Low
Cnmes	Not estimable	Low &	Low

* Limitations (risk of bias); ** Severe limitations (risk of bias); # Imprecision; ## Severe imprecision; $ Inconsistency; ¶ Cannot be estimated because the drug was not connected in a loop in the evidence network; % Contributing direct evidence of moderate quality; & Contributing direct evidence of low or very low quality; § Indirectness because of questionable comparability of trial populations to target population of NMA or because of intransitivity; ^ Incoherence, $$ Greater precision

H. Network results for ARAT

net plot

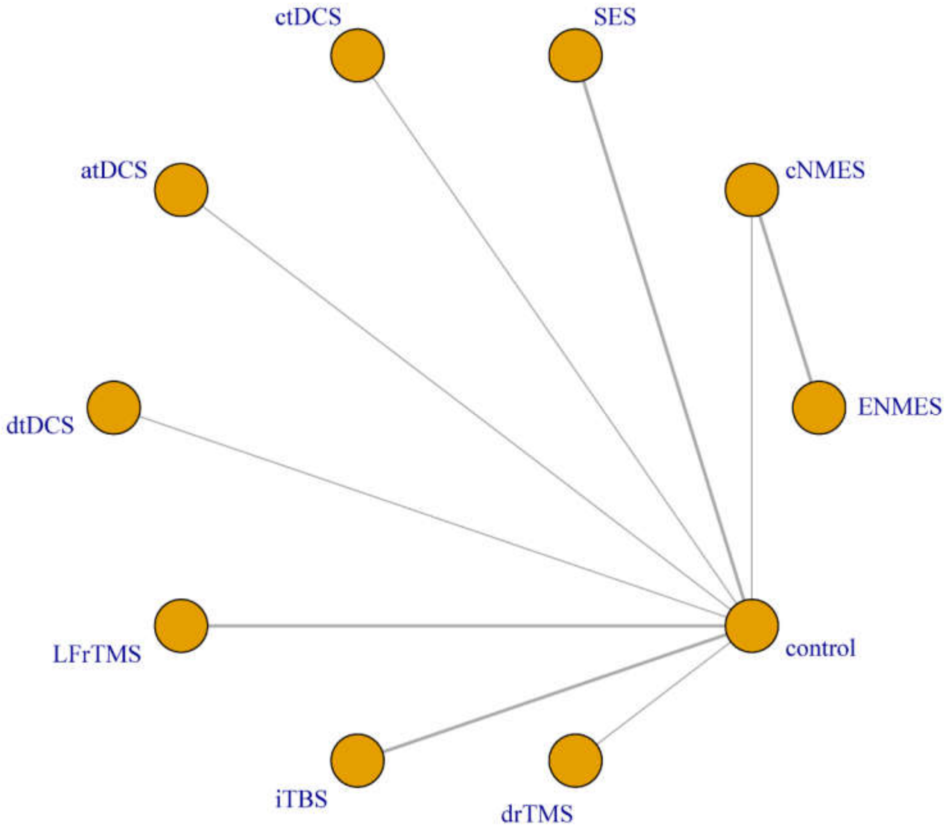

forest plot

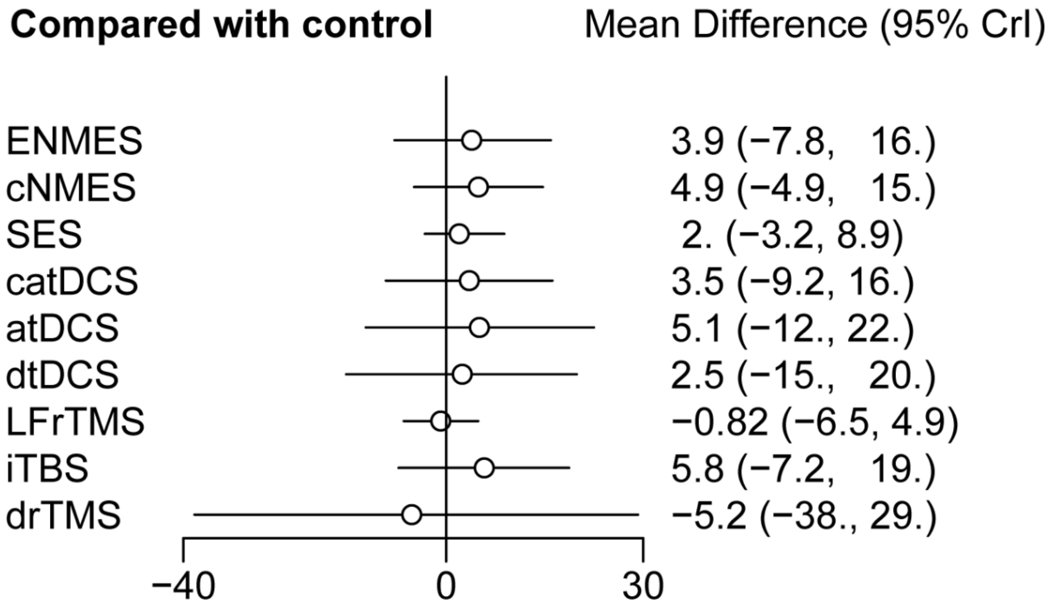

SUCRA

Neurostimulation	SUCRA (%)
ENMES	57.37
cNMES	65.36
SES	50.56
ctDCS	55.57
atDCS	50.68
dtDCS	50.17
LFrTMS	28.02
drTMS	33.01
iTBS	65.92
control	33.35

heterogeneity

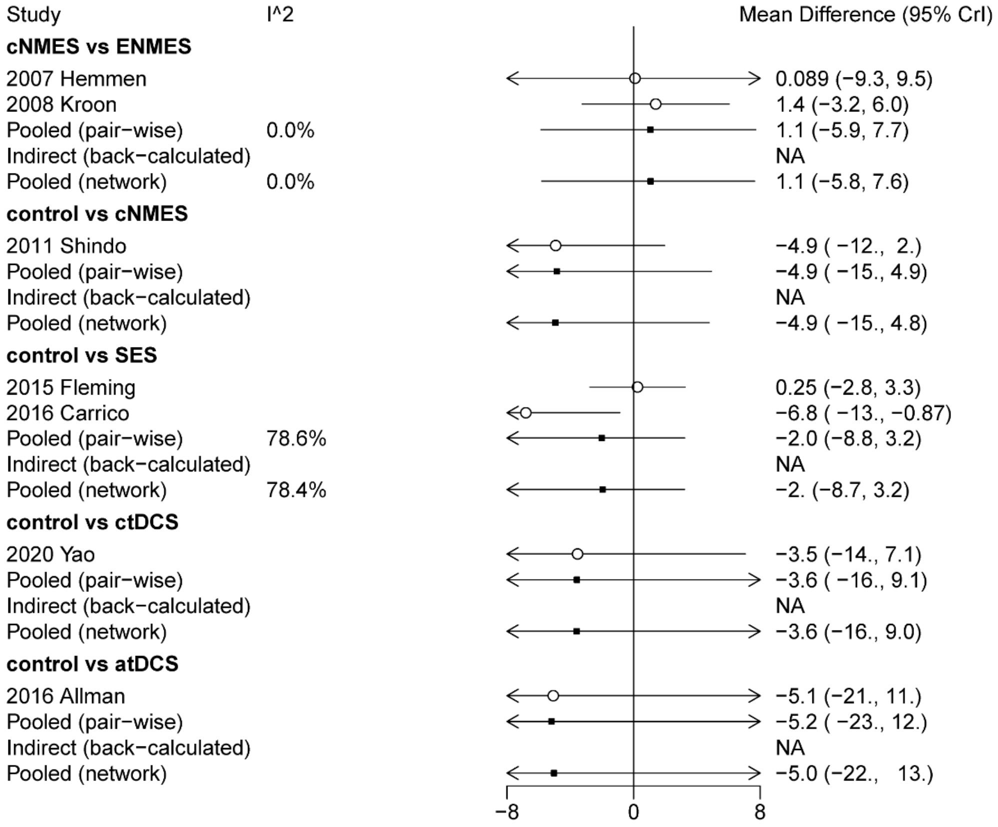

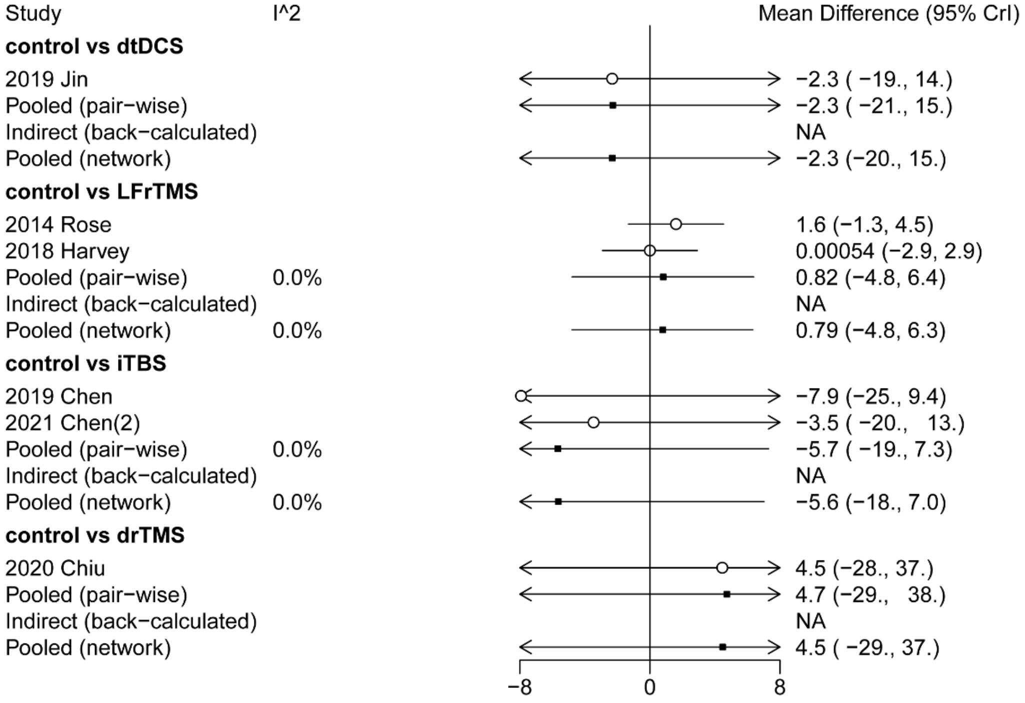

I. Network results for BBT

net plot

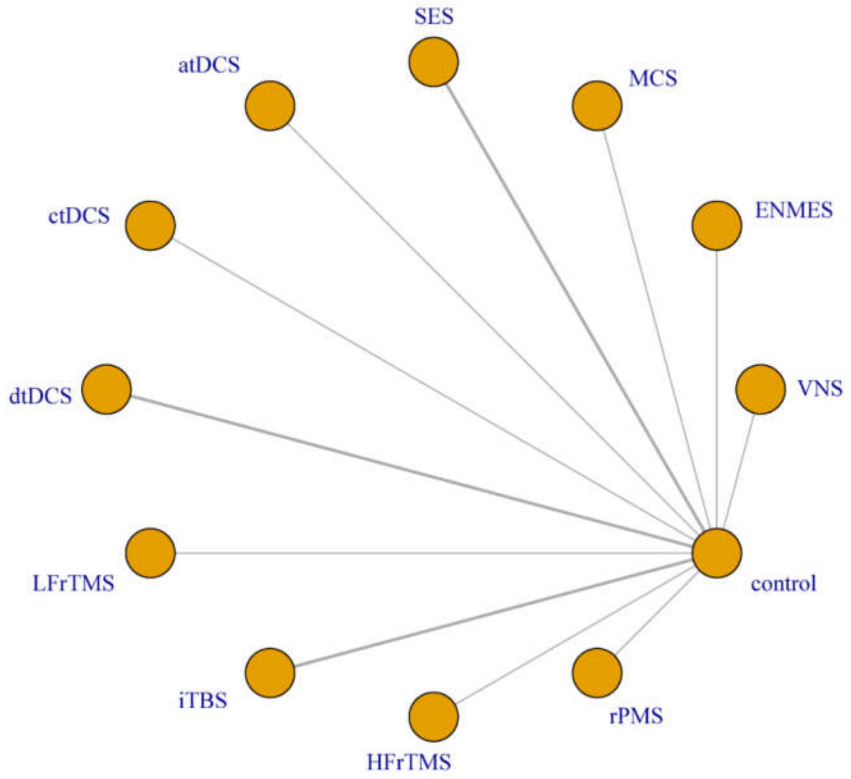

forest plot

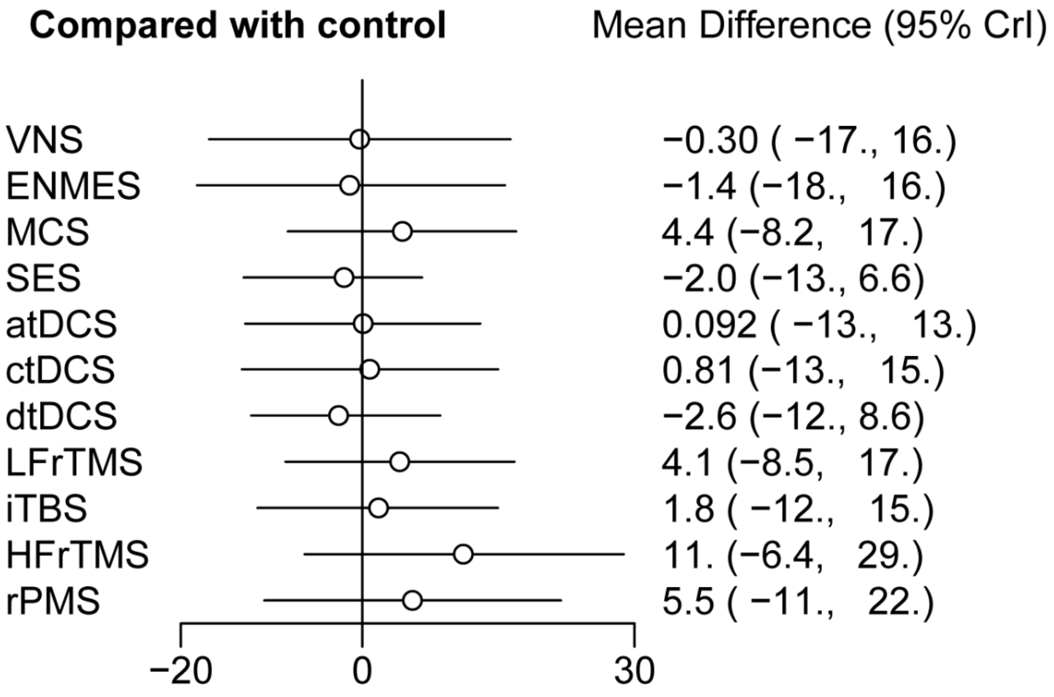

SUCRA

**Neurostimulation**	**SUCRA (%)**
VNS	42.25
ENMES	38.10
MCS	65.51
SES	30.01
atDCS	42.85
ctDCS	46.60
dtDCS	28.49
LFrTMS	64.08
HFrTMS	82.97
iTBS	51.43
rPMS	66.40
control	41.32

heterogeneity

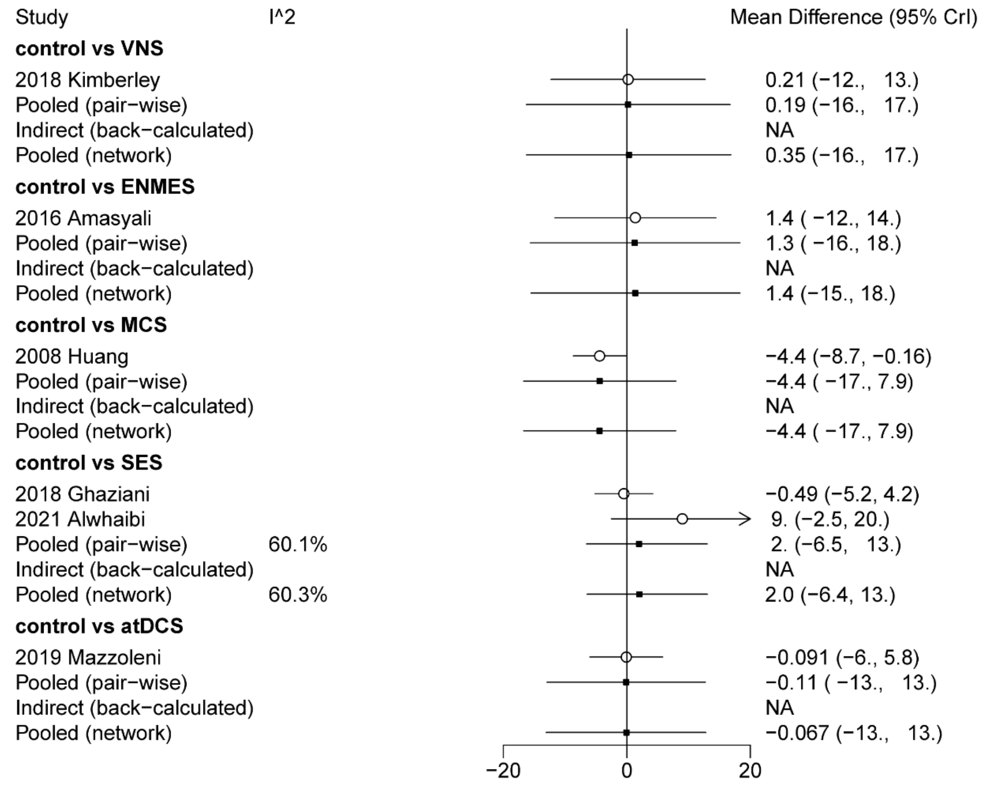

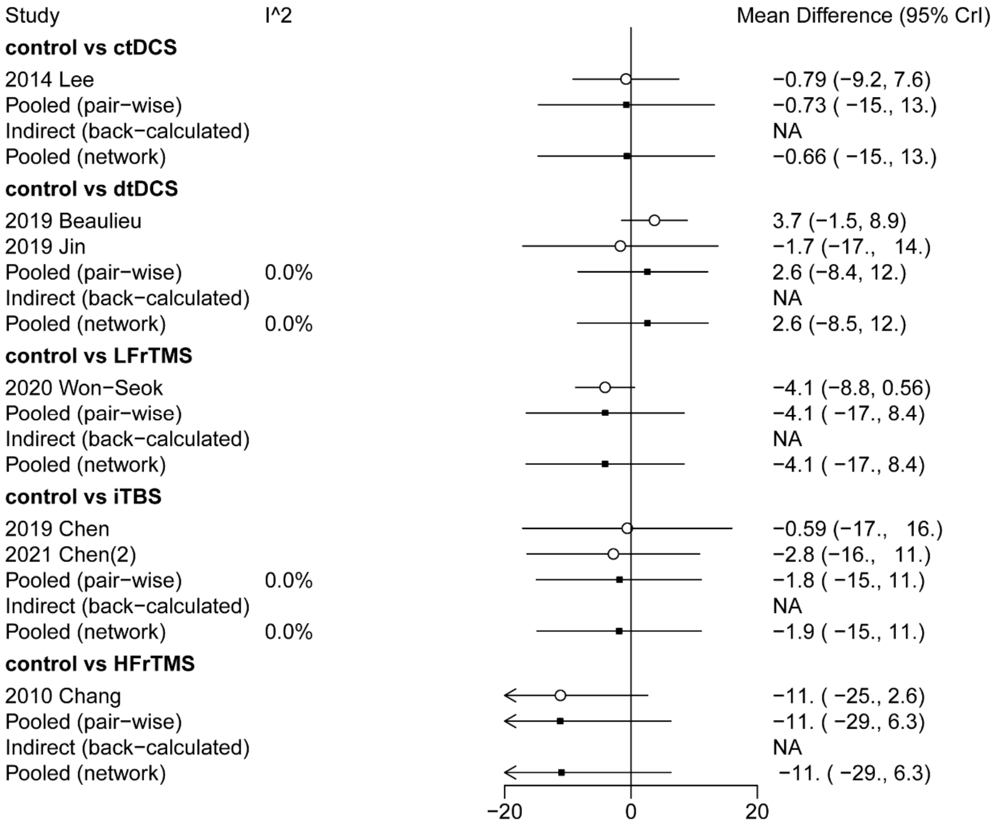

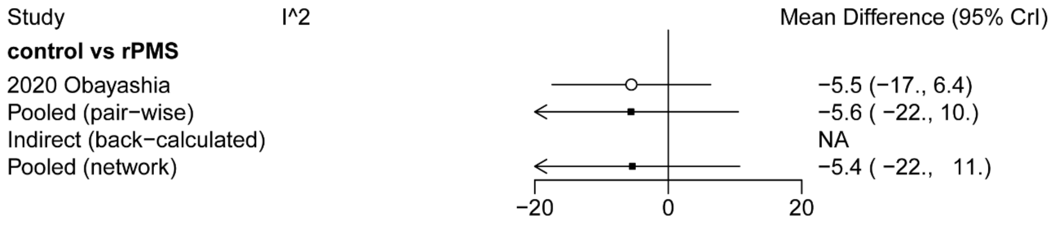

Study
I^2
Mean Difference (95% CrI)
control vs rPMS
2020 Obayashia
Pooled (pair−wise)
Indirect (back−calculated)
Pooled (network)
−5.5 (−17., 6.4)
−5.6 (−22., 10.)
NA
−5.4 (−22., 11.)
−20
0
20

J. Network meta-regression

Outcomes	Models	covariates	Model fit			Ratio	I^2 (%)
			Dbar	pD	DIC		
FMA-UE LFU	Random consistent model	-	175.38	137.18	312.56	0.9182	0
	Regression model	Mean age	175.02	138.46	313.48	0.9164	0
		Percentage of female	175.42	138.26	313.68	0.9184	0
		Mean FMA-UE baseline	175.08	138.22	313.30	0.9166	0
		Percentage of hemorrhagic stroke	175.44	138.17	313.60	0.9185	0
		Sample size	176.06	136.55	312.61	0.9218	0
		Mean time since stroke	173.05	136.99	310.04	0.906	0
FMA-UE EOT	Random consistent model	-	161.36	125.42	286.78	0.9065	0
	Regression model	Mean age	162.99	129.91	289.90	0.9157	0
		Percentage of female	162.86	126.84	289.70	0.9149	0
		Mean FMA-UE baseline	162.85	126.95	289.81	0.9149	0
		Percentage of hemorrhagic stroke	162.81	127.02	289.84	0.9147	0
		Sample size	163.07	124.16	287.23	0.9161	0
		Mean time since stroke	160.60	126.52	287.13	0.9023	0
FMA-UE EOT	Random consistent model	-	52.00	48.18	100.18	0.9629	0
	Regression model	Mean age	52.86	48.68	101.54	0.9789	0
		Percentage of female	52.67	48.05	100.72	0.9754	0
		Mean FMA-UE baseline	52.59	48.65	101.24	0.9738	0

		Percentage of hemorrhagic stroke	52.12	48.84	100.96	0.9652	0
		Sample size	52.46	48.76	101.22	0.9715	0
		Mean time since stroke	51.20	48.52	99.72	0.9481	0
FMA-UE EOT	Random consistent model	-	52.89	41.67	94.56	0.9616	0
	Regression model	Mean age	53.28	43.54	96.82	0.9687	0
		Percentage of female	54.25	44.11	98.36	0.9864	0.5
		Mean FMA-UE baseline	54.65	44.09	98.73	0.9936	1
		Percentage of hemorrhagic stroke	53.38	44.43	97.81	0.9706	0
		Sample size	51.74	41.63	93.37	0.9407	0
		Mean time since stroke	54.22	43.64	97.86	0.9858	0.4

K. Network global consistent analysis and sensitivity analysis

	Consistent model (fixed)			Consistent model (random)			Inconsistent model (UME)		
	Dbar	pD	DIC	Dbar	pD	DIC	Dbar	pD	DIC
FMA-UE LFU	210.11	110.07	320.18	178.38	139.18	317.56	179.15	145.48	324.63
FMA-UE EOT	189.98	104.06	294.04	164.36	127.42	291.78	165.07	133.26	298.33
FMA-UE 1 month	62.94	39.99	102.93	53.00	49.18	102.18	53.23	50.36	103.60
FMA-UE 3 month	56.67	39.01	95.68	52.89	41.67	94.56	53.71	46.56	100.27
ARAT	28.01	21.94	49.94	25.28	23.95	49.23	25.35	24.02	49.36
BBT	28.75	25.03	53.77	27.13	26.24	53.37	27.10	26.13	53.23

L. Network local consistent analysis and heterogeneity analysis of FMA-UE LFU

Consistency

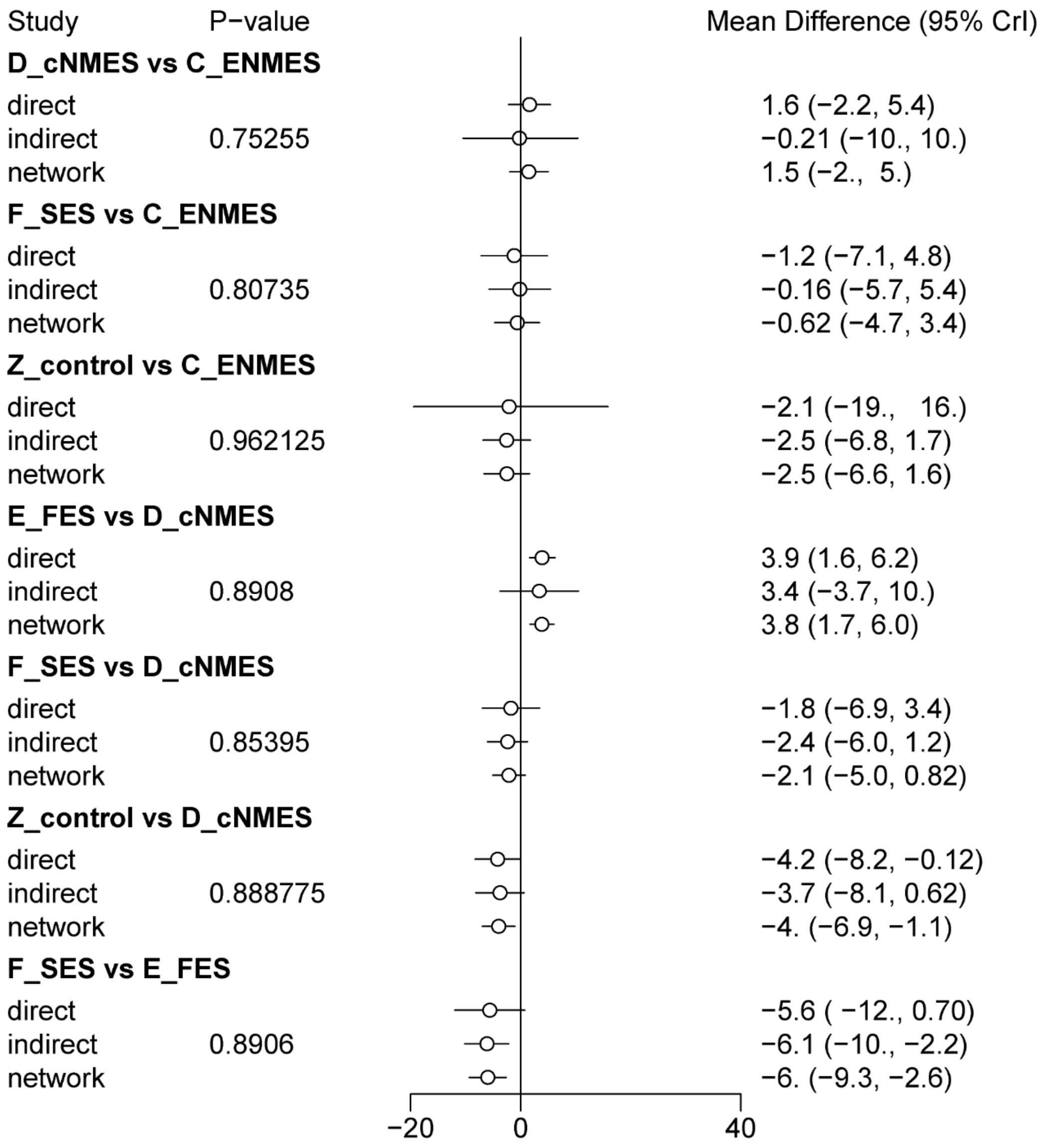

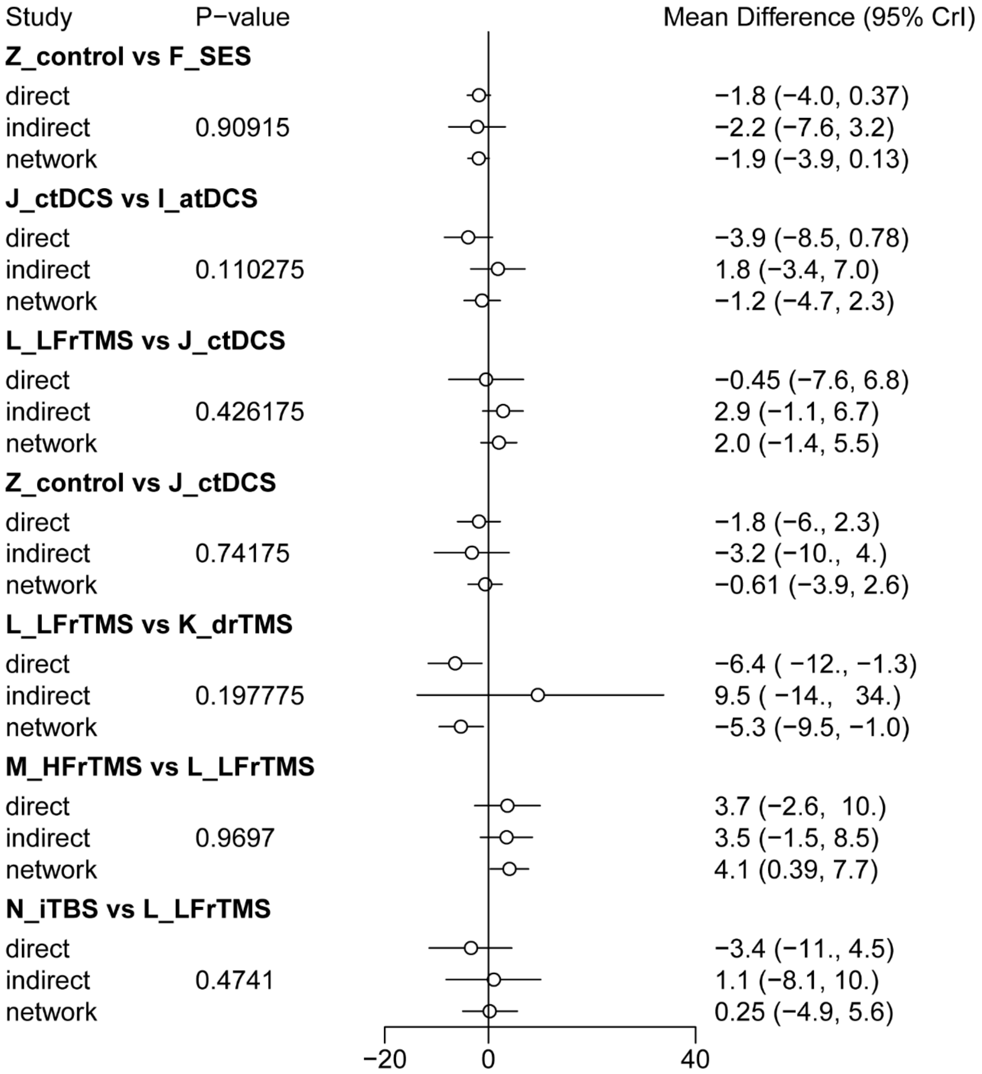
Study
P−value
Mean Difference (95% CrI)
Z_control vs F_SES
direct −1.8 (−4.0, 0.37)
indirect 0.90915 −2.2 (−7.6, 3.2)
network −1.9 (−3.9, 0.13)
J_ctDCS vs I_atDCS
direct −3.9 (−8.5, 0.78)
indirect 0.110275 1.8 (−3.4, 7.0)
network −1.2 (−4.7, 2.3)
L_LFrTMS vs J_ctDCS
direct −0.45 (−7.6, 6.8)
indirect 0.426175 2.9 (−1.1, 6.7)
network 2.0 (−1.4, 5.5)
Z_control vs J_ctDCS
direct −1.8 (−6., 2.3)
indirect 0.74175 −3.2 (−10., 4.)
network −0.61 (−3.9, 2.6)
L_LFrTMS vs K_drTMS
direct −6.4 (−12., −1.3)
indirect 0.197775 9.5 (−14., 34.)
network −5.3 (−9.5, −1.0)
M_HFrTMS vs L_LFrTMS
direct 3.7 (−2.6, 10.)
indirect 0.9697 3.5 (−1.5, 8.5)
network 4.1 (0.39, 7.7)
N_iTBS vs L_LFrTMS
direct −3.4 (−11., 4.5)
indirect 0.4741 1.1 (−8.1, 10.)
network 0.25 (−4.9, 5.6)
−20
0
40

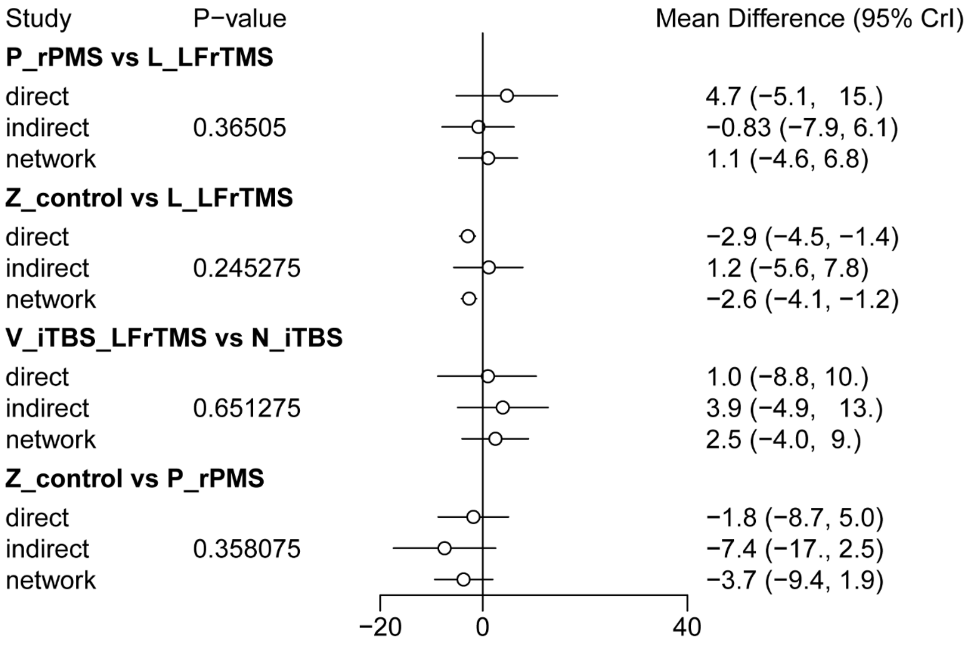

Heterogeneity

	t1	t2	i2.pair	i2.cons	incons.p
1	A_VNS	Z_control	0.00000	0.000000	NA
2	B_taVNS	Z_control	0.00000	0.000000	NA
3	C_ENMES	D_cNMES	0.00000	0.000000	0.83385139
4	C_ENMES	F_SES	0.00000	0.000000	0.87695961
5	C_ENMES	Z_control	NA	0.000000	0.96213085
6	D_cNMES	E_FES	86.25486	83.304080	0.92150073
7	D_cNMES	F_SES	0.00000	0.000000	0.85738013
8	D_cNMES	Z_control	44.42519	26.043547	0.88867514
9	E_FES	F_SES	NA	0.000000	0.89274899
10	F_SES	Z_control	22.54604	7.170121	0.86914046
11	G_MCS	Z_control	39.22036	40.242807	NA
12	H_dtDCS	Z_control	0.00000	0.000000	NA
13	I_atDCS	J_ctDCS	31.77805	69.281514	0.09268663
14	I_atDCS	Z_control	49.27672	59.067813	NA
15	J_ctDCS	L_LFrTMS	NA	0.000000	0.42263151
16	J_ctDCS	Z_control	0.00000	0.000000	0.42037394
17	K_drTMS	L_LFrTMS	NA	0.000000	0.52973006
18	K_drTMS	Z_control	27.95177	24.344698	NA
19	L_LFrTMS	M_HFrTMS	0.00000	0.000000	0.84740556
20	L_LFrTMS	N_iTBS	0.00000	0.000000	0.59051065
21	L_LFrTMS	O_cTBS	NA	NA	NA
22	L_LFrTMS	P_rPMS	NA	0.000000	0.37275610
23	L_LFrTMS	Q_cNMES_LFrTMS	NA	NA	NA
24	L_LFrTMS	S_LFrTMS_atDCS	NA	NA	NA

25	L_LFrTMS	T_LFrTMS_ctDCS	NA	NA	NA
26	L_LFrTMS	V_iTBS_LFrTMS	0.00000	0.000000	NA
27	L_LFrTMS	Z_control	61.14538	58.399219	0.59958757
28	M_HFrTMS	Z_control	0.00000	0.000000	NA
29	N_iTBS	V_iTBS_LFrTMS	NA	0.000000	0.74595269
30	N_iTBS	Z_control	0.00000	0.000000	NA
31	O_cTBS	Z_control	NA	NA	NA
32	P_rPMS	Z_control	0.00000	0.000000	0.32703730
33	Q_cNMES_LFrTMS	Z_control	NA	NA	NA
34	R_SES_dtDCS	Z_control	NA	NA	NA
35	S_LFrTMS_atDCS	T_LFrTMS_ctDCS	NA	NA	NA
36	S_LFrTMS_atDCS	Z_control	NA	NA	NA
37	T_LFrTMS_ctDCS	Z_control	NA	NA	NA
38	U_iTBS_atDCS	Z_control	NA	NA	NA
39	V_iTBS_LFrTMS	Z_control	0.00000	0.000000	NA
Global I-squared: ------------------------ i2.pair = 40.92401; i2.cons = 37.34933					

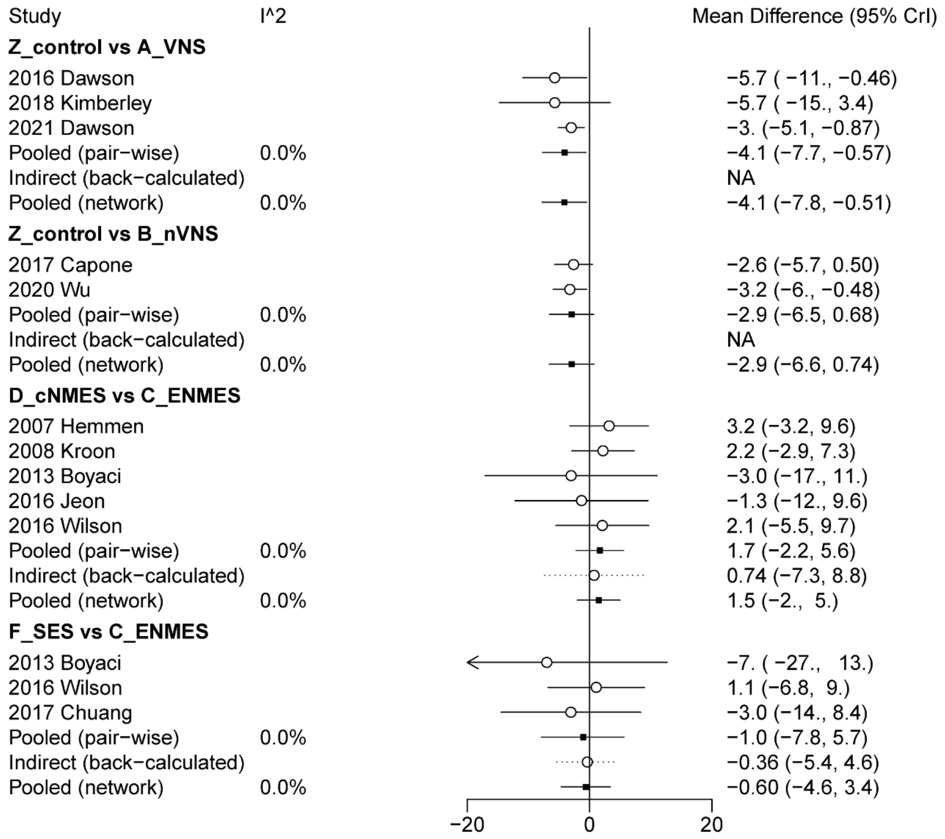

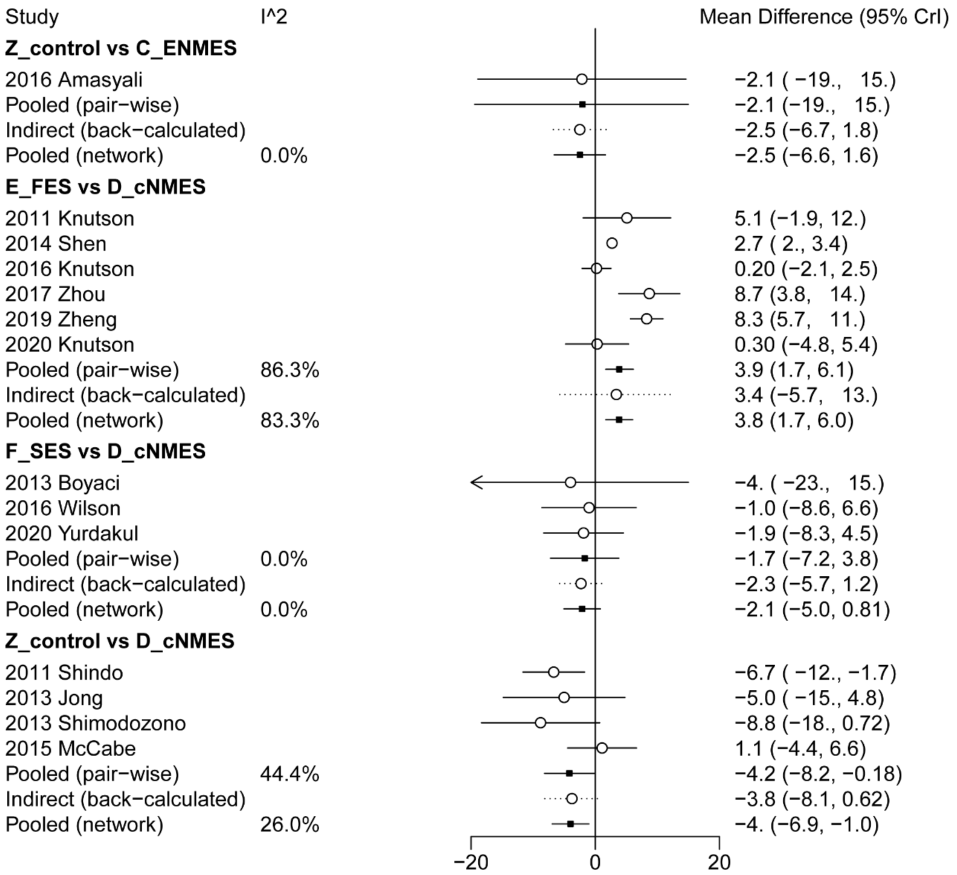
Study
I^2
Mean Difference (95% CrI)
Z_control vs C_ENMES
2016 Amasyali
−2.1 (−19., 15.)
Pooled (pair−wise)
−2.1 (−19., 15.)
Indirect (back−calculated)
−2.5 (−6.7, 1.8)
Pooled (network)
0.0%
−2.5 (−6.6, 1.6)
E_FES vs D_cNMES
2011 Knutson
5.1 (−1.9, 12.)
2014 Shen
2.7 (2., 3.4)
2016 Knutson
0.20 (−2.1, 2.5)
2017 Zhou
8.7 (3.8, 14.)
2019 Zheng
8.3 (5.7, 11.)
2020 Knutson
0.30 (−4.8, 5.4)
Pooled (pair−wise)
86.3%
3.9 (1.7, 6.1)
Indirect (back−calculated)
3.4 (−5.7, 13.)
Pooled (network)
83.3%
3.8 (1.7, 6.0)
F_SES vs D_cNMES
2013 Boyaci
−4. (−23., 15.)
2016 Wilson
−1.0 (−8.6, 6.6)
2020 Yurdakul
−1.9 (−8.3, 4.5)
Pooled (pair−wise)
0.0%
−1.7 (−7.2, 3.8)
Indirect (back−calculated)
−2.3 (−5.7, 1.2)
Pooled (network)
0.0%
−2.1 (−5.0, 0.81)
Z_control vs D_cNMES
2011 Shindo
−6.7 (−12., −1.7)
2013 Jong
−5.0 (−15., 4.8)
2013 Shimodozono
−8.8 (−18., 0.72)
2015 McCabe
1.1 (−4.4, 6.6)
Pooled (pair−wise)
44.4%
−4.2 (−8.2, −0.18)
Indirect (back−calculated)
−3.8 (−8.1, 0.62)
Pooled (network)
26.0%
−4. (−6.9, −1.0)
−20
0
20

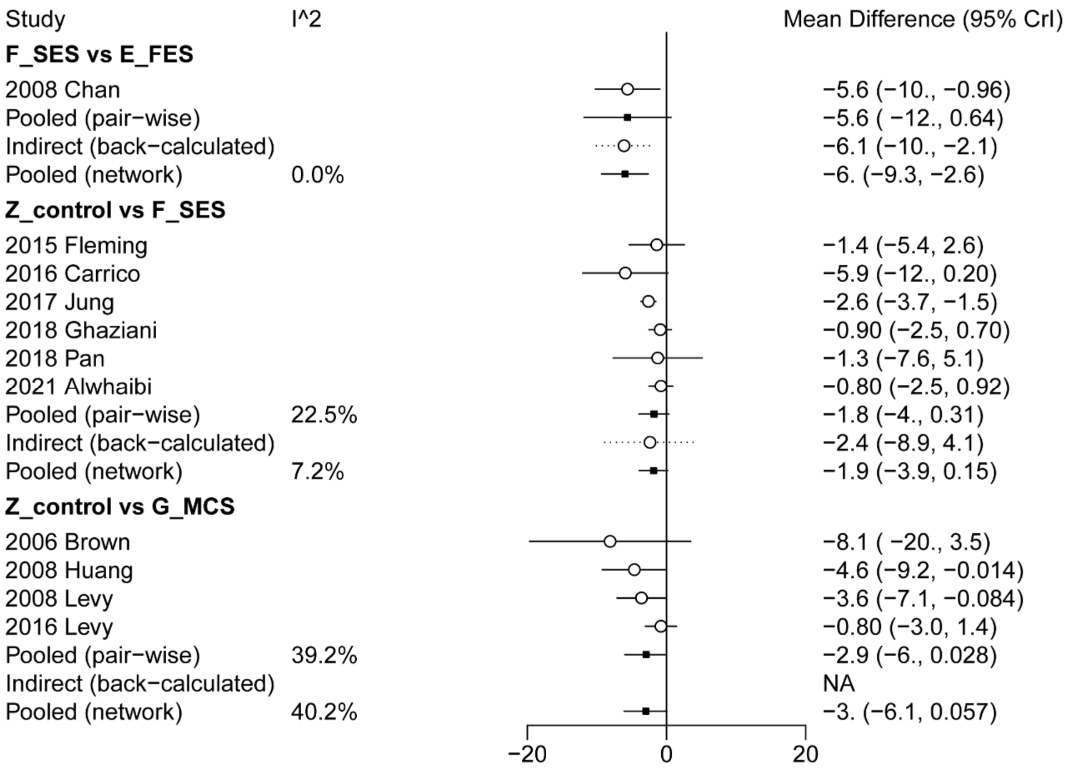
Study
I^2
Mean Difference (95% CrI)
F_SES vs E_FES
2008 Chan −5.6 (−10., −0.96)
Pooled (pair−wise) −5.6 (−12., 0.64)
Indirect (back−calculated) −6.1 (−10., −2.1)
Pooled (network) 0.0% −6. (−9.3, −2.6)
Z_control vs F_SES
2015 Fleming −1.4 (−5.4, 2.6)
2016 Carrico −5.9 (−12., 0.20)
2017 Jung −2.6 (−3.7, −1.5)
2018 Ghaziani −0.90 (−2.5, 0.70)
2018 Pan −1.3 (−7.6, 5.1)
2021 Alwhaibi −0.80 (−2.5, 0.92)
Pooled (pair−wise) 22.5% −1.8 (−4., 0.31)
Indirect (back−calculated) −2.4 (−8.9, 4.1)
Pooled (network) 7.2% −1.9 (−3.9, 0.15)
Z_control vs G_MCS
2006 Brown −8.1 (−20., 3.5)
2008 Huang −4.6 (−9.2, −0.014)
2008 Levy −3.6 (−7.1, −0.084)
2016 Levy −0.80 (−3.0, 1.4)
Pooled (pair−wise) 39.2% −2.9 (−6., 0.028)
Indirect (back−calculated) NA
Pooled (network) 40.2% −3. (−6.1, 0.057)
−20
0
20

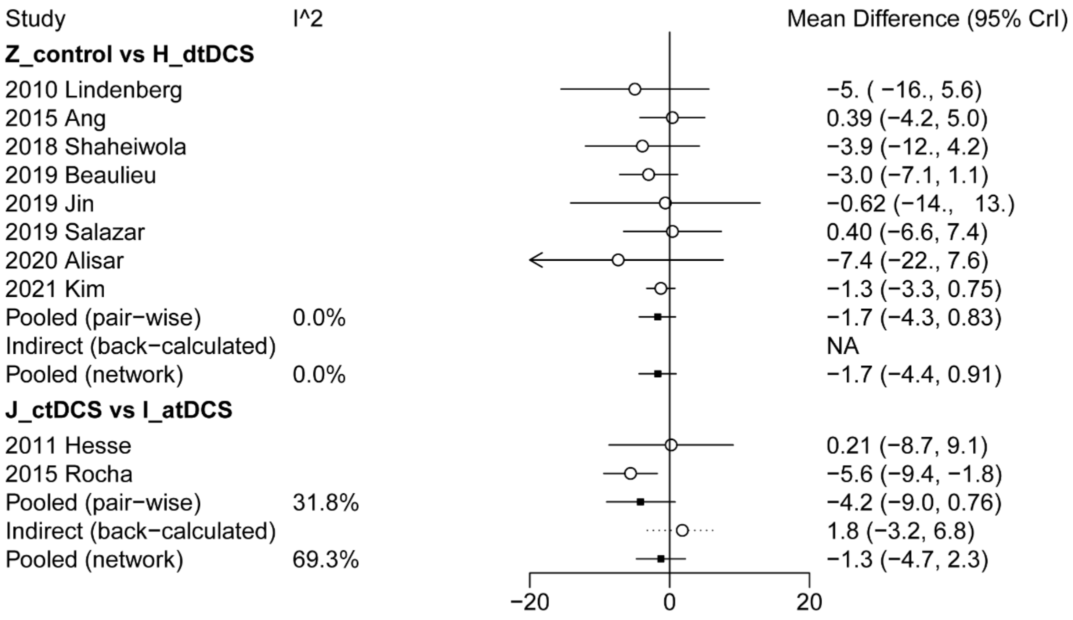
Study
I^2
Mean Difference (95% CrI)
Z_control vs H_dtDCS
2010 Lindenberg −5. (−16., 5.6)
2015 Ang 0.39 (−4.2, 5.0)
2018 Shaheiwola −3.9 (−12., 4.2)
2019 Beaulieu −3.0 (−7.1, 1.1)
2019 Jin −0.62 (−14., 13.)
2019 Salazar 0.40 (−6.6, 7.4)
2020 Alisar −7.4 (−22., 7.6)
2021 Kim −1.3 (−3.3, 0.75)
Pooled (pair−wise) 0.0% −1.7 (−4.3, 0.83)
Indirect (back−calculated) NA
Pooled (network) 0.0% −1.7 (−4.4, 0.91)
J_ctDCS vs I_atDCS
2011 Hesse 0.21 (−8.7, 9.1)
2015 Rocha −5.6 (−9.4, −1.8)
Pooled (pair−wise) 31.8% −4.2 (−9.0, 0.76)
Indirect (back−calculated) 1.8 (−3.2, 6.8)
Pooled (network) 69.3% −1.3 (−4.7, 2.3)
−20
0
20

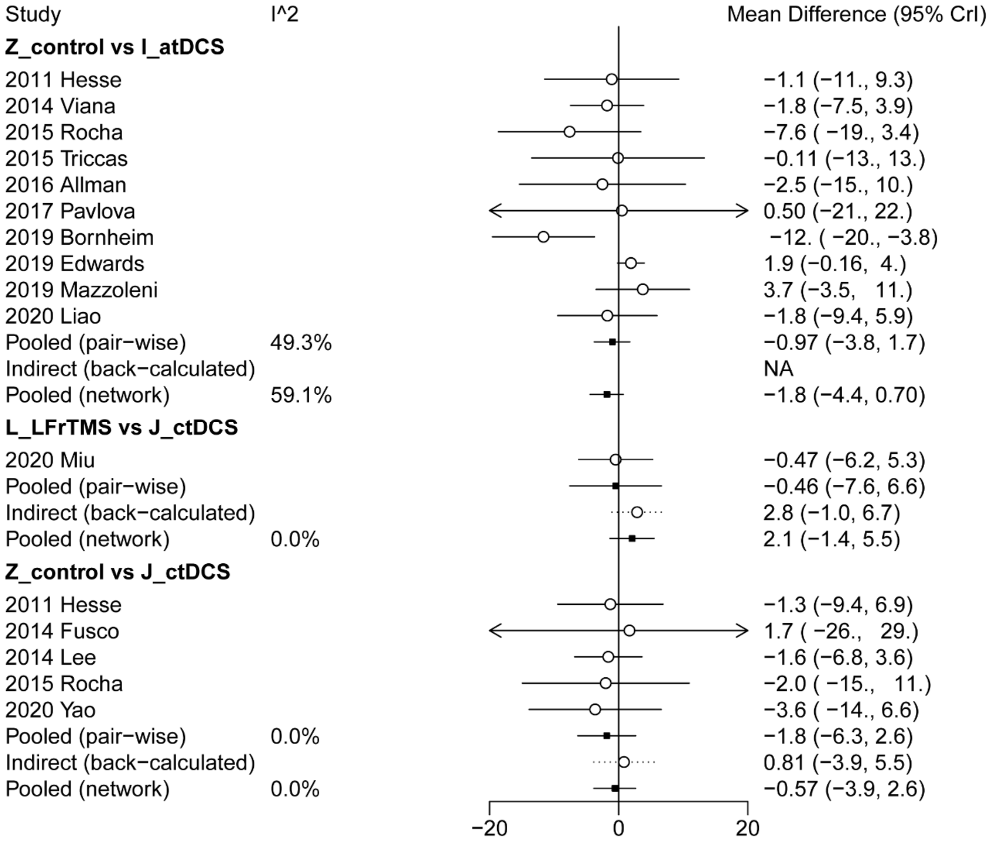
Study
I^2
Mean Difference (95% CrI)
Z_control vs I_atDCS
2011 Hesse −1.1 (−11., 9.3)
2014 Viana −1.8 (−7.5, 3.9)
2015 Rocha −7.6 (−19., 3.4)
2015 Triccas −0.11 (−13., 13.)
2016 Allman −2.5 (−15., 10.)
2017 Pavlova 0.50 (−21., 22.)
2019 Bornheim −12. (−20., −3.8)
2019 Edwards 1.9 (−0.16, 4.)
2019 Mazzoleni 3.7 (−3.5, 11.)
2020 Liao −1.8 (−9.4, 5.9)
Pooled (pair−wise) 49.3% −0.97 (−3.8, 1.7)
Indirect (back−calculated) NA
Pooled (network) 59.1% −1.8 (−4.4, 0.70)
L_LFrTMS vs J_ctDCS
2020 Miu −0.47 (−6.2, 5.3)
Pooled (pair−wise) −0.46 (−7.6, 6.6)
Indirect (back−calculated) 2.8 (−1.0, 6.7)
Pooled (network) 0.0% 2.1 (−1.4, 5.5)
Z_control vs J_ctDCS
2011 Hesse −1.3 (−9.4, 6.9)
2014 Fusco 1.7 (−26., 29.)
2014 Lee −1.6 (−6.8, 3.6)
2015 Rocha −2.0 (−15., 11.)
2020 Yao −3.6 (−14., 6.6)
Pooled (pair−wise) 0.0% −1.8 (−6.3, 2.6)
Indirect (back−calculated) 0.81 (−3.9, 5.5)
Pooled (network) 0.0% −0.57 (−3.9, 2.6)
−20
0
20

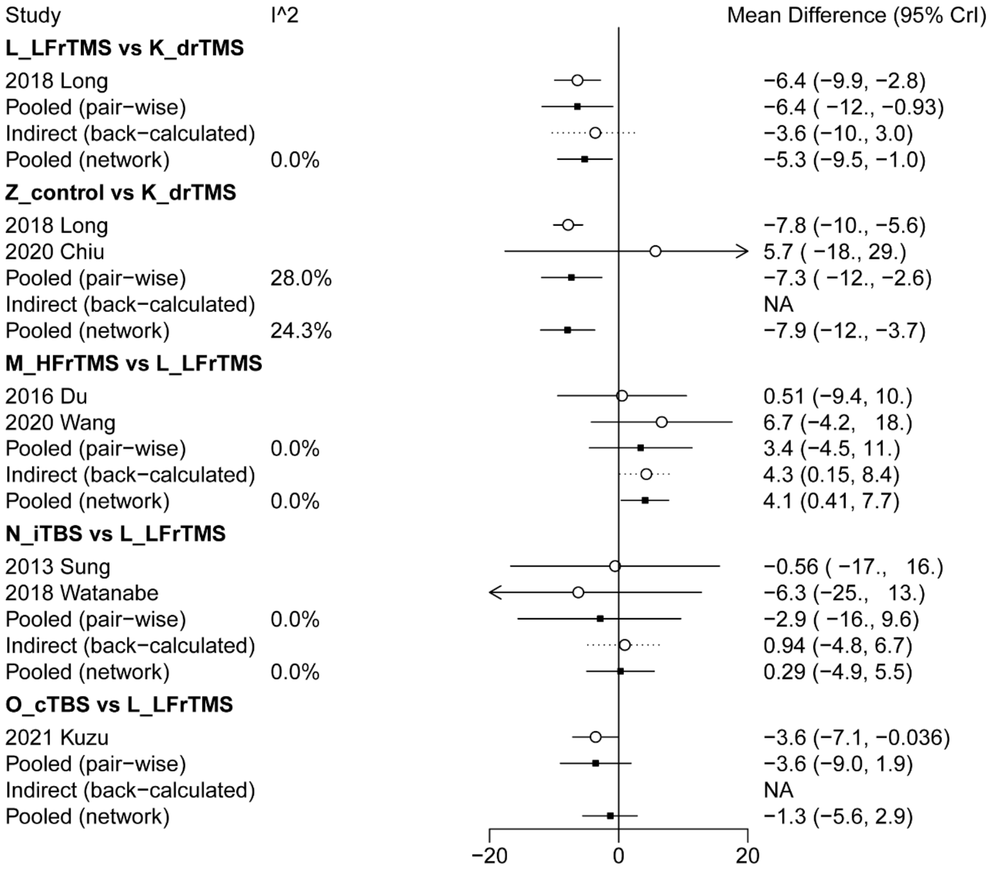
Study
I^2
Mean Difference (95% CrI)
L_LFrTMS vs K_drTMS
2018 Long −6.4 (−9.9, −2.8)
Pooled (pair−wise) −6.4 (−12., −0.93)
Indirect (back−calculated) −3.6 (−10., 3.0)
Pooled (network) 0.0% −5.3 (−9.5, −1.0)
Z_control vs K_drTMS
2018 Long −7.8 (−10., −5.6)
2020 Chiu 5.7 (−18., 29.)
Pooled (pair−wise) 28.0% −7.3 (−12., −2.6)
Indirect (back−calculated) NA
Pooled (network) 24.3% −7.9 (−12., −3.7)
M_HFrTMS vs L_LFrTMS
2016 Du 0.51 (−9.4, 10.)
2020 Wang 6.7 (−4.2, 18.)
Pooled (pair−wise) 0.0% 3.4 (−4.5, 11.)
Indirect (back−calculated) 4.3 (0.15, 8.4)
Pooled (network) 0.0% 4.1 (0.41, 7.7)
N_iTBS vs L_LFrTMS
2013 Sung −0.56 (−17., 16.)
2018 Watanabe −6.3 (−25., 13.)
Pooled (pair−wise) 0.0% −2.9 (−16., 9.6)
Indirect (back−calculated) 0.94 (−4.8, 6.7)
Pooled (network) 0.0% 0.29 (−4.9, 5.5)
O_cTBS vs L_LFrTMS
2021 Kuzu −3.6 (−7.1, −0.036)
Pooled (pair−wise) −3.6 (−9.0, 1.9)
Indirect (back−calculated) NA
Pooled (network) −1.3 (−5.6, 2.9)
−20
0
20

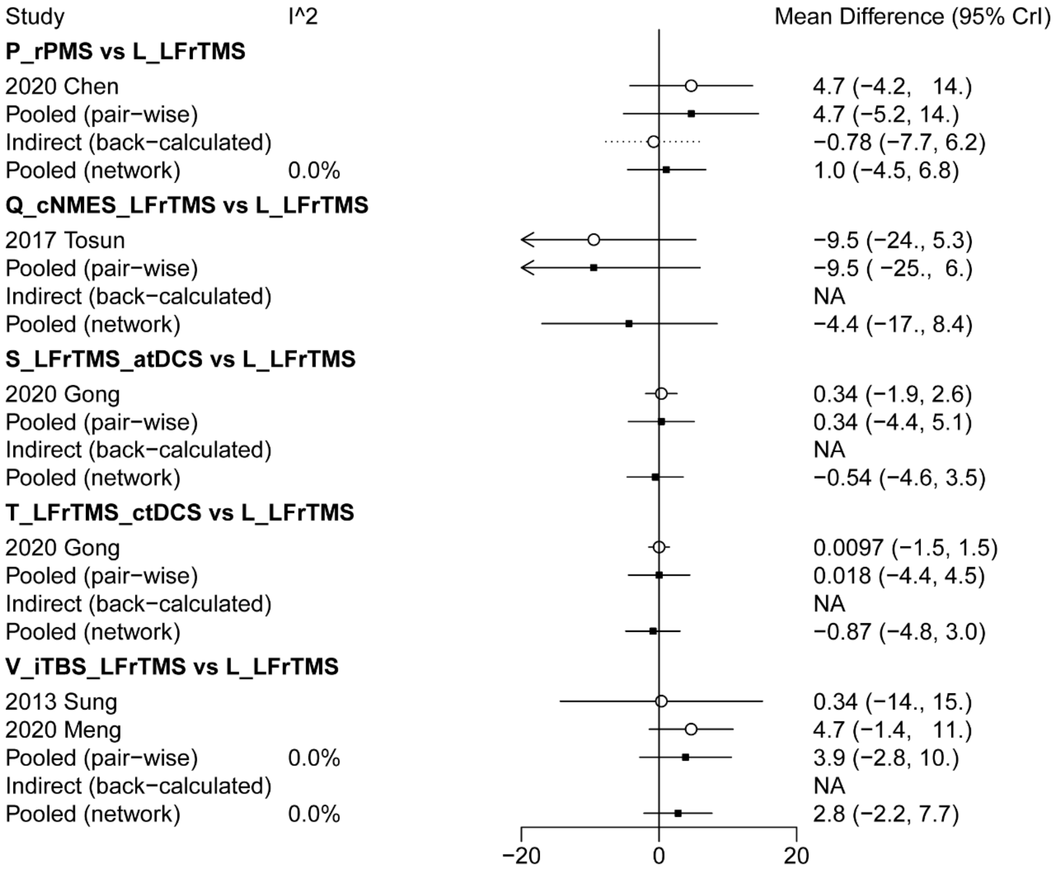
Study
I^2
Mean Difference (95% CrI)
P_rPMS vs L_LFrTMS
2020 Chen 4.7 (−4.2, 14.)
Pooled (pair−wise) 4.7 (−5.2, 14.)
Indirect (back−calculated) −0.78 (−7.7, 6.2)
Pooled (network) 0.0% 1.0 (−4.5, 6.8)
Q_cNMES_LFrTMS vs L_LFrTMS
2017 Tosun −9.5 (−24., 5.3)
Pooled (pair−wise) −9.5 (−25., 6.)
Indirect (back−calculated) NA
Pooled (network) −4.4 (−17., 8.4)
S_LFrTMS_atDCS vs L_LFrTMS
2020 Gong 0.34 (−1.9, 2.6)
Pooled (pair−wise) 0.34 (−4.4, 5.1)
Indirect (back−calculated) NA
Pooled (network) −0.54 (−4.6, 3.5)
T_LFrTMS_ctDCS vs L_LFrTMS
2020 Gong 0.0097 (−1.5, 1.5)
Pooled (pair−wise) 0.018 (−4.4, 4.5)
Indirect (back−calculated) NA
Pooled (network) −0.87 (−4.8, 3.0)
V_iTBS_LFrTMS vs L_LFrTMS
2013 Sung 0.34 (−14., 15.)
2020 Meng 4.7 (−1.4, 11.)
Pooled (pair−wise) 0.0% 3.9 (−2.8, 10.)
Indirect (back−calculated) NA
Pooled (network) 0.0% 2.8 (−2.2, 7.7)
−20
0
20

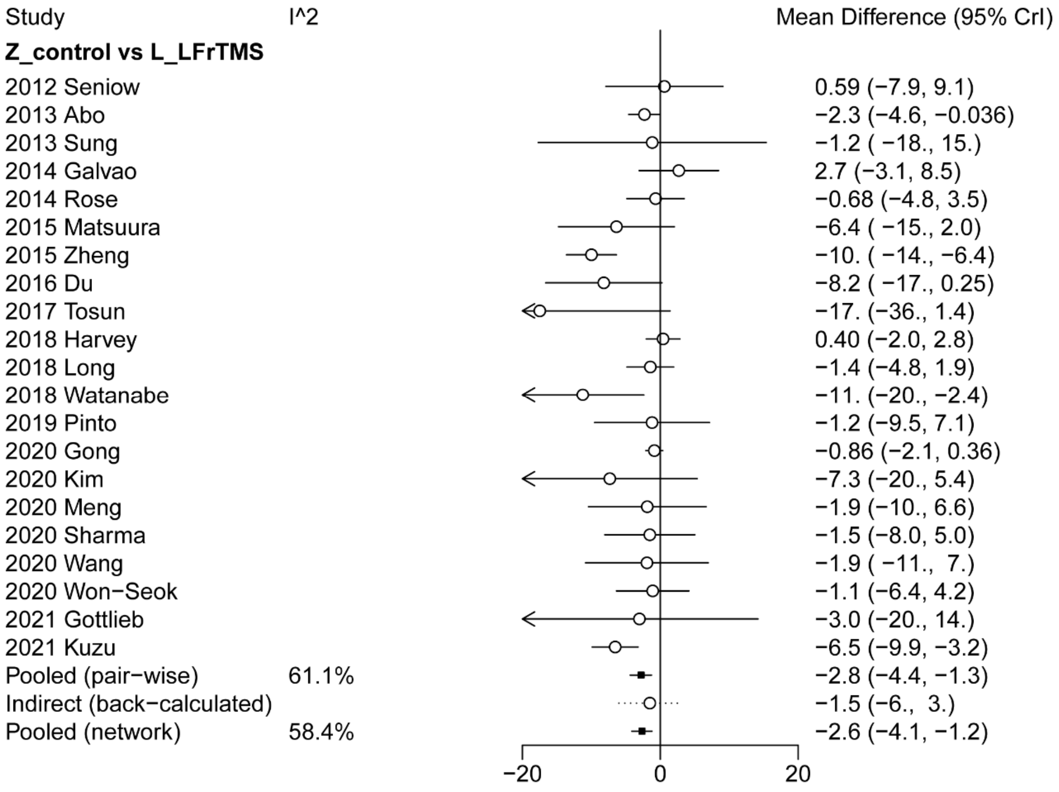
Study
I^2
Mean Difference (95% CrI)
Z_control vs L_LFrTMS
2012 Seniow 0.59 (−7.9, 9.1)
2013 Abo −2.3 (−4.6, −0.036)
2013 Sung −1.2 (−18., 15.)
2014 Galvao 2.7 (−3.1, 8.5)
2014 Rose −0.68 (−4.8, 3.5)
2015 Matsuura −6.4 (−15., 2.0)
2015 Zheng −10. (−14., −6.4)
2016 Du −8.2 (−17., 0.25)
2017 Tosun −17. (−36., 1.4)
2018 Harvey 0.40 (−2.0, 2.8)
2018 Long −1.4 (−4.8, 1.9)
2018 Watanabe −11. (−20., −2.4)
2019 Pinto −1.2 (−9.5, 7.1)
2020 Gong −0.86 (−2.1, 0.36)
2020 Kim −7.3 (−20., 5.4)
2020 Meng −1.9 (−10., 6.6)
2020 Sharma −1.5 (−8.0, 5.0)
2020 Wang −1.9 (−11., 7.)
2020 Won−Seok −1.1 (−6.4, 4.2)
2021 Gottlieb −3.0 (−20., 14.)
2021 Kuzu −6.5 (−9.9, −3.2)
Pooled (pair−wise) 61.1% −2.8 (−4.4, −1.3)
Indirect (back−calculated) −1.5 (−6., 3.)
Pooled (network) 58.4% −2.6 (−4.1, −1.2)
−20
0
20

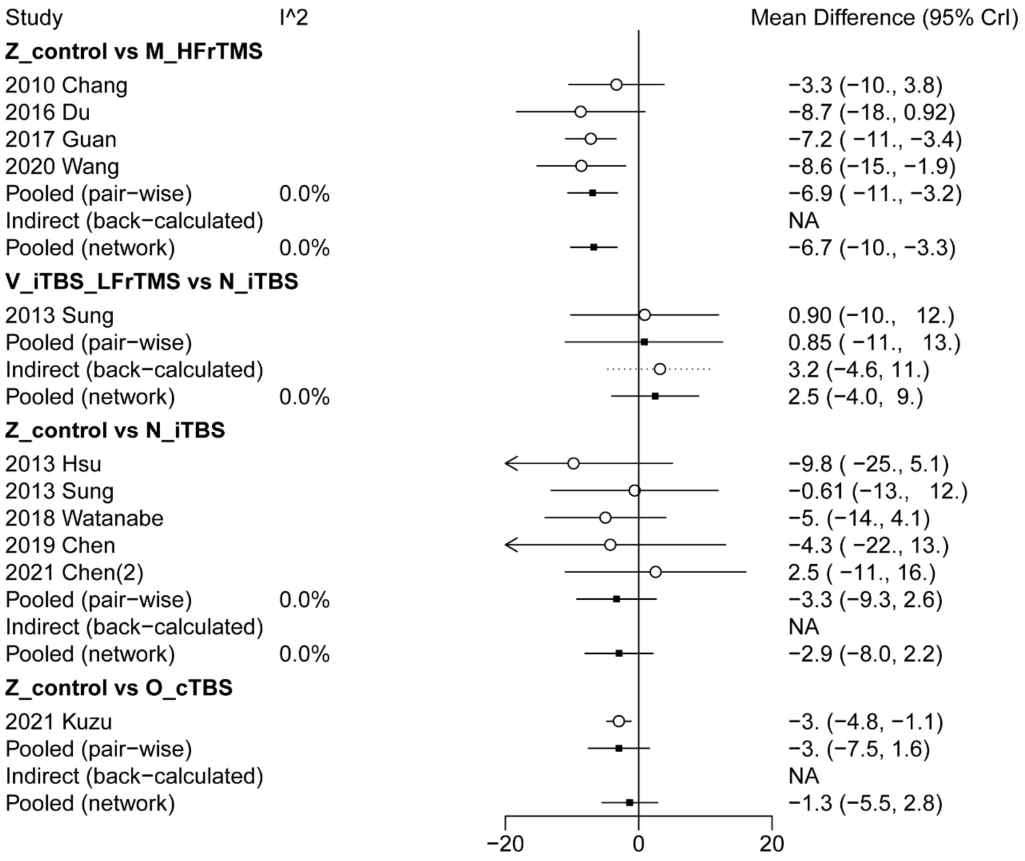
Study
I^2
Mean Difference (95% CrI)
Z_control vs M_HFrTMS
2010 Chang −3.3 (−10., 3.8)
2016 Du −8.7 (−18., 0.92)
2017 Guan −7.2 (−11., −3.4)
2020 Wang −8.6 (−15., −1.9)
Pooled (pair−wise) 0.0% −6.9 (−11., −3.2)
Indirect (back−calculated) NA
Pooled (network) 0.0% −6.7 (−10., −3.3)
V_iTBS_LFrTMS vs N_iTBS
2013 Sung 0.90 (−10., 12.)
Pooled (pair−wise) 0.85 (−11., 13.)
Indirect (back−calculated) 3.2 (−4.6, 11.)
Pooled (network) 0.0% 2.5 (−4.0, 9.)
Z_control vs N_iTBS
2013 Hsu −9.8 (−25., 5.1)
2013 Sung −0.61 (−13., 12.)
2018 Watanabe −5. (−14., 4.1)
2019 Chen −4.3 (−22., 13.)
2021 Chen(2) 2.5 (−11., 16.)
Pooled (pair−wise) 0.0% −3.3 (−9.3, 2.6)
Indirect (back−calculated) NA
Pooled (network) 0.0% −2.9 (−8.0, 2.2)
Z_control vs O_cTBS
2021 Kuzu −3. (−4.8, −1.1)
Pooled (pair−wise) −3. (−7.5, 1.6)
Indirect (back−calculated) NA
Pooled (network) −1.3 (−5.5, 2.8)
−20
0
20

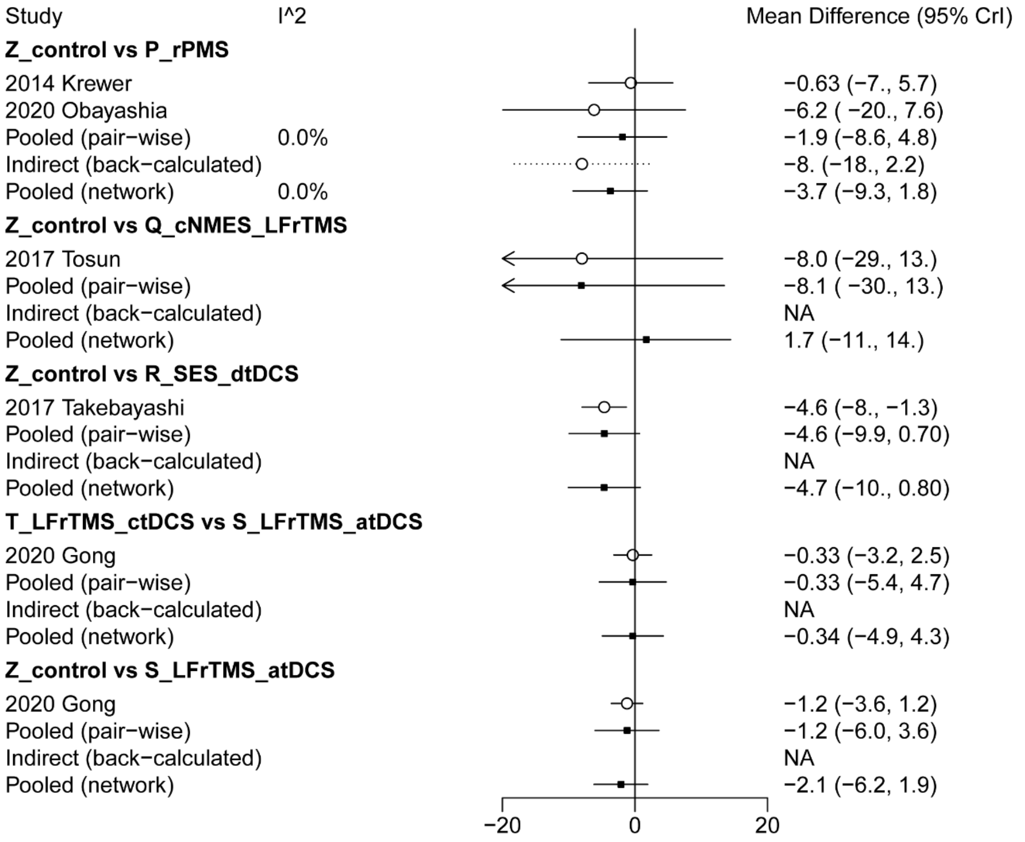
Study
I^2
Mean Difference (95% CrI)
Z_control vs P_rPMS
2014 Krewer −0.63 (−7., 5.7)
2020 Obayashia −6.2 (−20., 7.6)
Pooled (pair−wise) 0.0% −1.9 (−8.6, 4.8)
Indirect (back−calculated) −8. (−18., 2.2)
Pooled (network) 0.0% −3.7 (−9.3, 1.8)
Z_control vs Q_cNMES_LFrTMS
2017 Tosun −8.0 (−29., 13.)
Pooled (pair−wise) −8.1 (−30., 13.)
Indirect (back−calculated) NA
Pooled (network) 1.7 (−11., 14.)
Z_control vs R_SES_dtDCS
2017 Takebayashi −4.6 (−8., −1.3)
Pooled (pair−wise) −4.6 (−9.9, 0.70)
Indirect (back−calculated) NA
Pooled (network) −4.7 (−10., 0.80)
T_LFrTMS_ctDCS vs S_LFrTMS_atDCS
2020 Gong −0.33 (−3.2, 2.5)
Pooled (pair−wise) −0.33 (−5.4, 4.7)
Indirect (back−calculated) NA
Pooled (network) −0.34 (−4.9, 4.3)
Z_control vs S_LFrTMS_atDCS
2020 Gong −1.2 (−3.6, 1.2)
Pooled (pair−wise) −1.2 (−6.0, 3.6)
Indirect (back−calculated) NA
Pooled (network) −2.1 (−6.2, 1.9)
−20
0
20

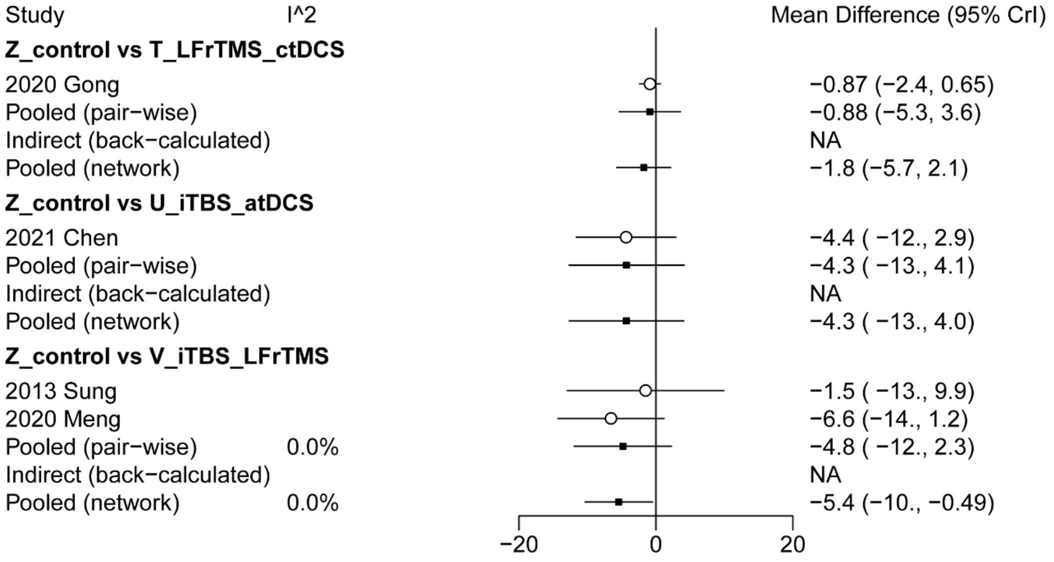
Study
I^2
Mean Difference (95% CrI)
Z_control vs T_LFrTMS_ctDCS
2020 Gong
−0.87 (−2.4, 0.65)
Pooled (pair−wise)
−0.88 (−5.3, 3.6)
Indirect (back−calculated)
NA
Pooled (network)
−1.8 (−5.7, 2.1)
Z_control vs U_iTBS_atDCS
2021 Chen
−4.4 (−12., 2.9)
Pooled (pair−wise)
−4.3 (−13., 4.1)
Indirect (back−calculated)
NA
Pooled (network)
−4.3 (−13., 4.0)
Z_control vs V_iTBS_LFrTMS
2013 Sung
−1.5 (−13., 9.9)
2020 Meng
−6.6 (−14., 1.2)
Pooled (pair−wise)
0.0%
−4.8 (−12., 2.3)
Indirect (back−calculated)
NA
Pooled (network)
0.0%
−5.4 (−10., −0.49)
−20
0
20

M. Network local consistent analysis and heterogeneity analysis of FMA-UE EOT

Consistency

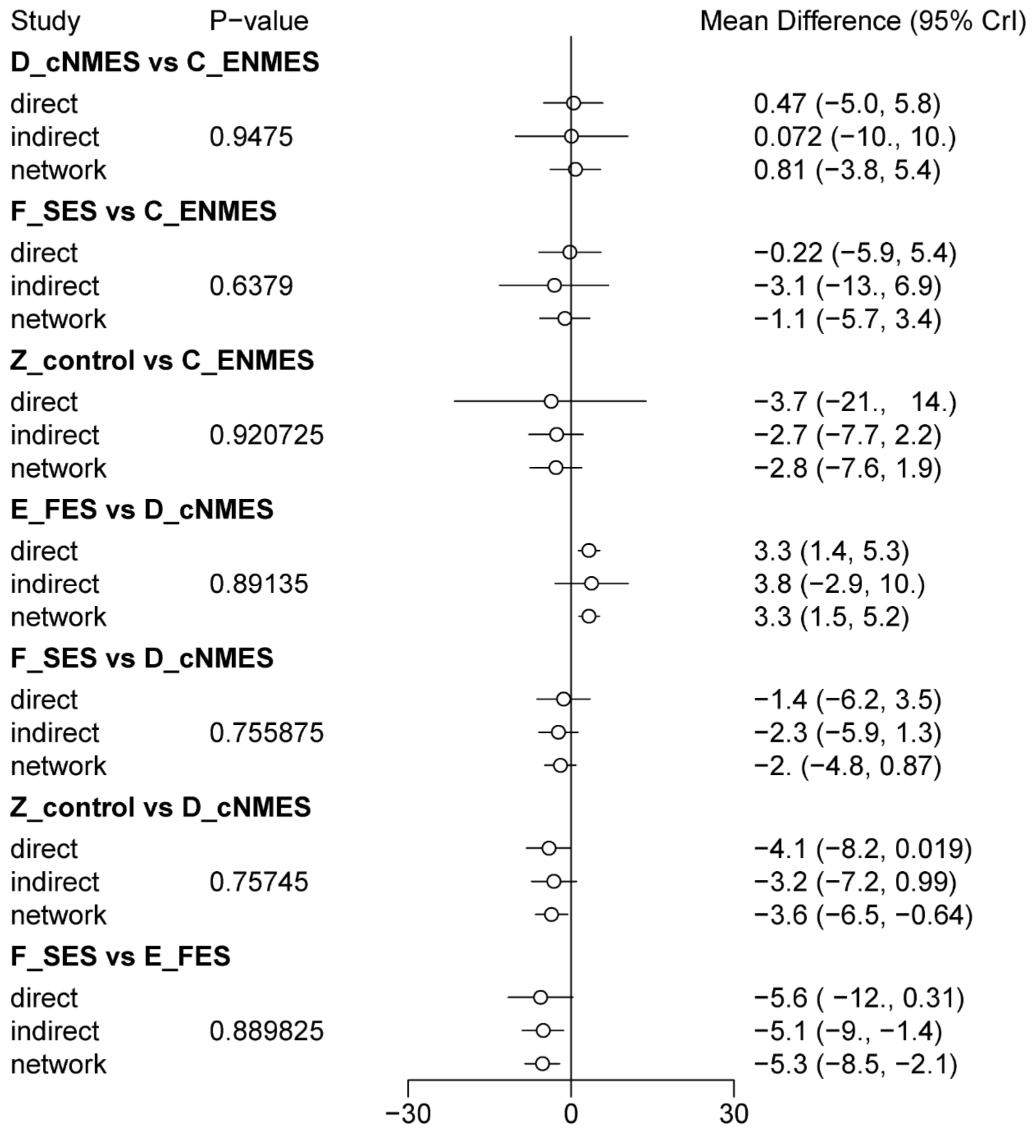

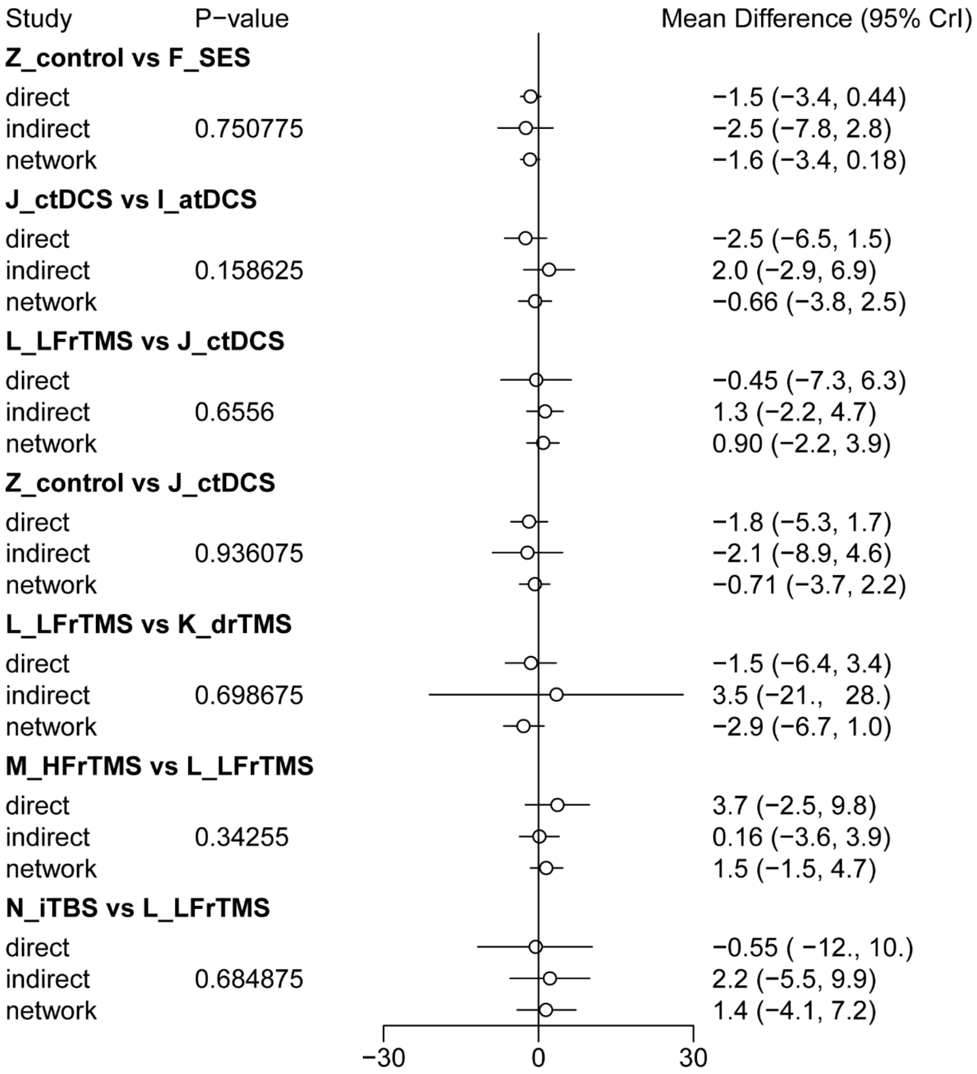
Study
P−value
Mean Difference (95% CrI)
Z_control vs F_SES
direct −1.5 (−3.4, 0.44)
indirect 0.750775 −2.5 (−7.8, 2.8)
network −1.6 (−3.4, 0.18)
J_ctDCS vs I_atDCS
direct −2.5 (−6.5, 1.5)
indirect 0.158625 2.0 (−2.9, 6.9)
network −0.66 (−3.8, 2.5)
L_LFrTMS vs J_ctDCS
direct −0.45 (−7.3, 6.3)
indirect 0.6556 1.3 (−2.2, 4.7)
network 0.90 (−2.2, 3.9)
Z_control vs J_ctDCS
direct −1.8 (−5.3, 1.7)
indirect 0.936075 −2.1 (−8.9, 4.6)
network −0.71 (−3.7, 2.2)
L_LFrTMS vs K_drTMS
direct −1.5 (−6.4, 3.4)
indirect 0.698675 3.5 (−21., 28.)
network −2.9 (−6.7, 1.0)
M_HFrTMS vs L_LFrTMS
direct 3.7 (−2.5, 9.8)
indirect 0.34255 0.16 (−3.6, 3.9)
network 1.5 (−1.5, 4.7)
N_iTBS vs L_LFrTMS
direct −0.55 (−12., 10.)
indirect 0.684875 2.2 (−5.5, 9.9)
network 1.4 (−4.1, 7.2)
−30
0
30

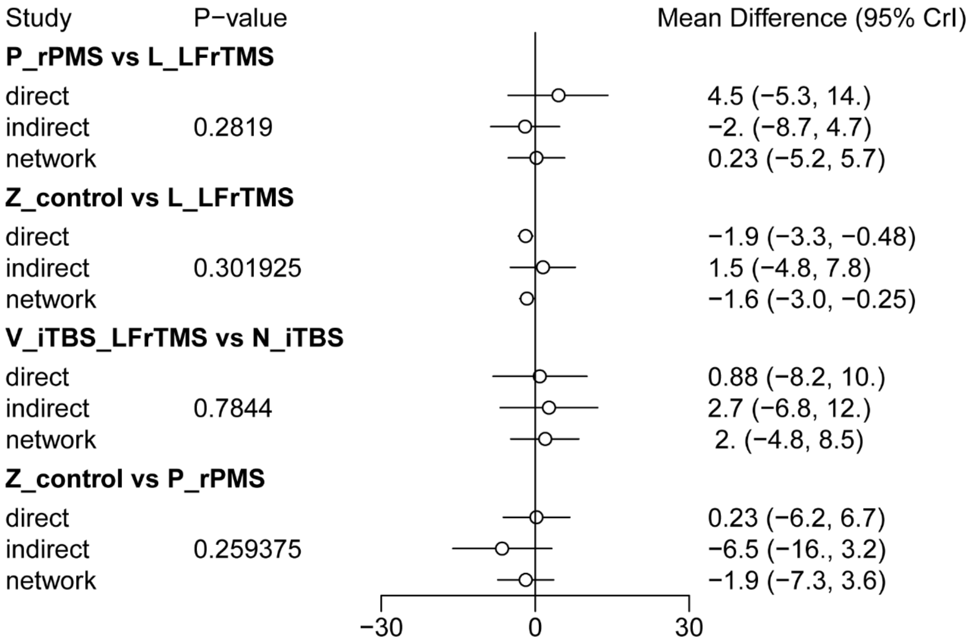

Heterogeneity

	t1	t2	i2.pair	i2.cons	incons.p
1	A_VNS	Z_control	0.000000	0.000000	NA
2	B_taVNS	Z_control	0.000000	0.000000	NA
3	C_ENMES	D_cNMES	0.000000	0.000000	0.8250775
4	C_ENMES	F_SES	0.000000	0.000000	0.7263454
5	C_ENMES	Z_control	NA	0.000000	0.8791687
6	D_cNMES	E_FES	87.635766	85.483686	0.7942303
7	D_cNMES	F_SES	0.000000	0.000000	0.7976673
8	D_cNMES	Z_control	62.565913	44.708316	0.7634873
9	E_FES	F_SES	NA	0.000000	0.8832595
10	F_SES	Z_control	50.948688	39.018147	0.7798413
11	G_MCS	Z_control	5.319601	7.181909	NA
12	H_dtDCS	Z_control	0.000000	0.000000	NA
13	I_atDCS	J_ctDCS	0.000000	38.608138	0.1976057
14	I_atDCS	Z_control	1.922872	13.035208	NA
15	J_ctDCS	L_LFrTMS	NA	0.000000	0.6366521
16	J_ctDCS	Z_control	0.000000	0.000000	0.5235552
17	K_drTMS	L_LFrTMS	NA	0.000000	0.5328031
18	K_drTMS	Z_control	0.000000	0.000000	NA
19	L_LFrTMS	M_HFrTMS	0.000000	0.000000	0.6321911
20	L_LFrTMS	N_iTBS	NA	0.000000	0.8038929
21	L_LFrTMS	O_cTBS	NA	NA	NA
22	L_LFrTMS	P_rPMS	NA	27.919366	0.2637662
23	L_LFrTMS	Q_cNMES_LFrTMS	NA	NA	NA
24	L_LFrTMS	S_LFrTMS_atDCS	NA	NA	NA

25	L_LFrTMS	T_LFrTMS_ctDCS	NA	NA	NA
26	L_LFrTMS	V_iTBS_LFrTMS	0.000000	0.000000	NA
27	L_LFrTMS	Z_control	5.306443	0.000000	0.7269299
28	M_HFrTMS	Z_control	34.515560	41.642084	NA
29	N_iTBS	V_iTBS_LFrTMS	NA	0.000000	0.8383613
30	N_iTBS	Z_control	0.000000	0.000000	NA
31	O_cTBS	Z_control	NA	NA	NA
32	P_rPMS	Z_control	11.460352	24.639553	0.2539980
33	Q_cNMES_LFrTMS	Z_control	NA	NA	NA
34	R_SES_dtDCS	Z_control	NA	NA	NA
35	S_LFrTMS_atDCS	T_LFrTMS_ctDCS	NA	NA	NA
36	S_LFrTMS_atDCS	Z_control	NA	NA	NA
37	T_LFrTMS_ctDCS	Z_control	NA	NA	NA
38	U_iTBS_atDCS	Z_control	NA	NA	NA
39	V_iTBS_LFrTMS	Z_control	0.000000	0.000000	NA
Global I-squared: ------------------------- i2.pair = 31.4507; i2.cons = 24.5804					

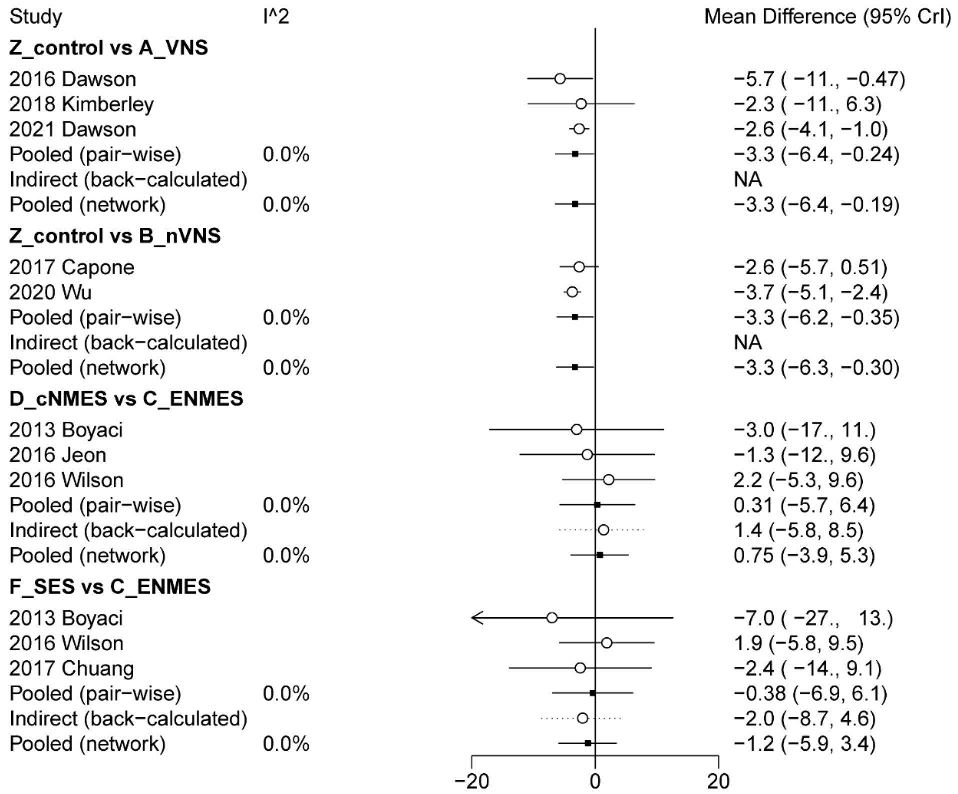

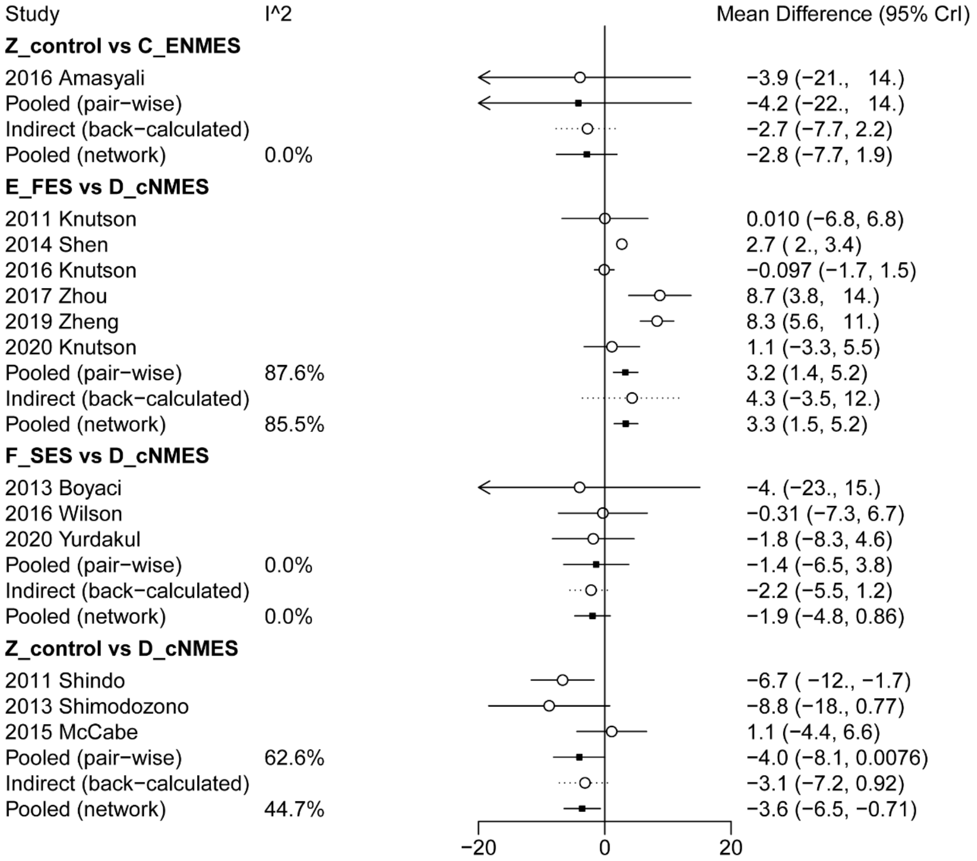
Study
I^2
Mean Difference (95% CrI)
Z_control vs C_ENMES
2016 Amasyali
−3.9 (−21., 14.)
Pooled (pair−wise)
−4.2 (−22., 14.)
Indirect (back−calculated)
−2.7 (−7.7, 2.2)
Pooled (network)
0.0%
−2.8 (−7.7, 1.9)
E_FES vs D_cNMES
2011 Knutson
0.010 (−6.8, 6.8)
2014 Shen
2.7 (2., 3.4)
2016 Knutson
−0.097 (−1.7, 1.5)
2017 Zhou
8.7 (3.8, 14.)
2019 Zheng
8.3 (5.6, 11.)
2020 Knutson
1.1 (−3.3, 5.5)
Pooled (pair−wise)
87.6%
3.2 (1.4, 5.2)
Indirect (back−calculated)
4.3 (−3.5, 12.)
Pooled (network)
85.5%
3.3 (1.5, 5.2)
F_SES vs D_cNMES
2013 Boyaci
−4. (−23., 15.)
2016 Wilson
−0.31 (−7.3, 6.7)
2020 Yurdakul
−1.8 (−8.3, 4.6)
Pooled (pair−wise)
0.0%
−1.4 (−6.5, 3.8)
Indirect (back−calculated)
−2.2 (−5.5, 1.2)
Pooled (network)
0.0%
−1.9 (−4.8, 0.86)
Z_control vs D_cNMES
2011 Shindo
−6.7 (−12., −1.7)
2013 Shimodozono
−8.8 (−18., 0.77)
2015 McCabe
1.1 (−4.4, 6.6)
Pooled (pair−wise)
62.6%
−4.0 (−8.1, 0.0076)
Indirect (back−calculated)
−3.1 (−7.2, 0.92)
Pooled (network)
44.7%
−3.6 (−6.5, −0.71)
−20
0
20

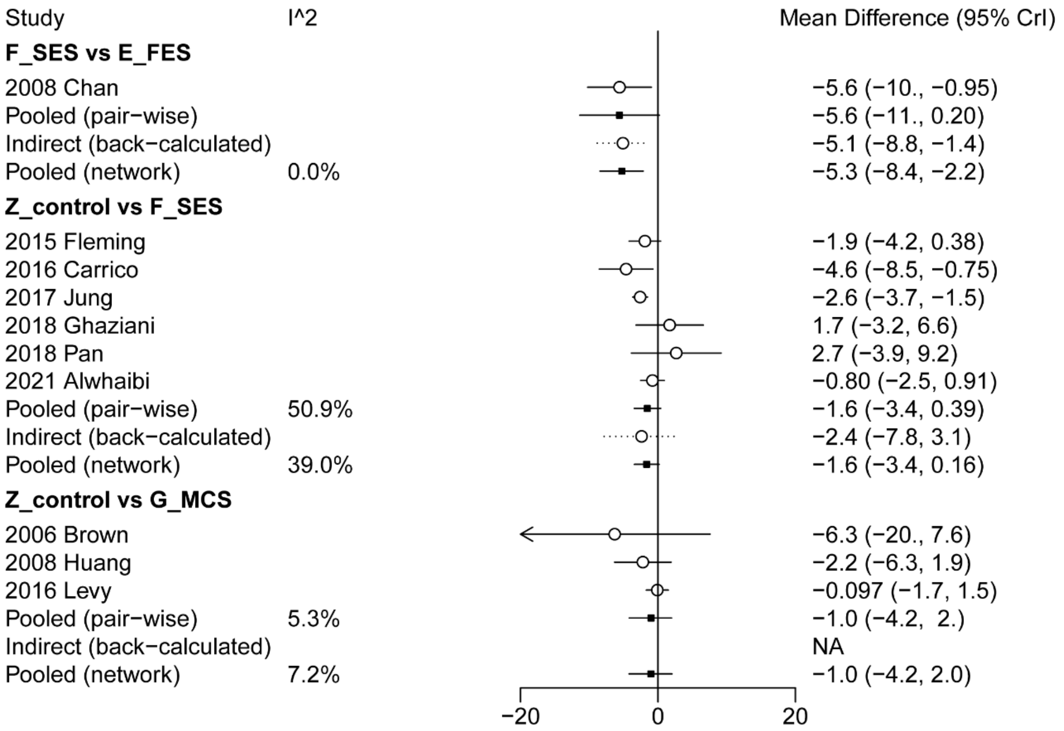
Study
I^2
Mean Difference (95% CrI)
F_SES vs E_FES
2008 Chan −5.6 (−10., −0.95)
Pooled (pair−wise) −5.6 (−11., 0.20)
Indirect (back−calculated) −5.1 (−8.8, −1.4)
Pooled (network) 0.0% −5.3 (−8.4, −2.2)
Z_control vs F_SES
2015 Fleming −1.9 (−4.2, 0.38)
2016 Carrico −4.6 (−8.5, −0.75)
2017 Jung −2.6 (−3.7, −1.5)
2018 Ghaziani 1.7 (−3.2, 6.6)
2018 Pan 2.7 (−3.9, 9.2)
2021 Alwhaibi −0.80 (−2.5, 0.91)
Pooled (pair−wise) 50.9% −1.6 (−3.4, 0.39)
Indirect (back−calculated) −2.4 (−7.8, 3.1)
Pooled (network) 39.0% −1.6 (−3.4, 0.16)
Z_control vs G_MCS
2006 Brown −6.3 (−20., 7.6)
2008 Huang −2.2 (−6.3, 1.9)
2016 Levy −0.097 (−1.7, 1.5)
Pooled (pair−wise) 5.3% −1.0 (−4.2, 2.)
Indirect (back−calculated) NA
Pooled (network) 7.2% −1.0 (−4.2, 2.0)
−20
0
20

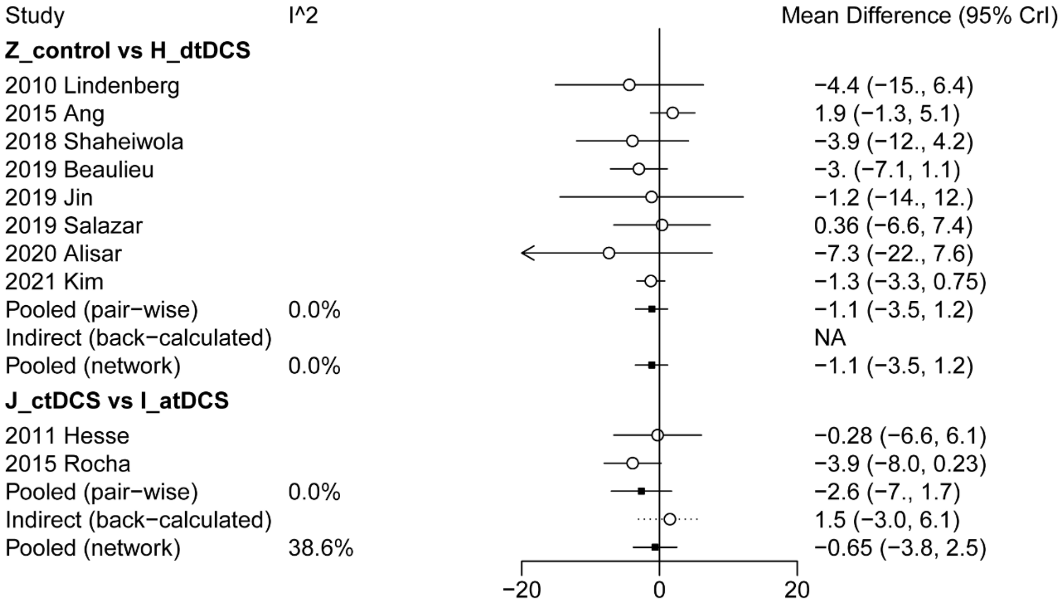
Study
I^2
Mean Difference (95% CrI)
Z_control vs H_dtDCS
2010 Lindenberg −4.4 (−15., 6.4)
2015 Ang 1.9 (−1.3, 5.1)
2018 Shaheiwola −3.9 (−12., 4.2)
2019 Beaulieu −3. (−7.1, 1.1)
2019 Jin −1.2 (−14., 12.)
2019 Salazar 0.36 (−6.6, 7.4)
2020 Alisar −7.3 (−22., 7.6)
2021 Kim −1.3 (−3.3, 0.75)
Pooled (pair−wise) 0.0% −1.1 (−3.5, 1.2)
Indirect (back−calculated) NA
Pooled (network) 0.0% −1.1 (−3.5, 1.2)
J_ctDCS vs I_atDCS
2011 Hesse −0.28 (−6.6, 6.1)
2015 Rocha −3.9 (−8.0, 0.23)
Pooled (pair−wise) 0.0% −2.6 (−7., 1.7)
Indirect (back−calculated) 1.5 (−3.0, 6.1)
Pooled (network) 38.6% −0.65 (−3.8, 2.5)
−20
0
20

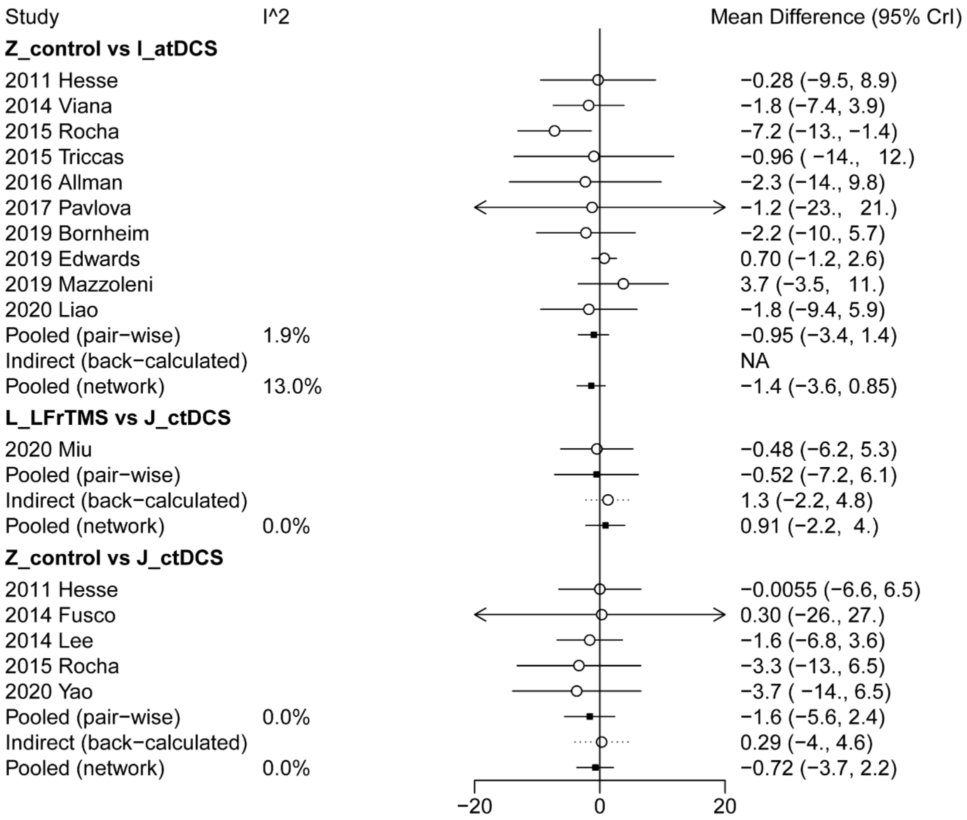
Study
I^2
Mean Difference (95% CrI)
Z_control vs I_atDCS
2011 Hesse −0.28 (−9.5, 8.9)
2014 Viana −1.8 (−7.4, 3.9)
2015 Rocha −7.2 (−13., −1.4)
2015 Triccas −0.96 (−14., 12.)
2016 Allman −2.3 (−14., 9.8)
2017 Pavlova −1.2 (−23., 21.)
2019 Bornheim −2.2 (−10., 5.7)
2019 Edwards 0.70 (−1.2, 2.6)
2019 Mazzoleni 3.7 (−3.5, 11.)
2020 Liao −1.8 (−9.4, 5.9)
Pooled (pair−wise) 1.9% −0.95 (−3.4, 1.4)
Indirect (back−calculated) NA
Pooled (network) 13.0% −1.4 (−3.6, 0.85)
L_LFrTMS vs J_ctDCS
2020 Miu −0.48 (−6.2, 5.3)
Pooled (pair−wise) −0.52 (−7.2, 6.1)
Indirect (back−calculated) 1.3 (−2.2, 4.8)
Pooled (network) 0.0% 0.91 (−2.2, 4.)
Z_control vs J_ctDCS
2011 Hesse −0.0055 (−6.6, 6.5)
2014 Fusco 0.30 (−26., 27.)
2014 Lee −1.6 (−6.8, 3.6)
2015 Rocha −3.3 (−13., 6.5)
2020 Yao −3.7 (−14., 6.5)
Pooled (pair−wise) 0.0% −1.6 (−5.6, 2.4)
Indirect (back−calculated) 0.29 (−4., 4.6)
Pooled (network) 0.0% −0.72 (−3.7, 2.2)
−20
0
20

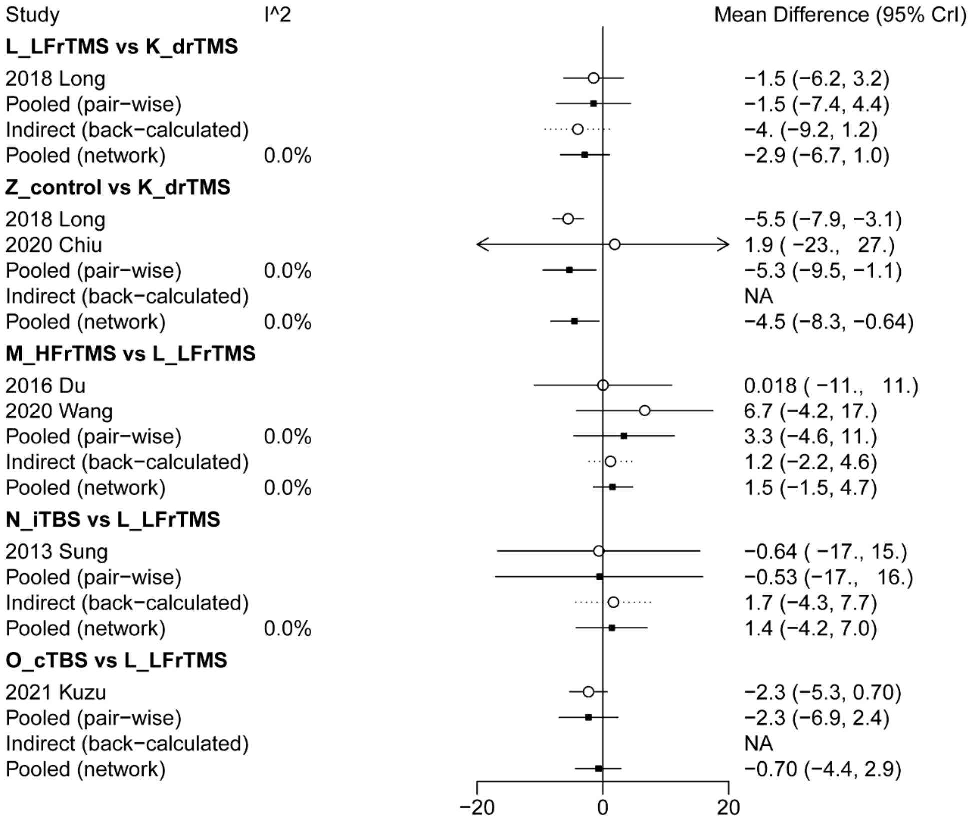
Study
I^2
Mean Difference (95% CrI)
L_LFrTMS vs K_drTMS
2018 Long −1.5 (−6.2, 3.2)
Pooled (pair−wise) −1.5 (−7.4, 4.4)
Indirect (back−calculated) −4. (−9.2, 1.2)
Pooled (network) 0.0% −2.9 (−6.7, 1.0)
Z_control vs K_drTMS
2018 Long −5.5 (−7.9, −3.1)
2020 Chiu 1.9 (−23., 27.)
Pooled (pair−wise) 0.0% −5.3 (−9.5, −1.1)
Indirect (back−calculated) NA
Pooled (network) 0.0% −4.5 (−8.3, −0.64)
M_HFrTMS vs L_LFrTMS
2016 Du 0.018 (−11., 11.)
2020 Wang 6.7 (−4.2, 17.)
Pooled (pair−wise) 0.0% 3.3 (−4.6, 11.)
Indirect (back−calculated) 1.2 (−2.2, 4.6)
Pooled (network) 0.0% 1.5 (−1.5, 4.7)
N_iTBS vs L_LFrTMS
2013 Sung −0.64 (−17., 15.)
Pooled (pair−wise) −0.53 (−17., 16.)
Indirect (back−calculated) 1.7 (−4.3, 7.7)
Pooled (network) 0.0% 1.4 (−4.2, 7.0)
O_cTBS vs L_LFrTMS
2021 Kuzu −2.3 (−5.3, 0.70)
Pooled (pair−wise) −2.3 (−6.9, 2.4)
Indirect (back−calculated) NA
Pooled (network) −0.70 (−4.4, 2.9)
−20
0
20

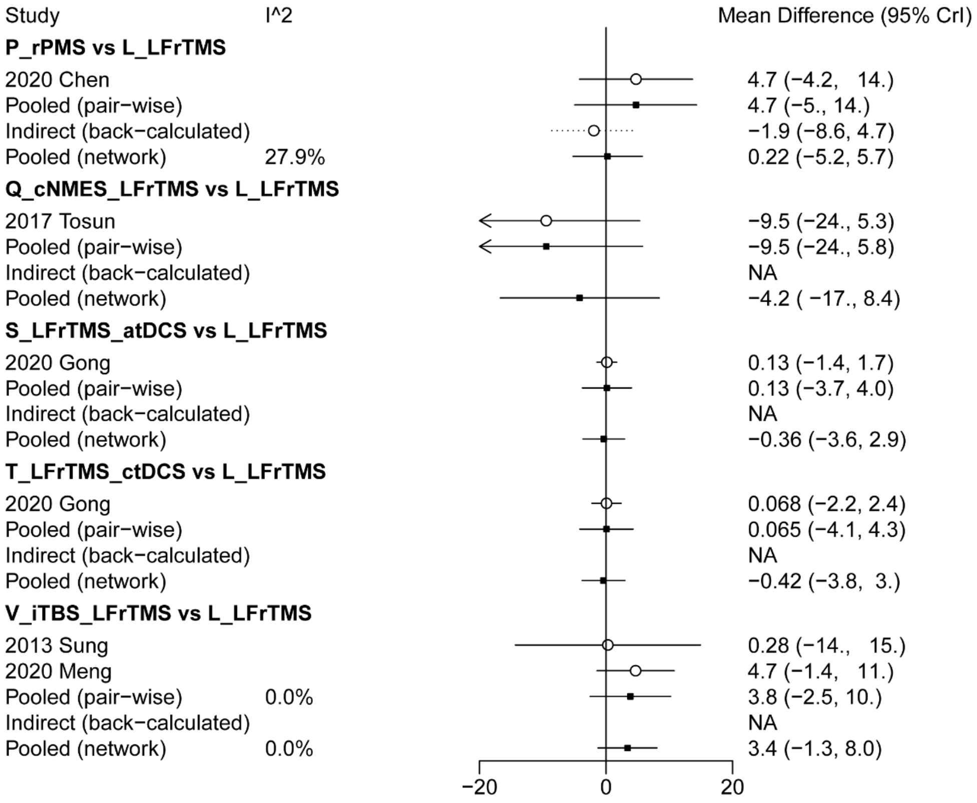
Study
I^2
Mean Difference (95% CrI)
P_rPMS vs L_LFrTMS
2020 Chen 4.7 (−4.2, 14.)
Pooled (pair−wise) 4.7 (−5., 14.)
Indirect (back−calculated) −1.9 (−8.6, 4.7)
Pooled (network) 27.9% 0.22 (−5.2, 5.7)
Q_cNMES_LFrTMS vs L_LFrTMS
2017 Tosun −9.5 (−24., 5.3)
Pooled (pair−wise) −9.5 (−24., 5.8)
Indirect (back−calculated) NA
Pooled (network) −4.2 (−17., 8.4)
S_LFrTMS_atDCS vs L_LFrTMS
2020 Gong 0.13 (−1.4, 1.7)
Pooled (pair−wise) 0.13 (−3.7, 4.0)
Indirect (back−calculated) NA
Pooled (network) −0.36 (−3.6, 2.9)
T_LFrTMS_ctDCS vs L_LFrTMS
2020 Gong 0.068 (−2.2, 2.4)
Pooled (pair−wise) 0.065 (−4.1, 4.3)
Indirect (back−calculated) NA
Pooled (network) −0.42 (−3.8, 3.)
V_iTBS_LFrTMS vs L_LFrTMS
2013 Sung 0.28 (−14., 15.)
2020 Meng 4.7 (−1.4, 11.)
Pooled (pair−wise) 0.0% 3.8 (−2.5, 10.)
Indirect (back−calculated) NA
Pooled (network) 0.0% 3.4 (−1.3, 8.0)
−20
0
20

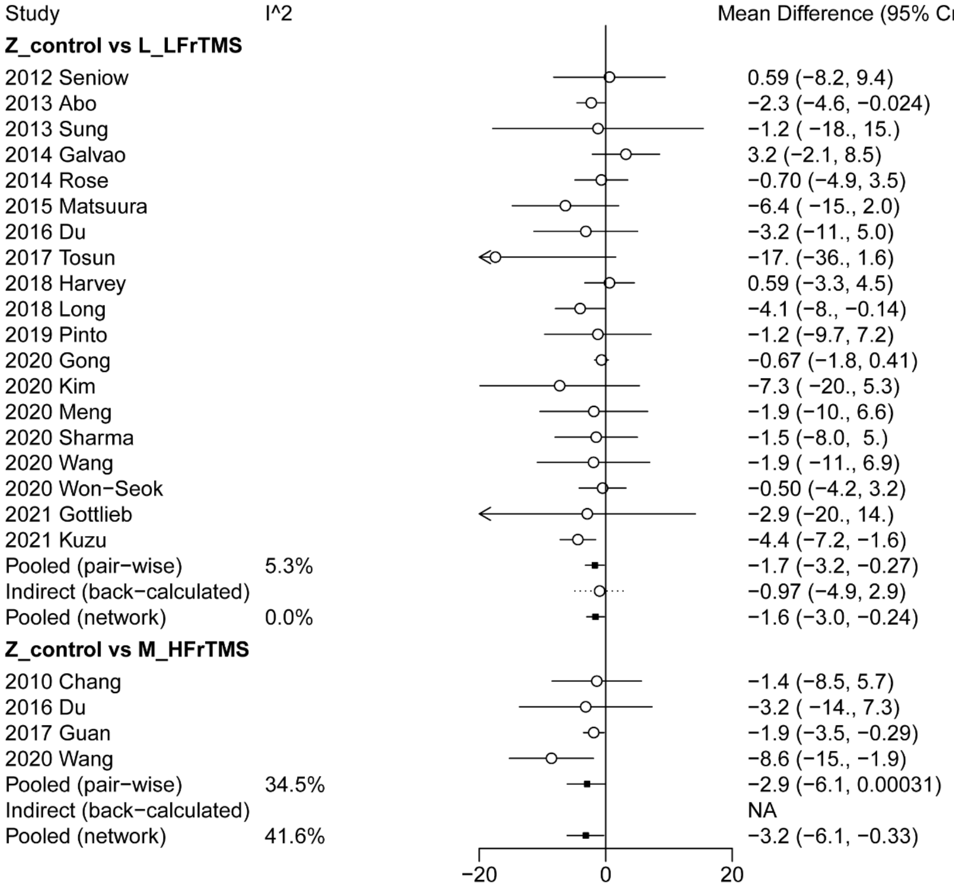
Study
I^2
Mean Difference (95% CrI)
Z_control vs L_LFrTMS
2012 Seniow 0.59 (−8.2, 9.4)
2013 Abo −2.3 (−4.6, −0.024)
2013 Sung −1.2 (−18., 15.)
2014 Galvao 3.2 (−2.1, 8.5)
2014 Rose −0.70 (−4.9, 3.5)
2015 Matsuura −6.4 (−15., 2.0)
2016 Du −3.2 (−11., 5.0)
2017 Tosun −17. (−36., 1.6)
2018 Harvey 0.59 (−3.3, 4.5)
2018 Long −4.1 (−8., −0.14)
2019 Pinto −1.2 (−9.7, 7.2)
2020 Gong −0.67 (−1.8, 0.41)
2020 Kim −7.3 (−20., 5.3)
2020 Meng −1.9 (−10., 6.6)
2020 Sharma −1.5 (−8.0, 5.)
2020 Wang −1.9 (−11., 6.9)
2020 Won−Seok −0.50 (−4.2, 3.2)
2021 Gottlieb −2.9 (−20., 14.)
2021 Kuzu −4.4 (−7.2, −1.6)
Pooled (pair−wise) 5.3% −1.7 (−3.2, −0.27)
Indirect (back−calculated) −0.97 (−4.9, 2.9)
Pooled (network) 0.0% −1.6 (−3.0, −0.24)
Z_control vs M_HFrTMS
2010 Chang −1.4 (−8.5, 5.7)
2016 Du −3.2 (−14., 7.3)
2017 Guan −1.9 (−3.5, −0.29)
2020 Wang −8.6 (−15., −1.9)
Pooled (pair−wise) 34.5% −2.9 (−6.1, 0.00031)
Indirect (back−calculated) NA
Pooled (network) 41.6% −3.2 (−6.1, −0.33)
−20
0
20

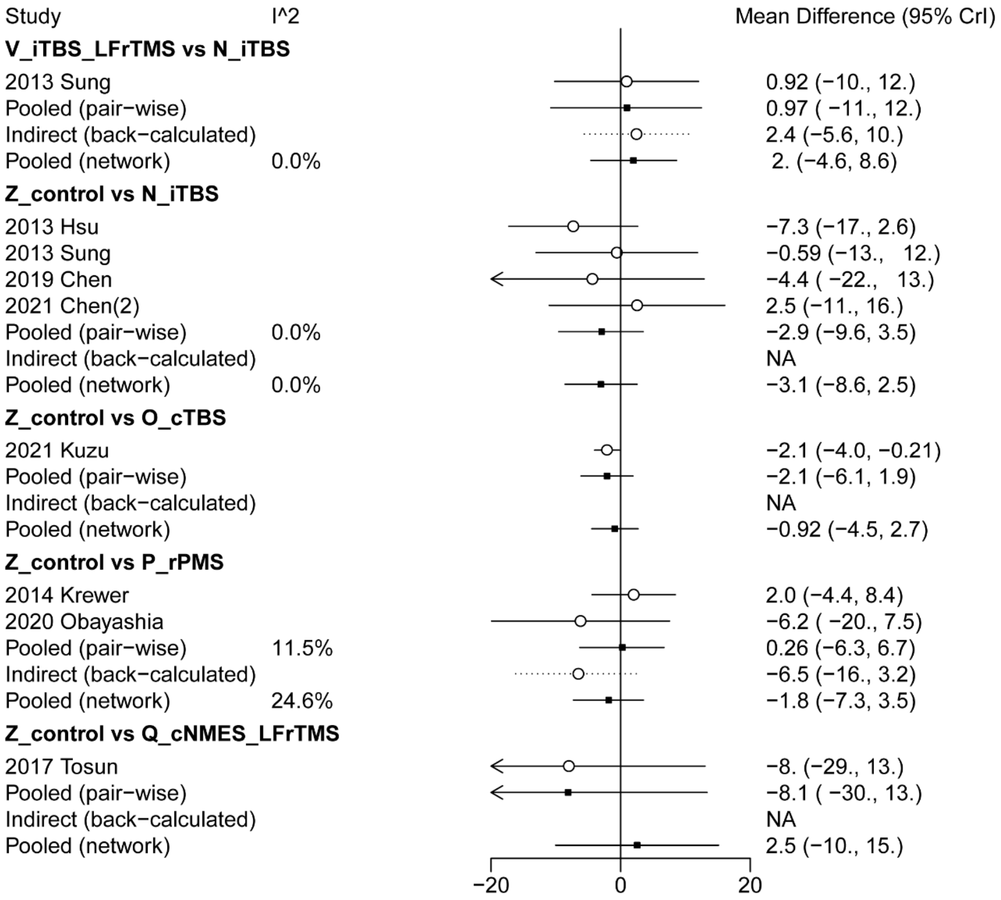
Study
I^2
Mean Difference (95% CrI)
V_iTBS_LFrTMS vs N_iTBS
2013 Sung 0.92 (−10., 12.)
Pooled (pair−wise) 0.97 (−11., 12.)
Indirect (back−calculated) 2.4 (−5.6, 10.)
Pooled (network) 0.0% 2. (−4.6, 8.6)
Z_control vs N_iTBS
2013 Hsu −7.3 (−17., 2.6)
2013 Sung −0.59 (−13., 12.)
2019 Chen −4.4 (−22., 13.)
2021 Chen(2) 2.5 (−11., 16.)
Pooled (pair−wise) 0.0% −2.9 (−9.6, 3.5)
Indirect (back−calculated) NA
Pooled (network) 0.0% −3.1 (−8.6, 2.5)
Z_control vs O_cTBS
2021 Kuzu −2.1 (−4.0, −0.21)
Pooled (pair−wise) −2.1 (−6.1, 1.9)
Indirect (back−calculated) NA
Pooled (network) −0.92 (−4.5, 2.7)
Z_control vs P_rPMS
2014 Krewer 2.0 (−4.4, 8.4)
2020 Obayashia −6.2 (−20., 7.5)
Pooled (pair−wise) 11.5% 0.26 (−6.3, 6.7)
Indirect (back−calculated) −6.5 (−16., 3.2)
Pooled (network) 24.6% −1.8 (−7.3, 3.5)
Z_control vs Q_cNMES_LFrTMS
2017 Tosun −8. (−29., 13.)
Pooled (pair−wise) −8.1 (−30., 13.)
Indirect (back−calculated) NA
Pooled (network) 2.5 (−10., 15.)
−20
0
20

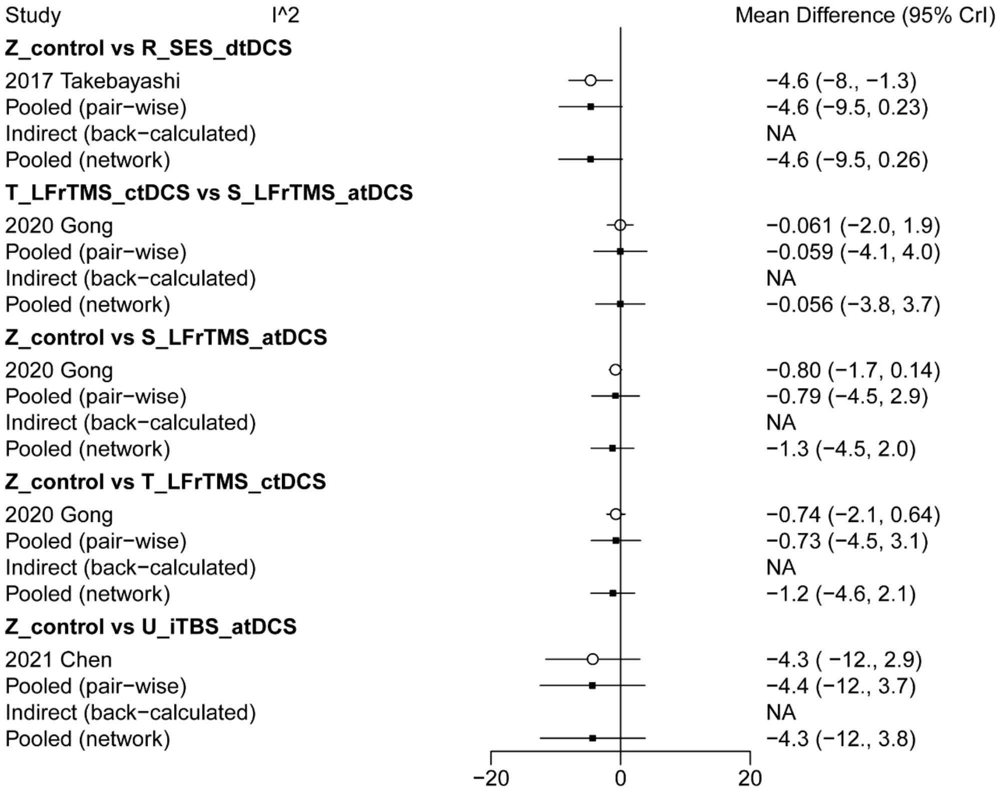
Study
I^2
Mean Difference (95% CrI)
Z_control vs R_SES_dtDCS
2017 Takebayashi −4.6 (−8., −1.3)
Pooled (pair−wise) −4.6 (−9.5, 0.23)
Indirect (back−calculated) NA
Pooled (network) −4.6 (−9.5, 0.26)
T_LFrTMS_ctDCS vs S_LFrTMS_atDCS
2020 Gong −0.061 (−2.0, 1.9)
Pooled (pair−wise) −0.059 (−4.1, 4.0)
Indirect (back−calculated) NA
Pooled (network) −0.056 (−3.8, 3.7)
Z_control vs S_LFrTMS_atDCS
2020 Gong −0.80 (−1.7, 0.14)
Pooled (pair−wise) −0.79 (−4.5, 2.9)
Indirect (back−calculated) NA
Pooled (network) −1.3 (−4.5, 2.0)
Z_control vs T_LFrTMS_ctDCS
2020 Gong −0.74 (−2.1, 0.64)
Pooled (pair−wise) −0.73 (−4.5, 3.1)
Indirect (back−calculated) NA
Pooled (network) −1.2 (−4.6, 2.1)
Z_control vs U_iTBS_atDCS
2021 Chen −4.3 (−12., 2.9)
Pooled (pair−wise) −4.4 (−12., 3.7)
Indirect (back−calculated) NA
Pooled (network) −4.3 (−12., 3.8)
−20
0
20

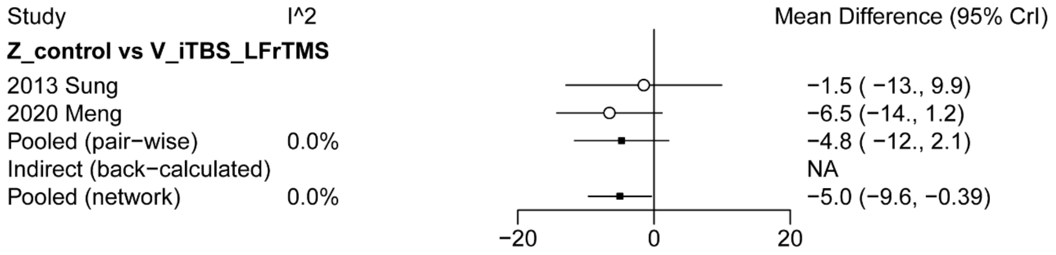
Study
I^2
Mean Difference (95% CrI)
Z_control vs V_iTBS_LFrTMS
2013 Sung
−1.5 (−13., 9.9)
2020 Meng
−6.5 (−14., 1.2)
Pooled (pair−wise)
0.0%
−4.8 (−12., 2.1)
Indirect (back−calculated)
NA
Pooled (network)
0.0%
−5.0 (−9.6, −0.39)
−20
0
20

N. Network local consistent analysis and heterogeneity analysis of FMA-UE 1 month

Consistency

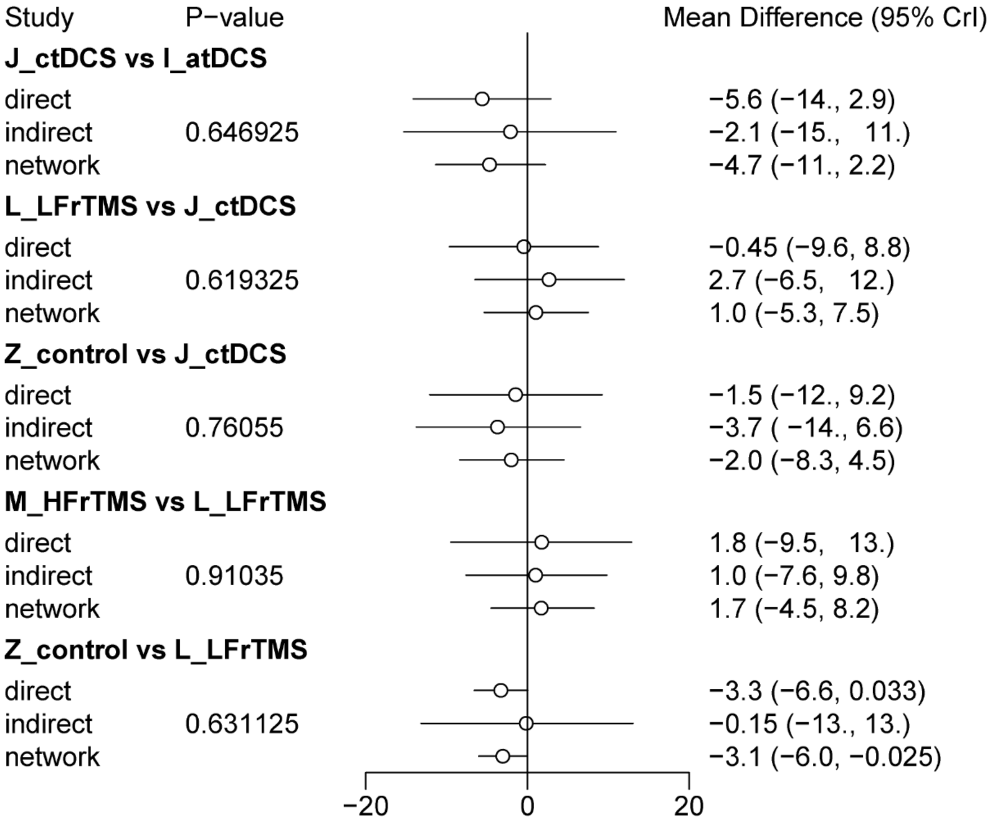

Heterogeneity

	t1	t2	i2.pair	i2.cons	incons.p
1	A_VNS	Z_control	NA	NA	NA
2	B_taVNS	Z_control	NA	NA	NA
3	C_ENMES	D_cNMES	NA	NA	NA
4	C_ENMES	F_SES	0.00000	0.0000000	NA
5	D_cNMES	E_FES	NA	NA	NA
6	D_cNMES	F_SES	NA	NA	NA
7	F_SES	Z_control	NA	NA	NA
8	G_MCS	Z_control	72.97526	72.5833513	NA
9	H_dtDCS	Z_control	NA	NA	NA
10	I_atDCS	J_ctDCS	NA	0.0000000	0.7320204
11	I_atDCS	Z_control	0.00000	0.0000000	NA
12	J_ctDCS	L_LFrTMS	NA	0.0000000	0.6604353
13	J_ctDCS	Z_control	0.00000	0.0000000	0.8657721
14	K_drTMS	Z_control	NA	NA	NA
15	L_LFrTMS	M_HFrTMS	NA	0.0000000	0.9949266
16	L_LFrTMS	N_cTBS	NA	NA	NA
17	L_LFrTMS	S_LFrTMS_atDCS	NA	NA	NA
18	L_LFrTMS	T_LFrTMS_ctDCS	NA	NA	NA
19	L_LFrTMS	Z_control	84.70032	81.6190225	0.8472052
20	M_HFrTMS	Z_control	10.71166	0.4117639	NA
21	N_cTBS	Z_control	NA	NA	NA
22	S_LFrTMS_atDCS	T_LFrTMS_ctDCS	NA	NA	NA
23	S_LFrTMS_atDCS	Z_control	NA	NA	NA
24	T_LFrTMS_ctDCS	Z_control	NA	NA	NA
Global I-squared: ------------------------ i2.pair = 43.61841; i2.cons = 33.9865					

Study	I^2	Mean Difference (95% CrI)
**Z_control vs A_VNS**		
2018 Kimberley		−2.2 (−11., 6.4)
Pooled (pair−wise)		−2.2 (−14., 9.2)
Indirect (back−calculated)		NA
Pooled (network)		−2.2 (−13., 8.9)
**Z_control vs B_nVNS**		
2020 Wu		−4.3 (−5.7, −3.)
Pooled (pair−wise)		−4.3 (−12., 3.4)
Indirect (back−calculated)		NA
Pooled (network)		−4.3 (−11., 2.9)
**D_cNMES vs C_ENMES**		
2016 Wilson		−0.40 (−7.7, 6.9)
Pooled (pair−wise)		−0.40 (−11., 10.)
Indirect (back−calculated)		NA
Pooled (network)		−1.2 (−10., 7.6)
**F_SES vs C_ENMES**		
2016 Wilson		1.9 (−5.8, 9.6)
2017 Chuang		−3.0 (−14., 8.4)
Pooled (pair−wise)	0.0%	0.042 (−8.4, 8.4)
Indirect (back−calculated)		NA
Pooled (network)	0.0%	0.34 (−7.4, 7.9)
**E_FES vs D_cNMES**		
2011 Knutson		0.72 (−6.2, 7.7)
Pooled (pair−wise)		0.69 (−9.4, 11.)
Indirect (back−calculated)		NA
Pooled (network)		0.70 (−9.2, 10.)

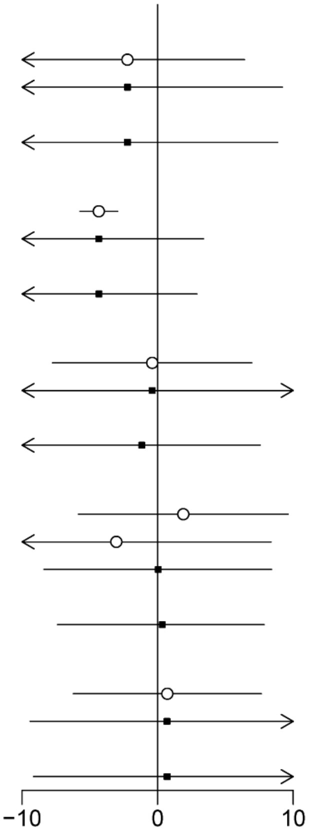

Study	I^2	Mean Difference (95% CrI)
**F_SES vs D_cNMES**		
2016 Wilson		2.3 (−5.0, 9.6)
Pooled (pair−wise)		2.3 (−8.1, 13.)
Indirect (back−calculated)		NA
Pooled (network)		1.5 (−7.3, 10.)
**Z_control vs F_SES**		
2016 Carrico		−5.9 (−12., 0.19)
Pooled (pair−wise)		−5.9 (−16., 3.7)
Indirect (back−calculated)		NA
Pooled (network)		−5.9 (−15., 3.3)
**Z_control vs G_MCS**		
2008 Huang		−3.6 (−7.2, 0.097)
2008 Levy		−3.6 (−7.1, −0.082)
2016 Levy		−0.30 (−1.8, 1.2)
Pooled (pair−wise)	73.0%	−2.3 (−7.1, 2.3)
Indirect (back−calculated)		NA
Pooled (network)	72.6%	−2.3 (−6.8, 2.1)
**Z_control vs H_dtDCS**		
2015 Ang		0.40 (−4.2, 5.0)
Pooled (pair−wise)		0.42 (−8.4, 9.2)
Indirect (back−calculated)		NA
Pooled (network)		0.37 (−8., 8.7)
**J_ctDCS vs I_atDCS**		
2015 Rocha		−5.6 (−9.4, −1.8)
Pooled (pair−wise)		−5.6 (−14., 2.9)
Indirect (back−calculated)		−3.2 (−14., 7.6)
Pooled (network)	0.0%	−4.7 (−11., 2.1)

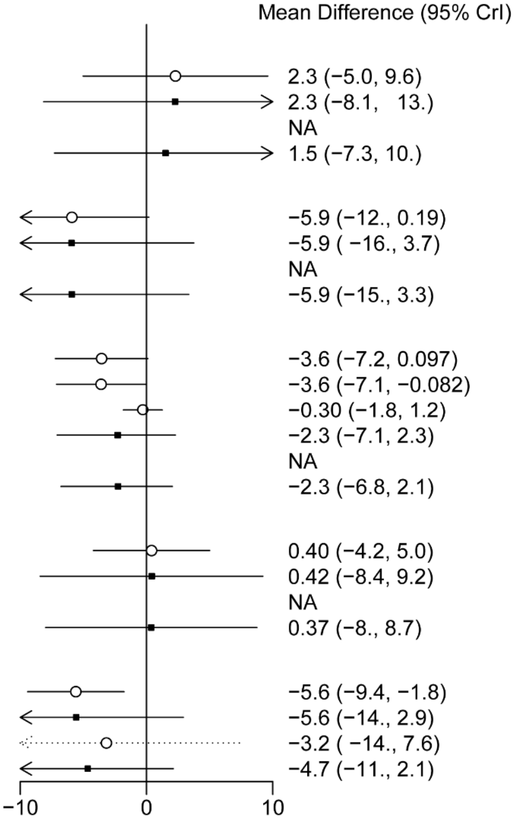

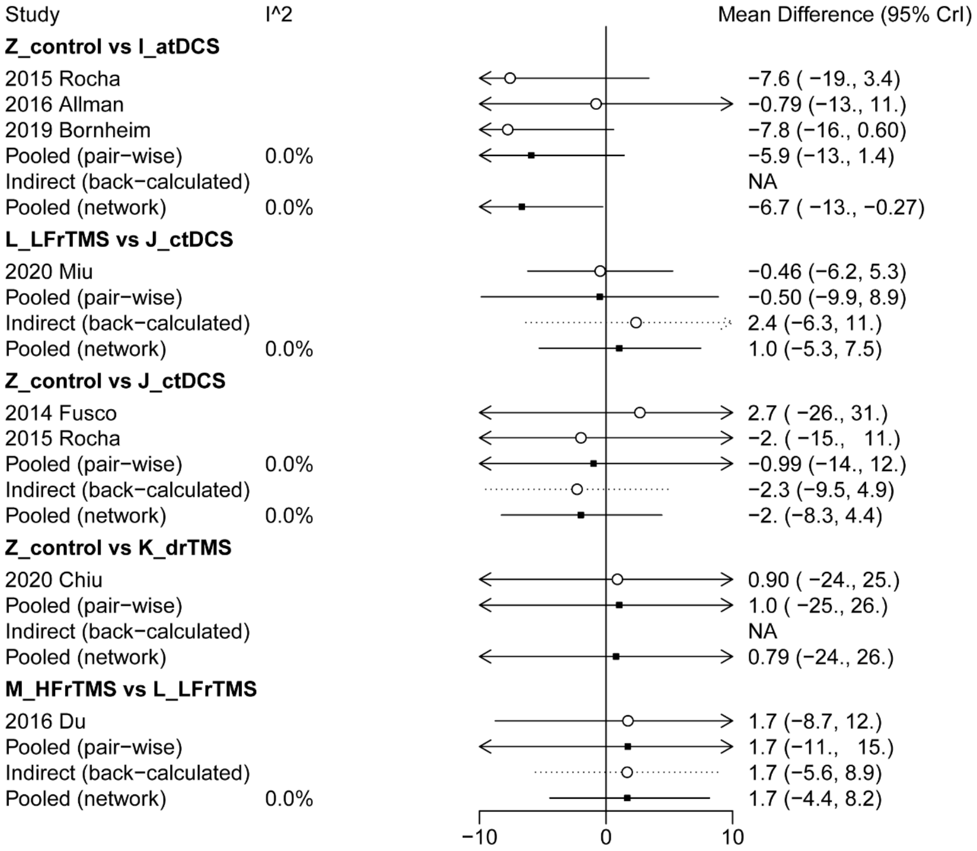
Study
I^2
Mean Difference (95% CrI)
Z_control vs I_atDCS
2015 Rocha −7.6 (−19., 3.4)
2016 Allman −0.79 (−13., 11.)
2019 Bornheim −7.8 (−16., 0.60)
Pooled (pair−wise) 0.0% −5.9 (−13., 1.4)
Indirect (back−calculated) NA
Pooled (network) 0.0% −6.7 (−13., −0.27)
L_LFrTMS vs J_ctDCS
2020 Miu −0.46 (−6.2, 5.3)
Pooled (pair−wise) −0.50 (−9.9, 8.9)
Indirect (back−calculated) 2.4 (−6.3, 11.)
Pooled (network) 0.0% 1.0 (−5.3, 7.5)
Z_control vs J_ctDCS
2014 Fusco 2.7 (−26., 31.)
2015 Rocha −2. (−15., 11.)
Pooled (pair−wise) 0.0% −0.99 (−14., 12.)
Indirect (back−calculated) −2.3 (−9.5, 4.9)
Pooled (network) 0.0% −2. (−8.3, 4.4)
Z_control vs K_drTMS
2020 Chiu 0.90 (−24., 25.)
Pooled (pair−wise) 1.0 (−25., 26.)
Indirect (back−calculated) NA
Pooled (network) 0.79 (−24., 26.)
M_HFrTMS vs L_LFrTMS
2016 Du 1.7 (−8.7, 12.)
Pooled (pair−wise) 1.7 (−11., 15.)
Indirect (back−calculated) 1.7 (−5.6, 8.9)
Pooled (network) 0.0% 1.7 (−4.4, 8.2)
−10
0
10

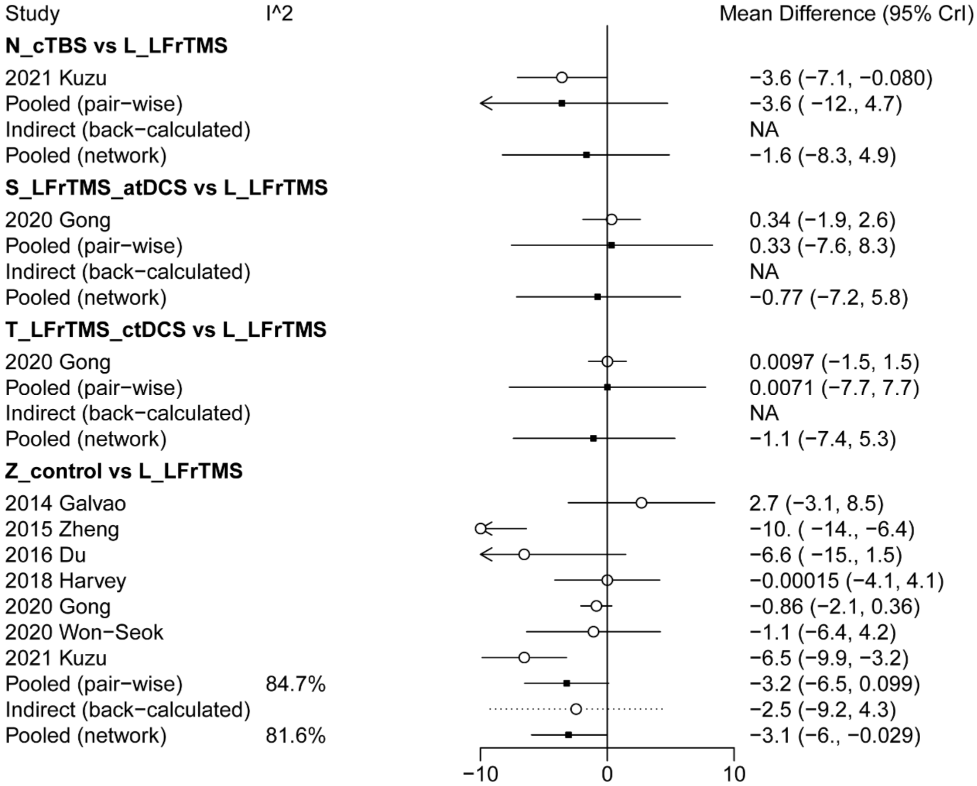
Study
I^2
Mean Difference (95% CrI)
N_cTBS vs L_LFrTMS
2021 Kuzu −3.6 (−7.1, −0.080)
Pooled (pair−wise) −3.6 (−12., 4.7)
Indirect (back−calculated) NA
Pooled (network) −1.6 (−8.3, 4.9)
S_LFrTMS_atDCS vs L_LFrTMS
2020 Gong 0.34 (−1.9, 2.6)
Pooled (pair−wise) 0.33 (−7.6, 8.3)
Indirect (back−calculated) NA
Pooled (network) −0.77 (−7.2, 5.8)
T_LFrTMS_ctDCS vs L_LFrTMS
2020 Gong 0.0097 (−1.5, 1.5)
Pooled (pair−wise) 0.0071 (−7.7, 7.7)
Indirect (back−calculated) NA
Pooled (network) −1.1 (−7.4, 5.3)
Z_control vs L_LFrTMS
2014 Galvao 2.7 (−3.1, 8.5)
2015 Zheng −10. (−14., −6.4)
2016 Du −6.6 (−15., 1.5)
2018 Harvey −0.00015 (−4.1, 4.1)
2020 Gong −0.86 (−2.1, 0.36)
2020 Won−Seok −1.1 (−6.4, 4.2)
2021 Kuzu −6.5 (−9.9, −3.2)
Pooled (pair−wise) 84.7% −3.2 (−6.5, 0.099)
Indirect (back−calculated) −2.5 (−9.2, 4.3)
Pooled (network) 81.6% −3.1 (−6., −0.029)
−10
0
10

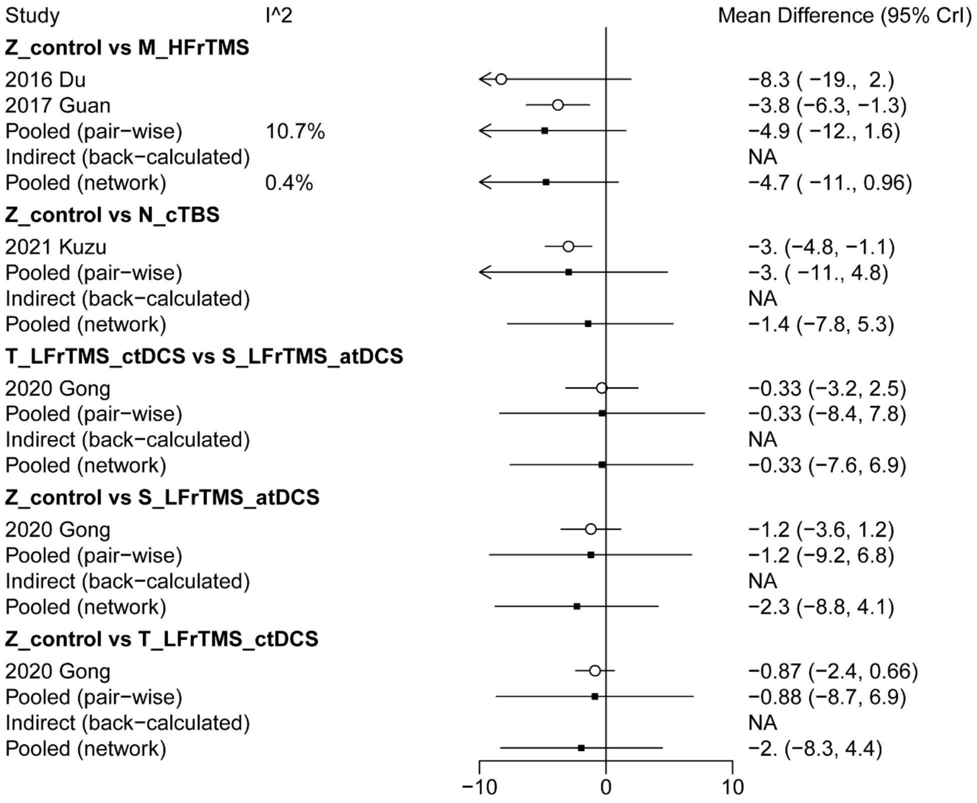
Study
I^2
Mean Difference (95% CrI)
Z_control vs M_HFrTMS
2016 Du −8.3 (−19., 2.)
2017 Guan −3.8 (−6.3, −1.3)
Pooled (pair−wise) 10.7% −4.9 (−12., 1.6)
Indirect (back−calculated) NA
Pooled (network) 0.4% −4.7 (−11., 0.96)
Z_control vs N_cTBS
2021 Kuzu −3. (−4.8, −1.1)
Pooled (pair−wise) −3. (−11., 4.8)
Indirect (back−calculated) NA
Pooled (network) −1.4 (−7.8, 5.3)
T_LFrTMS_ctDCS vs S_LFrTMS_atDCS
2020 Gong −0.33 (−3.2, 2.5)
Pooled (pair−wise) −0.33 (−8.4, 7.8)
Indirect (back−calculated) NA
Pooled (network) −0.33 (−7.6, 6.9)
Z_control vs S_LFrTMS_atDCS
2020 Gong −1.2 (−3.6, 1.2)
Pooled (pair−wise) −1.2 (−9.2, 6.8)
Indirect (back−calculated) NA
Pooled (network) −2.3 (−8.8, 4.1)
Z_control vs T_LFrTMS_ctDCS
2020 Gong −0.87 (−2.4, 0.66)
Pooled (pair−wise) −0.88 (−8.7, 6.9)
Indirect (back−calculated) NA
Pooled (network) −2. (−8.3, 4.4)
−10
0
10

O. Network local consistent analysis and heterogeneity analysis of FMA-UE 3 months

Consistency

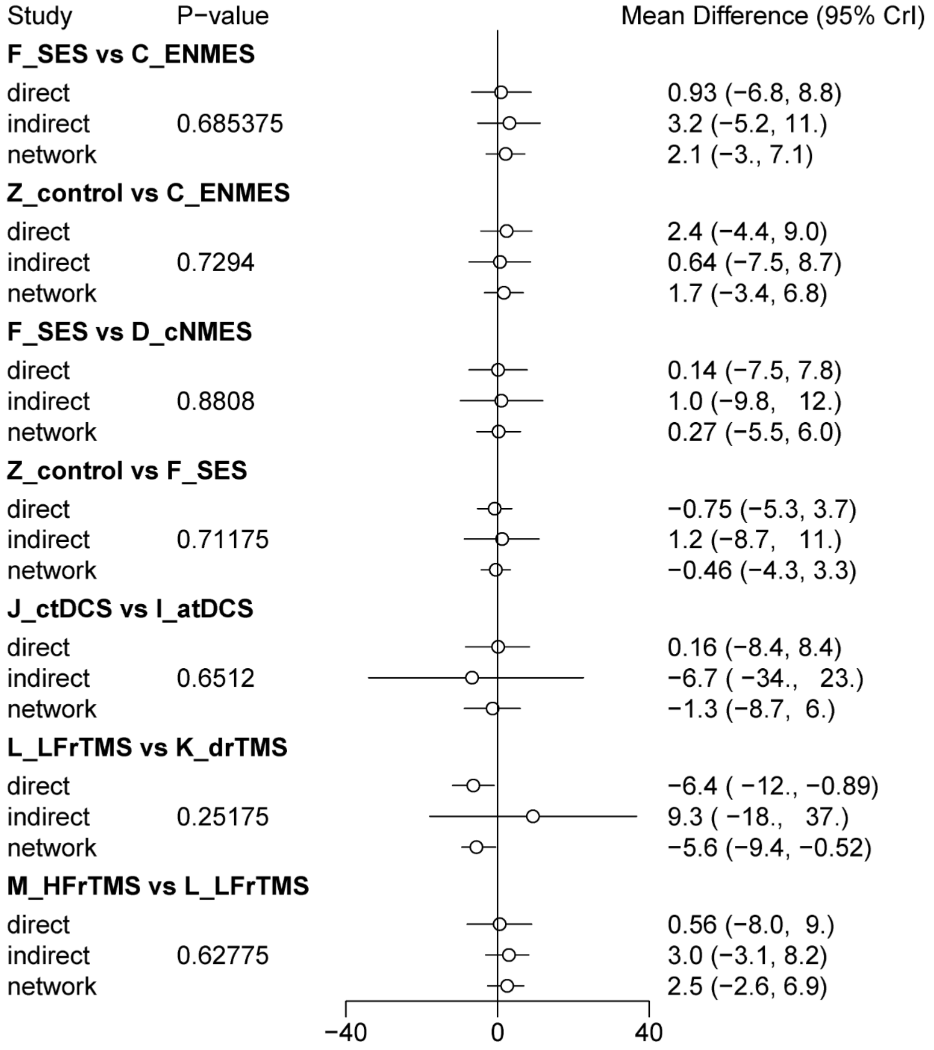

Heterogeneity

	t1	t2	i2.pair	i2.cons	incons.p
1	A_VNS	Z_control	0.000000	0.000000	NA
2	B_taVNS	Z_control	NA	NA	NA
3	C_ENMES	D_cNMES	0.000000	0.000000	NA
4	C_ENMES	F_SES	NA	0.000000	0.7499071
5	C_ENMES	Z_control	0.000000	0.000000	0.7307332
6	D_cNMES	E_FES	NA	NA	NA
7	D_cNMES	F_SES	NA	0.000000	0.9402436
8	F_SES	Z_control	0.000000	0.000000	0.8436701
9	G_MCS	Z_control	55.091326	54.498515	NA
10	I_atDCS	J_ctDCS	NA	0.000000	0.6424364
11	I_atDCS	Z_control	0.000000	0.000000	NA
12	J_ctDCS	Z_control	0.000000	0.000000	NA
13	K_drTMS	L_LFrTMS	NA	0.000000	0.6443444
14	K_drTMS	Z_control	7.050626	9.797828	NA
15	L_LFrTMS	M_HFrTMS	NA	0.000000	0.6777999
16	L_LFrTMS	N_iTBS	NA	NA	NA
17	L_LFrTMS	Z_control	63.510931	63.340200	NA
18	M_HFrTMS	Z_control	0.000000	0.000000	NA
19	N_iTBS	Z_control	NA	NA	NA
Global I-squared: ------------------------- i2.pair = 6.652957; i2.cons = 0					

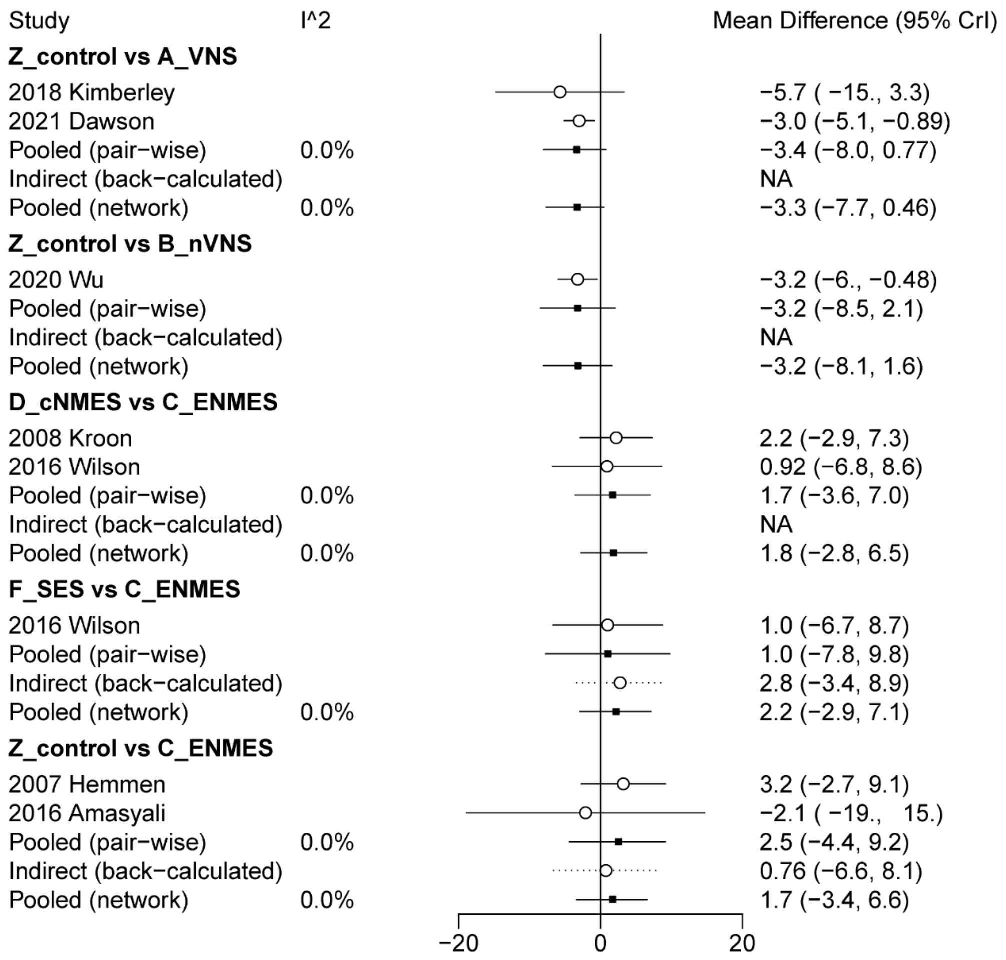
Study
I^2
Mean Difference (95% CrI)
Z_control vs A_VNS
2018 Kimberley −5.7 (−15., 3.3)
2021 Dawson −3.0 (−5.1, −0.89)
Pooled (pair−wise) 0.0% −3.4 (−8.0, 0.77)
Indirect (back−calculated) NA
Pooled (network) 0.0% −3.3 (−7.7, 0.46)
Z_control vs B_nVNS
2020 Wu −3.2 (−6., −0.48)
Pooled (pair−wise) −3.2 (−8.5, 2.1)
Indirect (back−calculated) NA
Pooled (network) −3.2 (−8.1, 1.6)
D_cNMES vs C_ENMES
2008 Kroon 2.2 (−2.9, 7.3)
2016 Wilson 0.92 (−6.8, 8.6)
Pooled (pair−wise) 0.0% 1.7 (−3.6, 7.0)
Indirect (back−calculated) NA
Pooled (network) 0.0% 1.8 (−2.8, 6.5)
F_SES vs C_ENMES
2016 Wilson 1.0 (−6.7, 8.7)
Pooled (pair−wise) 1.0 (−7.8, 9.8)
Indirect (back−calculated) 2.8 (−3.4, 8.9)
Pooled (network) 0.0% 2.2 (−2.9, 7.1)
Z_control vs C_ENMES
2007 Hemmen 3.2 (−2.7, 9.1)
2016 Amasyali −2.1 (−19., 15.)
Pooled (pair−wise) 0.0% 2.5 (−4.4, 9.2)
Indirect (back−calculated) 0.76 (−6.6, 8.1)
Pooled (network) 0.0% 1.7 (−3.4, 6.6)
−20
0
20

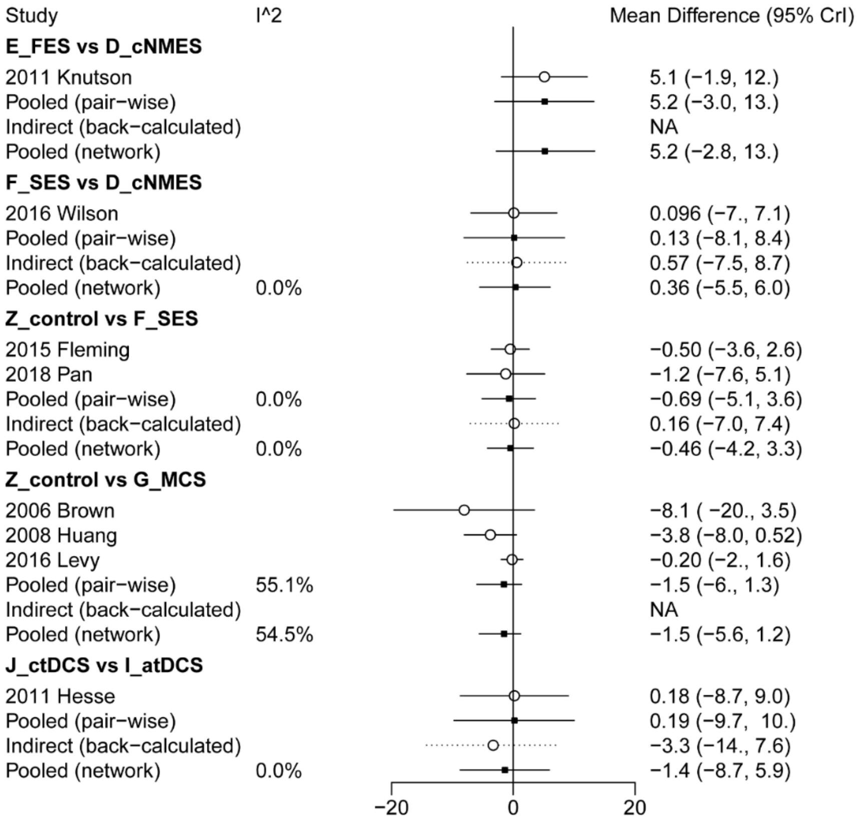
Study
I^2
Mean Difference (95% CrI)
E_FES vs D_cNMES
2011 Knutson 5.1 (−1.9, 12.)
Pooled (pair−wise) 5.2 (−3.0, 13.)
Indirect (back−calculated) NA
Pooled (network) 5.2 (−2.8, 13.)
F_SES vs D_cNMES
2016 Wilson 0.096 (−7., 7.1)
Pooled (pair−wise) 0.13 (−8.1, 8.4)
Indirect (back−calculated) 0.57 (−7.5, 8.7)
Pooled (network) 0.0% 0.36 (−5.5, 6.0)
Z_control vs F_SES
2015 Fleming −0.50 (−3.6, 2.6)
2018 Pan −1.2 (−7.6, 5.1)
Pooled (pair−wise) 0.0% −0.69 (−5.1, 3.6)
Indirect (back−calculated) 0.16 (−7.0, 7.4)
Pooled (network) 0.0% −0.46 (−4.2, 3.3)
Z_control vs G_MCS
2006 Brown −8.1 (−20., 3.5)
2008 Huang −3.8 (−8.0, 0.52)
2016 Levy −0.20 (−2., 1.6)
Pooled (pair−wise) 55.1% −1.5 (−6., 1.3)
Indirect (back−calculated) NA
Pooled (network) 54.5% −1.5 (−5.6, 1.2)
J_ctDCS vs I_atDCS
2011 Hesse 0.18 (−8.7, 9.0)
Pooled (pair−wise) 0.19 (−9.7, 10.)
Indirect (back−calculated) −3.3 (−14., 7.6)
Pooled (network) 0.0% −1.4 (−8.7, 5.9)
−20
0
20

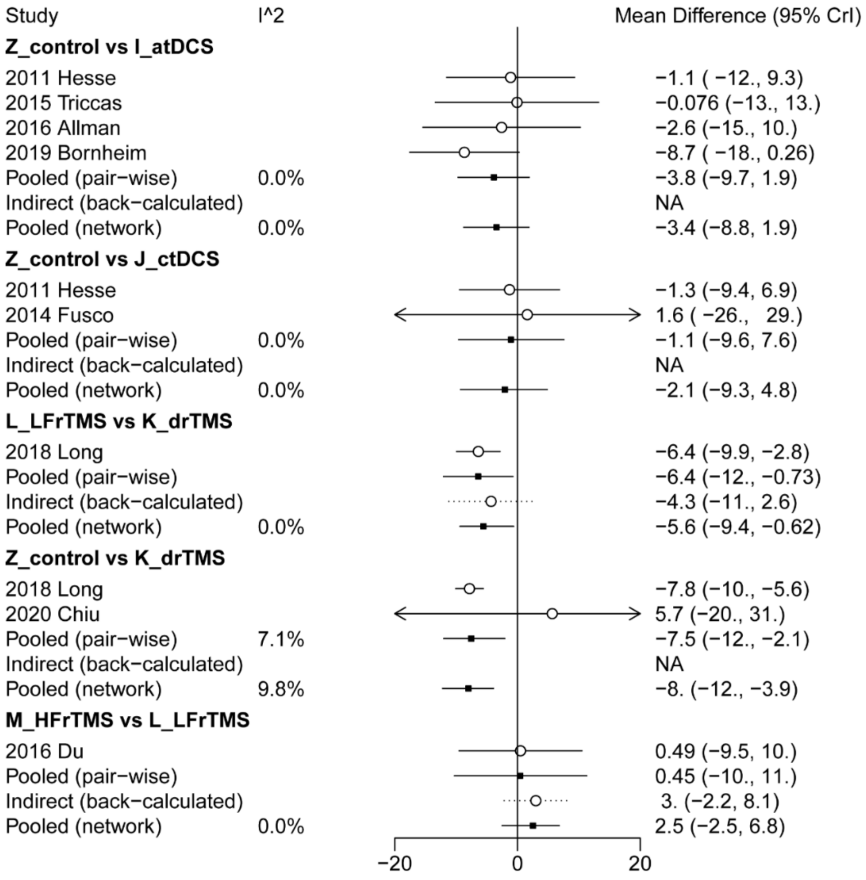
Study
I^2
Mean Difference (95% CrI)
Z_control vs I_atDCS
2011 Hesse −1.1 (−12., 9.3)
2015 Triccas −0.076 (−13., 13.)
2016 Allman −2.6 (−15., 10.)
2019 Bornheim −8.7 (−18., 0.26)
Pooled (pair−wise) 0.0% −3.8 (−9.7, 1.9)
Indirect (back−calculated) NA
Pooled (network) 0.0% −3.4 (−8.8, 1.9)
Z_control vs J_ctDCS
2011 Hesse −1.3 (−9.4, 6.9)
2014 Fusco 1.6 (−26., 29.)
Pooled (pair−wise) 0.0% −1.1 (−9.6, 7.6)
Indirect (back−calculated) NA
Pooled (network) 0.0% −2.1 (−9.3, 4.8)
L_LFrTMS vs K_drTMS
2018 Long −6.4 (−9.9, −2.8)
Pooled (pair−wise) −6.4 (−12., −0.73)
Indirect (back−calculated) −4.3 (−11., 2.6)
Pooled (network) 0.0% −5.6 (−9.4, −0.62)
Z_control vs K_drTMS
2018 Long −7.8 (−10., −5.6)
2020 Chiu 5.7 (−20., 31.)
Pooled (pair−wise) 7.1% −7.5 (−12., −2.1)
Indirect (back−calculated) NA
Pooled (network) 9.8% −8. (−12., −3.9)
M_HFrTMS vs L_LFrTMS
2016 Du 0.49 (−9.5, 10.)
Pooled (pair−wise) 0.45 (−10., 11.)
Indirect (back−calculated) 3. (−2.2, 8.1)
Pooled (network) 0.0% 2.5 (−2.5, 6.8)
−20
0
20

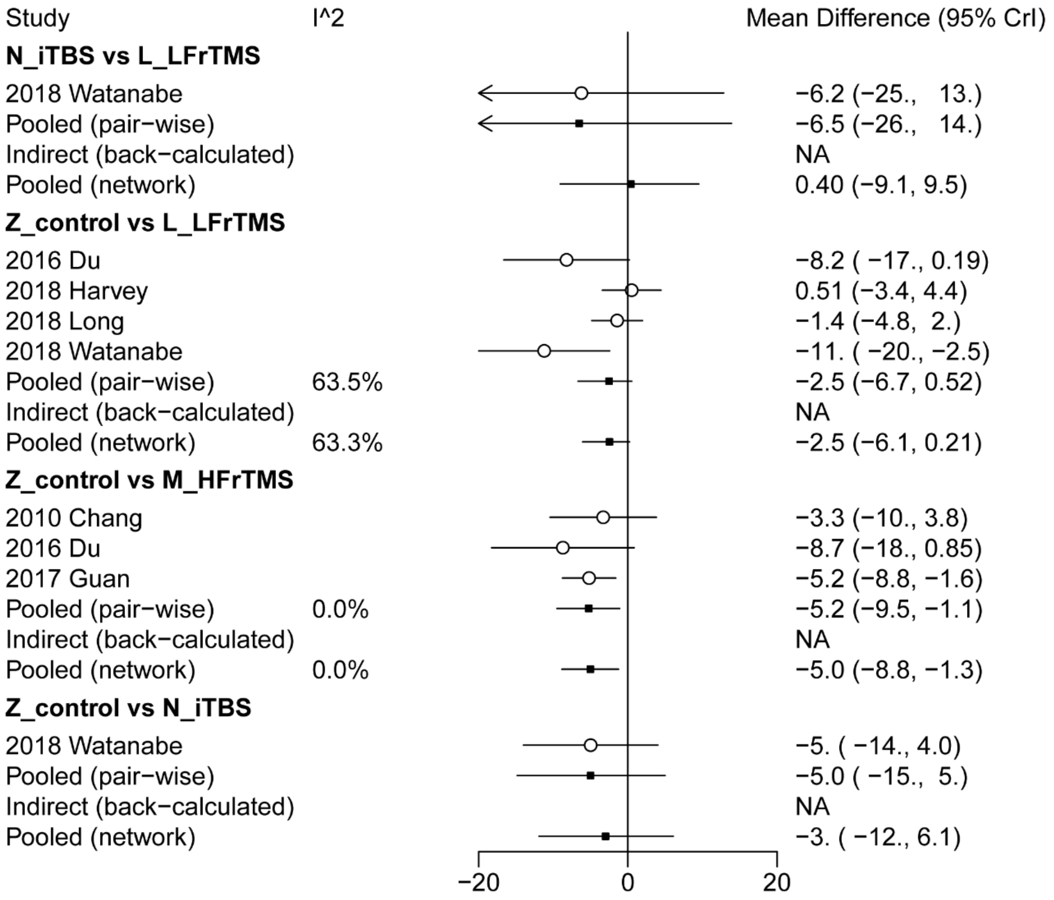
Study
I^2
Mean Difference (95% CrI)
N_iTBS vs L_LFrTMS
2018 Watanabe −6.2 (−25., 13.)
Pooled (pair−wise) −6.5 (−26., 14.)
Indirect (back−calculated) NA
Pooled (network) 0.40 (−9.1, 9.5)
Z_control vs L_LFrTMS
2016 Du −8.2 (−17., 0.19)
2018 Harvey 0.51 (−3.4, 4.4)
2018 Long −1.4 (−4.8, 2.)
2018 Watanabe −11. (−20., −2.5)
Pooled (pair−wise) 63.5% −2.5 (−6.7, 0.52)
Indirect (back−calculated) NA
Pooled (network) 63.3% −2.5 (−6.1, 0.21)
Z_control vs M_HFrTMS
2010 Chang −3.3 (−10., 3.8)
2016 Du −8.7 (−18., 0.85)
2017 Guan −5.2 (−8.8, −1.6)
Pooled (pair−wise) 0.0% −5.2 (−9.5, −1.1)
Indirect (back−calculated) NA
Pooled (network) 0.0% −5.0 (−8.8, −1.3)
Z_control vs N_iTBS
2018 Watanabe −5. (−14., 4.0)
Pooled (pair−wise) −5.0 (−15., 5.)
Indirect (back−calculated) NA
Pooled (network) −3. (−12., 6.1)
−20
0
20

P. Risk of bias for included studies

	Random sequence generation (selection bias)	Allocation concealment (selection bias)	Blinding of participants and personnel (performance bias)	Blinding of outcome assessment (detection bias)	Incomplete outcome data (attrition bias)	Selective reporting (reporting bias)	Other bias
FES vs. NMES_2011 Knutson	+	?	−	+	+	+	−
FES vs. NMES_2014 Shen	+	+	−	−	+	−	+
FES vs. NMES_2016 Knutson	+	+	−	+	+	+	+
FES vs. NMES_2017 Zhou	+	+	−	+	+	+	+
FES vs. NMES_2019 Zheng	+	+	−	?	+	+	?
FES vs. NMES_2020 Knutson	+	+	−	+	+	+	?
FES vs. SES_2008 Chan	+	+	+	+	+	+	+
iTBS_2013 Hsu	+	+	+	+	+	?	+
iTBS_2019 Chen	+	+	+	+	+	+	+
iTBS_2021 Chen (2)	+	+	+	+	+	+	+
MCS_2006 Brown	+	+	−	?	?	+	?
MCS_2008 Huang	+	+	−	−	+	+	+
MCS_2008 Levy	+	+	−	−	+	+	+
MCS_2016 Levy	+	+	−	+	+	+	+
NMES_2007 Hemmen	+	+	−	+	+	?	+
NMES_2008 Kroon	+	+	−	+	+	+	+
NMES_2011 Shindo	+	+	−	+	+	?	+
NMES_2013 Jong	+	+	−	+	+	+	+
NMES_2013 Shimodozono	+	+	+	+	+	+	+
NMES_2015 McCabe	+	+	−	+	+	+	+
NMES_2016 Amasyali	+	+	−	+	−	−	+

NMES_2016 Jeon	+	+	−	+	+	+	+
NMES vs. SES_2013 Boyaci	+	+	?	+	+	+	+
NMES vs. SES_2016 Wilson	+	+	−	+	+	+	+
NMES vs. SES_2017 Chuang	+	+	+	?	+	+	+
NMES vs.SES_2020 Yurdakul	+	+	+	+	?	−	+
nVNS_2017 Capone	+	?	+	?	+	+	+
nVNS_2020 Wu	+	+	−	+	−	?	+
rPMS_2014 Krewer	+	+	+	+	+	+	+
rPMS_2020 Obayashia	?	+	−	?	+	+	+
rPMS vs TMS_2020 Chen	+	+	−	+	+	+	+
rTMS_2010 Chang	+	?	−	+	+	+	+
rTMS_2012 Seniow	+	+	+	+	?	?	+
rTMS_2013 Abo	+	+	?	?	+	+	+
rTMS_2014 Galvao	+	+	+	−	+	+	+
rTMS_2014 Rose	+	+	+	+	+	+	+
rTMS_2015 Matsuura	+	+	+	+	+	+	+
rTMS_2015 Zheng	+	+	+	+	+	−	+
rTMS_2016 Du	+	+	+	−	+	+	+
rTMS_2017 Guan	+	+	+	+	+	+	−
rTMS_2018 Harvey	+	+	+	+	+	+	+
rTMS_2018 Long	+	+	+	+	+	+	−
rTMS_2019 Pinto	+	+	+	+	+	+	+
rTMS_2020 Chiu	+	+	+	+	+	−	+
rTMS_2020 Gong	+	+	?	+	+	+	+
rTMS_2020 Kim	+	+	−	+	+	+	+
rTMS_2020 Sharma	+	+	+	+	+	+	+
rTMS_2020 Wang	+	+	−	+	+	+	+
rTMS_2020 Won-Seok	+	+	+	+	+	+	+
rTMS_2021 Gottlieb	+	+	+	+	+	+	?
rTMS vs. cTBS vs. R_2021 Kuzu	+	+	+	+	+	+	+
rTMS vs. iTBS vs, R_2018 Watanabe	+	+	?	+	+	+	+
rTMS vs. iTBS vs. R_2020 Meng	+	+	+	+	+	+	+
rTMS vs. iTBS vs. rTMS+iTBS vs. R_2013 Sung	+	+	+	+	?	+	+
rTMS vs. NMES+rTMS vs. R_2017 Tosun	+	+	−	+	+	+	+

rTMS vs. tDCS_2020 Miu	+	+	–	+	+	+	+
SES_2015 Fleming	+	+	+	+	+	+	+
SES_2016 Carrico	+	+	+	+	+	+	+
SES_2017 Jung	+	+	–	+	+	+	+
SES_2018 Ghaziani	+	+	–	+	+	+	?
SES_2018 Pan	+	+	–	?	+	+	+
SES_2021 Alwhaibi	+	+	?	?	+	–	+
SES+dt_2017 Takebayashi	+	+	–	+	?	?	–
tDCS_2010 Lindenberg	+	+	+	+	+	+	+
tDCS_2011 Hesse	+	+	+	+	+	+	+
tDCS_2014 Fusco	+	+	+	+	+	+	+
tDCS_2014 Lee	+	+	–	+	+	+	+
tDCS_2014 Viana	+	+	+	+	+	+	+
tDCS_2015 Ang	+	+	?	?	+	+	+
tDCS_2015 Rocha	+	+	+	+	+	+	–
tDCS_2015 Triccas	+	+	+	+	+	+	+
tDCS_2016 Allman	+	+	+	+	+	+	+
tDCS_2017 Pavlova	+	+	+	?	+	?	+
tDCS_2018 Shaheiwola	+	+	?	+	+	+	+
tDCS_2019 Beaulieu	+	+	+	+	+	+	+
tDCS_2019 Bornheim	+	+	+	+	+	+	+
tDCS_2019 Edwards	+	+	+	+	+	+	+
tDCS_2019 Jin	+	+	?	?	+	+	+
tDCS_2019 Mazzoleni	+	+	–	+	+	+	+
tDCS_2019 Salazar	+	+	+	+	+	+	–
tDCS_2020 Alisar	+	+	+	+	+	+	+
tDCS_2020 Liao	+	+	+	+	?	–	+
tDCS_2020 Yao	+	+	+	–	+	+	–
tDCS_2021 Kim	+	+	+	+	+	+	+
tDCS+iTBS_2021 Chen	+	+	–	+	?	+	+
VNS_2016 Dawson	+	+	–	+	+	+	+
VNS_2018 Kimberley	+	+	+	?	+	+	+
VNS_2021 Dawson	+	+	+	+	+	+	+

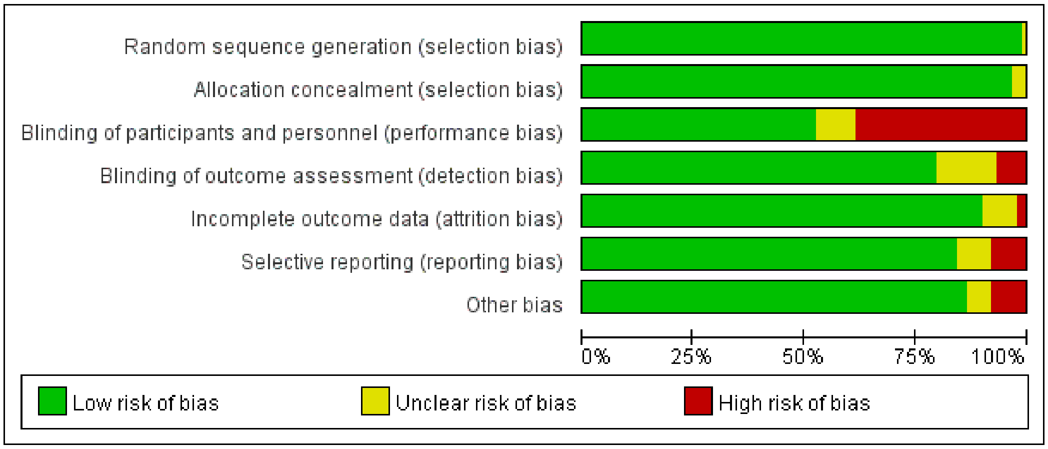
Random sequence generation (selection bias)
Allocation concealment (selection bias)
Blinding of participants and personnel (performance bias)
Blinding of outcome assessment (detection bias)
Incomplete outcome data (attrition bias)
Selective reporting (reporting bias)
Other bias
0%
25%
50%
75%
100%
Low risk of bias
Unclear risk of bias
High risk of bias

Q. Network funnel plot

LFU

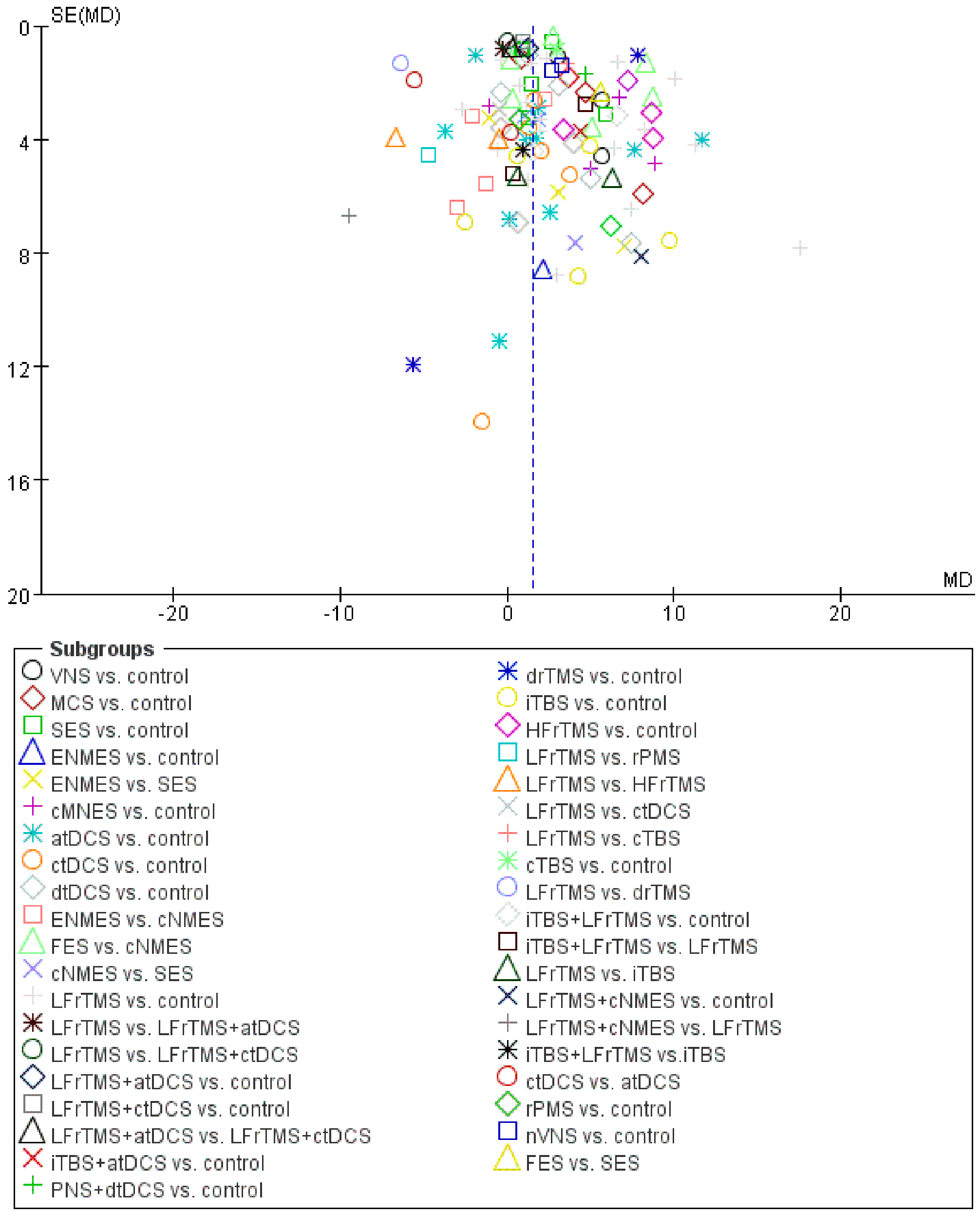

EOT

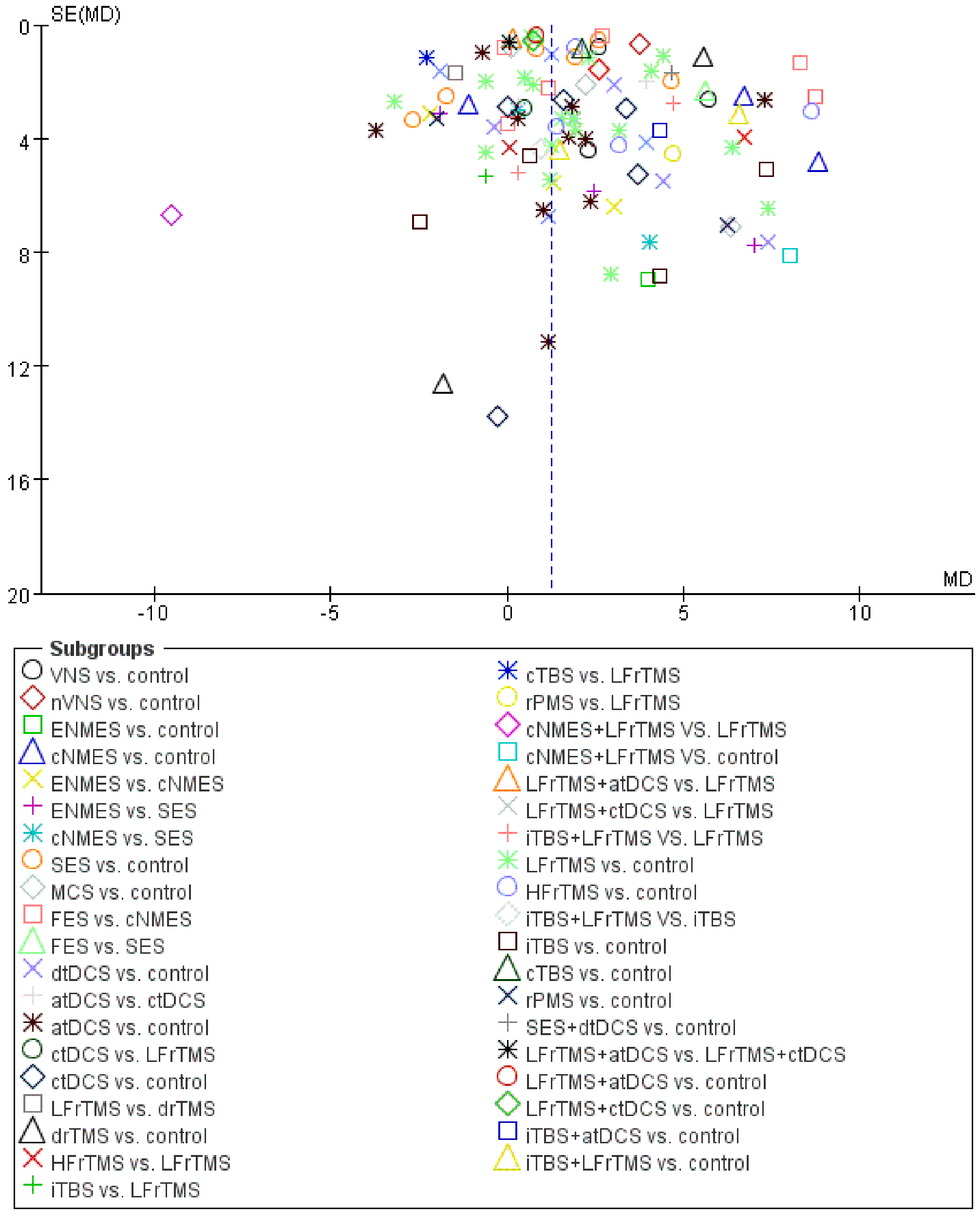
SE(MD)
0
4
8
12
16
20
MD
-10
-5
0
5
10
Subgroups
VNS vs. control
nVNS vs. control
ENMES vs. control
cNMES vs. control
ENMES vs. cNMES
ENMES vs. SES
cNMES vs. SES
SES vs. control
MCS vs. control
FES vs. cNMES
FES vs. SES
dtDCS vs. control
atDCS vs. ctDCS
atDCS vs. control
ctDCS vs. LFrTMS
ctDCS vs. control
LFrTMS vs. drTMS
drTMS vs. control
HFrTMS vs. LFrTMS
iTBS vs. LFrTMS
cTBS vs. LFrTMS
rPMS vs. LFrTMS
cNMES+LFrTMS VS. LFrTMS
cNMES+LFrTMS VS. control
LFrTMS+atDCS vs. LFrTMS
LFrTMS+ctDCS vs. LFrTMS
iTBS+LFrTMS VS. LFrTMS
LFrTMS vs. control
HFrTMS vs. control
iTBS+LFrTMS VS. iTBS
iTBS vs. control
cTBS vs. control
rPMS vs. control
SES+dtDCS vs. control
LFrTMS+atDCS vs. LFrTMS+ctDCS
LFrTMS+atDCS vs. control
LFrTMS+ctDCS vs. control
iTBS+atDCS vs. control
iTBS+LFrTMS vs. control

1 month

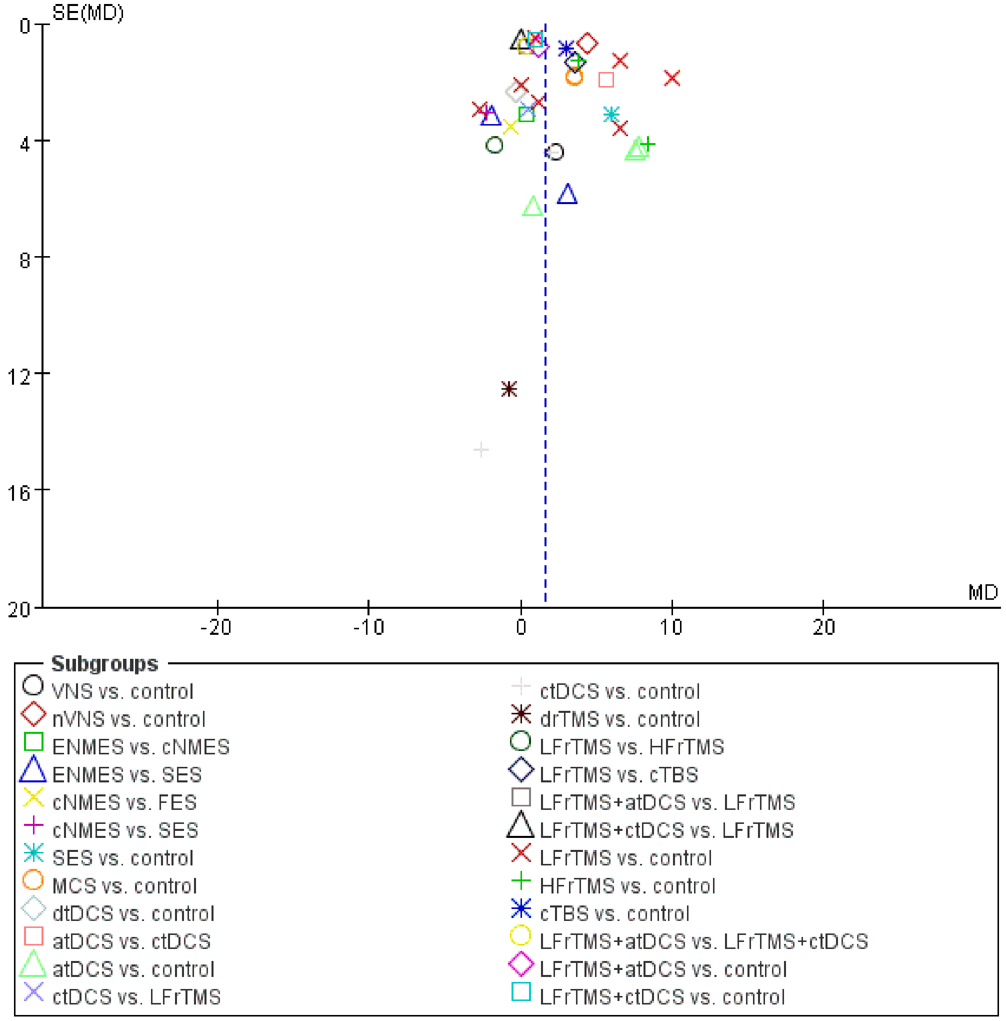
SE(MD)
0
4
8
12
16
20
MD
-20
-10
0
10
20
Subgroups
VNS vs. control
nVNS vs. control
ENMES vs. cNMES
ENMES vs. SES
cNMES vs. FES
cNMES vs. SES
SES vs. control
MCS vs. control
dtDCS vs. control
atDCS vs. ctDCS
atDCS vs. control
ctDCS vs. LFrTMS
ctDCS vs. control
drTMS vs. control
LFrTMS vs. HFrTMS
LFrTMS vs. cTBS
LFrTMS+atDCS vs. LFrTMS
LFrTMS+ctDCS vs. LFrTMS
LFrTMS vs. control
HFrTMS vs. control
cTBS vs. control
LFrTMS+atDCS vs. LFrTMS+ctDCS
LFrTMS+atDCS vs. control
LFrTMS+ctDCS vs. control

3 months

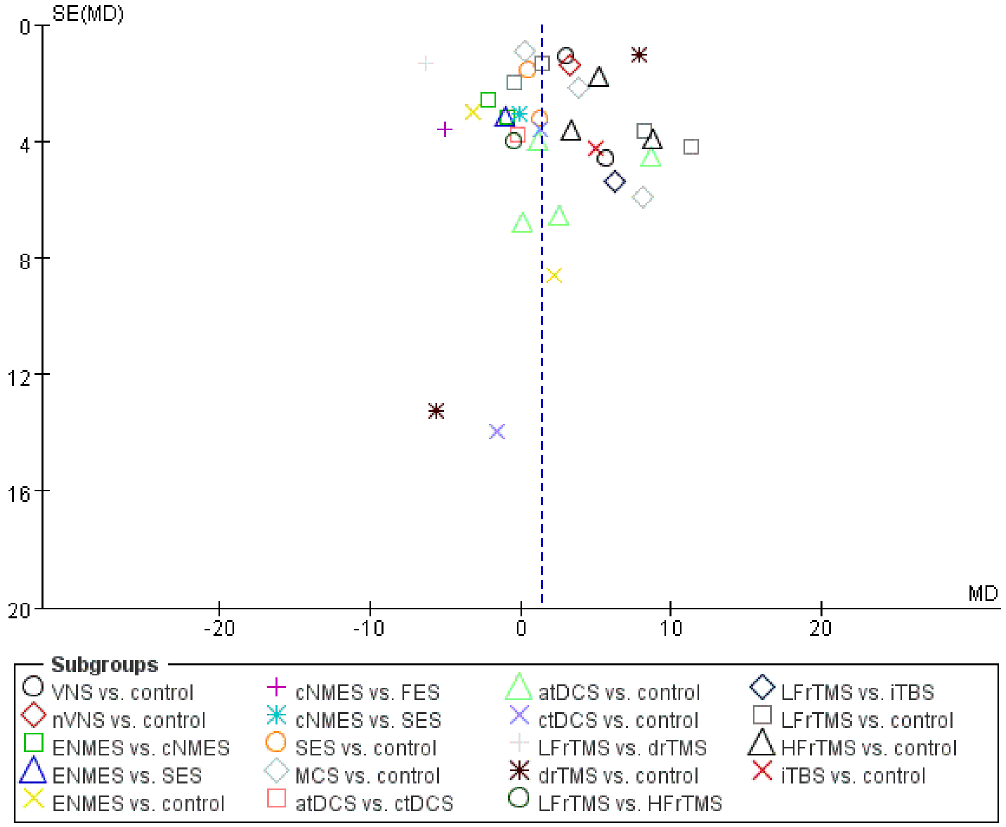
SE(MD)
0
4
8
12
16
20
-20
-10
0
10
20
MD
Subgroups
VNS vs. control
nVNS vs. control
ENMES vs. cNMES
ENMES vs. SES
ENMES vs. control
cNMES vs. FES
cNMES vs. SES
SES vs. control
MCS vs. control
atDCS vs. ctDCS
atDCS vs. control
ctDCS vs. control
LFrTMS vs. drTMS
drTMS vs. control
LFrTMS vs. HFrTMS
LFrTMS vs. iTBS
LFrTMS vs. control
HFrTMS vs. control
iTBS vs. control
